# Diet of juvenile skipjack herring *Alosa chrysochloris*: Ontogenetic shifts, predator–prey size ratios and mouth gape allometry

**DOI:** 10.1111/jfb.70390

**Published:** 2026-03-28

**Authors:** Joshua P. Egan, Peter J. Hundt, Neil P. Rude, Jason A. DeBoer, Benjamin J. Lubinski, Kristopher A. Maxson, Levi E. Solomon, Andrya L. Whitten Harris, Andrew M. Simons

**Affiliations:** ^1^ Great Lakes Fishery Commission Ann Arbor Michigan USA; ^2^ Bell Museum of Natural History University of Minnesota Saint Paul Minnesota USA; ^3^ Department of Fisheries, Wildlife and Conservation Biology University of Minnesota Saint Paul Minnesota USA; ^4^ Minnesota Department of Natural Resources Lake City Minnesota USA; ^5^ Illinois River Biological Station, Illinois Natural History Survey, Prairie Research Institute University of Illinois at Urbana‐Champaign Havana Illinois USA; ^6^ Alton Field Office, Illinois Department of Natural Resources Alton Illinois USA

**Keywords:** Clupeiformes, gut content analysis, *Hypophthalmichthys*, Mississippi River basin, silver carp, trophic ecology

## Abstract

The skipjack herring *Alosa chrysochloris* inhabits Gulf of Mexico drainages, primarily the Mississippi River Basin, and estuarine environments in the Gulf of Mexico. The trophic ecology of the skipjack herring is poorly understood, therefore we quantified the length, mouth gape and gut contents of juvenile skipjack herring from the Mississippi River (fish *n* = 6), Illinois River (fish *n* = 181) and Ohio River (fish *n* = 255) and tested for changes in diets and predator–prey size ratios during ontogeny. We identified statistically significant changes in prey type consumption during ontogeny. Aquatic insects dominated the diets of skipjack herring <40 mm standard length (SL), with zooplankton and terrestrial insects also contributing to diets. The diets of skipjack herring >40 mm SL were primarily composed of fishes. The most prevalent fish species consumed by skipjack herring were invasive bigheaded carps *Hypophthalmichthys* spp. and preliminary evidence indicated that inter‐annual variation in the consumption of bigheaded carps was driven by variation in juvenile bigheaded carp abundance. Additional research is needed to determine if the enhancement of native skipjack herring populations could be used as a tool to limit bigheaded carp population sizes. Skipjack herring SL and mouth gape were both significantly positively correlated with maximum, median and minimum sizes of prey consumed. There were allometric changes in the mouth gape of juvenile skipjack herring, with the ratio of mouth gape to SL increasing until ~80 mm SL, then gradually declining. Maximum mouth gape to SL ratios corresponded to substantial increases in the consumption of fishes, indicating that mouth gape allometry may facilitate the ontogenetic transition to piscivory in the skipjack herring. This study improves our understanding of skipjack herring ecology and provides data that can be incorporated into future ecological and evolutionary research, such as the development of food web models.

## 1 INTRODUCTION

The skipjack herring *Alosa chrysochloris* is a member of the order Clupeiformes (anchovies, sardines, herrings and relatives) that inhabits Gulf of Mexico drainages, primarily the Mississippi River Basin, and estuarine environments in the Gulf of Mexico (Bailey et al., 1954; Etnier & Starnes, 1993; O'Connell et al., 2004; Whitehead et al., 1988). Historically, the skipjack herring occurred in lotic habitats throughout much of the Mississippi River Basin, but has largely been extirpated from the upper basin in Minnesota and Wisconsin following the construction of impoundments (Carlander, 1954; Coker, 1930; Steuck et al., 2010) and is listed as endangered by the Wisconsin and Minnesota Departments of Natural Resources (Minnesota Department of Natural Resources, 2013; Wisconsin Department of Natural Resources, 2015). The skipjack herring is rheophilic and potamodromous, matures >240 mm standard length (SL) and grows to a maximum length of ~500 mm SL (Becker, 1983; Etnier & Starnes, 1993; Whitehead et al., 1988; Wolfe, 1969). The skipjack herring is a sport fish and can be highly abundant in some middle and lower Mississippi River Basin main channel fish assemblages, making it an important prey species for a variety of aquatic and terrestrial predators (Angradi et al., 2009; Barko et al., 2005; Etnier & Starnes, 1993; Holderby et al., 2014; Lorentz et al., 2006). Despite its abundance in certain areas and distinct ecology, skipjack herring trophic niches are poorly understood (McBride & Matheson, 2011; Whitehead et al., 1988).

A single study quantitatively characterized the diets of skipjack herring using specimens from the Watts Bar Reservoir on the Tennessee River (McLean et al., 1985). McLean et al. (1985) found that skipjack herring fed exclusively on fishes, primarily the pelagic threadfin shad *Dorosoma petenense*. However, McLean et al. (1985) did not report the sizes of skipjack herring included in their study, nor the sizes of prey skipjack herring consumed. Whitehead et al. (1988) and Etnier and Starnes (1993) reported consumption of both insects and fishes by skipjack herring but did not provide quantitative descriptions of diets or information about the sizes of skipjack herring examined or where they were collected. Large population sizes of skipjack herring in some locations and existing diet data suggest that they may be an important link between lower and higher trophic levels (Stein et al., 1995; Tumolo & Flinn, 2017). However, to further resolve the roles of the skipjack herring in food webs, quantitative diet studies that report sampling locations and the sizes of individuals examined are needed.

Piscivorous freshwater fishes are often zooplanktivorous during early ontogeny, and many species transition to diets containing moderately‐sized invertebrates (e.g. terrestrial insects, aquatic insects or amphipods) before becoming piscivorous (Mittelbach & Persson, 1998; Scharf et al., 2000). The size at which fishes undergo ontogenetic diet shifts varies among species and can be governed by a variety of factors. There are numerous potential drivers of ontogenetic diet shifts, including predation risk, competition and habitat use, that can vary considerably among species and ecosystems (Power, 1987; Sánchez‐Hernández et al., 2019). Ontogenetic changes in phenotypic traits also play a role in ontogenetic diet transitions. For example, mouth gape, gill raker morphology and swimming performance can regulate the sizes and types of prey consumed (Bachiller & Irigoien, 2013; Krebs & Turingan, 2003; Magnuson & Heitz, 1971). Ontogenetic changes in the morphology of feeding structures can be allometric relative to changes in body size (i.e. the sizes of feeding structures relative to body size change during ontogeny), resulting in ontogenetic differences in predator–prey size ratios and interspecific differences in predator–prey size relationships (Christensen, 1996; Luecke, 1986; Mittelbach & Persson, 1998; Scharf et al., 2000). For example, in some fishes, mouth gape has been shown to increase allometrically relative to SL during periods of ontogeny, with disproportionate increases in mouth gape coinciding with ontogenetic shifts to the consumption of larger prey (Bachiller & Irigoien, 2013; Luecke, 1986). Describing diets throughout a species' life history is critical for determining their functional roles in ecosystems and allows spurious inferences due to ontogenetic diet shifts to be avoided when making inter‐ and intraspecific comparisons of trophic ecology and evolution (Delong, 2010; Egan et al., 2018a, 2018b; Scharf et al., 2009). Characterizing ontogenetic relationships between diet and morphology can shed light on phenotypic attributes and energetic investments that facilitate ontogenetic diet shifts and allow for morphology‐based predictions of diet (Bachiller & Irigoien, 2013; Budy et al., 2005; Sánchez‐Hernández et al., 2019; Scharf et al., 2000).

Given that little is known about the feeding ecology, trophic morphology and functional roles of the skipjack herring, particularly during early ontogeny, we conducted a study on this important mesopredator with four primary objectives: (1) describe the diets of skipjack herring from large river ecosystems and characterize ontogenetic diet shifts, (2) describe predator–prey size ratios, (3) determine if the skipjack herring exhibits ontogenetic changes in predator–prey size ratios and (4) if any changes in predator–prey size ratios are identified, test the hypothesis that these changes are associated with mouth gape allometry. To accomplish these objectives, we conducted gut content analysis of skipjack herring from the Mississippi, Illinois and Ohio rivers in Illinois and measured mouth gape. We used these data to conduct a suite of regression analyses testing for ontogenetic changes in diet, predator–prey size ratios and mouth gape. Silver carp *H. molitrix*, bighead carp *H. nobilis* and bighead carp × silver carp hybrids (collectively referred to as bigheaded carps), are known to occur in our study area (Anderson et al., 2023; Lamer et al., 2015; Locher, 2018), therefore an additional secondary objective was to determine if skipjack herring consumed bigheaded carps. The findings of this study improve our understanding of skipjack herring ecology and can inform resource management in a number of ways, such as by providing quantitative data that can be incorporated into food web models (Bizzarro et al., 2017).

## 2 METHODS

### 2.1 Ethics statement

Research was conducted according to animal care and use protocol 1304‐30541A approved by the University of Minnesota Animal Care and Use Committee.

### 2.2 Fish collection and preservation

**FIGURE 1:**
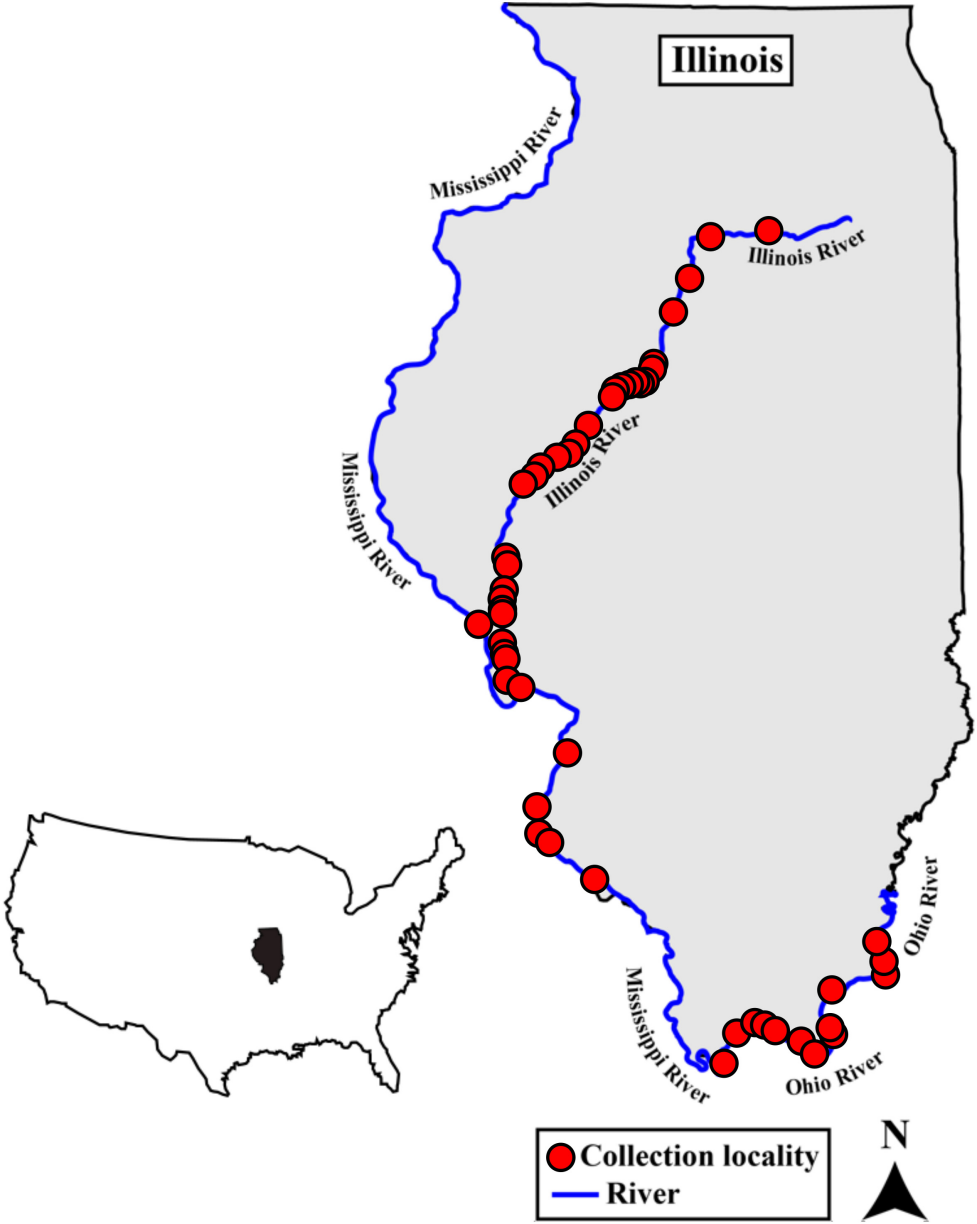
Map of the United States of America with the state of Illinois shaded in black (left) and enlarged map of Illinois showing locations (*n* = 52) where we collected skipjack herring *Alosa chrysochloris* during 2015, 2016 and 2017 in the Illinois, Ohio and Mississippi rivers in Illinois (right). Additional collection locality information is in Table S1.

We opportunistically collected skipjack herring from June through November in 2015, 2016 and 2017 in the Mississippi, Illinois and Ohio rivers (Figure 1) using pulsed‐direct‐current (60 Hz) electrofishing, standardized to a 3000‐watt power goal (Burkhardt & Gutreuter, 1995) as part of the Long‐Term Survey and Assessment of Large‐River Fishes in Illinois (Fritts et al., 2017), Ohio River Fish Population Monitoring and Sport Fisheries Investigations in Southern Illinois (Whitledge et al., 2015) and the Long‐Term Resource Monitoring element of the Upper Mississippi River Restoration Program (Ickes et al., 2014; Ratcliff et al., 2014). Specimens were retained in the field for this study when feasible and the numbers of specimens included from different years and locations are not necessarily entirely indicative of differences in skipjack herring abundance. Skipjack herring were immediately euthanized using 50 mg/L buffered MS‐222 following capture, then placed on ice for transport to the laboratory. At the laboratory, whole specimens were fixed in a 10% formalin solution for 7 days, then transferred to a 70% ethanol solution. Specimens were deposited in the University of Minnesota James Ford Bell Museum of Natural History, Minnesota, USA. Specimen catalogue numbers and collection locality information are given in Table .

### 2.3 Diet quantification

We measured SL of each skipjack herring using digital callipers, then dissected gut contents onto a microscope slide with a 1 × 1 mm grid. We quantified prey in the digestive tract up to the posterior end of stomach to avoid examining highly digested prey, which can bias diet descriptions (Gannon, 1976). We excluded individuals from our study if the anterior region of their digestive tract was empty or contained primarily highly degraded prey. We identified prey to the lowest practical taxonomic level and photographed them using a microscope‐mounted Spot Insight digital camera (Model 14.2 Colour Mosaic). We estimated the volumes of prey items following Alcaraz et al. (2003) and Egan, Gibbs and Simons (2018). First, we measured the maximum width, length and area of each prey item from photographs using ImageJ software (http://www.imagej.nih.gov/ij). We excluded appendages and the urosome from measurements of arthropods and fins from measurements of fishes. Using prey measurements and the cylinder equation for filamentous algae and ellipsoid equation for other prey types, we estimated prey volumes. In instances when we were only able to accurately measure the maximum width of prey, we used linear regression equations to make width‐based estimates of prey volume. We log‐transformed prey width versus volume data when relationships were non‐linear. In cases when skipjack herring guts contained large numbers of prey, we estimated the individual volumes of a random subset of prey. Then, we measured the entire volume of all prey dissected from the gut and used our estimates of the total volume of prey consumed and individual prey volumes to infer the relative contributions of each prey type to total prey volume (Table S2). In addition to calculating prey type consumption for individual skipjack herring, we also expressed prey type consumption for 10 mm SL bins (e.g. 20–30 mm SL) as percentage volume (volume of prey type/total prey volume), percentage number (number of individuals of prey type/total number of prey) and percentage frequency of occurrence (number of fish containing prey type/total number of fish).

### 2.4 Measurement of mouth gape

We measured the mouth gape of a representative subset (*n* = 60) of skipjack herring following Pusey and Bradshaw (1996) by opening the mouth to the point of maximum anterior maxillary and premaxillary rotation without unnatural distortion of the specimens, then measuring the dorsoventral distance between the anterior most points of the jaws using digital callipers (Table S3). We measured the mouth gape of each specimen three times and used the mean of these measurements in analyses.

### 2.5 Inter‐annual variation in bigheaded carps within survey catches

To determine if there were associations between inter‐annual variation in the abundance of bigheaded carps and the consumption of bigheaded carps by skipjack herring, we quantified the proportion of bigheaded carps (surveys did not distinguish between silver carp, bighead carp and bighead carp × silver carp hybrids) in long‐term survey forage fish catches (see section 2.2). We defined forage fish as any individual less than 80 mm total length (TL) following Ickes et al. (2022). Using data from the Illinois, Mississippi and Ohio rivers collected during our study period (2015–2017), we calculated the percentage contribution of bigheaded carps to the total annual catch of forage fish from each river. Survey and gut content analysis data allowed for simple comparisons of the abundance of bigheaded carps and the consumption of bigheaded carps by skipjack herring, but not statistical analyses.

### 2.6 Statistical analyses

We conducted statistical analyses in program R v3.4.0 (R Core Team, 2017). We condensed the 22 different categories of prey we identified into 11 prey types based on morphological and functional similarity, rather than taxonomy (Table 1). We tested for associations between prey type consumption and SL for prey types comprising at least 5% of the diet by volume of >1 skipjack herring using logistic regressions. We used a binary diet data coding in which skipjack herring were the unit of analysis, rather than the prey items themselves (i.e. a skipjack herring did or did not consume the focal prey type). For logistic regressions we used Bonferroni‐corrected *P* values of 0.0125 as the threshold for statistical significance (Rice, 1989).

**TABLE 1:** Prey categories comprising each more general prey type (not taxonomic) used in Skipjack Herring *Alosa chrysochloris* diet analyses.

Prey type	Constituent prey categories
Algae	Filamentous algae
Annelida	Nematoda, unidentified Annelida
Detritus	Detritus
Eggs	Invertebrate eggs, vertebrate eggs
Fish	Fish
Terrestrial Insecta/Arachnida	Terrestrial adult and sub‐adult Insecta and Arachnida
Aquatic Insecta	Aquatic adult and sub‐adult Insecta
Mollusca	Non‐planktonic Bivalva, non‐planktonic Gastropoda
Phytoplankton	Dinoflagellata
Plant	Aquatic and terrestrial plant material
Zooplankton	Copepoda, Cladocera, Ostracoda, Bivlava veliger, Gastropoda veliger

We examined differences in skipjack herring diets between two periods (June–August versus September–November) and two sampling areas (Illinois versus Ohio Rivers) using non‐metric multidimensional scaling ordination (NMDS) based on Bray–Curtis dissimilarity in prey type consumption using the metaMDS command in the vegan package (Oksansen et al., 2016). We conducted analyses of similarity (ANOSIM) to determine if differences in diet between sampling periods and rivers were statistically significant and analyses of similarity percentages (SIMPER) to characterize the relative contributions of individual prey types to differences in diet using the anosim and simper vegan package commands. Regression analyses revealed changes in skipjack herring diets correlated with SL. Therefore, when exploring spatial and temporal variation in diet, we made diet comparisons within 20–80, 80–120 and 120–160 mm SL bins to minimize biases due to skipjack herring SL differences between samples. Sample sizes were insufficient to use narrower SL bins, a SL bin containing skipjack herring >160 mm SL, samples from the Mississippi River or conduct higher‐resolution analyses of spatial and temporal diet variation. Tests for spatial and temporal variation in diet (NMDS/ANOSIM/SIMPER) were the only analyses that used size bins and did not include data from all sampled skipjack herring.

We identified associations between prey size consumption and SL using quantile regression with the quantreg package (Koenker et al., 2018) and an *α* value of 0.05 as the threshold for statistical significance. We used quantile regression because diagnostic regression plots (residuals versus fitted values, normal Q‐Q and scale‐location plots) and Breusch Pagan tests conducted with the lmtest package (Hothorn et al., 2017) revealed that the prey size versus predator SL data did not conform to the linear regression assumption of homoscedasticity. We tested for correlations between SL and minimum (0.01 quantile), median (0.5 quantile) and maximum (0.99 quantile) prey widths and volumes. We tested for correlations between SL and minimum (0.01 quantile), median (0.5 quantile) and maximum (0.99 quantile) prey width to SL ratios (prey width/SL) and prey volume to SL ratios (prey volume/SL). For quantile regression analyses, we iteratively fit increasingly complex models for each quantile, starting with a linear model and progressing to second‐, then third‐degree polynomial models if the more complex model was determined to be significantly better than the simpler model. We compared the fits of quantile regression models by conducting Wald tests with the anova.rq quantreg function, which tests the hypothesis that the smaller model is sufficient relative to the larger model (significance is indicated via a *p* value; Koenker & Bassett, 1982).

We used regression analyses to model the relationship between SL and mouth gape, and test the hypothesis that skipjack herring mouth gape changes allometrically relative to SL by testing for a correlation between SL and the mouth gape to SL ratio (mouth gape/SL), a standard approach in fish allometry studies (Bachiller & Irigoien, 2013; Scharf et al., 2000). A statistically significant correlation between SL and the mouth gape to SL ratio would indicate that the relationship between mouth gape and body length varies during skipjack herring ontogeny, supporting the hypothesis of mouth gape allometry (Bachiller & Irigoien, 2013; Scharf et al., 2000). Diagnostic regression plots indicated that the mouth gape versus SL data may not have been linear. Consequently, we fit linear and polynomial regression models to the SL versus mouth gape and SL versus mouth gape to SL ratio data and compared model fit with Akaike's Information Criterion (AIC).

To test the hypothesis that mouth gape influenced the maximum sizes of prey consumed by skipjack herring, we tested for (1) a correlation between maximum prey size consumption and mouth gape and (2) ontogenetic changes in the ratio of maximum prey size relative to mouth gape (i.e. are ratios between maximum prey size and mouth gape consistent across different sizes of mouth gape?). A positive correlation between maximum prey size consumption and mouth gape and a constant maximum prey size to mouth gape ratio through ontogeny (i.e. no correlation between maximum prey size to mouth gape ratio and mouth gape) would support the hypothesis that mouth gape is a key determinant of maximum prey size consumption. A weak relationship between maximum prey size consumption and mouth gape and ontogenetic changes in the ratio of maximum prey size relative to mouth gape are predicted if factors other than mouth gape, such as predator avoidance or swimming performance, limit prey size consumption in the Skipjack Herring (Scharf et al., 2000, 2009).

We used quantile regression to test for correlations between mouth gape and minimum (0.01 quantile), median (0.5 quantile) and maximum (0.99 quantile) prey widths and volumes. To identify ontogenetic changes in the relationship between maximum prey size and mouth gape, we used quantile regression to test for a correlation between the maximum (0.99 quantile) prey width to gape ratio (prey width/mouth gape) and mouth gape. For quantile regression involving mouth gape, we used the best fitting mouth gape versus SL regression equation (see above) to estimate mouth gape for skipjack herring for which we did not measure mouth gape. Then, we used both estimated and empirical gape data in quantile regression analyses.

## 3 RESULTS

We dissected 442 skipjack herring of which 6, 181 and 255 individuals were from the Mississippi, Illinois and Ohio rivers, respectively, and 382, 39 and six were collected in 2015, 2016 and 2017, respectively (Table S1). One hundred and four skipjack herring had digestive tracts that were either empty or only contained prey that were too digested to measure and 338 contained identifiable prey. Skipjack herring containing identifiable prey ranged from 28 to 231 mm SL (all fish were juvenile‐sized; Becker, 1983; Etnier & Starnes, 1993; Wolfe, 1969), from which we measured 3671 individual prey items (Tables 2 and S4). All statistical analyses used the full diet dataset (skipjack herring *n* = 338, prey items *n* = 3671), except for tests of spatial and temporal variation in diet (NMDS/ANOSIM/SIMPER) as described above, which excluded skipjack herring >160 mm SL and skipjack herring from the Mississippi River (skipjack herring with identifiable prey excluded *n* = 17, prey items excluded *n* = 69).

**TABLE 2:** Number of skipjack herring *Alosa chrysochloris* dissected (*n* pred) in each standard length (SL) bin (SL bin (mm)), number of skipjack herring containing identifiable and measurable prey (*n* pred *w*/ prey) in each SL bin, and number of prey consumed (*n* prey) by Skipjack Herring in each SL bin.

Bin	*n* pred total	*n* pred *w*/ prey	*n* prey	Fish			InsT			InsA			Plan			Zoop		
				%V	%F	%N	%V	%F	%N	%V	%F	%N	%V	%F	%N	%V	%F	%N
20–30	5	5	178	0.000	0.000	0.000	0.007	0.200	0.006	0.924	0.400	0.017	0.000	0.000	0.000	0.068	0.600	0.882
30–40	23	19	172	0.488	0.053	0.006	0.142	0.263	0.140	0.284	0.263	0.203	0.000	0.105	0.017	0.085	0.737	0.587
40–50	14	14	185	0.830	0.143	0.011	0.141	0.357	0.276	0.023	0.286	0.141	0.000	0.071	0.011	0.005	0.643	0.557
50–60	35	32	346	0.909	0.250	0.052	0.040	0.375	0.488	0.022	0.344	0.107	0.029	0.031	0.003	0.001	0.594	0.327
60–70	40	29	194	0.968	0.345	0.113	0.020	0.310	0.335	0.012	0.414	0.149	0.750	0.069	0.026	0.000	0.414	0.366
70–80	64	53	1141	0.878	0.094	0.010	0.033	0.321	0.069	0.056	0.396	0.138	0.018	0.057	0.003	0.006	0.623	0.772
80–90	44	39	693	0.785	0.179	0.029	0.194	0.410	0.078	0.017	0.359	0.092	0.000	0.000	0.000	0.004	0.513	0.789
90–100	38	33	280	0.967	0.273	0.086	0.016	0.455	0.211	0.016	0.273	0.086	0.000	0.030	0.007	0.000	0.121	0.243
100–110	32	28	131	0.989	0.607	0.374	0.008	0.250	0.237	0.003	0.179	0.366	0.000	0.000	0.000	0.000	0.071	0.015
110–120	36	24	123	0.994	0.708	0.447	0.004	0.292	0.276	0.002	0.250	0.236	0.001	0.083	0.016	0.000	0.000	0.000
120–130	30	17	71	0.990	0.529	0.282	0.006	0.588	0.437	0.002	0.085	0.225	0.000	0.000	0.000	0.000	0.000	0.000
130–140	19	8	65	0.984	0.500	0.138	0.005	0.250	0.077	0.011	0.031	0.754	0.000	0.125	0.015	0.000	0.125	0.015
140–150	23	14	32	0.977	0.429	0.344	0.001	0.214	0.286	0.013	0.571	0.469	0.008	0.071	0.031	0.000	0.000	0.000
150–160	16	11	17	0.999	0.727	0.706	0.000	0.182	0.118	0.000	0.182	0.176	0.000	0.000	0.000	0.000	0.000	0.000
160–170	4	3	6	0.992	1.000	0.500	0.008	0.333	0.500	0.000	0.000	0.000	0.000	0.000	0.000	0.000	0.000	0.000
170–180	6	4	19	0.998	0.750	0.684	0.002	0.250	0.263	0.000	0.000	0.000	0.000	0.000	0.000	0.000	0.000	0.000
180–190	2	1	2	1.000	1.000	1.000	0.000	0.000	0.000	0.000	0.000	0.000	0.000	0.000	0.000	0.000	0.000	0.000
190–200	3	1	11	0.000	0.000	0.000	1.000	1.000	1.000	0.000	0.000	0.000	0.000	0.000	0.000	0.000	0.000	0.000
200–210	2	1	2	0.999	1.000	0.500	0.001	1.000	0.500	0.000	0.000	0.000	0.000	0.000	0.000	0.000	0.000	0.000
210–220	1	1	1	1.000	1.000	1.000	0.000	0.000	0.000	0.000	0.000	0.000	0.000	0.000	0.000	0.000	0.000	0.000
220–230	3	0	0	‐	‐	‐	‐	‐	‐	‐	‐	‐	‐	‐	‐	‐	‐	‐
230–240	1	1	2	0.000	0.000	0.000	0.000	0.000	0.000	1.000	1.000	1.000	0.000	0.000	0.000	0.000	0.000	0.000
270–280	1	0	0	‐	‐	‐	‐	‐	‐	‐	‐	‐	‐	‐	‐	‐	‐	‐
**Total**	**442**	**338**	**3671**															

*Note*: Prey type consumption by skipjack herring for SL size bins expressed as percentage volume (%V; volume of prey type/total prey volume), percentage frequency of occurrence (%F; number of fish containing prey type/total number of fish) and percentage number (%N; number of individuals of prey type/total number of prey). We only included prey type categories that comprised at least 0.001 percentage of diet by volume for at least one size bin: Fish, teleost fishes; InsT, terrestrial Insecta; InsA, aquatic Insecta; Plan, plants; Zoop, zooplankton. Prey types are defined in Table 1.

### 3.1 Prey type consumption

The dominant prey types in skipjack herring diets were fish, aquatic and terrestrial insects, and zooplankton (Tables 2 and S4). The most prevalent fish species consumed by volume and number were bigheaded carps (we were unable to distinguish between silver carp, bighead carp, and bighead carp × silver carp hybrids). Of the 188 fish prey that we could identify, 148 individuals (79%) were bigheaded carps, which comprised 71% of the total volume of identifiable fish prey. This result was driven by 2015 data (the year during which we collected most [89%] skipjack herring samples), in which bigheaded carps comprised 78% of the volume of identifiable fish prey. During 2015, bigheaded carps were primarily consumed by skipjack herring in July (95% of the bigheaded carps consumed were eaten during this month; bigheaded carp consumption by month expressed as frequency of occurrence: April = 0%, June = 0%, July = 44%, August = 1%, September = 0%, October = 1%, November = 9%; Table S4). Bigheaded carps were not identified in the diets of skipjack herring collected in 2016 or 2017. Other common taxa consumed by skipjack herring were minnows (Leucisidae), especially *Notropis* spp. (11% of individual fish prey and 7% of fish prey volume), gizzard shad/threadfin shad *Dorosoma* spp. (3% of individual fish prey and 11% of fish prey volume), sunfish *Lepomis* spp. (2% of individual fish prey and 5% of fish prey volume) and western mosquitofish *Gambusia affinis* (2% of individual fish prey and 1% of fish prey volume). Copepoda were the most common zooplankton consumed (85% of zooplankton individuals, 84% of zooplankton volume), followed by Cladocera (10% of zooplankton individuals, 14% of zooplankton volume), then Ostracoda (6% of zooplankton individuals, 2% of zooplankton volume). Of identifiable terrestrial insects, the most common taxa were Hymenoptera (69% of terrestrial insect individuals, 61% of terrestrial insect volume), Apocrita (ants, bees and wasps) in particular, and Coleoptera (6% of terrestrial insect individuals, 9% of terrestrial insect volume). Of identifiable aquatic insects, the most common taxa were Trichoptera (61% of aquatic insect individuals, 76% of aquatic insect volume) and Diptera larvae and pupae (37% of aquatic insect individuals, 22% of aquatic insect volume).

### 3.2 Inter‐annual variation in the representation of bigheaded carps in survey catches

In the Illinois River, bigheaded carps comprised 0.6%, 0.4% and 0.3% of forage fishes collected by surveys in 2015, 2016 and 2017, respectively. In the Mississippi River, bigheaded carps comprised 0.3%, 0.2% and 0.0% of forage fishes collected by surveys in 2015, 2016 and 2017, respectively. In the Ohio River, bigheaded carps comprised 30.2%, 0.5% and 0.3% of forage fishes collected by surveys in 2015, 2016 and 2017, respectively.

### 3.3 Ontogenetic shifts in prey type consumption

**FIGURE 2:**
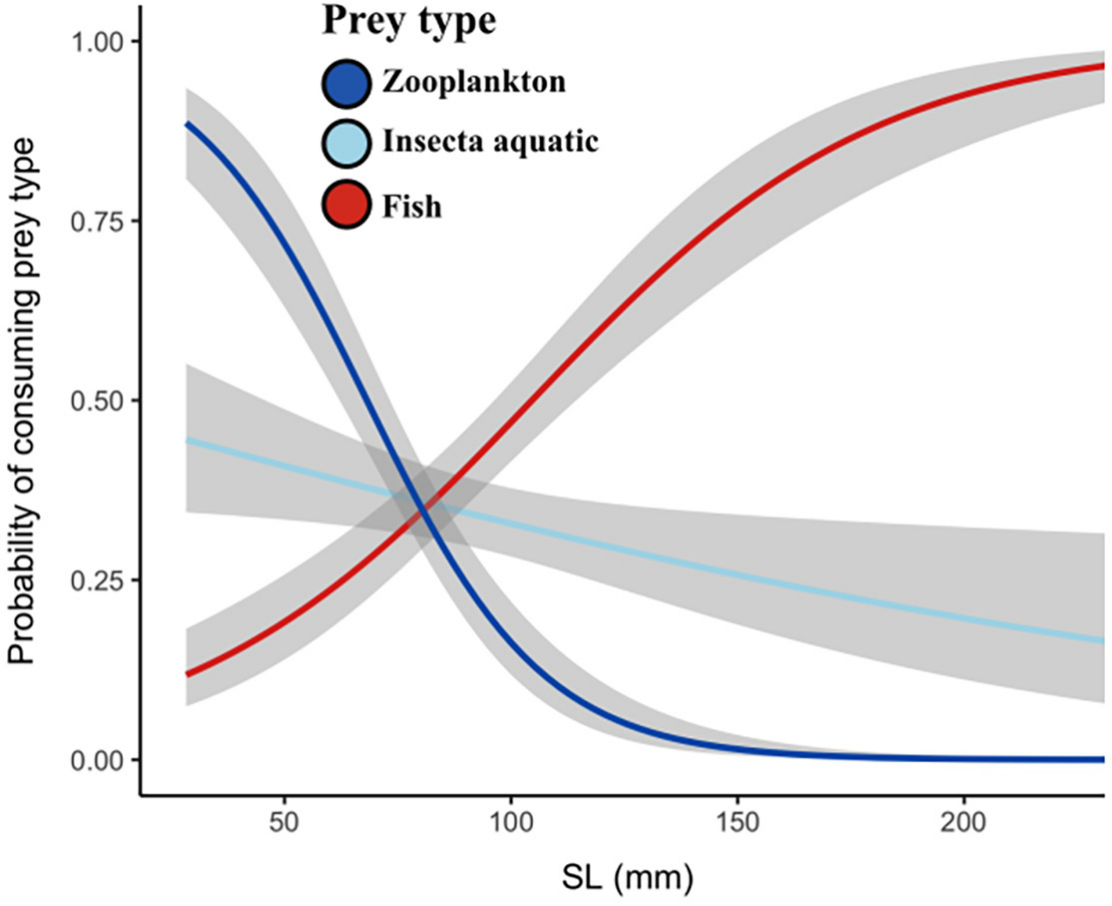
Logistic regression lines illustrating the modelled probability of a skipjack herring *Alosa chrysochloris* (*n* = 338) individual consuming a prey type (zooplankton, Insecta aquatic or fish) based on the relationship between skipjack herring standard length (SL) on the *x* axis and the frequency at which individual skipjack herring consumed focal prey types (i.e. did (1) or did not (0) consume focal prey type) on the *y* axis. Shading around regression lines shows the 95% confidence interval for each logistic regression model.

Aquatic insects dominated the diets of the smallest skipjack herring (20–30 mm SL bin), with zooplankton and terrestrial insects also contributing (Table 2). Fishes comprised almost half (~49%) of the prey volume in skipjack herring in the 30–40 mm SL bin. At SLs >40 mm, fishes constituted the majority of skipjack herring diets. Terrestrial and aquatic insects, and zooplankton were consumed in small quantities by skipjack herring 30–120 mm SL but rarely consumed by larger skipjack herring (>120 mm SL; Table 2). There were significant negative correlations between SL and zooplankton consumption, and SL and aquatic insect consumption, showing that the maximum probabilities of consuming zooplankton (~88%) and aquatic insects (~45%) occurred at 28 mm SL and declined as SL increased to probabilities of ~0.05% and ~17% at 231 mm SL for zooplankton and aquatic insects, respectively (Table 3 and Figure 2). There was a significant positive correlation between SL and fish consumption, with the minimum probability of consuming fish (~12%) occurring at 28 mm SL, which increased to a probability of ~98% at 231 mm SL (Table 3 and Figure 2). There was no significant correlation between SL and terrestrial insect consumption (Table 3).

**TABLE 3:** Logistic regression equations predicting the probability (PR) of an individual skipjack herring *Alosa chrysochloris* consuming the focal prey types and associated *p* values.

Prey type	Equation	*p*
Fish	PR = e^0.03769^SL – 5.85641/1 + e^0.03769^SL – 5.85641	<0.0001
Aquatic insects	PR = e^−0.00691^SL – 0.023695/1 + e^−0.00691^SL – 0.023695	0.02
Terrestrial insects	PR = e^−0.005542^SL – 0.351166/1 + e^−0.005542^SL – 0.351166	0.07
Zooplankton	PR = e^−0.051423^SL + 3.504931/1 + e^−0.051423^SL + 3.504931	<0.0001

*Note*: Logistic regression models describe the relationship between skipjack herring (*n* = 338), standard length (SL) in mm (predictor variable) versus a binary diet data coding (response variable): Individual skipjack herring did (1) or did not (0) consume focal prey type. PR = probability of consuming prey type. The prey categories comprising each prey type are listed in Table 1.

### 3.4 Spatial and temporal variation in diet

**FIGURE 3:**
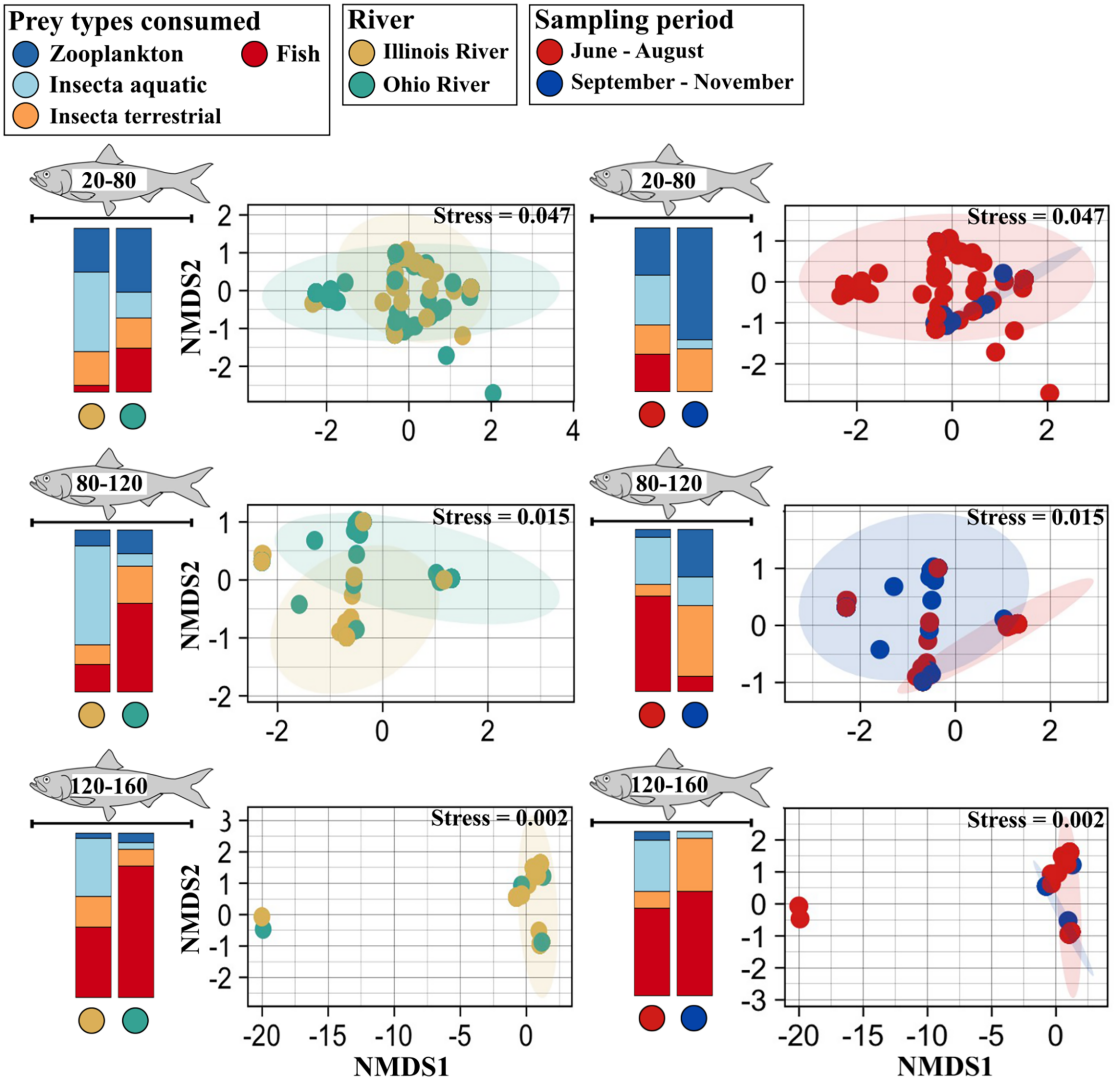
Bar plots and two‐dimensional NMDS ordination plots based on Bray–Curtis dissimilarity in prey type consumption comparing differences in the proportions of zooplankton, aquatic insects, terrestrial insects and fish in skipjack herring *Alosa chrysochloris* (*n* = 321) diets from different rivers (Ohio versus Illinois) and sampling periods (June–August versus September–November). We made diet comparisons among rivers and sampling periods within three SL bins (20–80 mm SL, 80–120 mm SL and 120–160 mm SL) to minimize biases due to differences in the proportions of skipjack herring SLs sampled in rivers and sampling periods.

There were no prey types unique to any sampling period (June–August vs. September–November) or river (Illinois vs. Ohio rivers). NMDS ordination plots showed substantial overlap in diets between sampling periods and locations for all SL bins (20–80, 80–120, 120–160 mm SL) but suggested minor dietary differences between rivers and seasons for 20–80 and 80–120 mm SL skipjack herring (Figure 3). There were statistically significant differences in diet between rivers for 20–80 (*p* = 0.031) and 80–120 mm SL fish (*p* < 0.001) and between sampling periods for 80–120 mm SL fish (*p* < 0.001). There were no significant differences in diet between rivers for 120–160 mm SL fish (*p* = 0.130) or between sampling periods for 20–80 (*p* = 0.944) or 120–160 mm SL fish (*p* = 0.373). Differences in diet between rivers for 20–80 mm SL fishes were driven by consumption of more aquatic insects and zooplankton, and fewer terrestrial insects in the Ohio River than the Illinois River (Figure 3). Differences in the diet between rivers for 80–120 mm SL fishes were due to consumption of more fishes and terrestrial insects and fewer aquatic insects in the Ohio River than the Illinois River (Figure 3). Differences in diet between sampling periods for 80–120 mm SL fishes were due to consumption of more fishes and aquatic insects and fewer terrestrial insects in June–August than September–November (Figure 3).

### 3.5 Ontogenetic shifts in prey size consumption and mouth gape

**FIGURE 4:**
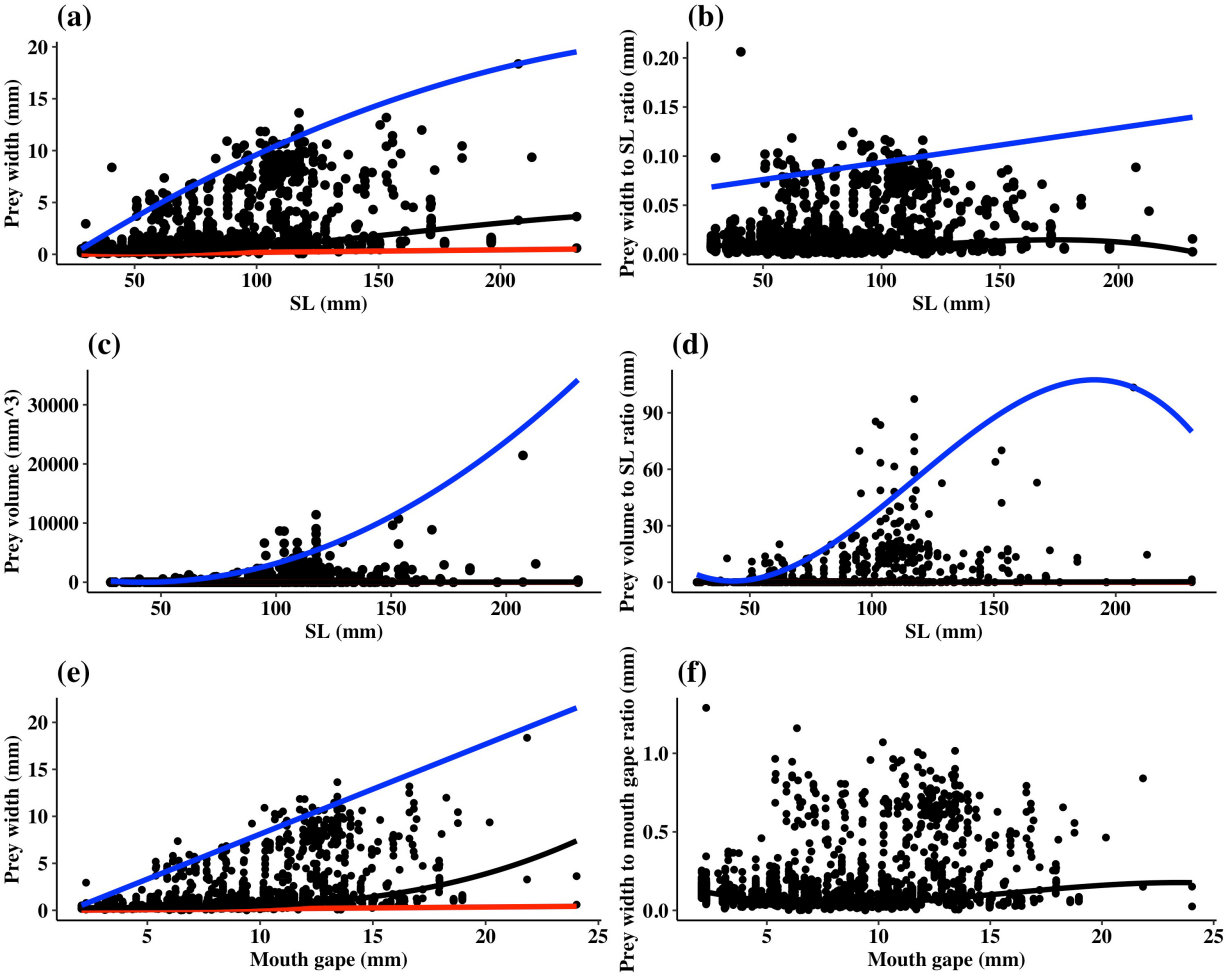
Scatter plots with quantile regression lines (blue = 0.99 quantile, black = 0.5 quantile and red = 0.01 quantile) for statistically significant relationships. (a) Skipjack herring *Alosa chrysochloris* (*n* = 338) standard length (SL; mm) versus prey (*n* = 3671) width (mm). (b) Skipjack herring (*n* = 338) SL (mm) versus prey (*n* = 3671) width to SL ratio (prey width (mm)/SL (mm)). (c) Skipjack herring (*n* = 338) SL (mm) versus prey (*n* = 3671) volume (mm^3^). (d) Skipjack herring (*n* = 338) SL (mm) versus prey (*n* = 3671) volume to SL ratio (mm). (e) Skipjack herring (*n* = 338) mouth gape (mm) versus prey (*n* = 3671) width (mm). (f) Skipjack herring (*n* = 338) mouth gape (mm) versus prey (*n* = 3671) width to mouth gape ratio (mm). In (c) and (d) median prey and minimum prey size quantile regression lines are nearly identical and as a result, the median prey size regression lines are partially obscured.

There were statistically significant positive correlations between maximum prey width and SL, maximum prey volume and SL, maximum prey width to SL ratio and SL, and maximum prey volume to SL ratio and SL (Table 4 and Figure 4). There were also significant correlations between median and minimum prey size and SL, and between median and minimum prey size to SL ratios and SL. However, in all cases, ontogenetic changes in median and minimum prey sizes, and median and minimum prey size to SL ratios were minor (Table 4 and Figure 4).

**TABLE 4:** Coefficient values (Estimate) and *p* values from best‐fitting quantile regression models of skipjack herring *Alosa chrysochloris* (*n* = 338) standard length (SL) versus prey size (*n* = 3671; Response).

Response	Quantile	Coefficient	Estimate	*p*	Wald *p* value
PW	0.99	Intercept	−3.75167 ± 1.31841	0.00446	0.01569
		SL	0.15865 ± 0.03259	<0.00001	
		SL^2^	−0.0025 ± 0.00019	0.18953	
PW	0.50	Intercept	0.86023 ± 0.16451	<0.00001	0.01789
		SL	−0.02607 ± 0.00776	0.00078	
		SL^2^	0.00031 ± 0.00011	0.00447	
		SL^3^	0.00000 ± 0.00000	0.17812	
PW	0.01	Intercept	−0.04965 ± 0.04568	0.27714	
		SL	0.00239 ± 0.00061	0.00009	
RPW	0.99	Intercept	0.05861 ± 0.01402	0.00003	
		SL	0.00035 ± 0.00015	0.02261	
RPW	0.50	Intercept	0.02210 ± 0.00153	<0.00001	<0.00001
		SL	−0.00059 ± 0.00006	<0.00001	
		SL^2^	0.00001 ± 0.00000	<0.00001	
		SL^3^	0.00000 ± 0.00000	<0.00001	
RPW	0.01	Intercept	0.00110 ± 0.00052	0.03597	
		SL	0.00001 ± 0.00001	0.24266	
PV	0.99	Intercept	1723.17941 ± 859.61259	0.04508	<0.00006
		SL	−81.26495 ± 28.66659	0.00461	
		SL^2^	0.95946 ± 0.23737	0.00005	
PV	0.50	Intercept	3.62571 ± 0.74389	<0.00001	<0.00001
		SL	−0.13129 ± 0.02659	<0.00001	
		SL^2^	0.00112 ± 0.00022	<0.00001	
PV	0.01	Intercept	−0.04947 ± 0.05143	0.33614	<0.00001
		SL	0.00296 ± 0.00254	0.24506	
		SL^2^	−0.00006 ± 0.00004	0.17341	
		SL^3^	0.00000 ± 0.00000	0.11358	
RPV	0.99	Intercept	5.90290 ± 6.17063	0.33883	0.01796
		SL	0.38249 ± 0.23295	0.10069	
		SL^2^	0.00633 ± 0.00203	0.00187	
RPV	0.5	Intercept	−0.00264 ± 0.00027	<0.00001	
		SL	0.00007 ± 0.00001	<0.00001	
RPV	0.01	Intercept	−0.00012 ± 0.00002	<0.00001	
		SL	0.00000 ± 0.00000	<0.00001	

*Note*: We report coefficients, *p* values from regression analyses (*p* value), and *p* values from Wald regression model fit tests (Wald *p* value) for response variables that exhibit a statistically significant correlations with SL and for the smallest non‐significant regression model for each SL versus prey size relationship. We fit models to maximum (0.99 quantile), median (0.50 quantile), and minimum (0.01 quantile) quantiles. Prey size response variables included prey width (PW), prey width to SL ratio (prey width/SL; RPW), prey volume (PV) and prey volume to SL ratios (prey volume/SL; RPV) in μm.

There were statistically significant positive correlations between maximum, median and minimum prey width and mouth gape (Table 5). There were no significant correlations between maximum (0.99 quantile) or minimum (0.01 quantile) prey width to mouth gape ratio and mouth gape (Table 5). However, the slope of the fitted regression line for maximum prey width to mouth gape ratio and mouth gape was slightly positive (slope = 0.02) and the relationship was nearly significant (*p* = 0.052). In other words, although this relationship was not statistically significant and the slope of the fitted regression line was very shallow, there is a small amount of evidence that fish with larger gapes ate slightly larger prey relative to their gape. There was a statistically significant correlation between median (0.50 quantile) prey width to mouth gape ratio and mouth gape (Table 5).

**TABLE 5:** Coefficient values (Estimate) and *p* values from best‐fitting quantile regression models of skipjack herring *Alosa chrysochloris* (*n* = 338) mouth gape versus prey size (*n* = 3671; Response).

Response	Quantile	Coefficient	Estimate	*p*	Wald *p* value
Prey width to mouth gape ratio	0.99	Intercept	0.56883 ± 0.12047	<0.00001	
		Mouth gape	0.02163 ± 0.01115	0.05234	
Prey width to mouth gape ratio	0.50	Intercept	0.7374 ± 0.00308	<0.00001	
		Mouth gape	−0.00256 ± 0.00034	<0.00001	
Prey width to mouth gape ratio	0.50	Intercept	0.16457 ± 0.00649	<0.00001	<0.00001
		Mouth gape	−0.2752 ± 0.00193	<0.00001	
		Mouth gape^2	0.00157 ± 0.00014	<0.00001	
Prey width to mouth gape ratio	0.50	Intercept	0.19708 ± 0.01258	<0.00001	0.01971
		Mouth gape	−0.04281 ± 0.00577	<0.00001	
		Mouth gape^2	0.00354 ± 0.00077	<0.00001	
		Mouth gape^3	−0.00007 ± 0.00003	0.01573	
Prey width to mouth gape ratio	0.01	Intercept	0.00946 ± 0.00494	0.11126	
		Mouth gape	0.00057 ± 0.00060	0.34069	
Prey width	0.99	Intercept	−1.46430 ± 0.33370	0.00001	
		Mouth gape	0.95742 ± 0.04160	<0.00001	
Prey width	0.99	Intercept	−1.40388 ± 1.15089	0.22261	0.7061
		Mouth gape	0.92361 ± 0.27390	0.00075	
		Mouth gape^2	0.00231 ± 0.01513	0.87889	
Prey width	0.50	Intercept	0.10489 ± 0.01427	<0.00001	
		Mouth gape	0.03798 ± 0.00199	<0.00001	
Prey width	0.50	Intercept	0.55144 ± 0.04356	<0.00001	<0.00001
		Mouth gape	−0.11686 ± 0.01562	<0.00001	
		Mouth gape^2	0.01129 ± 0.00122	<0.00001	
Prey width	0.50	Intercept	0.19836 ± 0.10942	0.06994	0.02691
		Mouth gape	0.05670 ± 0.06060	0.34952	
		Mouth gape^2	−0.01208 ± 0.00916	0.18723	
		Mouth gape^3	0.00092 ± 0.00042	0.02646	
Prey width	0.01	Intercept	−0.02757 ± 0.05328	0.60491	
		Mouth gape	0.01880 ± 0.00637	0.00316	
Prey width	0.01	Intercept	0.04159 ± 0.05338	0.43593	0.2947
		Mouth gape	0.00074 ± 0.01407	0.9583	
		Mouth gape^2	0 l00094 ± 0.00079	0.23323	

*Note*: We report coefficients, *p* values from regression analyses (*p* value), and *p* values from Wald regression model fit tests (Wald *p* value) for response variables that exhibit a statistically significant correlations with mouth gape and for the smallest non‐significant regression model for each mouth gape versus prey size relationship. We fit models to maximum (0.99 quantile), median (0.50 quantile) and minimum (0.01 quantile) quantiles. Prey size response variables were prey width and prey width to mouth gape ratio (prey width/mouth gape) in μm.

**FIGURE 5:**
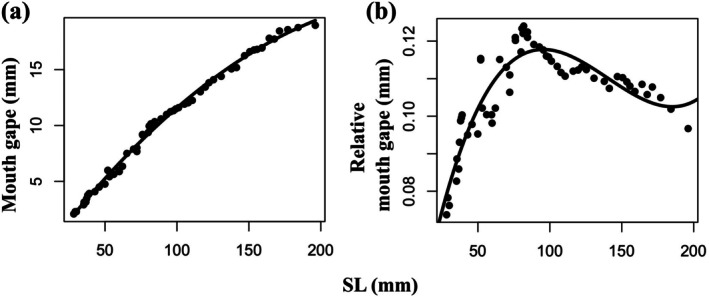
Scatter plots of (a) skipjack herring *Alosa chrysochloris* (*n* = 338) standard length (SL) (*x* axis) versus mouth gape (*y* axis) with the best fit (second degree polynomial) regression line and (b) skipjack herring (*n* = 338) SL (*x* axis) versus relative mouth gape (mouth gape/SL; *y* axis) with the best fit (third degree polynomial) regression line.

A second‐degree polynomial model best described the association between mouth gape and SL (highest *R* ^2^ value) and was selected as the best model by AIC, revealing mouth gape allometry in the skipjack herring (Table 6). A third‐degree polynomial model best characterized the relationship between mouth gape to SL ratio and SL (highest *R* ^2^ value) and was selected as the best model by AIC (Table 6). Mouth gape relative to SL increased between 28 and ~60 mm SL, was largest at intermediate SLs (~ 60–110 mm), then decreased again at SLs larger than ~110 mm (Figure 5).

**TABLE 6:** Linear, second degree (2°) and third degree (3°) polynomial regression (Model) equations (Equation), *p* values, *R* ^2^ values and AIC values resulting from regressions of skipjack herring *Alosa chrysochloris* (*n* = 338) standard length (SL) versus mm mouth gape (G) and mouth gape to SL ratio (i.e. relative gape; RG) versus SL.

Model	Equation	*p*	*R* ^2^	AIC
G vs. SL (linear)	G = 0.108883SL − 0.0007684	<0.0001	0.98	134.9
G vs. SL (2° polynomial)	G = −2.625 + 0.1731SL − 0.0003099SL^2^	<0.0001	0.996	48.7
RG vs. SL (linear)	RG = 0.00009143SL + 0.09826	0.004	0.13	−366.7
RG vs. SL (2° polynomial)	RG = 0.06079 + 0.001008SL‐4.425e^−6^SL^2^	<0.0001	0.72	−431.7
RG vs. SL (3° polynomial)	RG = 0.02899 + 0.002246SL‐1.783e^−5^SL^2^ + 4.237e^−8^SL^3^	<0.0001	0.82	−457.0

*Note*: Standard length and mouth gape were measured in mm.

## 4 DISCUSSION

This study is the first quantitative description of juvenile skipjack herring diets. We documented an ontogenetic shift from diets containing large quantities of zooplankton and insects in skipjack herring <40 mm SL to diets dominated by fishes in skipjack herring >40 mm SL. These changes in prey type consumption corresponded to large increases in the maximum sizes of prey eaten along with modest increases in median and minimum prey sizes. We found that mouth gape was positively correlated with maximum prey size and identified a relatively constant ratio of maximum prey size to mouth gape, suggesting that mouth gape is a key determinant of maximum prey size consumption by juvenile skipjack herring. We discovered allometric changes in mouth gape and found that the largest mouth gape to SL ratios occurred between ~60 and 110 mm SL, which was during the ontogenetic transition to piscivory. These relationships provide preliminary evidence that mouth gape allometry may facilitate the transition to piscivory in the skipjack herring.

We documented ontogenetic changes in the diet of the skipjack herring involving increases in fish consumption and decreases in zooplankton and aquatic insect consumption that corresponded to increases in the sizes of prey ingested. Fishes made up the bulk of skipjack herring diets at all but the smallest SLs (<40 mm SL), at which they consumed greater volumes of aquatic and terrestrial insects than fishes. This study confirms the previously reported piscivorous feeding habits of the skipjack herring (Etnier & Starnes, 1993; McLean et al., 1985; Whitehead et al., 1988) and the importance of insects in the diets of small individuals (Whitehead et al., 1988). Our study is the first to document consumption of zooplankton by skipjack herring, likely because we examined smaller individuals than previous studies (Etnier & Starnes, 1993; McLean et al., 1985; Whitehead et al., 1988). Our results suggest that in large river food webs skipjack herring <40 mm SL are functionally similar to lotic minnows, such as the emerald shiner *Notropis atherinoides*, and the juveniles of large‐bodied piscivores, such as white bass *Morone chrysops* (Mittelbach & Persson, 1998; Roberts & Taylor, 2008). Multiple co‐occurring piscivores, including longnose gar *Lepisosteus osseus* (Scott & Crossman, 1979), largemouth bass *Micropterus salmoides* (Keast, 1985), walleye *Sander vitreus* (Mathias & Li, 1982) and sauger (Nelson, 1968), undergo similar ontogenetic diet changes, with zooplankton and insects comprising large proportions of the diets of small juveniles before these species transition to diets primarily consisting of fishes and other large, evasive prey.

The smallest skipjack herring in our dataset that consumed fishes was 38 mm SL/46 mm TL and fishes comprised the majority of diets at SLs >40 mm regardless of sampling location or year. The length at which skipjack herring transitioned to piscivory is similar to co‐occurring freshwater piscivores such as longnose gar (Scott & Crossman, 1979), largemouth bass (Keast, 1985) and walleye (Mathias & Li, 1982) and shorter than co‐distributed large river fishes such as bowfin *Amia calva* (Becker, 1983), yellow bullhead *Ameiurus natalis* (Keast, 1985) and sauger (Nelson, 1968), which begin consuming fishes at ~70–100, ~180–240 and ~70–100 mm TL, respectively. Bigheaded carps were the most common fish consumed by skipjack herring but were only in diets during 2015 (the year in which we collected ~89% of the skipjack herring sampled). Survey data indicated that bigheaded carps small enough to be consumed by skipjack herring were much more abundant during 2015 in the Ohio River (30.2% of forage fish catches) compared to other sampling locations and years, in which bigheaded carps never represented more than 0.6% of forage fish catches. Therefore, low abundance of small bigheaded carps (<80 mm TL) in the sampling area may explain why bigheaded carps were absent from skipjack herring diets in 2016 and 2017. However, this hypothesis should be treated with caution because our study included smaller skipjack herring sample sizes in 2016 and 2017 relative to 2015. Minnows, gizzard shads and threadfin shads were also frequently consumed, indicating that skipjack herring primarily feed on pelagic, rheophilic species. Fishes with diets similar to >40 mm SL skipjack herring that commonly occur in lotic habitats in the Mississippi River Basin include white bass, longnose gar and sauger (Bellgraph et al., 2008; Dettmers et al., 2001a, 2001b; Hartman, 1998; Solomon et al., 2019; Voigtlander & Wissing, 1974).

Bigheaded carps are highly invasive throughout large portions of the Mississippi River Basin (Anderson et al., 2023; Chick & Pegg, 2001; Chick, Gibson‐Reinemer, & Soeken‐Gittinger, 2020; Gibson‐Reinemer et al., 2017; Lamer et al., 2010, 2015; Lampo et al., 2023; Locher, 2018) and there is interest in using native predator enhancement to reduce their abundance (Anderson et al., 2023; Lampo et al., 2023). Consequently, our finding that bigheaded carps comprised 71% of the total volume of identifiable fish prey consumed by juvenile skipjack herring may be of interest to fishery managers. Several other native predatory fishes have also been documented to consume bigheaded carps in the Mississippi River Basin, including white bass, shortnose gar *Lepisosteus platostomus*, black crappie *Pomoxis nigromaculatus*, channel catfish *Ictalurus punctatus*, smallmouth bass *Micropterus dolomieu*, largemouth bass, white crappie *Pomoxis annularis*, yellow perch *Perca flavescens*, flathead catfish *Pylodictis olivaris*, freshwater drum *Aplodinotus grunniens* and yellow bass *Morone mississippiensis* (Anderson et al., 2023; Lampo et al., 2023). Anderson et al. (2023) found that in the Illinois River, white bass consumed greater quantities of bigheaded carps than any other native predator species they investigated, particularly during August and September when the frequency of occurrence of bigheaded carps in white bass diets was 60%. This is greater than the peak consumption of bigheaded carps by skipjack herring (of all sizes) that we observed in July (40% frequency of occurrence). However, several of the skipjack herring sampled by our study were <100 mm SL, sizes at which skipjack herring are still undergoing the transition to piscivory. The frequency of occurrence of bigheaded carps in skipjack herring >100 mm SL (*n* = 57) was 100% in July. Bigheaded carps grow rapidly (e.g. reaching ~110–220 mm TL at age 1 in the Illinois River), thus likely achieving sizes exceeding the gape limits of most skipjack herring in the Mississippi River Basin relatively quickly (Lampo et al., 2023). Furthermore, myriad interacting factors (bottom‐and top‐down forces) govern the composition of fish communities in the Mississippi River Basin (DeBoer et al., 2018; Delong, 2010; Love et al., 2018). Consequently, additional research is needed to determine if enhancement of skipjack herring population sizes, possibly in concert with other native fishes, can increase predation on bigheaded carps sufficiently to reduce population sizes in the Mississippi River Basin. Furthermore, feasible management actions that could potentially increase the abundances of skipjack herring and other native predators of bigheaded carps need to be identified.

Increases in maximum prey size consumption, maximum prey size to SL ratio and the volumes of large prey in diets accompanied changes in prey type consumption by skipjack herring. Only modest changes in median and minimum prey size consumption and median and minimum prey size to SL ratios occurred in juvenile skipjack herring. Increases in maximum prey size consumption through ontogeny with relatively moderate changes in median and minimum prey size consumption, have been documented in a range of clupeiform and non‐clupeiform fishes (Egan et al., 2017; Egan, Gibbs, & Simons, 2018; Gaeta et al., 2018; Niiranen et al., 2019; Scharf et al., 2000). For example, in a study of nine marine clupeiforms with SL ranges similar to those examined by this study, eight, nine and three species exhibited statistically significant increases in maximum, median and minimum prey size consumption, respectively (Egan, Gibbs, & Simons, 2018). Egan, Gibbs, and Simons (2018) reported that slopes from statistically significant maximum prey size regression equations varied widely among species but were all steeper than corresponding median and minimum prey size regression slopes, indicating greater ontogenetic increases in maximum prey size consumption compared to median and minimum prey size consumption. Although increasing prey size consumption during ontogeny is common in fishes, this pattern is not ubiquitous. For example, several fish species that undergo ontogenetic transitions from zooplanktivorous to detritivorous diets, or remain zooplanktivorous during ontogeny, exhibit relatively consistent maximum prey size consumption through ontogeny (Egan et al., 2017; Egan, Gibbs, & Simons, 2018; Gaeta et al., 2018; Niiranen et al., 2019; Scharf et al., 2000). Modest ontogenetic increases in minimum prey size consumption appear to be common in fishes regardless of trophic guild, but additional investigation of predator–prey size relationships on a wider range of species is warranted to better characterize the generality of this pattern (Egan et al., 2017; Egan, Gibbs, & Simons, 2018; Gaeta et al., 2018; Niiranen et al., 2019; Scharf et al., 2000).

A variety of forces drive ontogenetic diet shifts, which can vary considerably among species and ecosystems, contributing to substantial variation in the trophic ontogeny of fishes (Power, 1987; Sánchez‐Hernández et al., 2019). Ontogenetic transitions to piscivory appear to be facilitated in part by ontogenetic changes in the ability of fishes to capture larger and more evasive prey, such as increases in mouth gape and swimming performance (Budy et al., 2005; Christensen, 1996; Krebs & Turingan, 2003; Luecke, 1986; Scharf et al., 2000). We found that the ratio of maximum prey size to mouth gape remained relatively constant in skipjack herring, suggesting that mouth gape limits maximum prey size consumption by small skipjack herring and is a key determinant of the SLs at which skipjack herring begin consuming fishes (Scharf et al., 2000, 2009). We identified allometric changes in the mouth gape of juvenile skipjack herring; the ratio of mouth gape to SL increased until ~80 mm SL, at which point this ratio gradually declined. Maximum mouth gape to SL ratios corresponded to substantial increases in the consumption of fishes, indicating that mouth gape allometry may facilitate the transition to piscivory in the skipjack herring.

This study improves our understanding of the diet of the skipjack herring, a poorly studied mesopredator that has declined throughout much of its native range due to habitat degradation and the construction of impoundments (Carlander, 1954; Coker, 1930; Steuck et al., 2010). This work provides data that can be incorporated into future ecological and evolutionary research, such as the development of quantitative food web models that include the skipjack herring and account for ontogenetic diet shifts. Our finding that skipjack herring ate large quantities of bigheaded carps suggests that additional study is needed to determine if enhancement of native skipjack herring populations could be an effective tool for limiting population sizes of bigheaded carps. Further work is needed to describe the diets of skipjack herring during early and late ontogeny, and spring and winter because the present study was limited to individuals ranging from 28 to 231 mm SL collected from June through October. Research characterizing differences in the diets of skipjack herring occupying different habitats (e.g. estuarine versus freshwater) and in ecosystems with and without invasive bigheaded carps is also needed.

## AUTHOR CONTRIBUTIONS

Ideas: J.P.E. Data generation: J.P.E, P.J.H., P.J.H., N.P.R., J.A.D, B.J.L., K.A.M., L.E.S. and A.L.W.H. Data analysis: J.P.E. Manuscript preparation: J.P.E., P.J.H., N.P.R., J.A.D, B.J.L., K.A.M., L.E.S., A.L.W.H. and A.M.S. Funding: J.P.E. and A.M.S.

## FUNDING INFORMATION

This work was funded in part by the Dayton Research Fund (Bell Museum of Natural History, University of Minnesota), and the Minnesota Agricultural Experiment Station. During the preparation of this manuscript J.P.E. received financial support from a National Science Foundation Graduate Research Fellowship (00039202).

## CONFLICT OF INTEREST STATEMENT

The authors declare no they have no conflicts of interest.

## Supporting information


**TABLE S1.** Catalogue numbers (Catalogue #), number of specimens examined (*n*), collection locality (Locality) and collection date (date) associated with all skipjack herring *Alosa chrysochloris* collecting events associated with this study.
**TABLE S2.** Proportion of total prey volume subsampled and measured (proportion measured) for skipjack herring *Alosa chrysochloris* from which we did not measure all prey items dissected from the digestive tract.
**TABLE S3.** Average mouth gape measurements for skipjack herring *Alosa chrysochloris* individuals.
**TABLE S4.** Diet data used for analyses and associated information: James Ford Bell Museum catalogue number (Catalogue #), number assigned to individual skipjack herring *Alosa chrysochloris* within each museum jar (Individual), standard length of each skipjack herring (SL [mm]), prey type (Prey), widths of individual prey (Width [mm]), lengths of individual prey (Length [mm]) and volumes of individual prey (Volume (mm^3)).

## Data Availability

The data that supports the findings of this study are available in the Supporting Information of this article.
