# Supplementary material for: Diet of juvenile skipjack herring Alosa chrysochloris: Ontogenetic shifts, predator–prey size ratios and mouth gape allometry
Source: J Fish Biol. 2026 Mar 28;109(1):384–99. doi: 10.1111/jfb.70390 (PMC13397264; doi:10.1111/jfb.70390)
Supplement: Supplementary file 1 — TABLE S1. Catalogue numbers (Catalogue #), number of specimens examined (n), collection locality (Locality) and collection date (date) associated with all skipjack herring Alosa chrysochloris collecting events associated with this study. TABLE S2. Proportion of total prey volume subsampled and measured (proportion measured) for skipjack herring Alosa chrysochloris from which we did not measure all prey items dissected from the digestive tract. TABLE S3. Average mouth gape measurements for skipjack herring Alosa chrysochloris individuals. TABLE S4. Diet data used for analyses and associated information: James Ford Bell Museum catalogue number (Catalogue #), number assigned to individual skipjack herring Alosa chrysochloris within each museum jar (Individual), standard length of each skipjack herring (SL [mm]), prey type (Prey), widths of individual prey (Width [mm]), lengths of individual prey (Length [mm]) and volumes of individual prey (Volume (mm^3)). [file JFB-109-384-s001.docx]

**Supplementary materials for:**

**Diet of juvenile Skipjack Herring (*Alosa chrysochloris*): ontogenetic diet shifts, predator-prey size ratios, and mouth gape allometry**

**SUPPLEMENTARY TABLES**

**Supplementary Table 1.** Catalog numbers (Catalog #), number of specimens examined (n), collection locality (Locality), and collection date (date) associated with all Skipjack Herring (*Alosa chrysochloris*) collecting events associated with this study.

| **Catalog #** | **n** | **Locality** | **Date** |
| --- | --- | --- | --- |
| JFBM48451 | 2 | Illinois River (41.115422°N, 89.339721°W) | 2015 |
| JFBM48452 | 2 | Illinois River (41.115422°N, 89.339721°W) | 2015 |
| JFBM48476 | 5 | Illinois River (41.308117°N, 89.175317°W) | 7/27/2015 |
| JFBM48450 | 2 | Illinois River (39.137742°N, 90.612368°W) | 8/17/2015 |
| JFBM48454 | 1 | Illinois River (39.191724°N, 90.595131°W) | 8/17/2015 |
| JFBM48465 | 3 | Illinois River (39.396124°N, 90.614239°W) | 8/17/2015 |
| JFBM48469 | 5 | Illinois River (40.047549°N, 90.429824°W) | 8/17/2015 |
| JFBM48568 | 5 | Illinois River (39.124371°N, 90.605764°W) | 8/17/2015 |
| JFBM48258 | 3 | Illinois River (40.548193°N, 89.785077°W) | 8/20/2015 |
| JFBM48462 | 2 | Illinois River (40.529569°N, 89.835429°W) | 8/20/2015 |
| JFBM48989 | 32 | Illinois River (40.551653°N, 89.750163°W) | 8/20/2015 |
| JFBM49192 | 15 | Illinois River (40.552237°N, 89.738917°W) | 8/20/2015 |
| JFBM48563 | 5 | Illinois River (40.627562°N, 89.629370°W) | 8/21/2015 |
| JFBM48570 | 13 | Illinois River (40.321883°N, 90.054036°W) | 8/21/2015 |
| JFBM48468 | 2 | Illinois River (40.321883°N, 90.054036°W) | 8/24/2015 |
| JFBM48607 | 3 | Illinois River (40.315500°N, 90.063339°W) | 8/24/2015 |
| JFBM48470 | 13 | Illinois River (39.362677°N, 90.621912°W) | 8/25/2015 |
| JFBM48584 | 6 | Illinois River (40.209249°N, 90.136663°W) | 8/25/2015 |
| JFBM49180 | 4 | Illinois River (40.209196°N, 90.138886°W) | 8/25/2015 |
| JFBM48475 | 6 | Illinois River (40.918949°N, 89.472883°W) | 8/26/2015 |
| JFBM48537 | 6 | Illinois River (40.461066°N, 89.899967°W) | 8/26/2015 |
| JFBM48554 | 7 | Illinois River (39.415059°N, 90.617171°W) | 8/28/2015 |
| JFBM48565 | 5 | Illinois River (39.396124°N, 90.614239°W) | 8/28/2015 |
| JFBM48456 | 1 | Illinois River (38.966290°N, 90.490981°W) | 8/31/2015 |
| JFBM48457 | 1 | Illinois River (38.985018°N, 90.563519°W) | 8/31/2015 |
| JFBM48463 | 2 | Illinois River (38.600989°N, 90.191474°W) | 9/2/2015 |
| JFBM48464 | 1 | Illinois River (41.332068°N, 88.770556°W) | 9/21/2015 |
| JFBM48453 | 3 | Illinois River (39.998827°N, 90.487874°W) | 9/30/2015 |
| JFBM48931 | 1 | Illinois River (39.468166°N, 90.604817°W) | 10/5/2015 |
| JFBM49005 | 1 | Illinois River (40.055228°N, 90.404531°W) | 4/21/2016 |
| JFBM49028 | 1 | Illinois River (39.647969°N, 90.614925°W) | 7/15/2016 |
| JFBM49455 | 3 | Illinois River (39.607968°N, 90.599562°W) | 7/15/2016 |
| JFBM49006 | 1 | Illinois River (40.563968°N, 89.660981°W) | 8/11/2016 |
| JFBM49004 | 2 | Illinois River (40.551653°N, 89.750163°W) | 8/12/2016 |
| JFBM49003 | 2 | Illinois River (40.145553°N, 90.297214°W) | 8/29/2016 |
| JFBM49016 | 1 | Illinois River (40.604996°N, 89.647121°W) | 9/20/2016 |
| JFBM49457 | 12 | Illinois River (40.164301°N, 90.195497°W) | 10/10/2016 |
| JFBM49007 | 1 | Illinois River (40.499925°N, 89.883039°W) | 10/14/2016 |
| JFBM49015 | 1 | Illinois River (40.557010°N, 89.716830°W) | 10/28/2016 |
| JFBM49456 | 1 | Mississippi River (38.370523°N, 90.353279°W) | 8/13/2015 |
| JFBM48461 | 2 | Mississippi River (37.968806°N, 89.948171°W) | 8/27/2015 |
| JFBM49013 | 1 | Mississippi River (39.308538°N, 90.792810°W) | 10/19/2015 |
| JFBM49014 | 1 | Mississippi River (38.206540°N, 90.347630°W) | 10/28/2015 |
| JFBM49018 | 1 | Mississippi River (38.160896°N, 90.279833°W) | 10/28/2015 |
| JFBM48935 | 2 | Ohio River | 2015 |
| Uncataloged | 1 | Ohio River | 2015 |
| JFBM48606 | 28 | Ohio River (37.177341°N, 88.434401°W) | 6/30/2015 |
| JFBM48967 | 40 | Ohio River (37.561340°N, 88.103658°W) | 7/15/2015 |
| JFBM48535 | 8 | Ohio River (37.680970°N, 88.138736°W) | 7/16/2015 |
| JFBM48947 | 9 | Ohio River (37.189897°N, 88.817830°W) | 7/22/2015 |
| JFBM48948 | 68 | Ohio River (37.226555°N, 88.956174°W) | 7/23/2015 |
| JFBM48585 | 52 | Ohio River (37.011962°N, 89.171102°W) | 10/6/2015 |
| JFBM48562 | 2 | Ohio River (37.414180°N, 88.389215°W) | 10/13/2015 |
| JFBM48678 | 11 | Ohio River (37.066295°N, 88.542155°W) | 11/8/2015 |
| JFBM49017 | 6 | Ohio River | 6/29/2016 |
| JFBM49183 | 3 | Ohio River (37.113721°N, 88.612813°W) | 6/30/2016 |
| JFBM49459 | 7 | Ohio River (37.167995°N, 89.071516°W) | 8/5/2016 |
| JFBM49029 | 9 | Ohio River (37.200574°N, 88.448035°W) | 9/21/2016 |
| JFBM49181 | 3 | Ohio River (37.680970°N, 88.138736°W) | 10/24/2016 |
| JFBM49179 | 6 | Ohio River (37.2091°N, 88.8781°W) | 6/21/2017 |
| **Total** | **442** |  |  |

**Supplementary Table 2.** Proportion of total prey volume subsampled and measured (proportion measured) for Skipjack Herring from which we did not measure all prey items dissected from the digestive tract.

| **Catalog #** | **Individual** | **Proportion measured** |
| --- | --- | --- |
| JFBM48476 | 5 | 0.25 |
| JFBM48568 | 4 | 0.25 |
| JFBM48967 | 39 | 0.25 |
| JFBM49181 | 2 | 0.5 |
| JFBM49181 | 3 | 0.5 |
| JFBM49455 | 3 | 0.16 |

**Supplementary Table 3.** Average mouth gape measurements for Skipjack Herring individuals.

| Catalog # | Individual | SL (mm) | Mouth gape (mm) |
| --- | --- | --- | --- |
| JFBM48606 | 28 | 28.25 | 2.083 |
| JFBM48606 | 11 | 29.17 | 2.283 |
| JFBM48606 | 27 | 30.09 | 2.293 |
| JFBM48606 | 5 | 35.15 | 2.907 |
| JFBM48606 | 16 | 35.44 | 3.140 |
| JFBM48606 | 19 | 36.66 | 3.150 |
| JFBM48606 | 18 | 37.22 | 3.463 |
| JFBM48606 | 12 | 37.97 | 3.750 |
| JFBM48606 | 4 | 38.59 | 3.843 |
| JFBM48606 | 2 | 39.04 | 3.917 |
| JFBM48606 | 13 | 42.9 | 4.077 |
| JFBM48606 | 10 | 45.94 | 4.493 |
| JFBM48606 | 8 | 49.94 | 4.757 |
| JFBM48470 | 7 | 51.95 | 5.993 |
| JFBM48470 | 3 | 51.97 | 5.977 |
| JFBM48967 | 9 | 53.06 | 5.420 |
| JFBM48606 | 9 | 56.07 | 5.630 |
| JFBM48470 | 6 | 59.57 | 5.980 |
| JFBM48967 | 25 | 59.88 | 5.877 |
| JFBM48967 | 4 | 62.14 | 6.347 |
| JFBM48989 | 10 | 65.18 | 7.503 |
| JFBM48989 | 25 | 69.77 | 7.887 |
| JFBM48967 | 22 | 72.14 | 7.673 |
| JFBM48585 | 25 | 72.17 | 8.010 |
| JFBM48989 | 24 | 76.02 | 9.197 |
| JFBM48585 | 44 | 76.11 | 9.147 |
| JFBM48607 | 2 | 79.98 | 9.367 |
| JFBM48585 | 38 | 80.65 | 9.947 |
| JFBM48989 | 20 | 81.42 | 9.937 |
| JFBM48585 | 37 | 81.85 | 10.150 |
| JFBM48585 | 50 | 84.49 | 10.343 |
| JFBM48585 | 20 | 84.52 | 10.227 |
| JFBM48585 | 14 | 88.91 | 10.590 |
| JFBM48585 | 12 | 92.76 | 10.987 |
| JFBM48585 | 9 | 95.52 | 11.237 |
| JFBM48585 | 6 | 97.61 | 11.327 |
| JFBM48585 | 10 | 99.17 | 11.483 |
| JFBM49004 | 1 | 101.13 | 11.597 |
| JFBM49003 | 2 | 105.15 | 11.910 |
| JFBM49004 | 2 | 107.79 | 12.033 |
| JFBM48948 | 67 | 110.67 | 12.247 |
| JFBM48947 | 6 | 116.25 | 13.020 |
| JFBM48570 | 11 | 119.66 | 13.437 |
| JFBM48947 | 1 | 122.23 | 13.830 |
| JFBM49192 | 5 | 125.4 | 14.097 |
| JFBM48678 | 5 | 130.75 | 14.397 |
| JFBM48554 | 7 | 137.87 | 15.067 |
| JFBM49007 | 1 | 141.4 | 15.187 |
| JFBM49457 | 7 | 146.99 | 16.250 |
| JFBM48537 | 1 | 150.66 | 16.613 |
| JFBM48456 | 1 | 153.55 | 16.760 |
| JFBM49459 | 1 | 155.71 | 16.817 |
| JFBM48935 | 1 | 159.04 | 16.937 |
| JFBM48453 | 1 | 159.14 | 16.963 |
| JFBM49016 | 1 | 164.14 | 17.807 |
| JFBM48562 | 1 | 167.71 | 17.737 |
| JFBM49183 | 1 | 171.29 | 18.460 |
| JFBM48452 | 1 | 177.05 | 18.580 |
| JFBM49013 | 1 | 184.24 | 18.770 |
| JFBM48452 | 2 | 196.16 | 18.967 |

**Supplementary Table 4.** Diet data used for analyses and associated information: James Ford Bell Museum catalog number (Catalog #), number assigned to individual Skipjack Herring (*Alosa chrysochloris*) within each museum jar (Individual), standard length of each skipjack (SL (mm)), prey type (Prey), widths of individual prey (Width (mm)), lengths of individual prey (Length (mm)), and volumes of individual prey (Volume (mm^3)).

| **Catalog #** | **Individual** | **SL (mm)** | **Prey** | **Width (mm)** | **Length (mm)** | **Volume (mm^3)** |
| --- | --- | --- | --- | --- | --- | --- |
| UNCATALOGED | 1 | 173 | Fish unidentifiable | 8.132 |  | 2933.544 |
| JFBM48450 | 2 | 104 | Trichoptera larvae | 1.381 | 7.04 | 14.53588 |
| JFBM48450 | 2 | 104 | Trichoptera larvae | 0.895 | 5.512 | 5.04937 |
| JFBM48450 | 2 | 104 | Trichoptera larvae | 1.593 | 9.695 | 0.917 |
| JFBM48451 | 2 | 122.66 | Diptera terrestrial | 1.035 |  | 1.8814 |
| JFBM48452 | 1 | 177.05 | Ants | 1.231 |  | 2.35366 |
| JFBM48452 | 1 | 177.05 | Ants | 1.074 |  | 1.97573 |
| JFBM48452 | 1 | 177.05 | Ants | 1.142 |  | 2.13942 |
| JFBM48452 | 1 | 177.05 | Ants | 0.961 |  | 1.70372 |
| JFBM48452 | 1 | 177.05 | Ants | 1.092 |  | 2.01906 |
| JFBM48452 | 1 | 177.05 | Ants | 1.056 |  | 1.9324 |
| JFBM48452 | 2 | 196.16 | Insecta terrestrial Coleoptera | 1.245 |  | 2.38736 |
| JFBM48452 | 2 | 196.16 | Insecta terrestrial Coleoptera | 1.197 |  | 2.27182 |
| JFBM48452 | 2 | 196.16 | Insecta terrestrial Hymenoptera | 1.583 |  | 3.201 |
| JFBM48452 | 2 | 196.16 | Insecta terrestrial Hymenoptera | 1.387 |  | 2.72919 |
| JFBM48452 | 2 | 196.16 | Insecta terrestrial Hymenoptera | 1.232 |  | 2.35607 |
| JFBM48452 | 2 | 196.16 | Insecta terrestrial Hymenoptera | 1.413 |  | 2.79177 |
| JFBM48452 | 2 | 196.16 | Insecta terrestrial Hymenoptera | 1.631 |  | 3.31654 |
| JFBM48452 | 2 | 196.16 | Insecta terrestrial Hymenoptera | 1.429 |  | 2.83029 |
| JFBM48452 | 2 | 196.16 | Insecta terrestrial Hymenoptera | 1.513 |  | 3.03249 |
| JFBM48452 | 2 | 196.16 | Insecta terrestrial Hymenoptera | 1.07 |  | 1.9661 |
| JFBM48452 | 2 | 196.16 | Insecta terrestrial Hymenoptera | 1.266 |  | 2.43792 |
| JFBM48453 | 1 | 159.14 | Insecta terrestrial Hymenoptera | 0.871 |  | 1.48707 |
| JFBM48453 | 2 | 80.01 | Trichoptera larva | 0.866 |  | 4.43842 |
| JFBM48453 | 2 | 80.01 | Trichoptera larva | 0.926 |  | 4.94062 |
| JFBM48456 | 1 | 153.55 | Fish | 6.986 |  | 1166.412 |
| JFBM48457 | 1 | 150.66 | *Lepomis humilis* | 12.475 |  | 9630.45 |
| JFBM48461 | 1 | 153.2 | Fish *Hypophthalmichthys* | 10.417 |  | 6457.014 |
| JFBM48461 | 1 | 153.2 | Fish *Hypophthalmichthys* | 7.637 |  | 2170.254 |
| JFBM48461 | 1 | 153.2 | Fish *Dorosoma* | 13.187 |  | 10728.354 |
| JFBM48463 | 1 | 128.06 | Insecta terrestrial Hymenoptera | 2.518 |  | 5.45173 |
| JFBM48463 | 1 | 128.06 | Insecta terrestrial Hymenoptera | 2.906 |  | 6.38572 |
| JFBM48463 | 1 | 128.06 | Insecta terrestrial Hymenoptera | 2.113 |  | 4.47681 |
| JFBM48463 | 1 | 128.06 | Insecta terrestrial Hymenoptera | 2.25 |  | 4.8066 |
| JFBM48463 | 1 | 128.06 | Insecta terrestrial Hymenoptera | 2.268 |  | 4.84993 |
| JFBM48463 | 1 | 128.06 | Insecta terrestrial Hymenoptera | 2.265 |  | 4.84271 |
| JFBM48463 | 1 | 128.06 | Trichoptera larva | 2.177 |  | 15.41149 |
| JFBM48463 | 1 | 128.06 | Fish | 6.679 |  | 693.018 |
| JFBM48463 | 2 | 98.71 | Trichoptera larva | 1.418 |  | 9.05866 |
| JFBM48463 | 2 | 98.71 | Trichoptera larva | 1.577 |  | 10.38949 |
| JFBM48463 | 2 | 98.71 | Trichoptera larva | 1.829 |  | 12.49873 |
| JFBM48463 | 2 | 98.71 | Trichoptera larva | 1.26 |  | 7.7362 |
| JFBM48463 | 2 | 98.71 | Insecta terrestrial Hymenoptera | 1.813 |  | 3.75465 |
| JFBM48464 | 1 | 150.33 | Insecta terrestrial Hymenoptera | 1.261 | 4.306 | 7.9613 |
| JFBM48465 | 3 | 155.61 | Fish *Gambusia affinis* | 6.266 | 25.112 | 972.81607 |
| JFBM48468 | 1 | 77.39 | Trichoptera larva | 1.524 |  | 9.94588 |
| JFBM48468 | 2 | 147.35 | Insecta terrestrial Diptera | 1.333 |  | 2.5992 |
| JFBM48468 | 2 | 147.35 | *Notropis atherinoides* | 7.891 |  | 2561.922 |
| JFBM48469 | 1 | 128.73 | *Lepomis* spp. | 10.62 |  | 6770.04 |
| JFBM48469 | 1 | 128.73 | Plecoptera | 1.421 |  | 2.81103 |
| JFBM48469 | 2 | 120.78 | *Gambusia affinis* | 5.75 |  | 755.5 |
| JFBM48469 | 2 | 120.78 | Hemiptera terrestrial | 1.787 |  | 3.69207 |
| JFBM48469 | 2 | 120.78 | *Notropis atherinoides* | 5.057 |  | 512.95 |
| JFBM48469 | 2 | 120.78 | *Notropis atherinoides* | 6.161 |  | 899.35 |
| JFBM48469 | 3 | 87.33 | Trichoptera larva aquatic | 0.766 |  | 3.60142 |
| JFBM48469 | 3 | 87.33 | Trichoptera larva aquatic | 0.778 |  | 3.70186 |
| JFBM48469 | 4 | 86.22 | Daphnia | 0.394 | 0.635 | 0.09474 |
| JFBM48469 | 4 | 86.22 | Daphnia | 0.314 |  | 0.01521 |
| JFBM48469 | 4 | 86.22 | Daphnia | 0.408 | 0.761 | 0.12945 |
| JFBM48469 | 4 | 86.22 | Daphnia | 0.347 | 0.625 | 0.16903 |
| JFBM48469 | 4 | 86.22 | Daphnia | 0.347 | 0.626 | 0.15789 |
| JFBM48469 | 4 | 86.22 | Copepoda | 0.388 | 0.762 | 0.16195 |
| JFBM48469 | 4 | 86.22 | Daphnia | 0.388 | 0.64 | 0.108 |
| JFBM48469 | 4 | 86.22 | Daphnia | 0.336 | 0.729 | 0.0191 |
| JFBM48469 | 4 | 86.22 | Daphnia | 0.291 | 0.661 | 0.05024 |
| JFBM48469 | 4 | 86.22 | Daphnia | 0.351 | 0.603 | 0.11697 |
| JFBM48469 | 4 | 86.22 | Daphnia | 0.304 | 0.549 | 0.07702 |
| JFBM48469 | 4 | 86.22 | Daphnia | 0.309 | 0.682 | 0.11822 |
| JFBM48469 | 4 | 86.22 | Daphnia | 0.371 | 0.741 | 0.11081 |
| JFBM48469 | 4 | 86.22 | Daphnia | 0.383 | 0.749 | 0.05133 |
| JFBM48469 | 4 | 86.22 | Daphnia | 0.444 | 0.77 | 0.08581 |
| JFBM48469 | 4 | 86.22 | Daphnia | 0.312 | 0.559 | 0.04071 |
| JFBM48469 | 4 | 86.22 | Daphnia | 0.333 | 0.631 | 0.06533 |
| JFBM48469 | 4 | 86.22 | Daphnia | 0.4 | 0.957 | 0.07562 |
| JFBM48469 | 4 | 86.22 | Daphnia | 0.369 | 0.511 | 0.09083 |
| JFBM48469 | 4 | 86.22 | Daphnia | 0.359 | 0.622 | 0.08235 |
| JFBM48469 | 4 | 86.22 | Daphnia | 0.331 |  | 0.0182 |
| JFBM48469 | 4 | 86.22 | Daphnia | 0.361 | 0.559 | 0.09798 |
| JFBM48469 | 4 | 86.22 | Daphnia | 0.369 |  | 0.027 |
| JFBM48469 | 4 | 86.22 | Daphnia | 0.408 |  | 0.04027 |
| JFBM48469 | 4 | 86.22 | Daphnia | 0.29 | 0.735 | 0.07823 |
| JFBM48469 | 4 | 86.22 | Daphnia | 0.32 |  | 0.01633 |
| JFBM48469 | 4 | 86.22 | Daphnia | 0.384 | 0.76 | 0.1147 |
| JFBM48469 | 4 | 86.22 | Daphnia | 0.315 |  | 0.01543 |
| JFBM48469 | 4 | 86.22 | Daphnia | 0.333 |  | 0.01853 |
| JFBM48469 | 4 | 86.22 | Daphnia | 0.364 |  | 0.02555 |
| JFBM48469 | 4 | 86.22 | Daphnia | 0.348 |  | 0.02176 |
| JFBM48469 | 4 | 86.22 | Daphnia | 0.315 | 0.622 | 0.12706 |
| JFBM48469 | 4 | 86.22 | Daphnia | 0.308 | 0.621 | 0.08644 |
| JFBM48469 | 4 | 86.22 | Daphnia | 0.391 | 0.912 | 0.17985 |
| JFBM48469 | 4 | 86.22 | Daphnia | 0.367 | 0.67 | 0.23384 |
| JFBM48469 | 4 | 86.22 | Daphnia | 0.347 |  | 0.02144 |
| JFBM48469 | 4 | 86.22 | Daphnia | 0.354 | 0.666 | 0.11141 |
| JFBM48469 | 4 | 86.22 | Daphnia | 0.475 | 0.708 | 0.19534 |
| JFBM48469 | 4 | 86.22 | Daphnia | 0.347 |  | 0.02153 |
| JFBM48469 | 4 | 86.22 | Daphnia | 0.347 |  | 0.02145 |
| JFBM48469 | 4 | 86.22 | Daphnia | 0.393 |  | 0.03441 |
| JFBM48469 | 4 | 86.22 | Daphnia | 0.424 | 0.837 | 0.10016 |
| JFBM48469 | 4 | 86.22 | Daphnia | 0.433 |  | 0.05199 |
| JFBM48469 | 4 | 86.22 | Daphnia | 0.454 | 0.747 | 0.1408 |
| JFBM48469 | 4 | 86.22 | Daphnia | 0.383 |  | 0.03116 |
| JFBM48469 | 4 | 86.22 | Daphnia | 0.322 |  | 0.01662 |
| JFBM48469 | 4 | 86.22 | Daphnia | 0.417 | 0.697 | 0.21045 |
| JFBM48469 | 4 | 86.22 | Daphnia | 0.346 |  | 0.02117 |
| JFBM48469 | 4 | 86.22 | Daphnia | 0.368 |  | 0.0266 |
| JFBM48469 | 4 | 86.22 | Daphnia | 0.333 | 0.82 | 0.12026 |
| JFBM48469 | 4 | 86.22 | Daphnia | 0.365 | 0.846 | 0.08669 |
| JFBM48469 | 4 | 86.22 | Daphnia | 0.367 |  | 0.02645 |
| JFBM48469 | 4 | 86.22 | Daphnia | 0.376 | 0.807 | 0.11928 |
| JFBM48469 | 4 | 86.22 | Daphnia | 0.41 | 0.757 | 0.14984 |
| JFBM48469 | 4 | 86.22 | Daphnia | 0.542 |  | 0.16132 |
| JFBM48469 | 4 | 86.22 | Daphnia | 0.46 | 0.676 | 0.1038 |
| JFBM48469 | 4 | 86.22 | Daphnia | 0.412 |  | 0.0421 |
| JFBM48469 | 4 | 86.22 | Daphnia | 0.311 | 0.749 | 0.07791 |
| JFBM48469 | 4 | 86.22 | Daphnia | 0.288 | 0.538 | 0.08588 |
| JFBM48469 | 5 | 56.43 | Coleoptera terrestrial | 1.372 |  | 2.69333 |
| JFBM48470 | 1 | 56.43 | Ostracoda | 0.17 |  | 0.00097 |
| JFBM48470 | 1 | 56.43 | Ostracoda | 0.197 |  | 0.00219 |
| JFBM48470 | 1 | 56.43 | Ostracoda | 0.167 |  | 0.00084 |
| JFBM48470 | 1 | 56.43 | Ostracoda | 0.196 |  | 0.00216 |
| JFBM48470 | 1 | 56.43 | Ostracoda | 0.155 |  | 0.00031 |
| JFBM48470 | 1 | 56.43 | Ostracoda | 0.167 |  | 0.00085 |
| JFBM48470 | 1 | 56.43 | Ostracoda | 0.179 |  | 0.00141 |
| JFBM48470 | 1 | 56.43 | Ostracoda | 0.153 |  | 0.00023 |
| JFBM48470 | 1 | 56.43 | Ostracoda | 0.203 |  | 0.00245 |
| JFBM48470 | 1 | 56.43 | Ostracoda | 0.175 |  | 0.0012 |
| JFBM48470 | 1 | 56.43 | Ostracoda | 0.173 |  | 0.0011 |
| JFBM48470 | 1 | 56.43 | Ostracoda | 0.185 |  | 0.00167 |
| JFBM48470 | 1 | 56.43 | Ostracoda | 0.174 |  | 0.00116 |
| JFBM48470 | 1 | 56.43 | Ostracoda | 0.178 |  | 0.00133 |
| JFBM48470 | 1 | 56.43 | Ostracoda | 0.202 |  | 0.00242 |
| JFBM48470 | 1 | 56.43 | Ostracoda | 0.186 |  | 0.00169 |
| JFBM48470 | 2 | 72.24 | Ostracoda | 0.185 |  | 0.00167 |
| JFBM48470 | 3 | 51.97 | Filamentous algae | 0.016 |  | 0.00007 |
| JFBM48470 | 3 | 51.97 | Insecta terrestrial | 0.381 |  | 0.37503 |
| JFBM48470 | 4 | 58.89 | Filamentous algae | 0.016 |  | 0.00007 |
| JFBM48470 | 4 | 58.89 | Filamentous algae | 0.014 |  | 0.00006 |
| JFBM48470 | 4 | 58.89 | Ostracoda | 0.179 |  | 0.0014 |
| JFBM48470 | 4 | 58.89 | Ostracoda | 0.155 |  | 0.00033 |
| JFBM48470 | 4 | 58.89 | Ostracoda | 0.198 |  | 0.00222 |
| JFBM48470 | 4 | 58.89 | Ostracoda | 0.196 |  | 0.00215 |
| JFBM48470 | 5 | 58.75 | Copepoda | 0.13 |  | 0.00229 |
| JFBM48470 | 5 | 58.75 | Filamentous algae | 0.014 |  | 0.00006 |
| JFBM48470 | 5 | 58.75 | Filamentous algae | 0.017 |  | 0.00008 |
| JFBM48470 | 5 | 58.75 | Filamentous algae | 0.009 |  | 0.00004 |
| JFBM48470 | 6 | 59.57 | Ostracoda | 0.421 |  | 0.01218 |
| JFBM48470 | 7 | 51.95 | Copepoda | 0.166 |  | 0.00331 |
| JFBM48470 | 8 | 58.35 | Insecta terrestrial | 0.339 |  | 0.20714 |
| JFBM48470 | 8 | 58.35 | Ostracoda | 0.18 |  | 0.00143 |
| JFBM48470 | 9 | 59.45 | Copepoda | 0.17 |  | 0.00347 |
| JFBM48470 | 9 | 59.45 | Daphnia | 0.151 |  | 0.00285 |
| JFBM48470 | 9 | 59.45 | Daphnia | 0.117 |  | 0.002 |
| JFBM48470 | 9 | 59.45 | Daphnia | 0.188 |  | 0.00415 |
| JFBM48470 | 9 | 59.45 | Daphnia | 0.136 |  | 0.00243 |
| JFBM48470 | 9 | 59.45 | Daphnia | 0.151 |  | 0.00286 |
| JFBM48470 | 9 | 59.45 | Daphnia | 0.17 |  | 0.00347 |
| JFBM48470 | 9 | 59.45 | Daphnia | 0.151 |  | 0.00285 |
| JFBM48470 | 9 | 59.45 | Daphnia | 0.155 |  | 0.00295 |
| JFBM48470 | 9 | 59.45 | Daphnia | 0.171 |  | 0.0035 |
| JFBM48470 | 9 | 59.45 | Filamentous Algae | 0.013 |  | 0.00006 |
| JFBM48470 | 9 | 59.45 | Filamentous Algae | 0.026 |  | 0.00011 |
| JFBM48470 | 9 | 59.45 | Ostracoda | 0.154 |  | 0.00029 |
| JFBM48470 | 9 | 59.45 | Ostracoda | 0.15 |  | 0.00007 |
| JFBM48470 | 9 | 59.45 | Ostracoda | 0.225 |  | 0.00342 |
| JFBM48470 | 9 | 59.45 | Ostracoda | 0.166 |  | 0.00082 |
| JFBM48470 | 9 | 59.45 | Diptera larva | 0.096 |  | 0.19071 |
| JFBM48470 | 10 | 61.62 | Copepoda | 0.134 |  | 0.00238 |
| JFBM48470 | 10 | 61.62 | Copepoda | 0.091 |  | 0.00152 |
| JFBM48470 | 10 | 61.62 | Filamentous Algae | 0.013 |  | 0.00006 |
| JFBM48470 | 10 | 61.62 | Gastropoda larvae | 0.14 |  | 0.00253 |
| JFBM48470 | 10 | 61.62 | Ostracoda | 0.168 |  | 0.00089 |
| JFBM48470 | 10 | 61.62 | Ostracoda | 0.123 |  | 0.00213 |
| JFBM48470 | 10 | 61.62 | Ostracoda | 0.214 |  | 0.00293 |
| JFBM48470 | 10 | 61.62 | Ostracoda | 0.168 |  | 0.0009 |
| JFBM48470 | 10 | 61.62 | Ostracoda | 0.165 |  | 0.00077 |
| JFBM48470 | 10 | 61.62 | Ostracoda | 0.159 |  | 0.00047 |
| JFBM48470 | 10 | 61.62 | Plant matter | 0.04 |  | 0.0009 |
| JFBM48470 | 10 | 61.62 | Ostracoda | 0.183 |  | 0.00156 |
| JFBM48470 | 10 | 61.62 | Ostracoda | 0.166 |  | 0.00079 |
| JFBM48470 | 10 | 61.62 | Ostracoda | 0.172 |  | 0.00107 |
| JFBM48470 | 10 | 61.62 | Ostracoda | 0.176 |  | 0.00123 |
| JFBM48470 | 10 | 61.62 | Ostracoda | 0.179 |  | 0.00137 |
| JFBM48470 | 10 | 61.62 | Ostracoda | 0.196 |  | 0.00214 |
| JFBM48470 | 10 | 61.62 | Ostracoda | 0.191 |  | 0.00191 |
| JFBM48470 | 10 | 61.62 | Ostracoda | 0.072 |  | 0.00126 |
| JFBM48470 | 10 | 61.62 | Ostracoda | 0.199 |  | 0.00227 |
| JFBM48470 | 10 | 61.62 | Ostracoda | 0.196 |  | 0.00216 |
| JFBM48470 | 10 | 61.62 | Plant matter | 0.064 |  | 0.00116 |
| JFBM48470 | 10 | 61.62 | Plant matter | 0.078 |  | 0.00134 |
| JFBM48470 | 10 | 61.62 | Plant matter | 0.059 |  | 0.0011 |
| JFBM48470 | 11 | 67 | Copepoda | 0.321 |  | 0.00773 |
| JFBM48470 | 11 | 67 | Copepoda | 0.181 |  | 0.00389 |
| JFBM48470 | 11 | 67 | Copepoda | 0.136 |  | 0.00243 |
| JFBM48470 | 11 | 67 | Copepoda | 0.147 |  | 0.00272 |
| JFBM48470 | 11 | 67 | Copepoda | 0.115 |  | 0.00197 |
| JFBM48470 | 11 | 67 | Copepoda | 0.145 |  | 0.00267 |
| JFBM48470 | 11 | 67 | Daphnia | 0.092 |  | 0.00155 |
| JFBM48470 | 11 | 67 | Daphnia | 0.173 |  | 0.00113 |
| JFBM48470 | 11 | 67 | Ostracoda | 0.164 |  | 0.00073 |
| JFBM48470 | 11 | 67 | Ostracoda | 0.196 |  | 0.00216 |
| JFBM48470 | 11 | 67 | Ostracoda | 0.158 |  | 0.00046 |
| JFBM48470 | 11 | 67 | Ostracoda | 0.136 |  | 0.00243 |
| JFBM48470 | 11 | 67 | Ostracoda | 0.188 |  | 0.00177 |
| JFBM48470 | 11 | 67 | Trichoptera larva | 0.069 |  | 0.18227 |
| JFBM48470 | 11 | 67 | Trichoptera larva | 0.148 |  | 0.20743 |
| JFBM48470 | 12 | 81.34 | Filamentous Algae | 0.014 |  | 0.00006 |
| JFBM48470 | 12 | 81.34 | Filamentous Algae | 0.011 |  | 0.00005 |
| JFBM48470 | 12 | 81.34 | Filamentous Algae | 0.009 |  | 0.00004 |
| JFBM48470 | 12 | 81.34 | Filamentous Algae | 0.011 |  | 0.00005 |
| JFBM48470 | 12 | 81.34 | Filamentous Algae | 0.011 |  | 0.00005 |
| JFBM48470 | 12 | 81.34 | Filamentous Algae | 0.012 |  | 0.00005 |
| JFBM48470 | 12 | 81.34 | Gastropoda larvae | 0.12 |  | 0.00207 |
| JFBM48470 | 12 | 81.34 | Gastropoda larvae | 0.133 |  | 0.00237 |
| JFBM48470 | 12 | 81.34 | Gastropoda larvae | 0.154 |  | 0.00292 |
| JFBM48470 | 12 | 81.34 | Copepoda | 0.233 |  | 0.00663 |
| JFBM48470 | 13 | 111.61 | Hemiptera aquatic | 1.813 |  | 12.36481 |
| JFBM48470 | 13 | 111.61 | Trichoptera larva | 1.333 |  | 8.34721 |
| JFBM48470 | 13 | 111.61 | Trichoptera larva | 1.623 |  | 10.77451 |
| JFBM48475 | 3 | 120.24 | Diptera larvae | 0.551 |  | 0.71677 |
| JFBM48475 | 3 | 120.24 | Hemioptera larva | 1.506 |  | 9.79522 |
| JFBM48475 | 3 | 120.24 | Trichoptera larva | 0.733 |  | 3.32521 |
| JFBM48475 | 6 | 89.73 | Diptera terrestrial | 0.641 | 1.409 | 0.73036 |
| JFBM48475 | 6 | 89.73 | Araneae | 0.858 | 2.15 | 1.45578 |
| JFBM48475 | 6 | 89.73 | Araneae | 1.341 | 2.89 | 0.60697 |
| JFBM48475 | 6 | 89.73 | Araneae | 1.208 | 2.414 | 0.50256 |
| JFBM48475 | 6 | 89.73 | Araneae | 1.086 | 1.906 | 0.2582 |
| JFBM48475 | 6 | 89.73 | Insecta terrestrial | 0.672 | 1.837 | 0.63653 |
| JFBM48475 | 6 | 89.73 | Insecta terrestrial | 0.416 | 1.576 | 0.32163 |
| JFBM48475 | 6 | 89.73 | Insecta terrestrial | 1.051 | 2.885 | 3.17404 |
| JFBM48475 | 6 | 89.73 | Trichoptera larva | 1.646 |  | 10.96702 |
| JFBM48475 | 6 | 89.73 | Trichoptera larva | 1.392 |  | 8.84104 |
| JFBM48475 | 6 | 89.73 | Trichoptera larva | 1.935 |  | 13.38595 |
| JFBM48475 | 6 | 89.73 | Trichoptera larva | 1.536 |  | 10.04632 |
| JFBM48475 | 6 | 89.73 | Trichoptera larva | 1.792 |  | 12.18904 |
| JFBM48475 | 6 | 89.73 | Trichoptera larva | 1.549 |  | 10.15513 |
| JFBM48475 | 6 | 89.73 | Trichoptera larva | 1.495 |  | 9.70315 |
| JFBM48475 | 6 | 89.73 | Trichoptera larva | 1.428 |  | 9.14236 |
| JFBM48475 | 6 | 89.73 | Trichoptera larva | 1.953 |  | 13.53661 |
| JFBM48475 | 6 | 89.73 | Trichoptera larva | 1.598 |  | 10.56526 |
| JFBM48475 | 6 | 89.73 | Trichoptera larva | 2.209 |  | 15.67933 |
| JFBM48475 | 6 | 89.73 | Trichoptera larva | 1.448 |  | 9.30976 |
| JFBM48475 | 6 | 89.73 | Trichoptera larva | 1.762 |  | 11.93794 |
| JFBM48475 | 6 | 89.73 | Trichoptera larva | 1.476 |  | 9.54412 |
| JFBM48475 | 6 | 89.73 | Trichoptera larva | 1.262 |  | 7.75294 |
| JFBM48475 | 6 | 89.73 | Trichoptera larva | 1.209 |  | 7.30933 |
| JFBM48475 | 6 | 89.73 | Trichoptera larva | 1.236 |  | 7.53532 |
| JFBM48475 | 6 | 89.73 | Trichoptera larva | 1.088 |  | 6.29656 |
| JFBM48475 | 6 | 89.73 | Trichoptera larva | 1.115 |  | 6.52255 |
| JFBM48475 | 6 | 89.73 | Trichoptera larva | 1.231 |  | 7.49347 |
| JFBM48475 | 6 | 89.73 | Trichoptera larva | 1.539 |  | 10.07143 |
| JFBM48475 | 6 | 89.73 | Trichoptera larva | 1.231 |  | 7.49347 |
| JFBM48475 | 6 | 89.73 | Trichoptera larva | 1.665 |  | 11.12605 |
| JFBM48475 | 6 | 89.73 | Trichoptera larva | 1.679 | 9.464 | 15.45142 |
| JFBM48475 | 6 | 89.73 | Trichoptera larva | 1.331 | 7.676 | 9.81899 |
| JFBM48475 | 6 | 89.73 | Trichoptera larva | 1.579 | 8.099 | 9.9801 |
| JFBM48475 | 6 | 89.73 | Trichoptera larva | 1.52 | 7.714 | 7.74867 |
| JFBM48476 | 1 | 212.82 | Fish (*Notropis atherinoides*) | 9.36 | 44.86 | 3107.11665 |
| JFBM48476 | 2 | 113.68 | Diptera larvae | 0.454 |  | 0.98998 |
| JFBM48476 | 2 | 113.68 | Diptera larvae | 2.305 |  | 16.48285 |
| JFBM48476 | 2 | 113.68 | Diptera pupae | 2.04 |  | 14.2648 |
| JFBM48476 | 2 | 113.68 | Insecta Trichoptera terrestrial | 0.991 |  | 1.77594 |
| JFBM48476 | 2 | 113.68 | Insecta Trichoptera terrestrial | 0.721 |  | 1.12599 |
| JFBM48476 | 2 | 113.68 | Insecta Trichoptera terrestrial | 0.64 |  | 0.93101 |
| JFBM48476 | 2 | 113.68 | Insecta Trichoptera terrestrial | 1.118 |  | 2.08165 |
| JFBM48476 | 2 | 113.68 | Insecta Trichoptera terrestrial | 0.965 |  | 1.71335 |
| JFBM48476 | 3 | 120.24 | Amphipoda | 1.216 |  | 3.61853 |
| JFBM48476 | 3 | 120.24 | Insecta Hemioptera terrestrial | 0.697 |  | 1.06822 |
| JFBM48476 | 3 | 120.24 | Insecta Hemioptera terrestrial | 0.676 |  | 1.01767 |
| JFBM48476 | 3 | 120.24 | Insecta Hemioptera terrestrial | 1.124 |  | 2.09609 |
| JFBM48476 | 3 | 120.24 | Trichoptera aquatic | 1.435 |  | 9.20095 |
| JFBM48476 | 3 | 120.24 | Trichoptera aquatic | 0.417 |  | 0.68029 |
| JFBM48476 | 4 | 120.82 | Coleoptera | 1.027 |  | 5.78599 |
| JFBM48476 | 4 | 120.82 | Diptera larvae | 0.472 | 5.516 | 1.07365 |
| JFBM48476 | 4 | 120.82 | Diptera larvae | 0.47 | 5.1 | 0.65082 |
| JFBM48476 | 4 | 120.82 | Diptera pupae | 0.746 | 3.658 | 1.65299 |
| JFBM48476 | 4 | 120.82 | Diptera pupae | 1.59 | 7.933 | 24.71486 |
| JFBM48476 | 4 | 120.82 | Insecta Hemioptera terrestrial | 0.749 | 2.186 | 2.12503 |
| JFBM48476 | 4 | 120.82 | Insecta Hemioptera terrestrial | 1.671 | 3.989 | 16.84923 |
| JFBM48476 | 4 | 120.82 | Trichoptera | 1.108 | 6.851 | 13.20195 |
| JFBM48476 | 4 | 120.82 | Bivalva | 1.4 | 2.93 | 6.46306 |
| JFBM48476 | 4 | 120.82 | Bivalva | 2.006 | 2.629 | 14.07425 |
| JFBM48476 | 5 | 104.02 | Insecta larva aquataic | 1.92 | 8.69 | 46.55793 |
| JFBM48476 | 5 | 104.02 | Insecta larva aquataic | 1.318 |  | 8.22166 |
| JFBM48476 | 5 | 104.02 | Coleoptera terrestrial | 2.291 | 5.84 | 32.29017 |
| JFBM48535 | 1 | 35.66 | Diptera larva | 0.063 |  | 0.18037 |
| JFBM48535 | 1 | 35.66 | Diptera larva | 0.137 |  | 0.20392 |
| JFBM48535 | 1 | 35.66 | Ostracoda | 0.163 |  | 0.00065 |
| JFBM48535 | 1 | 35.66 | Ostracoda | 0.165 |  | 0.00076 |
| JFBM48535 | 1 | 35.66 | Diptera larva | 0.194 |  | 0.22193 |
| JFBM48535 | 1 | 35.66 | Ostracoda | 0.195 |  | 0.00211 |
| JFBM48535 | 1 | 35.66 | Trichoptera larva | 0.209 |  | 0.22669 |
| JFBM48535 | 1 | 35.66 | Daphnia | 0.214 |  | 0.00542 |
| JFBM48535 | 2 | 35.39 | Ostracoda | 0.035 |  | 0.00086 |
| JFBM48535 | 2 | 35.39 | Insecta terrestrial Diptera | 0.053 |  | 0.00104 |
| JFBM48535 | 2 | 35.39 | Insecta terrestrial Diptera | 0.054 |  | 0.00104 |
| JFBM48535 | 2 | 35.39 | Insecta terrestrial Trichoptera | 0.102 |  | 0.00172 |
| JFBM48535 | 2 | 35.39 | Daphnia | 0.168 |  | 0.0009 |
| JFBM48535 | 2 | 35.39 | Copepoda | 0.169 |  | 0.00343 |
| JFBM48535 | 2 | 35.39 | Ostracoda | 0.195 |  | 0.0021 |
| JFBM48535 | 3 | 37.94 | Copepoda | 0.263 | 0.64 | 0.00499 |
| JFBM48535 | 3 | 37.94 | Copepoda | 0.273 |  | 0.24692 |
| JFBM48535 | 3 | 37.94 | Copepoda | 0.263 |  | 0.24393 |
| JFBM48535 | 3 | 37.94 | Cladocera | 0.236 |  | 0.23527 |
| JFBM48535 | 3 | 37.94 | Copepoda | 0.258 | 0.544 | 0.00373 |
| JFBM48535 | 3 | 37.94 | Insecta | 0.305 |  | 0.25728 |
| JFBM48535 | 3 | 37.94 | Copepoda | 0.413 | 0.605 | 0.01191 |
| JFBM48535 | 3 | 37.94 | Copepoda | 0.425 | 0.855 | 0.01275 |
| JFBM48535 | 3 | 37.94 | Copepoda | 0.463 |  | 0.30744 |
| JFBM48535 | 3 | 37.94 | Copepoda | 0.276 | 0.695 | 0.0079 |
| JFBM48535 | 3 | 37.94 | Copepoda | 0.324 |  | 0.26328 |
| JFBM48535 | 3 | 37.94 | Copepoda | 0.427 | 0.818 | 0.01687 |
| JFBM48535 | 3 | 37.94 | Copepoda | 0.259 | 0.493 | 0.00495 |
| JFBM48535 | 3 | 37.94 | Copepoda | 0.295 |  | 0.2541 |
| JFBM48535 | 3 | 37.94 | Copepoda | 0.243 | 0.508 | 0.00291 |
| JFBM48535 | 3 | 37.94 | Copepoda | 0.278 | 0.631 | 0.00657 |
| JFBM48535 | 3 | 37.94 | Copepoda | 0.29 |  | 0.25244 |
| JFBM48535 | 3 | 37.94 | Copepoda | 0.317 | 0.664 | 0.01293 |
| JFBM48535 | 3 | 37.94 | Copepoda | 0.256 | 0.535 | 0.00454 |
| JFBM48535 | 3 | 37.94 | Copepoda | 0.282 |  | 0.24984 |
| JFBM48535 | 3 | 37.94 | Copepoda | 0.206 |  | 0.22584 |
| JFBM48535 | 3 | 37.94 | Copepoda | 0.341 | 0.736 | 0.01236 |
| JFBM48535 | 3 | 37.94 | Copepoda | 0.263 | 0.601 | 0.00667 |
| JFBM48535 | 3 | 37.94 | Copepoda | 0.263 | 0.525 | 0.00434 |
| JFBM48535 | 3 | 37.94 | Copepoda | 0.285 |  | 0.25085 |
| JFBM48535 | 3 | 37.94 | Diptera larva aquatic | 1.282 | 5.644 | 0.19268 |
| JFBM48535 | 3 | 37.94 | Diptera larva aquatic | 0.182 | 4.747 | 0.03916 |
| JFBM48535 | 3 | 37.94 | Diptera larva aquatic | 0.152 |  | 0.20872 |
| JFBM48535 | 3 | 37.94 | Diptera larva aquatic | 0.174 | 1.606 | 0.00293 |
| JFBM48535 | 3 | 37.94 | Copepoda | 0.28 | 0.713 | 0.00815 |
| JFBM48535 | 3 | 37.94 | Diptera larva aquatic | 0.306 | 3.765 | 0.02121 |
| JFBM48535 | 3 | 37.94 | Diptera larva aquatic | 0.345 |  | 0.26995 |
| JFBM48535 | 3 | 37.94 | Diptera larva aquatic | 0.237 | 3.583 | 0.03286 |
| JFBM48535 | 3 | 37.94 | Diptera larva aquatic | 0.225 | 2.113 | 0.01174 |
| JFBM48535 | 3 | 37.94 | Diptera pupa aquatic | 0.259 | 1.161 | 0.00867 |
| JFBM48535 | 3 | 37.94 | Diptera larva aquatic | 0.257 |  | 0.24195 |
| JFBM48535 | 3 | 37.94 | Diptera larva aquatic | 0.255 | 3 | 0.02864 |
| JFBM48535 | 3 | 37.94 | Ostracoda | 0.405 | 0.534 | 0.00954 |
| JFBM48535 | 3 | 37.94 | Copepoda | 0.243 | 0.591 | 0.00518 |
| JFBM48535 | 3 | 37.94 | Insecta terrestrial | 0.424 |  | 0.29498 |
| JFBM48535 | 3 | 37.94 | Insecta terrestrial | 0.35 |  | 0.27145 |
| JFBM48535 | 3 | 37.94 | Insecta terrestrial | 0.323 |  | 0.26295 |
| JFBM48535 | 3 | 37.94 | Insecta terrestrial | 0.298 |  | 0.25505 |
| JFBM48535 | 3 | 37.94 | Copepoda | 0.255 |  | 0.24146 |
| JFBM48535 | 3 | 37.94 | Insecta terrestrial | 0.324 |  | 0.26316 |
| JFBM48535 | 3 | 37.94 | Insecta terrestrial | 0.436 |  | 0.2989 |
| JFBM48535 | 3 | 37.94 | Insecta terrestrial | 0.346 |  | 0.27036 |
| JFBM48535 | 3 | 37.94 | Copepoda | 0.237 |  | 0.23568 |
| JFBM48535 | 3 | 37.94 | Insecta terrestrial | 0.223 |  | 0.23132 |
| JFBM48535 | 3 | 37.94 | Insecta terrestrial | 0.143 | 1.519 | 0.00305 |
| JFBM48535 | 3 | 37.94 | Ostracoda | 0.393 | 0.533 | 0.01014 |
| JFBM48535 | 3 | 37.94 | Ostracoda | 0.271 | 0.63 | 0.00757 |
| JFBM48535 | 3 | 37.94 | Insecta terrestrial | 0.35 |  | 0.27141 |
| JFBM48535 | 3 | 37.94 | Ostracoda | 0.407 | 0.605 | 0.01268 |
| JFBM48535 | 3 | 37.94 | Copepoda | 0.359 |  | 0.27442 |
| JFBM48535 | 3 | 37.94 | Copepoda | 0.41 | 0.723 | 0.02321 |
| JFBM48535 | 3 | 37.94 | Ostracoda | 0.46 | 0.556 | 0.01327 |
| JFBM48535 | 3 | 37.94 | Ostracoda | 0.506 | 0.664 | 0.02087 |
| JFBM48535 | 3 | 37.94 | Ostracoda | 0.417 | 0.534 | 0.01136 |
| JFBM48535 | 3 | 37.94 | Insecta larva aquataic | 0.26 |  | 0.24286 |
| JFBM48535 | 3 | 37.94 | Insecta larva aquataic | 0.119 | 1.314 | 0.00122 |
| JFBM48535 | 6 | 43.66 | Diptera terrestrial | 0.438 | 0.605 | 0.01079 |
| JFBM48535 | 6 | 43.66 | Diptera larva aquatic | 0.368 | 4.987 | 0.06726 |
| JFBM48535 | 6 | 43.66 | Diptera larva aquatic | 0.345 |  | 0.08146 |
| JFBM48535 | 6 | 43.66 | Diptera larva aquatic | 0.293 |  | 0.25344 |
| JFBM48535 | 6 | 43.66 | Diptera larva aquatic | 0.266 | 2.743 | 0.02611 |
| JFBM48535 | 6 | 43.66 | Diptera larva aquatic | 0.24 |  | 0.23658 |
| JFBM48535 | 6 | 43.66 | Diptera larva aquatic | 0.287 |  | 0.25141 |
| JFBM48535 | 6 | 43.66 | Diptera larva aquatic | 0.287 |  | 0.25153 |
| JFBM48535 | 6 | 43.66 | Diptera larva aquatic | 0.28 |  | 0.24921 |
| JFBM48535 | 6 | 43.66 | Diptera larva aquatic | 0.311 |  | 0.25918 |
| JFBM48535 | 6 | 43.66 | Diptera larva aquatic | 0.215 |  | 0.22864 |
| JFBM48535 | 6 | 43.66 | Diptera larva aquatic | 0.214 |  | 0.22827 |
| JFBM48535 | 6 | 43.66 | Diptera larva aquatic | 0.097 |  | 0.19125 |
| JFBM48535 | 6 | 43.66 | Diptera larva aquatic | 0.202 |  | 0.22437 |
| JFBM48535 | 6 | 43.66 | Diptera larva aquatic | 0.337 |  | 0.26742 |
| JFBM48535 | 6 | 43.66 | Seed | 0.523 | 0.735 | 0.02253 |
| JFBM48535 | 6 | 43.66 | Seed | 0.528 | 0.733 | 0.0338 |
| JFBM48535 | 6 | 43.66 | Insecta larva aquatic | 0.304 |  | 0.2568 |
| JFBM48535 | 6 | 43.66 | Insecta larva aquatic | 0.411 |  | 0.62945 |
| JFBM48535 | 8 | 79.09 | Diptera larva aquatic | 0.511 |  | 1.46913 |
| JFBM48535 | 8 | 79.09 | Trichoptera larva aquatic | 1.206 |  | 7.28422 |
| JFBM48535 | 8 | 79.09 | Trichoptera larva aquatic | 1.044 | 7.728 | 7.25115 |
| JFBM48535 | 8 | 79.09 | Insecta larva aquatic | 1.016 |  | 5.69392 |
| JFBM48535 | 8 | 79.09 | Insecta larva aquatic | 1.286 |  | 7.95382 |
| JFBM48535 | 8 | 79.09 | Trichoptera larva aquatic | 1.636 | 8.477 | 25.52805 |
| JFBM48535 | 8 | 79.09 | Trichoptera larva aquatic | 1.493 | 8.993 | 17.77297 |
| JFBM48537 | 1 | 146.99 | Insecta larvae aquatic | 1.119 |  | 6.55603 |
| JFBM48537 | 3 | 103.63 | Gerridae | 1.237 |  | 0.04868 |
| JFBM48537 | 4 | 83.15 | Araneae terrestrial | 0.627 |  | 0.02141 |
| JFBM48537 | 4 | 83.15 | Diptera pupae aquatic | 0.846 |  | 4.27102 |
| JFBM48537 | 4 | 83.15 | Diptera pupae aquatic | 0.736 |  | 3.35032 |
| JFBM48537 | 5 | 79.49 | Araneae | 0.707 |  | 3.10759 |
| JFBM48554 | 1 | 72.23 | Copepoda | 0.514 | 0.653 | 0.03037 |
| JFBM48554 | 1 | 72.23 | Copepoda | 0.317 | 0.576 | 0.0069 |
| JFBM48554 | 1 | 72.23 | Copepoda | 0.457 |  | 0.06714 |
| JFBM48554 | 1 | 72.23 | Copepoda | 0.409 | 0.748 | 0.02275 |
| JFBM48554 | 1 | 72.23 | Copepoda | 0.308 | 0.758 | 0.00838 |
| JFBM48554 | 1 | 72.23 | Copepoda | 0.413 | 0.874 | 0.02549 |
| JFBM48554 | 1 | 72.23 | Copepoda | 0.338 | 0.886 | 0.01629 |
| JFBM48554 | 1 | 72.23 | Copepoda | 0.371 |  | 0.02748 |
| JFBM48554 | 1 | 72.23 | Copepoda | 0.419 | 0.701 | 0.01943 |
| JFBM48554 | 1 | 72.23 | Copepoda | 0.397 | 0.723 | 0.01533 |
| JFBM48554 | 1 | 72.23 | Copepoda | 0.367 | 0.703 | 0.02019 |
| JFBM48554 | 1 | 72.23 | Copepoda | 0.359 | 0.846 | 0.01757 |
| JFBM48554 | 1 | 72.23 | Copepoda | 0.276 |  | 0.01032 |
| JFBM48554 | 1 | 72.23 | Copepoda | 0.389 | 0.866 | 0.02288 |
| JFBM48554 | 1 | 72.23 | Copepoda | 0.25 |  | 0.00791 |
| JFBM48554 | 1 | 72.23 | Copepoda | 0.445 | 0.971 | 0.01715 |
| JFBM48554 | 1 | 72.23 | Copepoda | 0.455 | 0.933 | 0.01934 |
| JFBM48554 | 1 | 72.23 | Copepoda | 0.446 | 0.904 | 0.01841 |
| JFBM48554 | 1 | 72.23 | Copepoda | 0.455 |  | 0.06536 |
| JFBM48554 | 1 | 72.23 | Copepoda | 0.484 | 0.728 | 0.01881 |
| JFBM48554 | 1 | 72.23 | Copepoda | 0.154 | 0.508 | 0.00158 |
| JFBM48554 | 1 | 72.23 | Copepoda | 0.341 |  | 0.02012 |
| JFBM48554 | 1 | 72.23 | Copepoda | 0.407 | 0.87 | 0.01985 |
| JFBM48554 | 1 | 72.23 | Copepoda | 0.329 | 0.655 | 0.00963 |
| JFBM48554 | 1 | 72.23 | Copepoda | 0.254 | 0.717 | 0.00898 |
| JFBM48554 | 1 | 72.23 | Copepoda | 0.252 |  | 0.00807 |
| JFBM48554 | 1 | 72.23 | Copepoda | 0.314 |  | 0.01535 |
| JFBM48554 | 1 | 72.23 | Copepoda | 0.448 | 0.883 | 0.03048 |
| JFBM48554 | 1 | 72.23 | Copepoda | 0.328 |  | 0.01759 |
| JFBM48554 | 1 | 72.23 | Diptera larva aquatic | 0.614 | 4.701 | 0.18602 |
| JFBM48554 | 1 | 72.23 | Diptera larva aquatic | 0.668 | 4.357 | 0.20817 |
| JFBM48554 | 1 | 72.23 | Insecta terrestrial | 0.8 | 1.957 | 0.17083 |
| JFBM48554 | 3 | 57.29 | Insecta larvae aquatic | 1.054 | 5.161 | 17.97045 |
| JFBM48554 | 3 | 57.29 | Copepoda | 0.307 | 0.671 | 0.05792 |
| JFBM48554 | 4 | 55.17 | Copepoda | 0.282 | 0.75 | 0.01618 |
| JFBM48554 | 4 | 55.17 | Copepoda | 0.376 | 0.823 | 0.01742 |
| JFBM48554 | 4 | 55.17 | Copepoda | 0.42 | 0.757 | 0.01737 |
| JFBM48554 | 4 | 55.17 | Copepoda | 0.263 | 0.63 | 0.0083 |
| JFBM48554 | 4 | 55.17 | Copepoda | 0.375 | 0.677 | 0.00966 |
| JFBM48554 | 4 | 55.17 | Insecta terrestrial | 1.28 |  | 7.9036 |
| JFBM48554 | 4 | 55.17 | Insecta terrestrial | 1.386 |  | 8.79082 |
| JFBM48554 | 4 | 55.17 | Insecta terrestrial | 1.498 |  | 9.72826 |
| JFBM48554 | 4 | 55.17 | Insecta terrestrial | 1.563 |  | 10.27231 |
| JFBM48554 | 4 | 55.17 | Insecta terrestrial | 1.476 |  | 9.54412 |
| JFBM48554 | 4 | 55.17 | Insecta terrestrial | 1.736 |  | 11.72032 |
| JFBM48554 | 5 | 71.98 | Copepoda | 0.373 | 0.762 | 0.01946 |
| JFBM48554 | 5 | 71.98 | Copepoda | 0.308 | 0.305 | 0.0732 |
| JFBM48554 | 5 | 71.98 | Copepoda | 0.356 | 0.828 | 0.01177 |
| JFBM48554 | 5 | 71.98 | Copepoda | 0.298 | 0.736 | 0.00892 |
| JFBM48554 | 5 | 71.98 | Copepoda | 0.345 | 0.797 | 0.01225 |
| JFBM48554 | 5 | 71.98 | Copepoda | 0.345 | 0.778 | 0.0203 |
| JFBM48554 | 5 | 71.98 | Copepoda | 0.318 | 0.807 | 0.01196 |
| JFBM48554 | 5 | 71.98 | Copepoda | 0.213 | 0.5 | 0.00579 |
| JFBM48554 | 5 | 71.98 | Copepoda | 0.402 | 0.894 | 0.0185 |
| JFBM48554 | 5 | 71.98 | Diptera larvae aquatic | 1.064 |  | 6.09927 |
| JFBM48554 | 5 | 71.98 | Diptera larvae aquatic | 0.733 |  | 3.32618 |
| JFBM48554 | 5 | 71.98 | Diptera larvae aquatic | 0.663 |  | 2.73674 |
| JFBM48554 | 5 | 71.98 | Ostracoda | 0.378 |  | 0.35358 |
| JFBM48554 | 7 | 134.01 | Aranaea | 0.725 | 1.17 | 3.25825 |
| JFBM48554 | 7 | 134.01 | Trichoptera larvae aquatic | 0.199 |  | 0.2234 |
| JFBM48554 | 7 | 134.01 | Trichoptera larvae aquatic | 1.249 | 7.537 | 7.64413 |
| JFBM48562 | 1 | 167.71 | Fish unidentifiable | 11.982 |  | 8870.244 |
| JFBM48563 | 1 | 147.64 | Insecta Trichoptera terrestrial | 1.411 |  | 2.78696 |
| JFBM48563 | 1 | 147.64 | Diptera pupae aquatic | 1.739 |  | 11.74543 |
| JFBM48563 | 2 | 150.47 | Diptera larvae aquatic | 0.993 |  | 5.50141 |
| JFBM48563 | 2 | 150.47 | Diptera larvae aquatic | 0.458 |  | 1.02346 |
| JFBM48563 | 5 | 142.92 | Diptera pupae aquatic | 0.756 |  | 3.51772 |
| JFBM48563 | 5 | 142.92 | Diptera larvae aquatic | 0.876 |  | 4.52212 |
| JFBM48563 | 5 | 142.92 | Diptera pupae aquatic | 0.979 |  | 5.38423 |
| JFBM48565 | 2 | 63.83 | Diptera larvae aquatic | 0.825 |  | 4.09525 |
| JFBM48565 | 4 | 111.52 | Insecta Trichoptera larvae aquatic | 1.716 | 9.699 | 36.42879 |
| JFBM48565 | 4 | 111.52 | Insecta Trichoptera larvae aquatic | 1.281 | 5.956 | 10.45002 |
| JFBM48565 | 5 | 113.58 | Fish unidentifiable | 4.181 | 18.333 | 268.83199 |
| JFBM48568 | 1 | 140.69 | Diptera larvae aquatic | 0.452 |  | 0.97324 |
| JFBM48568 | 1 | 140.69 | Insecta Trichoptera terrestrial | 0.854 |  | 1.44615 |
| JFBM48568 | 1 | 140.69 | Coleoptera | 2.719 |  | 5.93558 |
| JFBM48568 | 2 | 143.99 | Diptera larvae aquatic | 1.548 | 10.289 | 21.91481 |
| JFBM48568 | 2 | 143.99 | Diptera larvae aquatic | 1.741 | 11.84 | 55.07213 |
| JFBM48568 | 3 | 115.38 | Trichoptera larvae aquatic | 0.421 |  | 0.71377 |
| JFBM48568 | 3 | 115.38 | Trichoptera larvae aquatic | 0.525 | 2.886 | 0.60556 |
| JFBM48568 | 3 | 115.38 | Trichoptera larvae aquatic | 1.01 | 5.056 | 11.79875 |
| JFBM48568 | 3 | 115.38 | Trichoptera larvae aquatic | 1.491 | 6.176 | 10.48118 |
| JFBM48568 | 3 | 115.38 | Trichoptera larvae aquatic | 0.882 | 4.675 | 2.1924 |
| JFBM48568 | 3 | 115.38 | Trichoptera larvae aquatic | 1.125 |  | 6.60625 |
| JFBM48568 | 3 | 115.38 | Trichoptera larvae aquatic | 1.173 |  | 7.00801 |
| JFBM48568 | 3 | 115.38 | Trichoptera larvae aquatic | 1.144 |  | 6.76528 |
| JFBM48568 | 3 | 115.38 | Trichoptera larvae aquatic | 0.895 |  | 4.68115 |
| JFBM48568 | 3 | 115.38 | Trichoptera larvae aquatic | 1.081 |  | 6.23797 |
| JFBM48568 | 3 | 115.38 | Trichoptera larvae aquatic | 0.649 |  | 2.62213 |
| JFBM48568 | 3 | 115.38 | Trichoptera larvae aquatic | 0.907 |  | 4.78159 |
| JFBM48568 | 3 | 115.38 | Trichoptera larvae aquatic | 1.065 |  | 6.10405 |
| JFBM48568 | 3 | 115.38 | Trichoptera larvae aquatic | 0.806 |  | 3.93622 |
| JFBM48568 | 3 | 115.38 | Trichoptera larvae aquatic | 0.678 |  | 2.86486 |
| JFBM48568 | 3 | 115.38 | Trichoptera larvae aquatic | 1.109 |  | 6.47233 |
| JFBM48568 | 3 | 115.38 | Trichoptera larvae aquatic | 1.072 |  | 6.16264 |
| JFBM48568 | 3 | 115.38 | Trichoptera larvae aquatic | 0.841 |  | 4.22917 |
| JFBM48568 | 4 | 93.34 | Diptera pupae aquatic | 1.865 | 9.432 | 21.09111 |
| JFBM48568 | 4 | 93.34 | Diptera pupae aquatic | 1.946 | 10.703 | 22.65784 |
| JFBM48568 | 4 | 93.34 | Diptera pupae aquatic | 1.378 |  | 8.72386 |
| JFBM48568 | 4 | 93.34 | Insecta larvae aquatic | 0.332 | 2.632 | 0.58126 |
| JFBM48568 | 4 | 93.34 | Insecta larvae aquatic | 0.431 | 4.371 | 1.20725 |
| JFBM48568 | 4 | 93.34 | Trichoptera larvae aquatic | 1.622 | 9.948 | 22.26593 |
| JFBM48568 | 4 | 93.34 | Trichoptera larvae aquatic | 1.378 | 7.297 | 29.59202 |
| JFBM48568 | 4 | 93.34 | Trichoptera larvae aquatic | 1.433 | 7.077 | 8.16452 |
| JFBM48568 | 4 | 93.34 | Trichoptera larvae aquatic | 1.129 |  | 6.63973 |
| JFBM48568 | 4 | 93.34 | Trichoptera larvae aquatic | 1.189 | 9.634 | 25.76556 |
| JFBM48568 | 4 | 93.34 | Trichoptera larvae aquatic | 1.221 |  | 7.40977 |
| JFBM48568 | 4 | 93.34 | Trichoptera larvae aquatic | 1.297 | 7.079 | 14.46092 |
| JFBM48568 | 4 | 93.34 | Trichoptera larvae aquatic | 1.136 | 7.484 | 5.65508 |
| JFBM48568 | 4 | 93.34 | Trichoptera larvae aquatic | 1.147 | 6.223 | 7.68394 |
| JFBM48568 | 4 | 93.34 | Trichoptera larvae aquatic | 1.06 | 6.178 | 9.88904 |
| JFBM48568 | 4 | 93.34 | Trichoptera larvae aquatic | 0.911 | 6.131 | 5.35134 |
| JFBM48568 | 4 | 93.34 | Trichoptera larvae aquatic | 1.088 | 5.38 | 4.90936 |
| JFBM48568 | 4 | 93.34 | Trichoptera larvae aquatic | 0.823 | 5.366 | 5.74186 |
| JFBM48568 | 4 | 93.34 | Trichoptera larvae aquatic | 1.029 | 5.263 | 3.53233 |
| JFBM48568 | 4 | 93.34 | Trichoptera larvae aquatic | 1.236 | 6.238 | 6.91287 |
| JFBM48568 | 4 | 93.34 | Trichoptera larvae aquatic | 1.223 | 6.564 | 7.23082 |
| JFBM48568 | 4 | 93.34 | Trichoptera larvae aquatic | 1.148 | 6.056 | 8.1787 |
| JFBM48568 | 4 | 93.34 | Trichoptera larvae aquatic | 0.995 | 5.226 | 4.20469 |
| JFBM48568 | 4 | 93.34 | Trichoptera larvae aquatic | 1.294 | 8.179 | 17.32592 |
| JFBM48568 | 4 | 93.34 | Trichoptera larvae aquatic | 1.377 | 5.127 | 5.05579 |
| JFBM48568 | 4 | 93.34 | Trichoptera larvae aquatic | 1.012 | 5.516 | 9.21371 |
| JFBM48568 | 4 | 93.34 | Trichoptera larvae aquatic | 1.318 | 6.426 | 8.42511 |
| JFBM48568 | 4 | 93.34 | Trichoptera larvae aquatic | 0.936 | 4.867 | 2.48503 |
| JFBM48568 | 4 | 93.34 | Trichoptera larvae aquatic | 1.197 | 4.166 | 7.24998 |
| JFBM48568 | 4 | 93.34 | Trichoptera larvae aquatic | 0.793 | 4.307 | 7.96141 |
| JFBM48568 | 4 | 93.34 | Trichoptera larvae aquatic | 0.725 | 3.127 | 3.8668 |
| JFBM48568 | 4 | 93.34 | Trichoptera larvae aquatic | 0.749 | 4.598 | 5.22897 |
| JFBM48568 | 5 | 102.94 | Diptera larvae aquatic | 1.794 |  | 12.20578 |
| JFBM48568 | 5 | 102.94 | Diptera larvae aquatic | 0.422 |  | 0.72214 |
| JFBM48568 | 5 | 102.94 | Trichoptera larvae aquatic | 1.068 |  | 6.12916 |
| JFBM48568 | 5 | 102.94 | Trichoptera larvae aquatic | 1.437 |  | 9.21769 |
| JFBM48568 | 5 | 102.94 | Trichoptera larvae aquatic | 0.653 |  | 2.65561 |
| JFBM48568 | 5 | 102.94 | Trichoptera larvae aquatic | 1.055 |  | 6.02035 |
| JFBM48568 | 5 | 102.94 | Trichoptera larvae aquatic | 1.355 |  | 8.53135 |
| JFBM48568 | 5 | 102.94 | Trichoptera larvae aquatic | 1.129 |  | 6.63973 |
| JFBM48568 | 5 | 102.94 | Trichoptera larvae aquatic | 0.767 |  | 3.60979 |
| JFBM48568 | 5 | 102.94 | Trichoptera larvae aquatic | 0.733 |  | 3.32521 |
| JFBM48568 | 5 | 102.94 | Trichoptera larvae aquatic | 0.712 |  | 3.14944 |
| JFBM48568 | 5 | 102.94 | Trichoptera larvae aquatic | 1.597 |  | 10.55689 |
| JFBM48568 | 5 | 102.94 | Trichoptera larvae aquatic | 1.254 |  | 7.68598 |
| JFBM48568 | 5 | 102.94 | Trichoptera larvae aquatic | 0.756 |  | 3.51772 |
| JFBM48568 | 5 | 102.94 | Trichoptera larvae aquatic | 0.885 |  | 4.59745 |
| JFBM48568 | 5 | 102.94 | Trichoptera larvae aquatic | 1.044 |  | 5.92828 |
| JFBM48568 | 5 | 102.94 | Trichoptera larvae aquatic | 0.716 |  | 3.18292 |
| JFBM48568 | 5 | 102.94 | Trichoptera larvae aquatic | 0.914 |  | 4.84018 |
| JFBM48568 | 5 | 102.94 | Trichoptera larvae aquatic | 0.911 |  | 4.81507 |
| JFBM48568 | 5 | 102.94 | Trichoptera larvae aquatic | 0.912 |  | 4.82344 |
| JFBM48568 | 5 | 102.94 | Trichoptera larvae aquatic | 1.006 |  | 5.61022 |
| JFBM48568 | 5 | 102.94 | Trichoptera larvae aquatic | 0.61 |  | 2.2957 |
| JFBM48568 | 5 | 102.94 | Trichoptera larvae aquatic | 1.793 |  | 12.19741 |
| JFBM48568 | 5 | 102.94 | Trichoptera larvae aquatic | 1.355 |  | 8.53135 |
| JFBM48568 | 5 | 102.94 | Trichoptera larvae aquatic | 1.348 |  | 8.47276 |
| JFBM48568 | 5 | 102.94 | Trichoptera larvae aquatic | 0.761 |  | 3.55957 |
| JFBM48568 | 5 | 102.94 | Trichoptera larvae aquatic | 0.879 |  | 4.54723 |
| JFBM48568 | 5 | 102.94 | Trichoptera larvae aquatic | 1.271 |  | 7.82827 |
| JFBM48568 | 5 | 102.94 | Trichoptera larvae aquatic | 0.844 |  | 4.25428 |
| JFBM48568 | 5 | 102.94 | Trichoptera larvae aquatic | 0.926 |  | 4.94062 |
| JFBM48568 | 5 | 102.94 | Trichoptera larvae aquatic | 0.953 |  | 5.16661 |
| JFBM48568 | 5 | 102.94 | Trichoptera larvae aquatic | 0.997 |  | 5.53489 |
| JFBM48568 | 5 | 102.94 | Trichoptera larvae aquatic | 1.612 |  | 10.68244 |
| JFBM48568 | 5 | 102.94 | Trichoptera larvae aquatic | 0.869 |  | 4.46353 |
| JFBM48568 | 5 | 102.94 | Trichoptera larvae aquatic | 1.163 |  | 6.92431 |
| JFBM48568 | 5 | 102.94 | Trichoptera larvae aquatic | 0.745 |  | 3.42565 |
| JFBM48568 | 5 | 102.94 | Trichoptera larvae aquatic | 0.848 |  | 4.28776 |
| JFBM48568 | 5 | 102.94 | Trichoptera larvae aquatic | 0.772 |  | 3.65164 |
| JFBM48568 | 5 | 102.94 | Trichoptera larvae aquatic | 0.99 |  | 5.4763 |
| JFBM48568 | 5 | 102.94 | Trichoptera larvae aquatic | 0.938 |  | 5.04106 |
| JFBM48570 | 3 | 74.98 | Diptera larvae aquatic | 0.353 |  | 0.14554 |
| JFBM48570 | 3 | 74.98 | Diptera larvae aquatic | 0.872 |  | 4.49261 |
| JFBM48570 | 3 | 74.98 | Insecta terrestrial | 0.614 |  | 0.86911 |
| JFBM48570 | 3 | 74.98 | Insecta terrestrial | 0.362 |  | 0.26135 |
| JFBM48570 | 4 | 87.92 | Diptera larvae aquatic | 0.406 |  | 0.58952 |
| JFBM48570 | 4 | 87.92 | Diptera larvae aquatic | 0.354 |  | 0.15071 |
| JFBM48570 | 4 | 87.92 | Ostracoda | 0.399 | 0.824 | 0.02092 |
| JFBM48570 | 4 | 87.92 | Trichoptera larvae aquatic | 0.504 |  | 1.40445 |
| JFBM48570 | 5 | 92.11 | Diptera larvae aquatic | 0.276 |  | 0.01027 |
| JFBM48570 | 7 | 85.78 | Diptera larvae aquatic | 0.22 | 1.926 | 0.00818 |
| JFBM48570 | 7 | 85.78 | Diptera larvae aquatic | 0.339 | 3.092 | 0.02661 |
| JFBM48570 | 7 | 85.78 | Diptera larvae aquatic | 0.371 |  | 0.29158 |
| JFBM48570 | 7 | 85.78 | Diptera larvae aquatic | 0.265 |  | 0.00921 |
| JFBM48570 | 7 | 85.78 | Diptera larvae aquatic | 0.607 |  | 2.26653 |
| JFBM48570 | 7 | 85.78 | Diptera larvae aquatic | 0.251 |  | 0.23993 |
| JFBM48570 | 8 | 96.61 | Diptera larvae aquatic | 0.489 |  | 1.28059 |
| JFBM48570 | 8 | 96.61 | Ostracoda | 0.316 | 0.551 | 0.01086 |
| JFBM48570 | 8 | 96.61 | Diptera larvae aquatic | 0.509 | 3.744 | 0.07954 |
| JFBM48570 | 8 | 96.61 | Trichoptera larvae aquatic | 0.604 |  | 2.24567 |
| JFBM48570 | 8 | 96.61 | Diptera larvae aquatic | 0.263 | 1.256 | 0.01404 |
| JFBM48570 | 8 | 96.61 | Diptera larvae aquatic | 0.253 |  | 0.24075 |
| JFBM48570 | 8 | 96.61 | Diptera larvae aquatic | 0.301 | 2.337 | 0.02388 |
| JFBM48570 | 8 | 96.61 | Trichoptera larvae aquatic | 0.827 |  | 4.11365 |
| JFBM48570 | 8 | 96.61 | Trichoptera larvae aquatic | 0.604 |  | 2.24567 |
| JFBM48570 | 8 | 96.61 | Coleoptera | 0.476 |  | 0.53649 |
| JFBM48570 | 8 | 96.61 | Trichoptera larvae aquatic | 0.305 | 2.491 | 0.02406 |
| JFBM48570 | 9 | 91.21 | Trichoptera larvae aquatic | 0.68 |  | 2.88319 |
| JFBM48570 | 9 | 91.21 | Trichoptera larvae aquatic | 0.772 | 3.709 | 0.10642 |
| JFBM48570 | 9 | 91.21 | Trichoptera larvae aquatic | 0.471 | 3.527 | 0.06175 |
| JFBM48570 | 9 | 91.21 | Trichoptera larvae aquatic | 0.433 | 3.433 | 0.02141 |
| JFBM48570 | 10 | 141.62 | Ephemeroptera aquatic | 2.893 |  | 21.40441 |
| JFBM48570 | 11 | 119.66 | Diptera larvae aquatic | 3.893 | 9.081 | 26.0958 |
| JFBM48570 | 11 | 119.66 | Diptera larvae aquatic | 0.783 | 4.122 | 1.36923 |
| JFBM48570 | 13 | 100.37 | Ostracoda | 0.208 | 0.208 | 0.02641 |
| JFBM48570 | 13 | 100.37 | Trichoptera larvae aquatic | 0.453 |  | 0.98394 |
| JFBM48570 | 13 | 100.37 | Trichoptera larvae aquatic | 0.294 |  | 0.25365 |
| JFBM48584 | 1 | 67.05 | Diptera larvae aquatic | 0.485 |  | 1.24769 |
| JFBM48584 | 1 | 67.05 | Ostracoda | 0.335 | 0.494 | 0.00868 |
| JFBM48584 | 1 | 67.05 | Ostracoda | 0.343 | 0.477 | 0.00994 |
| JFBM48584 | 1 | 67.05 | Ostracoda | 0.4 | 0.556 | 0.01185 |
| JFBM48584 | 1 | 67.05 | Ostracoda | 0.323 | 0.473 | 0.00824 |
| JFBM48584 | 1 | 67.05 | Ostracoda | 0.355 | 0.533 | 0.01095 |
| JFBM48584 | 2 | 78.5 | Ostracoda | 0.192 | 0.361 | 0.00211 |
| JFBM48584 | 2 | 78.5 | Trichoptera larvae aquatic | 0.687 |  | 2.94255 |
| JFBM48584 | 2 | 78.5 | Trichoptera larvae aquatic | 0.579 |  | 2.03519 |
| JFBM48584 | 2 | 78.5 | Trichoptera larvae aquatic | 0.556 |  | 1.84766 |
| JFBM48584 | 3 | 86.29 | Copepoda | 0.333 |  | 0.01857 |
| JFBM48584 | 3 | 86.29 | Copepoda Calanoida | 0.304 | 0.627 | 0.0091 |
| JFBM48584 | 3 | 86.29 | Copepoda Calanoida | 0.367 | 0.851 | 0.01755 |
| JFBM48584 | 3 | 86.29 | Egg | 0.226 | 0.449 | 0.00273 |
| JFBM48584 | 3 | 86.29 | Ostracoda | 0.297 | 0.499 | 0.00862 |
| JFBM48584 | 3 | 86.29 | Ostracoda | 0.387 | 0.526 | 0.01228 |
| JFBM48584 | 4 | 73.53 | Ostracoda | 0.291 | 0.558 | 0.01073 |
| JFBM48584 | 4 | 73.53 | Ostracoda | 0.296 | 0.455 | 0.00604 |
| JFBM48585 | 2 | 147.74 | Fish Cyprinidae | 4.316 | 21.911 | 382.24927 |
| JFBM48585 | 4 | 93.33 | Araneae terrestrial | 1.522 | 4.966 | 8.63189 |
| JFBM48585 | 4 | 93.33 | Coleoptera terrestrial | 0.952 | 2.183 | 1.51963 |
| JFBM48585 | 4 | 93.33 | Coleoptera terrestrial | 0.911 | 2.034 | 1.44901 |
| JFBM48585 | 4 | 93.33 | Hymenoptera terrestrial | 0.488 | 1.679 | 0.3781 |
| JFBM48585 | 4 | 93.33 | Hymenoptera terrestrial | 0.81 | 2.568 | 1.60519 |
| JFBM48585 | 4 | 93.33 | Hymenoptera terrestrial | 1.083 |  | 1.9974 |
| JFBM48585 | 4 | 93.33 | Hymenoptera terrestrial | 1.019 | 4.036 | 2.43162 |
| JFBM48585 | 4 | 93.33 | Hymenoptera terrestrial | 1.118 |  | 2.08165 |
| JFBM48585 | 4 | 93.33 | Hymenoptera terrestrial | 1.237 |  | 2.36811 |
| JFBM48585 | 4 | 93.33 | Hymenoptera terrestrial | 1.007 | 2.639 | 1.55197 |
| JFBM48585 | 4 | 93.33 | Hymenoptera terrestrial | 1.437 | 5.129 | 8.09563 |
| JFBM48585 | 4 | 93.33 | Hymenoptera terrestrial | 1.128 | 3.961 | 3.90168 |
| JFBM48585 | 4 | 93.33 | Hymenoptera terrestrial | 1.007 | 3.625 | 6.87231 |
| JFBM48585 | 4 | 93.33 | Seed | 0.743 | 2.257 | 1.49436 |
| JFBM48585 | 4 | 93.33 | Seed | 0.635 | 1.58 | 0.78815 |
| JFBM48585 | 5 | 99.06 | Insecta terrestrial Culicidae | 2.604 | 8.114 | 32.86195 |
| JFBM48585 | 5 | 99.06 | Insecta aquatic diptera pupa | 1.132 | 6.428 | 2.43895 |
| JFBM48585 | 5 | 99.06 | Insecta aquatic diptera pupa | 0.951 | 4.832 | 6.06354 |
| JFBM48585 | 5 | 99.06 | Insecta terrestrial Hemiptera | 0.983 | 2.072 | 1.84163 |
| JFBM48585 | 5 | 99.06 | Insecta terrestrial Hymenoptera | 1.424 | 3.908 | 7.07806 |
| JFBM48585 | 5 | 99.06 | Insecta terrestrial Hymenoptera | 1.329 | 4.162 | 5.41679 |
| JFBM48585 | 5 | 99.06 | Insecta terrestrial Hymenoptera | 1.532 | 4.375 | 6.33069 |
| JFBM48585 | 5 | 99.06 | Insecta terrestrial Hymenoptera | 1.157 |  | 2.17553 |
| JFBM48585 | 5 | 99.06 | Insecta terrestrial Hymenoptera | 1.211 |  | 2.30552 |
| JFBM48585 | 5 | 99.06 | Insecta terrestrial Hymenoptera | 1.237 | 4.576 | 8.29323 |
| JFBM48585 | 5 | 99.06 | Insecta terrestrial Hymenoptera | 1.113 | 3.782 | 7.31 |
| JFBM48585 | 5 | 99.06 | Insecta terrestrial Hymenoptera | 1.459 | 4.687 | 7.27795 |
| JFBM48585 | 5 | 99.06 | Insecta terrestrial Hymenoptera | 1.41 |  | 2.78455 |
| JFBM48585 | 5 | 99.06 | Insecta terrestrial Hymenoptera | 1.163 | 4.606 | 7.10222 |
| JFBM48585 | 5 | 99.06 | Insecta terrestrial Hymenoptera | 1.027 |  | 1.86259 |
| JFBM48585 | 5 | 99.06 | Insecta terrestrial Hymenoptera | 1.548 |  | 3.11675 |
| JFBM48585 | 6 | 97.61 | Insecta terrestrial | 1.081 | 4.587 | 5.96881 |
| JFBM48585 | 6 | 97.61 | Insecta larvae aquatic Trichoptera | 1.25 |  | 2.3994 |
| JFBM48585 | 8 | 99.39 | Amphipoda | 0.611 | 3.248 | 1.31517 |
| JFBM48585 | 8 | 99.39 | Insecta terrestrial Culicidae | 1.226 | 4 | 5.63147 |
| JFBM48585 | 8 | 99.39 | Insecta terrestrial Culicidae | 0.46 | 1.83 | 0.32321 |
| JFBM48585 | 8 | 99.39 | Insecta larvae aquatic diptera | 1.108 | 5.861 | 7.3531 |
| JFBM48585 | 8 | 99.39 | Insecta larvae aquatic diptera | 0.385 | 5.769 | 1.10135 |
| JFBM48585 | 8 | 99.39 | Insecta larvae aquatic diptera | 0.585 | 5.963 | 2.36774 |
| JFBM48585 | 8 | 99.39 | Insecta larvae aquatic diptera | 1.489 | 4.62 | 5.64855 |
| JFBM48585 | 8 | 99.39 | Insecta terrestrial hymenoptera | 1.646 | 5.216 | 9.39323 |
| JFBM48585 | 8 | 99.39 | Insecta larvae aquatic | 1.382 |  | 2.71715 |
| JFBM48585 | 8 | 99.39 | Insecta larvae aquatic | 0.907 | 5.689 | 3.13278 |
| JFBM48585 | 9 | 95.52 | Insecta terrestrial | 2.179 | 5.667 | 47.16153 |
| JFBM48585 | 9 | 95.52 | Trichoptera larvae aquatic | 0.847 | 4.698 | 4.66587 |
| JFBM48585 | 10 | 99.17 | Trichoptera larvae aquatic | 1.343 | 7.743 | 23.87723 |
| JFBM48585 | 11 | 93.67 | Hymenoptera terrestrial | 0.99 |  | 1.77353 |
| JFBM48585 | 11 | 93.67 | Hymenoptera terrestrial | 1.263 | 3.636 | 5.63633 |
| JFBM48585 | 11 | 93.67 | Hymenoptera terrestrial | 1.154 | 3.924 | 11.28567 |
| JFBM48585 | 11 | 93.67 | Hymenoptera terrestrial | 1.273 | 4.696 | 9.76392 |
| JFBM48585 | 11 | 93.67 | Hymenoptera terrestrial | 1.4 | 3.933 | 7.05684 |
| JFBM48585 | 12 | 92.76 | Copepoda Calanoida | 0.377 | 0.756 | 0.10046 |
| JFBM48585 | 12 | 92.76 | Copepoda Calanoida | 0.32 | 0.64 | 0.04621 |
| JFBM48585 | 12 | 92.76 | Copepoda | 0.313 |  | 0.01514 |
| JFBM48585 | 12 | 92.76 | Copepoda | 0.275 |  | 0.01018 |
| JFBM48585 | 12 | 92.76 | Copepoda | 0.319 |  | 0.01605 |
| JFBM48585 | 12 | 92.76 | Copepoda | 0.271 |  | 0.00984 |
| JFBM48585 | 12 | 92.76 | Copepoda | 0.224 |  | 0.00606 |
| JFBM48585 | 12 | 92.76 | Copepoda | 0.303 | 0.815 | 0.1292 |
| JFBM48585 | 12 | 92.76 | Copepoda Calanoida | 0.441 | 1.169 | 0.19479 |
| JFBM48585 | 12 | 92.76 | Copepoda Calanoida | 0.245 | 0.831 | 0.12203 |
| JFBM48585 | 12 | 92.76 | Copepoda Calanoida | 0.253 |  | 0.00814 |
| JFBM48585 | 12 | 92.76 | Copepoda Calanoida | 0.26 |  | 0.00876 |
| JFBM48585 | 12 | 92.76 | Copepoda Calanoida | 0.34 |  | 0.01988 |
| JFBM48585 | 12 | 92.76 | Copepoda Calanoida | 0.313 |  | 0.01512 |
| JFBM48585 | 12 | 92.76 | Copepoda Calanoida | 0.286 | 0.939 | 0.0713 |
| JFBM48585 | 12 | 92.76 | Copepoda Calanoida | 0.412 | 0.822 | 0.19142 |
| JFBM48585 | 12 | 92.76 | Copepoda Calanoida | 0.276 |  | 0.0103 |
| JFBM48585 | 12 | 92.76 | Copepoda Calanoida | 0.231 |  | 0.00647 |
| JFBM48585 | 12 | 92.76 | Copepoda Calanoida | 0.523 | 0.809 | 0.15239 |
| JFBM48585 | 12 | 92.76 | Copepoda Calanoida | 0.412 | 1.179 | 0.18826 |
| JFBM48585 | 12 | 92.76 | Copepoda Calanoida | 0.383 |  | 0.0312 |
| JFBM48585 | 12 | 92.76 | Copepoda Calanoida | 0.45 | 0.932 | 0.20796 |
| JFBM48585 | 12 | 92.76 | Copepoda Calanoida | 0.423 | 0.91 | 0.14387 |
| JFBM48585 | 12 | 92.76 | Copepoda Calanoida | 0.416 | 0.782 | 0.16837 |
| JFBM48585 | 12 | 92.76 | Copepoda Calanoida | 0.389 | 1.073 | 0.13903 |
| JFBM48585 | 12 | 92.76 | Copepoda Calanoida | 0.31 |  | 0.01459 |
| JFBM48585 | 12 | 92.76 | Copepoda Calanoida | 0.266 | 0.859 | 0.10415 |
| JFBM48585 | 12 | 92.76 | Copepoda Calanoida | 0.398 |  | 0.03644 |
| JFBM48585 | 12 | 92.76 | Copepoda Calanoida | 0.375 |  | 0.02869 |
| JFBM48585 | 12 | 92.76 | Copepoda Calanoida | 0.449 | 0.943 | 0.17721 |
| JFBM48585 | 12 | 92.76 | Copepoda Calanoida | 0.418 | 0.801 | 0.16753 |
| JFBM48585 | 12 | 92.76 | Copepoda Calanoida | 0.337 |  | 0.01942 |
| JFBM48585 | 12 | 92.76 | Copepoda Calanoida | 0.421 | 0.809 | 0.1512 |
| JFBM48585 | 12 | 92.76 | Copepoda Calanoida | 0.377 | 0.875 | 0.14913 |
| JFBM48585 | 12 | 92.76 | Copepoda Calanoida | 0.399 |  | 0.03685 |
| JFBM48585 | 12 | 92.76 | Copepoda Calanoida | 0.505 | 0.971 | 0.25089 |
| JFBM48585 | 12 | 92.76 | Copepoda Calanoida | 0.372 | 0.834 | 0.14888 |
| JFBM48585 | 12 | 92.76 | Copepoda Calanoida | 0.287 |  | 0.01157 |
| JFBM48585 | 13 | 91.64 | Copepoda | 0.427 |  | 0.04917 |
| JFBM48585 | 13 | 91.64 | Copepoda | 0.289 |  | 0.01183 |
| JFBM48585 | 13 | 91.64 | Copepoda | 0.357 |  | 0.02382 |
| JFBM48585 | 13 | 91.64 | Copepoda | 0.444 |  | 0.05858 |
| JFBM48585 | 13 | 91.64 | Copepoda | 0.322 |  | 0.01655 |
| JFBM48585 | 13 | 91.64 | Copepoda | 0.381 |  | 0.03053 |
| JFBM48585 | 13 | 91.64 | Copepoda | 0.426 |  | 0.04848 |
| JFBM48585 | 13 | 91.64 | Copepoda | 0.292 |  | 0.0122 |
| JFBM48585 | 13 | 91.64 | Copepoda | 0.466 |  | 0.07294 |
| JFBM48585 | 13 | 91.64 | Copepoda | 0.346 |  | 0.02131 |
| JFBM48585 | 13 | 91.64 | Copepoda | 0.251 |  | 0.00794 |
| JFBM48585 | 13 | 91.64 | Copepoda | 0.454 |  | 0.06482 |
| JFBM48585 | 13 | 91.64 | Copepoda | 0.324 |  | 0.01695 |
| JFBM48585 | 13 | 91.64 | Copepoda | 0.296 |  | 0.01274 |
| JFBM48585 | 13 | 91.64 | Copepoda | 0.282 |  | 0.01097 |
| JFBM48585 | 13 | 91.64 | Copepoda | 0.265 |  | 0.00919 |
| JFBM48585 | 13 | 91.64 | Copepoda | 0.464 |  | 0.07208 |
| JFBM48585 | 13 | 91.64 | Copepoda | 0.299 |  | 0.01306 |
| JFBM48585 | 13 | 91.64 | Copepoda | 0.27 |  | 0.00972 |
| JFBM48585 | 13 | 91.64 | Copepoda | 0.359 |  | 0.02436 |
| JFBM48585 | 13 | 91.64 | Copepoda | 0.379 |  | 0.02991 |
| JFBM48585 | 13 | 91.64 | Copepoda | 0.451 |  | 0.06271 |
| JFBM48585 | 13 | 91.64 | Copepoda | 0.269 |  | 0.00958 |
| JFBM48585 | 13 | 91.64 | Copepoda | 0.35 |  | 0.02219 |
| JFBM48585 | 13 | 91.64 | Copepoda | 0.365 |  | 0.02573 |
| JFBM48585 | 13 | 91.64 | Copepoda | 0.436 |  | 0.05352 |
| JFBM48585 | 13 | 91.64 | Copepoda | 0.369 |  | 0.02693 |
| JFBM48585 | 13 | 91.64 | Diptera larvae aquatic | 0.128 |  | 0.00224 |
| JFBM48585 | 14 | 88.91 | Copepoda | 0.395 |  | 0.03513 |
| JFBM48585 | 14 | 88.91 | Copepoda | 0.097 |  | 0.00163 |
| JFBM48585 | 14 | 88.91 | Copepoda | 0.341 |  | 0.02017 |
| JFBM48585 | 14 | 88.91 | Copepoda | 0.391 |  | 0.03396 |
| JFBM48585 | 14 | 88.91 | Copepoda | 0.363 |  | 0.02534 |
| JFBM48585 | 14 | 88.91 | Copepoda | 0.301 |  | 0.01337 |
| JFBM48585 | 14 | 88.91 | Copepoda | 0.317 |  | 0.01577 |
| JFBM48585 | 14 | 88.91 | Copepoda | 0.363 |  | 0.02535 |
| JFBM48585 | 14 | 88.91 | Copepoda | 0.44 |  | 0.05578 |
| JFBM48585 | 14 | 88.91 | Copepoda | 0.448 |  | 0.06111 |
| JFBM48585 | 14 | 88.91 | Copepoda | 0.354 |  | 0.02318 |
| JFBM48585 | 14 | 88.91 | Copepoda | 0.316 |  | 0.01562 |
| JFBM48585 | 14 | 88.91 | Copepoda | 0.463 |  | 0.07082 |
| JFBM48585 | 14 | 88.91 | Copepoda | 0.36 |  | 0.02464 |
| JFBM48585 | 14 | 88.91 | Copepoda | 0.42 |  | 0.04561 |
| JFBM48585 | 14 | 88.91 | Copepoda | 0.493 |  | 0.09682 |
| JFBM48585 | 14 | 88.91 | Copepoda | 0.424 |  | 0.04776 |
| JFBM48585 | 14 | 88.91 | Copepoda | 0.418 |  | 0.04452 |
| JFBM48585 | 14 | 88.91 | Copepoda | 0.358 |  | 0.02401 |
| JFBM48585 | 14 | 88.91 | Copepoda | 0.476 |  | 0.08142 |
| JFBM48585 | 14 | 88.91 | Copepoda | 0.438 |  | 0.05517 |
| JFBM48585 | 14 | 88.91 | Copepoda | 0.472 |  | 0.07831 |
| JFBM48585 | 14 | 88.91 | Copepoda | 0.345 |  | 0.02097 |
| JFBM48585 | 14 | 88.91 | Copepoda | 0.44 |  | 0.05584 |
| JFBM48585 | 14 | 88.91 | Copepoda | 0.357 |  | 0.0239 |
| JFBM48585 | 14 | 88.91 | Copepoda | 0.499 |  | 0.10276 |
| JFBM48585 | 14 | 88.91 | Copepoda | 0.513 |  | 0.11952 |
| JFBM48585 | 14 | 88.91 | Copepoda | 0.51 |  | 0.1158 |
| JFBM48585 | 14 | 88.91 | Copepoda | 0.393 |  | 0.03434 |
| JFBM48585 | 14 | 88.91 | Copepoda | 0.32 |  | 0.01631 |
| JFBM48585 | 14 | 88.91 | Copepoda | 0.295 |  | 0.01258 |
| JFBM48585 | 14 | 88.91 | Copepoda | 0.403 |  | 0.03823 |
| JFBM48585 | 14 | 88.91 | Copepoda | 0.413 |  | 0.04243 |
| JFBM48585 | 14 | 88.91 | Copepoda | 0.316 |  | 0.01555 |
| JFBM48585 | 14 | 88.91 | Copepoda | 0.417 |  | 0.0444 |
| JFBM48585 | 14 | 88.91 | Copepoda | 0.366 |  | 0.02615 |
| JFBM48585 | 14 | 88.91 | Copepoda | 0.306 |  | 0.01401 |
| JFBM48585 | 14 | 88.91 | Copepoda | 0.346 |  | 0.02118 |
| JFBM48585 | 14 | 88.91 | Copepoda | 0.404 |  | 0.03883 |
| JFBM48585 | 14 | 88.91 | Copepoda | 0.365 |  | 0.02574 |
| JFBM48585 | 14 | 88.91 | Copepoda | 0.409 |  | 0.04088 |
| JFBM48585 | 14 | 88.91 | Copepoda | 0.418 |  | 0.04479 |
| JFBM48585 | 14 | 88.91 | Copepoda | 0.332 |  | 0.01839 |
| JFBM48585 | 14 | 88.91 | Copepoda | 0.467 |  | 0.07379 |
| JFBM48585 | 15 | 89.96 | Copepoda | 0.48 |  | 0.0849 |
| JFBM48585 | 15 | 89.96 | Copepoda | 0.341 |  | 0.0202 |
| JFBM48585 | 15 | 89.96 | Copepoda | 0.417 |  | 0.04416 |
| JFBM48585 | 15 | 89.96 | Copepoda | 0.382 |  | 0.03066 |
| JFBM48585 | 15 | 89.96 | Copepoda | 0.369 |  | 0.02698 |
| JFBM48585 | 15 | 89.96 | Copepoda | 0.39 |  | 0.03336 |
| JFBM48585 | 15 | 89.96 | Copepoda | 0.491 |  | 0.09498 |
| JFBM48585 | 15 | 89.96 | Copepoda | 0.397 |  | 0.03604 |
| JFBM48585 | 15 | 89.96 | Copepoda | 0.402 |  | 0.03806 |
| JFBM48585 | 15 | 89.96 | Copepoda | 0.451 |  | 0.06304 |
| JFBM48585 | 15 | 89.96 | Copepoda | 0.44 |  | 0.05589 |
| JFBM48585 | 15 | 89.96 | Copepoda | 0.475 |  | 0.08059 |
| JFBM48585 | 15 | 89.96 | Copepoda | 0.366 |  | 0.02606 |
| JFBM48585 | 15 | 89.96 | Copepoda | 0.379 |  | 0.02984 |
| JFBM48585 | 15 | 89.96 | Copepoda | 0.431 |  | 0.05109 |
| JFBM48585 | 15 | 89.96 | Copepoda | 0.457 |  | 0.0667 |
| JFBM48585 | 15 | 89.96 | Copepoda | 0.348 |  | 0.02176 |
| JFBM48585 | 15 | 89.96 | Copepoda | 0.374 |  | 0.02837 |
| JFBM48585 | 15 | 89.96 | Copepoda | 0.392 |  | 0.03408 |
| JFBM48585 | 19 |  | Copepoda Calanoida | 0.11 | 0.181 | 0.00054 |
| JFBM48585 | 19 |  | Copepoda Calanoida | 0.376 | 0.742 | 0.01197 |
| JFBM48585 | 19 |  | Copepoda Calanoida | 0.42 | 0.789 | 0.02249 |
| JFBM48585 | 19 |  | Copepoda Calanoida | 0.364 | 0.657 | 0.01102 |
| JFBM48585 | 19 |  | Copepoda Calanoida | 0.358 | 0.545 | 0.00775 |
| JFBM48585 | 19 |  | Copepoda Calanoida | 0.355 | 0.749 | 0.01416 |
| JFBM48585 | 19 |  | Copepoda Calanoida | 0.313 |  | 0.01514 |
| JFBM48585 | 19 |  | Copepoda Calanoida | 0.354 |  | 0.02317 |
| JFBM48585 | 20 | 84.52 | Copepoda | 0.722 | 1.018 | 0.05322 |
| JFBM48585 | 20 | 84.52 | Copepoda | 0.513 | 0.942 | 0.01804 |
| JFBM48585 | 20 | 84.52 | Insecta terrestrial Formicidae | 1.702 |  | 3.48745 |
| JFBM48585 | 20 | 84.52 | Insecta terrestrial Formicidae | 1.786 |  | 3.68966 |
| JFBM48585 | 20 | 84.52 | Insecta terrestrial Formicidae | 1.744 |  | 3.58856 |
| JFBM48585 | 20 | 84.52 | Insecta terrestrial Formicidae | 1.436 |  | 2.84714 |
| JFBM48585 | 20 | 84.52 | Insecta terrestrial Formicidae | 1.602 |  | 3.24673 |
| JFBM48585 | 20 | 84.52 | Insecta terrestrial Formicidae | 1.852 |  | 3.84853 |
| JFBM48585 | 20 | 84.52 | Insecta terrestrial Formicidae | 1.682 | 6.216 | 2.72258 |
| JFBM48585 | 21 | 76.31 | Copepoda | 0.371 | 0.681 | 0.00911 |
| JFBM48585 | 21 | 76.31 | Copepoda | 0.441 |  | 0.05656 |
| JFBM48585 | 21 | 76.31 | Copepoda | 0.431 | 0.763 | 0.01336 |
| JFBM48585 | 21 | 76.31 | Copepoda | 0.456 | 0.962 | 0.02321 |
| JFBM48585 | 21 | 76.31 | Copepoda | 0.431 |  | 0.05126 |
| JFBM48585 | 21 | 76.31 | Copepoda Calanoida | 0.466 | 1.121 | 0.02245 |
| JFBM48585 | 21 | 76.31 | Copepoda Calanoida | 0.481 | 0.859 | 0.02353 |
| JFBM48585 | 21 | 76.31 | Copepoda Calanoida | 0.372 | 1.043 | 0.02674 |
| JFBM48585 | 21 | 76.31 | Copepoda Calanoida | 0.295 |  | 0.01255 |
| JFBM48585 | 21 | 76.31 | Copepoda Calanoida | 0.379 | 0.869 | 0.01586 |
| JFBM48585 | 21 | 76.31 | Copepoda Calanoida | 0.409 | 0.748 | 0.01344 |
| JFBM48585 | 21 | 76.31 | Copepoda Calanoida | 0.44 | 1.089 | 0.0312 |
| JFBM48585 | 21 | 76.31 | Copepoda | 0.474 |  | 0.07986 |
| JFBM48585 | 21 | 76.31 | Copepoda Calanoida | 0.488 |  | 0.09203 |
| JFBM48585 | 21 | 76.31 | Copepoda Calanoida | 0.481 | 0.896 | 0.0267 |
| JFBM48585 | 21 | 76.31 | Copepoda Calanoida | 0.409 | 0.962 | 0.02329 |
| JFBM48585 | 21 | 76.31 | Copepoda Calanoida | 0.343 | 1.067 | 0.01351 |
| JFBM48585 | 21 | 76.31 | Copepoda Calanoida | 0.321 | 0.771 | 0.01044 |
| JFBM48585 | 21 | 76.31 | Copepoda Calanoida | 0.376 |  | 0.02899 |
| JFBM48585 | 21 | 76.31 | Copepoda Calanoida | 0.45 | 0.906 | 0.02508 |
| JFBM48585 | 21 | 76.31 | Copepoda Calanoida | 0.398 | 0.88 | 0.01287 |
| JFBM48585 | 21 | 76.31 | Copepoda Calanoida | 0.353 | 1.025 | 0.01754 |
| JFBM48585 | 21 | 76.31 | Copepoda Calanoida | 0.335 |  | 0.01895 |
| JFBM48585 | 21 | 76.31 | Copepoda Calanoida | 0.211 | 0.49 | 0.00254 |
| JFBM48585 | 22 | 77.75 | Copepoda Calanoida | 0.353 |  | 0.02282 |
| JFBM48585 | 22 | 77.75 | Copepoda Calanoida | 0.247 |  | 0.00768 |
| JFBM48585 | 22 | 77.75 | Copepoda Calanoida | 0.328 | 0.923 | 0.02093 |
| JFBM48585 | 22 | 77.75 | Copepoda Calanoida | 0.396 | 0.797 | 0.02296 |
| JFBM48585 | 22 | 77.75 | Copepoda | 0.522 | 0.768 | 0.01905 |
| JFBM48585 | 22 | 77.75 | Copepoda Calanoida | 0.315 | 0.528 | 0.00623 |
| JFBM48585 | 22 | 77.75 | Copepoda Calanoida | 0.44 |  | 0.05583 |
| JFBM48585 | 22 | 77.75 | Copepoda | 0.449 | 0.883 | 0.01621 |
| JFBM48585 | 22 | 77.75 | Copepoda | 0.362 | 0.844 | 0.01764 |
| JFBM48585 | 22 | 77.75 | Copepoda | 0.381 | 0.788 | 0.02748 |
| JFBM48585 | 22 | 77.75 | Copepoda | 0.362 | 0.892 | 0.01865 |
| JFBM48585 | 22 | 77.75 | Copepoda | 0.332 |  | 0.01837 |
| JFBM48585 | 22 | 77.75 | Copepoda | 0.245 | 0.683 | 0.00746 |
| JFBM48585 | 22 | 77.75 | Copepoda | 0.204 | 0.468 | 0.00136 |
| JFBM48585 | 22 | 77.75 | Copepoda | 0.333 |  | 0.01851 |
| JFBM48585 | 22 | 77.75 | Copepoda | 0.391 | 0.908 | 0.02386 |
| JFBM48585 | 22 | 77.75 | Copepoda | 0.416 | 0.81 | 0.01455 |
| JFBM48585 | 22 | 77.75 | Copepoda | 0.496 | 0.927 | 0.02513 |
| JFBM48585 | 22 | 77.75 | Copepoda | 0.395 | 0.709 | 0.01317 |
| JFBM48585 | 22 | 77.75 | Copepoda | 0.244 |  | 0.00744 |
| JFBM48585 | 22 | 77.75 | Copepoda | 0.476 | 0.845 | 0.0256 |
| JFBM48585 | 23 | 78.09 | Copepoda | 0.427 | 0.9 | 0.16097 |
| JFBM48585 | 23 | 78.09 | Copepoda | 0.504 | 0.892 | 0.20574 |
| JFBM48585 | 23 | 78.09 | Copepoda | 0.342 |  | 0.02035 |
| JFBM48585 | 23 | 78.09 | Copepoda | 0.459 | 0.809 | 0.24191 |
| JFBM48585 | 23 | 78.09 | Copepoda | 0.475 | 0.809 | 0.30133 |
| JFBM48585 | 23 | 78.09 | Copepoda | 0.533 | 0.891 | 0.33003 |
| JFBM48585 | 23 | 78.09 | Copepoda | 0.477 | 0.881 | 0.28011 |
| JFBM48585 | 23 | 78.09 | Copepoda | 0.424 |  | 0.04763 |
| JFBM48585 | 23 | 78.09 | Copepoda | 0.47 | 0.85 | 0.16674 |
| JFBM48585 | 23 | 78.09 | Copepoda | 0.468 | 0.65 | 0.18693 |
| JFBM48585 | 23 | 78.09 | Copepoda | 0.379 | 1.256 | 0.2392 |
| JFBM48585 | 23 | 78.09 | Copepoda | 0.436 | 0.906 | 0.1804 |
| JFBM48585 | 23 | 78.09 | Copepoda | 0.5 | 0.875 | 0.26169 |
| JFBM48585 | 23 | 78.09 | Copepoda | 0.496 | 0.826 | 0.26385 |
| JFBM48585 | 23 | 78.09 | Copepoda | 0.429 | 0.758 | 0.18968 |
| JFBM48585 | 23 | 78.09 | Copepoda | 0.35 |  | 0.02218 |
| JFBM48585 | 23 | 78.09 | Copepoda | 0.477 | 0.885 | 0.34518 |
| JFBM48585 | 23 | 78.09 | Copepoda | 0.519 | 1.052 | 0.2739 |
| JFBM48585 | 23 | 78.09 | Copepoda | 0.57 |  | 0.21367 |
| JFBM48585 | 23 | 78.09 | Copepoda | 0.359 | 0.777 | 0.08938 |
| JFBM48585 | 23 | 78.09 | Copepoda | 0.442 | 0.761 | 0.18288 |
| JFBM48585 | 24 | 82.02 | Copepoda | 0.395 |  | 0.03515 |
| JFBM48585 | 24 | 82.02 | Copepoda | 0.396 |  | 0.03574 |
| JFBM48585 | 24 | 82.02 | Copepoda | 0.329 |  | 0.01775 |
| JFBM48585 | 24 | 82.02 | Copepoda | 0.359 | 0.851 | 0.01109 |
| JFBM48585 | 24 | 82.02 | Copepoda | 0.424 | 1.081 | 0.02128 |
| JFBM48585 | 24 | 82.02 | Copepoda | 0.296 | 0.702 | 0.00253 |
| JFBM48585 | 24 | 82.02 | Copepoda | 0.317 | 0.607 | 0.00896 |
| JFBM48585 | 24 | 82.02 | Copepoda | 0.36 | 0.856 | 0.01296 |
| JFBM48585 | 24 | 82.02 | Copepoda | 0.356 | 1.017 | 0.0216 |
| JFBM48585 | 24 | 82.02 | Copepoda | 0.369 | 0.737 | 0.00986 |
| JFBM48585 | 24 | 82.02 | Copepoda | 0.508 |  | 0.1129 |
| JFBM48585 | 24 | 82.02 | Copepoda | 0.445 | 0.813 | 0.02819 |
| JFBM48585 | 24 | 82.02 | Copepoda | 0.448 | 0.825 | 0.0198 |
| JFBM48585 | 24 | 82.02 | Copepoda | 0.35 | 0.646 | 0.00806 |
| JFBM48585 | 24 | 82.02 | Copepoda | 0.374 |  | 0.02847 |
| JFBM48585 | 24 | 82.02 | Copepoda | 0.421 | 0.786 | 0.01574 |
| JFBM48585 | 24 | 82.02 | Copepoda | 0.467 | 0.848 | 0.02143 |
| JFBM48585 | 25 | 72.17 | Copepoda | 0.475 |  | 0.08071 |
| JFBM48585 | 25 | 72.17 | Copepoda | 0.426 |  | 0.04835 |
| JFBM48585 | 25 | 72.17 | Copepoda | 0.411 | 0.967 | 0.01712 |
| JFBM48585 | 25 | 72.17 | Copepoda | 0.357 | 0.919 | 0.01617 |
| JFBM48585 | 25 | 72.17 | Copepoda | 0.339 |  | 0.01971 |
| JFBM48585 | 25 | 72.17 | Copepoda | 0.416 | 0.738 | 0.01735 |
| JFBM48585 | 25 | 72.17 | Copepoda | 0.316 | 0.707 | 0.00874 |
| JFBM48585 | 25 | 72.17 | Copepoda | 0.417 | 0.737 | 0.01645 |
| JFBM48585 | 25 | 72.17 | Copepoda | 0.392 | 0.901 | 0.017 |
| JFBM48585 | 25 | 72.17 | Copepoda | 0.384 | 0.715 | 0.00886 |
| JFBM48585 | 25 | 72.17 | Copepoda | 0.446 | 0.792 | 0.0142 |
| JFBM48585 | 25 | 72.17 | Copepoda | 0.398 | 0.971 | 0.02112 |
| JFBM48585 | 25 | 72.17 | Copepoda | 0.402 |  | 0.03785 |
| JFBM48585 | 25 | 72.17 | Copepoda | 0.291 |  | 0.01211 |
| JFBM48585 | 25 | 72.17 | Copepoda | 0.483 |  | 0.08742 |
| JFBM48585 | 25 | 72.17 | Copepoda | 0.382 | 0.594 | 0.00609 |
| JFBM48585 | 25 | 72.17 | Copepoda | 0.476 | 0.818 | 0.02152 |
| JFBM48585 | 25 | 72.17 | Copepoda | 0.334 |  | 0.01877 |
| JFBM48585 | 25 | 72.17 | Hymenoptera terrestrial | 1.812 |  | 3.75225 |
| JFBM48585 | 25 | 72.17 | Hymenoptera terrestrial | 2.836 | 8.23 | 7.29803 |
| JFBM48585 | 26 | 73.9 | Copepoda | 0.363 | 0.55 | 0.05431 |
| JFBM48585 | 26 | 73.9 | Copepoda | 0.361 | 0.599 | 0.1053 |
| JFBM48585 | 26 | 73.9 | Copepoda | 0.329 | 0.65 | 0.11425 |
| JFBM48585 | 26 | 73.9 | Copepoda | 0.529 | 0.858 | 0.22791 |
| JFBM48585 | 26 | 73.9 | Copepoda | 0.434 | 0.883 | 0.20186 |
| JFBM48585 | 26 | 73.9 | Copepoda | 0.498 |  | 0.10245 |
| JFBM48585 | 26 | 73.9 | Copepoda | 0.435 | 0.71 | 0.17424 |
| JFBM48585 | 26 | 73.9 | Copepoda | 0.349 | 0.704 | 0.17817 |
| JFBM48585 | 26 | 73.9 | Copepoda | 0.504 | 0.837 | 0.37595 |
| JFBM48585 | 26 | 73.9 | Copepoda | 0.473 | 0.81 | 0.21445 |
| JFBM48585 | 26 | 73.9 | Copepoda | 0.344 | 0.715 | 0.13633 |
| JFBM48585 | 26 | 73.9 | Copepoda | 0.501 | 0.884 | 0.24356 |
| JFBM48585 | 26 | 73.9 | Copepoda | 0.26 | 0.496 | 0.06589 |
| JFBM48585 | 26 | 73.9 | Copepoda | 0.292 | 0.501 | 0.08083 |
| JFBM48585 | 26 | 73.9 | Copepoda | 0.44 | 0.683 | 0.15587 |
| JFBM48585 | 26 | 73.9 | Copepoda | 0.466 | 0.801 | 0.23383 |
| JFBM48585 | 26 | 73.9 | Copepoda | 0.432 | 0.821 | 0.23633 |
| JFBM48585 | 26 | 73.9 | Copepoda | 0.424 | 0.682 | 0.1724 |
| JFBM48585 | 26 | 73.9 | Copepoda | 0.515 | 0.891 | 0.24286 |
| JFBM48585 | 26 | 73.9 | Copepoda | 0.43 | 0.662 | 0.17983 |
| JFBM48585 | 26 | 73.9 | Copepoda | 0.427 |  | 0.04904 |
| JFBM48585 | 26 | 73.9 | Copepoda | 0.414 | 0.841 | 0.23459 |
| JFBM48585 | 26 | 73.9 | Copepoda | 0.481 | 0.848 | 0.3022 |
| JFBM48585 | 26 | 73.9 | Copepoda | 0.477 | 0.81 | 0.15553 |
| JFBM48585 | 26 | 73.9 | Copepoda | 0.422 | 0.691 | 0.16686 |
| JFBM48585 | 26 | 73.9 | Formicidae (ant) | 1.38 | 3.525 | 2.71234 |
| JFBM48585 | 26 | 73.9 | Formicidae (ant) | 0.981 | 3.515 | 1.75186 |
| JFBM48585 | 26 | 73.9 | Formicidae (ant) | 1.05 | 4.116 | 1.91796 |
| JFBM48585 | 27 | 79.92 | Copepoda | 0.39 | 0.79 | 0.01398 |
| JFBM48585 | 27 | 79.92 | Copepoda | 0.459 |  | 0.06799 |
| JFBM48585 | 27 | 79.92 | Copepoda | 0.321 | 0.737 | 0.00987 |
| JFBM48585 | 27 | 79.92 | Copepoda | 0.425 | 0.821 | 0.01841 |
| JFBM48585 | 27 | 79.92 | Copepoda | 0.322 | 0.78 | 0.01289 |
| JFBM48585 | 27 | 79.92 | Copepoda | 0.489 | 0.877 | 0.02397 |
| JFBM48585 | 27 | 79.92 | Copepoda | 0.4 | 0.617 | 0.01275 |
| JFBM48585 | 27 | 79.92 | Copepoda | 0.307 |  | 0.01421 |
| JFBM48585 | 27 | 79.92 | Copepoda | 0.366 | 0.771 | 0.02118 |
| JFBM48585 | 27 | 79.92 | Copepoda | 0.314 |  | 0.01524 |
| JFBM48585 | 27 | 79.92 | Copepoda | 0.354 |  | 0.02303 |
| JFBM48585 | 27 | 79.92 | Copepoda | 0.301 |  | 0.01331 |
| JFBM48585 | 27 | 79.92 | Copepoda | 0.314 | 0.543 | 0.0127 |
| JFBM48585 | 27 | 79.92 | Copepoda | 0.34 | 0.864 | 0.00985 |
| JFBM48585 | 27 | 79.92 | Copepoda | 0.349 | 0.732 | 0.01393 |
| JFBM48585 | 27 | 79.92 | Copepoda | 0.518 | 0.847 | 0.02427 |
| JFBM48585 | 27 | 79.92 | Copepoda | 0.458 |  | 0.0673 |
| JFBM48585 | 27 | 79.92 | Copepoda | 0.205 | 0.428 | 0.00281 |
| JFBM48585 | 27 | 79.92 | Copepoda | 0.393 | 0.823 | 0.01171 |
| JFBM48585 | 27 | 79.92 | Copepoda | 0.516 | 0.855 | 0.02542 |
| JFBM48585 | 27 | 79.92 | Copepoda | 0.425 | 0.824 | 0.01935 |
| JFBM48585 | 27 | 79.92 | Copepoda | 0.367 |  | 0.02639 |
| JFBM48585 | 27 | 79.92 | Copepoda | 0.506 | 0.769 | 0.02653 |
| JFBM48585 | 27 | 79.92 | Copepoda | 0.474 | 0.881 | 0.02293 |
| JFBM48585 | 27 | 79.92 | Copepoda | 0.317 |  | 0.01578 |
| JFBM48585 | 27 | 79.92 | Copepoda | 0.341 | 0.827 | 0.0132 |
| JFBM48585 | 27 | 79.92 | Copepoda | 0.321 |  | 0.01649 |
| JFBM48585 | 27 | 79.92 | Copepoda | 0.339 | 0.842 | 0.01941 |
| JFBM48585 | 27 | 79.92 | Copepoda | 0.387 |  | 0.03236 |
| JFBM48585 | 27 | 79.92 | Copepoda | 0.346 |  | 0.02116 |
| JFBM48585 | 27 | 79.92 | Copepoda | 0.455 | 0.877 | 0.01937 |
| JFBM48585 | 27 | 79.92 | Copepoda | 0.483 | 0.891 | 0.02568 |
| JFBM48585 | 27 | 79.92 | Copepoda | 0.328 | 0.724 | 0.00745 |
| JFBM48585 | 27 | 79.92 | Copepoda | 0.393 |  | 0.03451 |
| JFBM48585 | 27 | 79.92 | Copepoda | 0.428 | 0.809 | 0.01808 |
| JFBM48585 | 27 | 79.92 | Copepoda | 0.536 | 0.879 | 0.02692 |
| JFBM48585 | 27 | 79.92 | Copepoda | 0.454 |  | 0.06487 |
| JFBM48585 | 27 | 79.92 | Hymenoptera terrestrial | 2.182 |  | 4.64291 |
| JFBM48585 | 27 | 79.92 | Hymenoptera terrestrial | 1.906 |  | 3.97852 |
| JFBM48585 | 27 | 79.92 | Hymenoptera terrestrial | 2.996 | 8.764 | 9.14739 |
| JFBM48585 | 27 | 79.92 | Hymenoptera terrestrial | 2.794 |  | 6.11612 |
| JFBM48585 | 27 | 79.92 | Hymenoptera terrestrial | 2.382 | 8.228 | 5.3614 |
| JFBM48585 | 27 | 79.92 | Insecta terrestrial | 2.198 | 5.914 | 4.04542 |
| JFBM48585 | 27 | 79.92 | Hymenoptera terrestrial | 1.808 |  | 3.74262 |
| JFBM48585 | 27 | 79.92 | Insecta terrestrial | 1.88 |  | 3.91594 |
| JFBM48585 | 28 | 70.83 | Cladocera | 0.418 |  | 0.01206 |
| JFBM48585 | 28 | 70.83 | Cladocera | 0.289 | 0.457 | 0.00904 |
| JFBM48585 | 28 | 70.83 | Copepoda | 0.388 |  | 0.0328 |
| JFBM48585 | 28 | 70.83 | Ostracoda | 0.226 | 0.427 | 0.00208 |
| JFBM48585 | 28 | 70.83 | Copepoda | 0.437 | 1.051 | 0.02665 |
| JFBM48585 | 28 | 70.83 | Copepoda | 0.442 |  | 0.0575 |
| JFBM48585 | 28 | 70.83 | Copepoda | 0.415 | 0.815 | 0.01559 |
| JFBM48585 | 28 | 70.83 | Copepoda | 0.445 | 0.691 | 0.01327 |
| JFBM48585 | 28 | 70.83 | Copepoda | 0.363 | 0.838 | 0.01838 |
| JFBM48585 | 28 | 70.83 | Copepoda | 0.316 | 0.76 | 0.01046 |
| JFBM48585 | 28 | 70.83 | Copepoda | 0.498 | 0.845 | 0.03534 |
| JFBM48585 | 28 | 70.83 | Copepoda | 0.368 | 0.631 | 0.00721 |
| JFBM48585 | 28 | 70.83 | Copepoda | 0.475 | 0.847 | 0.02514 |
| JFBM48585 | 28 | 70.83 | Copepoda | 0.518 |  | 0.12503 |
| JFBM48585 | 28 | 70.83 | Copepoda | 0.369 | 0.953 | 0.01242 |
| JFBM48585 | 28 | 70.83 | Copepoda | 0.368 | 0.911 | 0.01693 |
| JFBM48585 | 28 | 70.83 | Copepoda | 0.293 | 0.551 | 0.0056 |
| JFBM48585 | 28 | 70.83 | Copepoda | 0.406 |  | 0.03953 |
| JFBM48585 | 28 | 70.83 | Copepoda | 0.366 |  | 0.02613 |
| JFBM48585 | 28 | 70.83 | Insecta terrestrial | 1.928 |  | 4.03148 |
| JFBM48585 | 28 | 70.83 | Insecta terrestrial | 1.836 |  | 3.81002 |
| JFBM48585 | 29 | 80.38 | Copepoda | 0.313 | 0.784 | 0.00755 |
| JFBM48585 | 29 | 80.38 | Copepoda | 0.389 | 1.006 | 0.01854 |
| JFBM48585 | 29 | 80.38 | Copepoda | 0.283 |  | 0.0111 |
| JFBM48585 | 29 | 80.38 | Copepoda | 0.29 |  | 0.01195 |
| JFBM48585 | 29 | 80.38 | Copepoda | 0.41 | 0.799 | 0.01472 |
| JFBM48585 | 29 | 80.38 | Copepoda | 0.327 | 0.647 | 0.00944 |
| JFBM48585 | 29 | 80.38 | Copepoda | 0.366 |  | 0.02619 |
| JFBM48585 | 29 | 80.38 | Copepoda | 0.545 | 1.293 | 0.06618 |
| JFBM48585 | 29 | 80.38 | Copepoda | 0.363 |  | 0.02538 |
| JFBM48585 | 29 | 80.38 | Copepoda | 0.293 |  | 0.01225 |
| JFBM48585 | 29 | 80.38 | Copepoda | 0.313 |  | 0.01511 |
| JFBM48585 | 29 | 80.38 | Copepoda | 0.268 |  | 0.00954 |
| JFBM48585 | 29 | 80.38 | Copepoda | 0.376 |  | 0.02886 |
| JFBM48585 | 29 | 80.38 | Copepoda | 0.408 | 0.712 | 0.01109 |
| JFBM48585 | 29 | 80.38 | Copepoda | 0.332 |  | 0.01842 |
| JFBM48585 | 29 | 80.38 | Copepoda | 0.417 | 0.777 | 0.01648 |
| JFBM48585 | 29 | 80.38 | Copepoda | 0.444 | 0.959 | 0.01909 |
| JFBM48585 | 29 | 80.38 | Copepoda | 0.334 |  | 0.01872 |
| JFBM48585 | 29 | 80.38 | Copepoda | 0.389 |  | 0.03317 |
| JFBM48585 | 29 | 80.38 | Copepoda | 0.441 |  | 0.05675 |
| JFBM48585 | 29 | 80.38 | Copepoda | 0.448 | 0.888 | 0.02197 |
| JFBM48585 | 29 | 80.38 | Copepoda | 0.464 | 1.062 | 0.0271 |
| JFBM48585 | 29 | 80.38 | Copepoda | 0.492 | 0.864 | 0.02257 |
| JFBM48585 | 29 | 80.38 | Copepoda | 0.253 | 0.725 | 0.00539 |
| JFBM48585 | 29 | 80.38 | Copepoda | 0.317 |  | 0.01583 |
| JFBM48585 | 29 | 80.38 | Egg | 0.199 | 0.309 | 0.00178 |
| JFBM48585 | 29 | 80.38 | Insecta terrestrial | 2.874 |  | 6.30869 |
| JFBM48585 | 29 | 80.38 | Insecta terrestrial | 3.232 |  | 7.17047 |
| JFBM48585 | 29 | 80.38 | Insecta terrestrial | 2.078 |  | 4.39256 |
| JFBM48585 | 29 | 80.38 | Insecta terrestrial | 1.528 |  | 3.0686 |
| JFBM48585 | 29 | 80.38 | Insecta terrestrial | 1.698 |  | 3.47783 |
| JFBM48585 | 30 | 76.7 | Copepoda | 0.456 |  | 0.06643 |
| JFBM48585 | 30 | 76.7 | Copepoda | 0.361 |  | 0.02472 |
| JFBM48585 | 30 | 76.7 | Copepoda | 0.286 | 0.604 | 0.0056 |
| JFBM48585 | 30 | 76.7 | Copepoda | 0.374 | 1.007 | 0.01927 |
| JFBM48585 | 30 | 76.7 | Copepoda | 0.486 | 1.106 | 0.02465 |
| JFBM48585 | 30 | 76.7 | Copepoda | 0.375 |  | 0.02877 |
| JFBM48585 | 30 | 76.7 | Copepoda | 0.389 |  | 0.03308 |
| JFBM48585 | 30 | 76.7 | Copepoda | 0.258 |  | 0.00856 |
| JFBM48585 | 30 | 76.7 | Copepoda | 0.345 |  | 0.02097 |
| JFBM48585 | 30 | 76.7 | Copepoda | 0.31 |  | 0.01462 |
| JFBM48585 | 30 | 76.7 | Copepoda | 0.311 |  | 0.01488 |
| JFBM48585 | 30 | 76.7 | Copepoda | 0.427 | 1.124 | 0.01792 |
| JFBM48585 | 30 | 76.7 | Copepoda | 0.424 | 0.862 | 0.02114 |
| JFBM48585 | 30 | 76.7 | Copepoda | 0.354 |  | 0.02315 |
| JFBM48585 | 30 | 76.7 | Copepoda | 0.312 |  | 0.01501 |
| JFBM48585 | 30 | 76.7 | Copepoda | 0.302 |  | 0.01356 |
| JFBM48585 | 30 | 76.7 | Copepoda | 0.316 |  | 0.01556 |
| JFBM48585 | 30 | 76.7 | Copepoda | 0.471 |  | 0.07755 |
| JFBM48585 | 30 | 76.7 | Copepoda | 0.338 |  | 0.01957 |
| JFBM48585 | 30 | 76.7 | Copepoda | 0.44 |  | 0.05582 |
| JFBM48585 | 30 | 76.7 | Copepoda | 0.437 | 0.949 | 0.01965 |
| JFBM48585 | 30 | 76.7 | Copepoda | 0.404 |  | 0.0386 |
| JFBM48585 | 30 | 76.7 | Copepoda | 0.416 | 1.091 | 0.019 |
| JFBM48585 | 30 | 76.7 | Copepoda | 0.301 | 0.824 | 0.01144 |
| JFBM48585 | 30 | 76.7 | Copepoda | 0.324 |  | 0.01686 |
| JFBM48585 | 30 | 76.7 | Copepoda | 0.516 |  | 0.12316 |
| JFBM48585 | 30 | 76.7 | Copepoda | 0.471 | 1.132 | 0.03212 |
| JFBM48585 | 30 | 76.7 | Copepoda | 0.446 | 0.941 | 0.01634 |
| JFBM48585 | 30 | 76.7 | Copepoda | 0.361 | 0.794 | 0.01274 |
| JFBM48585 | 30 | 76.7 | Copepoda | 0.427 | 1.053 | 0.01962 |
| JFBM48585 | 30 | 76.7 | Copepoda | 0.453 | 1.09 | 0.02616 |
| JFBM48585 | 30 | 76.7 | Copepoda | 0.387 |  | 0.03229 |
| JFBM48585 | 30 | 76.7 | Copepoda | 0.391 |  | 0.03374 |
| JFBM48585 | 30 | 76.7 | Copepoda | 0.503 |  | 0.10704 |
| JFBM48585 | 30 | 76.7 | Copepoda | 0.485 | 0.804 | 0.02175 |
| JFBM48585 | 30 | 76.7 | Copepoda | 0.443 | 0.786 | 0.01918 |
| JFBM48585 | 30 | 76.7 | Copepoda | 0.391 |  | 0.03387 |
| JFBM48585 | 30 | 76.7 | Copepoda | 0.425 |  | 0.04794 |
| JFBM48585 | 30 | 76.7 | Copepoda | 0.354 | 1.114 | 0.02713 |
| JFBM48585 | 30 | 76.7 | Copepoda | 0.38 | 0.985 | 0.02661 |
| JFBM48585 | 30 | 76.7 | Copepoda | 0.395 |  | 0.03526 |
| JFBM48585 | 30 | 76.7 | Copepoda | 0.498 |  | 0.10247 |
| JFBM48585 | 30 | 76.7 | Copepoda | 0.367 | 0.786 | 0.02362 |
| JFBM48585 | 30 | 76.7 | Copepoda | 0.398 | 0.929 | 0.0078 |
| JFBM48585 | 30 | 76.7 | Copepoda | 0.441 | 1.18 | 0.02768 |
| JFBM48585 | 30 | 76.7 | Copepoda | 0.383 | 0.884 | 0.02805 |
| JFBM48585 | 30 | 76.7 | Copepoda | 0.33 |  | 0.01801 |
| JFBM48585 | 30 | 76.7 | Cladocera | 0.577 |  | 0.01918 |
| JFBM48585 | 30 | 76.7 | Copepoda | 0.378 | 0.823 | 0.01536 |
| JFBM48585 | 30 | 76.7 | Copepoda | 0.349 |  | 0.02188 |
| JFBM48585 | 30 | 76.7 | Copepoda | 0.323 | 0.831 | 0.01449 |
| JFBM48585 | 30 | 76.7 | Copepoda | 0.481 |  | 0.08557 |
| JFBM48585 | 30 | 76.7 | Copepoda | 0.407 |  | 0.03989 |
| JFBM48585 | 30 | 76.7 | Insecta terrestrial | 0.753 |  | 1.20334 |
| JFBM48585 | 31 | 81.45 | Copepoda | 0.386 |  | 0.03225 |
| JFBM48585 | 31 | 81.45 | Copepoda | 0.266 |  | 0.00931 |
| JFBM48585 | 31 | 81.45 | Copepoda | 0.297 | 0.756 | 0.00879 |
| JFBM48585 | 31 | 81.45 | Copepoda | 0.346 |  | 0.02125 |
| JFBM48585 | 31 | 81.45 | Copepoda | 0.341 |  | 0.02024 |
| JFBM48585 | 31 | 81.45 | Copepoda | 0.514 |  | 0.1202 |
| JFBM48585 | 31 | 81.45 | Copepoda | 0.326 |  | 0.01726 |
| JFBM48585 | 31 | 81.45 | Copepoda | 0.381 |  | 0.03048 |
| JFBM48585 | 31 | 81.45 | Copepoda | 0.374 |  | 0.02847 |
| JFBM48585 | 31 | 81.45 | Copepoda | 0.204 | 0.652 | 0.00493 |
| JFBM48585 | 31 | 81.45 | Copepoda | 0.269 |  | 0.00959 |
| JFBM48585 | 31 | 81.45 | Copepoda | 0.424 |  | 0.04769 |
| JFBM48585 | 31 | 81.45 | Copepoda | 0.351 | 0.829 | 0.01388 |
| JFBM48585 | 31 | 81.45 | Copepoda | 0.343 | 0.795 | 0.00613 |
| JFBM48585 | 31 | 81.45 | Copepoda | 0.426 | 1.109 | 0.01864 |
| JFBM48585 | 31 | 81.45 | Copepoda | 0.326 | 0.826 | 0.01281 |
| JFBM48585 | 31 | 81.45 | Copepoda | 0.423 |  | 0.04685 |
| JFBM48585 | 31 | 81.45 | Copepoda | 0.404 |  | 0.03857 |
| JFBM48585 | 31 | 81.45 | Copepoda | 0.404 |  | 0.03857 |
| JFBM48585 | 31 | 81.45 | Copepoda | 0.365 |  | 0.02578 |
| JFBM48585 | 31 | 81.45 | Copepoda | 0.496 | 0.84 | 0.0304 |
| JFBM48585 | 31 | 81.45 | Copepoda | 0.467 | 0.757 | 0.02509 |
| JFBM48585 | 31 | 81.45 | Copepoda | 0.473 |  | 0.0785 |
| JFBM48585 | 31 | 81.45 | Copepoda | 0.671 |  | 0.60723 |
| JFBM48585 | 31 | 81.45 | Copepoda | 0.324 | 0.641 | 0.00866 |
| JFBM48585 | 31 | 81.45 | Copepoda | 0.397 |  | 0.03611 |
| JFBM48585 | 31 | 81.45 | Insecta larvae | 0.47 |  | 1.12295 |
| JFBM48585 | 32 | 83.26 | Formicidae | 1.06 |  | 1.94226 |
| JFBM48585 | 32 | 83.26 | Formicidae | 0.968 |  | 1.72163 |
| JFBM48585 | 32 | 83.26 | Insecta terrestrial | 9.231 | 12.002 | 1666.52479 |
| JFBM48585 | 33 | 87.88 | Copepoda | 0.509 |  | 0.11461 |
| JFBM48585 | 33 | 87.88 | Copepoda | 0.228 |  | 0.00628 |
| JFBM48585 | 33 | 87.88 | Copepoda | 0.275 | 0.334 | 0.01502 |
| JFBM48585 | 33 | 87.88 | Copepoda | 0.469 |  | 0.07531 |
| JFBM48585 | 33 | 87.88 | Copepoda | 0.423 | 0.835 | 0.25873 |
| JFBM48585 | 33 | 87.88 | Copepoda | 0.296 |  | 0.01271 |
| JFBM48585 | 33 | 87.88 | Copepoda | 0.444 | 0.787 | 0.19274 |
| JFBM48585 | 33 | 87.88 | Copepoda | 0.534 |  | 0.1474 |
| JFBM48585 | 33 | 87.88 | Copepoda | 0.332 |  | 0.01844 |
| JFBM48585 | 33 | 87.88 | Copepoda | 0.491 | 0.809 | 0.25321 |
| JFBM48585 | 33 | 87.88 | Copepoda | 0.373 |  | 0.02817 |
| JFBM48585 | 33 | 87.88 | Copepoda | 0.391 |  | 0.03393 |
| JFBM48585 | 33 | 87.88 | Copepoda | 0.477 |  | 0.08174 |
| JFBM48585 | 33 | 87.88 | Copepoda | 0.477 |  | 0.08174 |
| JFBM48585 | 33 | 87.88 | Copepoda | 0.347 |  | 0.02148 |
| JFBM48585 | 33 | 87.88 | Copepoda | 0.339 |  | 0.01969 |
| JFBM48585 | 33 | 87.88 | Copepoda | 1.094 |  | 47.81006 |
| JFBM48585 | 33 | 87.88 | Insecta terrestrial (fragments) | 10.912 | 13.352 | 1719.36701 |
| JFBM48585 | 34 | 78.98 | Copepoda | 0.357 |  | 0.02385 |
| JFBM48585 | 34 | 78.98 | Copepoda | 0.291 | 0.836 | 0.01213 |
| JFBM48585 | 34 | 78.98 | Copepoda | 0.369 |  | 0.02683 |
| JFBM48585 | 34 | 78.98 | Copepoda | 0.194 | 0.499 | 0.00132 |
| JFBM48585 | 34 | 78.98 | Copepoda | 0.355 |  | 0.02341 |
| JFBM48585 | 34 | 78.98 | Copepoda | 0.399 |  | 0.03675 |
| JFBM48585 | 34 | 78.98 | Copepoda | 0.351 |  | 0.02245 |
| JFBM48585 | 34 | 78.98 | Copepoda | 0.414 |  | 0.04273 |
| JFBM48585 | 34 | 78.98 | Copepoda | 0.365 |  | 0.02574 |
| JFBM48585 | 34 | 78.98 | Copepoda | 0.534 |  | 0.14776 |
| JFBM48585 | 34 | 78.98 | Copepoda | 0.371 | 1.191 | 0.01871 |
| JFBM48585 | 34 | 78.98 | Copepoda | 0.31 |  | 0.01466 |
| JFBM48585 | 34 | 78.98 | Copepoda | 0.29 | 0.74 | 0.01796 |
| JFBM48585 | 34 | 78.98 | Copepoda | 0.274 |  | 0.01009 |
| JFBM48585 | 34 | 78.98 | Copepoda | 0.409 | 1.078 | 0.03178 |
| JFBM48585 | 34 | 78.98 | Copepoda | 0.405 | 1.07 | 0.02842 |
| JFBM48585 | 34 | 78.98 | Copepoda | 0.405 |  | 0.03908 |
| JFBM48585 | 34 | 78.98 | Copepoda | 0.391 |  | 0.03374 |
| JFBM48585 | 34 | 78.98 | Copepoda | 0.313 | 0.773 | 0.01021 |
| JFBM48585 | 34 | 78.98 | Copepoda | 0.416 | 0.815 | 0.02042 |
| JFBM48585 | 34 | 78.98 | Copepoda | 0.402 | 1.046 | 0.02924 |
| JFBM48585 | 34 | 78.98 | Copepoda | 0.438 | 0.802 | 0.02602 |
| JFBM48585 | 34 | 78.98 | Copepoda | 0.303 |  | 0.01363 |
| JFBM48585 | 34 | 78.98 | Copepoda | 0.36 |  | 0.02454 |
| JFBM48585 | 34 | 78.98 | Copepoda | 0.317 |  | 0.01575 |
| JFBM48585 | 34 | 78.98 | Copepoda | 0.338 |  | 0.01952 |
| JFBM48585 | 34 | 78.98 | Copepoda | 0.41 | 0.999 | 0.02843 |
| JFBM48585 | 34 | 78.98 | Plant | 0.155 | 2.508 | 0.00483 |
| JFBM48585 | 34 | 78.98 | Copepoda | 0.301 | 1.053 | 0.02526 |
| JFBM48585 | 34 | 78.98 | Copepoda | 0.267 | 0.843 | 0.01396 |
| JFBM48585 | 34 | 78.98 | Copepoda | 0.397 | 1.106 | 0.0242 |
| JFBM48585 | 34 | 78.98 | Copepoda | 0.413 |  | 0.04257 |
| JFBM48585 | 34 | 78.98 | Copepoda | 0.349 |  | 0.02188 |
| JFBM48585 | 34 | 78.98 | Copepoda | 0.403 | 1.097 | 0.026 |
| JFBM48585 | 34 | 78.98 | Copepoda | 0.369 | 0.959 | 0.02715 |
| JFBM48585 | 34 | 78.98 | Copepoda | 0.33 |  | 0.01801 |
| JFBM48585 | 34 | 78.98 | Copepoda | 0.421 | 0.865 | 0.00775 |
| JFBM48585 | 34 | 78.98 | Copepoda | 0.327 | 0.789 | 0.00531 |
| JFBM48585 | 34 | 78.98 | Copepoda | 0.348 |  | 0.02166 |
| JFBM48585 | 34 | 78.98 | Copepoda | 0.364 | 0.917 | 0.01088 |
| JFBM48585 | 34 | 78.98 | Copepoda | 0.402 | 1.068 | 0.0225 |
| JFBM48585 | 34 | 78.98 | Copepoda | 0.423 | 0.779 | 0.02018 |
| JFBM48585 | 34 | 78.98 | Copepoda | 0.314 |  | 0.01532 |
| JFBM48585 | 34 | 78.98 | Copepoda | 0.457 | 0.893 | 0.0225 |
| JFBM48585 | 34 | 78.98 | Copepoda | 0.417 | 0.914 | 0.02535 |
| JFBM48585 | 34 | 78.98 | Copepoda | 0.269 | 0.75 | 0.0138 |
| JFBM48585 | 34 | 78.98 | Copepoda | 0.383 | 0.702 | 0.0136 |
| JFBM48585 | 34 | 78.98 | Copepoda | 0.441 | 0.861 | 0.01811 |
| JFBM48585 | 34 | 78.98 | Copepoda | 0.295 | 0.492 | 0.00576 |
| JFBM48585 | 34 | 78.98 | Copepoda | 0.536 |  | 0.15096 |
| JFBM48585 | 35 | 77.02 | Copepoda | 0.305 | 0.88 | 0.01564 |
| JFBM48585 | 35 | 77.02 | Copepoda | 0.304 |  | 0.01375 |
| JFBM48585 | 35 | 77.02 | Copepoda | 0.352 |  | 0.02258 |
| JFBM48585 | 35 | 77.02 | Copepoda | 0.282 |  | 0.01101 |
| JFBM48585 | 35 | 77.02 | Copepoda | 0.379 | 0.901 | 0.02029 |
| JFBM48585 | 35 | 77.02 | Copepoda | 0.33 |  | 0.018 |
| JFBM48585 | 35 | 77.02 | Copepoda | 0.411 | 0.919 | 0.02057 |
| JFBM48585 | 35 | 77.02 | Copepoda | 0.329 | 0.852 | 0.0127 |
| JFBM48585 | 35 | 77.02 | Copepoda | 0.237 |  | 0.00689 |
| JFBM48585 | 35 | 77.02 | Copepoda | 0.332 | 0.811 | 0.01026 |
| JFBM48585 | 35 | 77.02 | Copepoda | 0.421 |  | 0.04593 |
| JFBM48585 | 35 | 77.02 | Copepoda | 0.346 |  | 0.02131 |
| JFBM48585 | 35 | 77.02 | Copepoda | 0.323 |  | 0.01668 |
| JFBM48585 | 35 | 77.02 | Copepoda | 0.275 |  | 0.01026 |
| JFBM48585 | 35 | 77.02 | Copepoda | 0.309 |  | 0.01447 |
| JFBM48585 | 35 | 77.02 | Copepoda | 0.377 | 0.693 | 0.01684 |
| JFBM48585 | 35 | 77.02 | Copepoda | 0.457 |  | 0.06687 |
| JFBM48585 | 35 | 77.02 | Copepoda | 0.323 |  | 0.01672 |
| JFBM48585 | 35 | 77.02 | Copepoda | 0.362 | 1.078 | 0.02072 |
| JFBM48585 | 35 | 77.02 | Copepoda | 0.213 |  | 0.00541 |
| JFBM48585 | 35 | 77.02 | Copepoda | 0.256 |  | 0.00841 |
| JFBM48585 | 35 | 77.02 | Copepoda | 0.369 |  | 0.02685 |
| JFBM48585 | 35 | 77.02 | Copepoda | 0.387 | 1.055 | 0.02501 |
| JFBM48585 | 35 | 77.02 | Copepoda | 0.429 |  | 0.04998 |
| JFBM48585 | 35 | 77.02 | Copepoda | 0.444 |  | 0.05813 |
| JFBM48585 | 35 | 77.02 | Copepoda | 0.4 |  | 0.0371 |
| JFBM48585 | 35 | 77.02 | Copepoda | 0.416 |  | 0.0437 |
| JFBM48585 | 35 | 77.02 | Copepoda | 0.238 |  | 0.00695 |
| JFBM48585 | 35 | 77.02 | Copepoda | 0.274 | 0.852 | 0.02516 |
| JFBM48585 | 35 | 77.02 | Cladocera | 0.378 | 0.471 | 0.01099 |
| JFBM48585 | 35 | 77.02 | Copepoda | 0.323 |  | 0.01676 |
| JFBM48585 | 35 | 77.02 | Copepoda | 0.363 |  | 0.02545 |
| JFBM48585 | 35 | 77.02 | Copepoda | 0.43 | 0.775 | 0.01287 |
| JFBM48585 | 35 | 77.02 | Copepoda | 0.345 |  | 0.02111 |
| JFBM48585 | 35 | 77.02 | Copepoda | 0.279 |  | 0.01065 |
| JFBM48585 | 35 | 77.02 | Copepoda | 0.504 |  | 0.10823 |
| JFBM48585 | 35 | 77.02 | Copepoda | 0.333 |  | 0.01858 |
| JFBM48585 | 35 | 77.02 | Copepoda | 0.463 | 0.93 | 0.02365 |
| JFBM48585 | 35 | 77.02 | Copepoda | 0.431 | 0.85 | 0.02222 |
| JFBM48585 | 35 | 77.02 | Copepoda | 0.355 | 0.769 | 0.01325 |
| JFBM48585 | 35 | 77.02 | Copepoda | 0.445 | 1.109 | 0.02554 |
| JFBM48585 | 35 | 77.02 | Copepoda | 0.296 |  | 0.0127 |
| JFBM48585 | 35 | 77.02 | Copepoda | 0.408 |  | 0.0403 |
| JFBM48585 | 35 | 77.02 | Copepoda | 0.344 |  | 0.02084 |
| JFBM48585 | 35 | 77.02 | Copepoda | 0.283 |  | 0.0111 |
| JFBM48585 | 35 | 77.02 | Copepoda | 0.397 |  | 0.03586 |
| JFBM48585 | 35 | 77.02 | Copepoda | 0.275 | 0.684 | 0.00938 |
| JFBM48585 | 35 | 77.02 | Copepoda | 0.287 |  | 0.01152 |
| JFBM48585 | 35 | 77.02 | Copepoda | 0.43 | 0.998 | 0.01502 |
| JFBM48585 | 35 | 77.02 | Copepoda | 0.281 |  | 0.01087 |
| JFBM48585 | 35 | 77.02 | Copepoda | 0.33 | 1.047 | 0.02072 |
| JFBM48585 | 35 | 77.02 | Copepoda | 0.369 |  | 0.02691 |
| JFBM48585 | 35 | 77.02 | Copepoda | 0.436 | 1.109 | 0.024 |
| JFBM48585 | 35 | 77.02 | Copepoda | 0.454 |  | 0.06462 |
| JFBM48585 | 35 | 77.02 | Copepoda | 0.257 |  | 0.00848 |
| JFBM48585 | 35 | 77.02 | Copepoda | 0.382 | 1.075 | 0.01393 |
| JFBM48585 | 35 | 77.02 | Copepoda | 0.307 |  | 0.01418 |
| JFBM48585 | 35 | 77.02 | Copepoda | 0.392 |  | 0.0341 |
| JFBM48585 | 35 | 77.02 | Copepoda | 0.338 | 0.775 | 0.01012 |
| JFBM48585 | 35 | 77.02 | Copepoda | 0.38 |  | 0.0303 |
| JFBM48585 | 35 | 77.02 | Copepoda | 0.343 | 0.829 | 0.01433 |
| JFBM48585 | 35 | 77.02 | Copepoda | 0.309 | 0.764 | 0.01153 |
| JFBM48585 | 36 | 77.46 | Copepoda | 0.34 | 0.734 | 0.01847 |
| JFBM48585 | 36 | 77.46 | Copepoda | 0.353 |  | 0.02288 |
| JFBM48585 | 36 | 77.46 | Copepoda | 0.406 |  | 0.03958 |
| JFBM48585 | 36 | 77.46 | Copepoda | 0.229 |  | 0.00638 |
| JFBM48585 | 36 | 77.46 | Copepoda | 0.455 |  | 0.06554 |
| JFBM48585 | 36 | 77.46 | Copepoda | 0.466 |  | 0.07346 |
| JFBM48585 | 36 | 77.46 | Copepoda | 0.492 |  | 0.09577 |
| JFBM48585 | 37 | 81.85 | Copepoda | 0.452 | 0.798 | 0.03115 |
| JFBM48585 | 37 | 81.85 | Copepoda | 0.51 | 0.831 | 0.02542 |
| JFBM48585 | 37 | 81.85 | Copepoda | 0.41 |  | 0.04108 |
| JFBM48585 | 37 | 81.85 | Copepoda | 0.253 | 0.7 | 0.00724 |
| JFBM48585 | 37 | 81.85 | Copepoda | 0.492 | 0.91 | 0.0226 |
| JFBM48585 | 37 | 81.85 | Copepoda | 0.453 | 0.799 | 0.0251 |
| JFBM48585 | 37 | 81.85 | Copepoda | 0.41 | 1.012 | 0.01958 |
| JFBM48585 | 37 | 81.85 | Copepoda | 0.41 | 0.955 | 0.02176 |
| JFBM48585 | 37 | 81.85 | Copepoda | 0.3 | 0.61 | 0.00996 |
| JFBM48585 | 37 | 81.85 | Copepoda | 0.46 | 0.789 | 0.02178 |
| JFBM48585 | 37 | 81.85 | Copepoda | 0.397 |  | 0.03586 |
| JFBM48585 | 37 | 81.85 | Copepoda | 0.401 | 0.674 | 0.01919 |
| JFBM48585 | 37 | 81.85 | Copepoda | 0.368 | 0.722 | 0.01461 |
| JFBM48585 | 37 | 81.85 | Copepoda | 0.249 |  | 0.00783 |
| JFBM48585 | 37 | 81.85 | Copepoda | 0.374 | 0.867 | 0.02969 |
| JFBM48585 | 37 | 81.85 | Copepoda | 0.235 |  | 0.00674 |
| JFBM48585 | 37 | 81.85 | Copepoda | 0.435 | 0.991 | 0.02591 |
| JFBM48585 | 37 | 81.85 | Copepoda | 0.237 | 0.662 | 0.00406 |
| JFBM48585 | 37 | 81.85 | Copepoda | 0.375 |  | 0.02868 |
| JFBM48585 | 37 | 81.85 | Copepoda | 0.316 | 1.168 | 0.01985 |
| JFBM48585 | 37 | 81.85 | Copepoda | 0.351 | 1.041 | 0.01759 |
| JFBM48585 | 37 | 81.85 | Copepoda | 0.358 |  | 0.02405 |
| JFBM48585 | 37 | 81.85 | Copepoda | 0.499 |  | 0.10348 |
| JFBM48585 | 37 | 81.85 | Copepoda | 0.421 | 0.876 | 0.01926 |
| JFBM48585 | 37 | 81.85 | Copepoda | 0.294 |  | 0.01238 |
| JFBM48585 | 37 | 81.85 | Copepoda | 0.321 | 0.766 | 0.01431 |
| JFBM48585 | 37 | 81.85 | Copepoda | 0.303 | 0.653 | 0.00679 |
| JFBM48585 | 37 | 81.85 | Copepoda | 0.431 | 0.898 | 0.02342 |
| JFBM48585 | 37 | 81.85 | Copepoda | 0.395 | 0.79 | 0.01447 |
| JFBM48585 | 37 | 81.85 | Copepoda | 0.355 | 0.764 | 0.01864 |
| JFBM48585 | 37 | 81.85 | Copepoda | 0.432 | 0.864 | 0.01713 |
| JFBM48585 | 37 | 81.85 | Copepoda | 0.399 |  | 0.03679 |
| JFBM48585 | 37 | 81.85 | Copepoda | 0.344 |  | 0.02082 |
| JFBM48585 | 37 | 81.85 | Copepoda | 0.32 | 1.003 | 0.01751 |
| JFBM48585 | 37 | 81.85 | Copepoda | 0.375 |  | 0.02868 |
| JFBM48585 | 37 | 81.85 | Copepoda | 0.369 |  | 0.02685 |
| JFBM48585 | 37 | 81.85 | Copepoda | 0.353 | 0.644 | 0.00667 |
| JFBM48585 | 37 | 81.85 | Copepoda | 0.307 |  | 0.01416 |
| JFBM48585 | 37 | 81.85 | Copepoda | 0.276 | 0.549 | 0.00501 |
| JFBM48585 | 37 | 81.85 | Copepoda | 0.382 | 1.082 | 0.01923 |
| JFBM48585 | 37 | 81.85 | Copepoda | 0.448 | 1.166 | 0.02143 |
| JFBM48585 | 37 | 81.85 | Copepoda | 0.286 |  | 0.0114 |
| JFBM48585 | 37 | 81.85 | Copepoda | 0.369 | 1.055 | 0.02526 |
| JFBM48585 | 37 | 81.85 | Copepoda | 0.274 |  | 0.01012 |
| JFBM48585 | 37 | 81.85 | Hymenoptera terrestrial | 0.954 |  | 1.68748 |
| JFBM48585 | 37 | 81.85 | Copepoda | 0.381 |  | 0.0304 |
| JFBM48585 | 37 | 81.85 | Copepoda | 0.351 | 0.779 | 0.01193 |
| JFBM48585 | 37 | 81.85 | Copepoda | 0.365 |  | 0.02574 |
| JFBM48585 | 37 | 81.85 | Hymenoptera terrestrial | 2.888 | 8.338 | 9.3649 |
| JFBM48585 | 37 | 81.85 | Hymenoptera terrestrial | 2.008 |  | 4.22406 |
| JFBM48585 | 37 | 81.85 | Hymenoptera terrestrial | 2.95 | 9.508 | 7.41106 |
| JFBM48585 | 37 | 81.85 | Hymenoptera terrestrial | 2.394 |  | 5.15324 |
| JFBM48585 | 37 | 81.85 | Hymenoptera terrestrial | 1.92 |  | 4.01222 |
| JFBM48585 | 37 | 81.85 | Hymenoptera terrestrial | 1.968 |  | 4.12777 |
| JFBM48585 | 37 | 81.85 | Hymenoptera terrestrial | 1.702 |  | 3.48745 |
| JFBM48585 | 37 | 81.85 | Hymenoptera terrestrial | 1.872 |  | 3.89668 |
| JFBM48585 | 37 | 81.85 | Hymenoptera terrestrial | 1.92 |  | 4.01222 |
| JFBM48585 | 37 | 81.85 | Hymenoptera terrestrial | 1.702 |  | 3.48745 |
| JFBM48585 | 37 | 81.85 | Hymenoptera terrestrial | 2.256 |  | 4.82104 |
| JFBM48585 | 37 | 81.85 | Hymenoptera terrestrial | 1.914 |  | 3.99778 |
| JFBM48585 | 38 | 80.65 | Copepoda | 0.484 |  | 0.08838 |
| JFBM48585 | 38 | 80.65 | Copepoda | 0.325 |  | 0.01719 |
| JFBM48585 | 38 | 80.65 | Copepoda | 0.363 | 1.067 | 0.01439 |
| JFBM48585 | 38 | 80.65 | Copepoda | 0.476 |  | 0.08137 |
| JFBM48585 | 38 | 80.65 | Copepoda | 0.277 | 0.756 | 0.0083 |
| JFBM48585 | 38 | 80.65 | Copepoda | 0.444 |  | 0.05867 |
| JFBM48585 | 38 | 80.65 | Copepoda | 0.472 |  | 0.07831 |
| JFBM48585 | 38 | 80.65 | Copepoda | 0.264 | 0.895 | 0.00826 |
| JFBM48585 | 38 | 80.65 | Copepoda | 0.382 |  | 0.03066 |
| JFBM48585 | 38 | 80.65 | Copepoda | 0.379 |  | 0.02978 |
| JFBM48585 | 38 | 80.65 | Copepoda | 0.396 |  | 0.03578 |
| JFBM48585 | 38 | 80.65 | Copepoda | 0.47 |  | 0.07671 |
| JFBM48585 | 38 | 80.65 | Copepoda | 0.375 |  | 0.02857 |
| JFBM48585 | 39 | 77.67 | Copepoda | 0.245 | 0.602 | 0.00499 |
| JFBM48585 | 39 | 77.67 | Copepoda | 0.385 |  | 0.03162 |
| JFBM48585 | 39 | 77.67 | Copepoda | 0.394 | 0.806 | 0.00961 |
| JFBM48585 | 39 | 77.67 | Copepoda | 0.246 |  | 0.00757 |
| JFBM48585 | 39 | 77.67 | Copepoda | 0.41 | 1.151 | 0.021 |
| JFBM48585 | 39 | 77.67 | Copepoda | 0.319 | 0.877 | 0.01406 |
| JFBM48585 | 39 | 77.67 | Copepoda | 0.375 |  | 0.02872 |
| JFBM48585 | 39 | 77.67 | Copepoda | 0.389 |  | 0.03315 |
| JFBM48585 | 39 | 77.67 | Copepoda | 0.242 |  | 0.0073 |
| JFBM48585 | 39 | 77.67 | Copepoda | 0.381 |  | 0.03042 |
| JFBM48585 | 39 | 77.67 | Copepoda | 0.484 | 0.907 | 0.02836 |
| JFBM48585 | 39 | 77.67 | Copepoda | 0.426 |  | 0.04848 |
| JFBM48585 | 39 | 77.67 | Copepoda | 0.414 | 0.865 | 0.01582 |
| JFBM48585 | 39 | 77.67 | Copepoda | 0.427 |  | 0.04893 |
| JFBM48585 | 39 | 77.67 | Copepoda | 0.316 |  | 0.01557 |
| JFBM48585 | 39 | 77.67 | Copepoda | 0.48 |  | 0.08432 |
| JFBM48585 | 39 | 77.67 | Copepoda | 0.206 | 0.588 | 0.00271 |
| JFBM48585 | 39 | 77.67 | Copepoda | 0.314 |  | 0.01531 |
| JFBM48585 | 39 | 77.67 | Copepoda | 0.333 | 0.639 | 0.01522 |
| JFBM48585 | 39 | 77.67 | Copepoda | 0.284 |  | 0.01117 |
| JFBM48585 | 39 | 77.67 | Copepoda | 0.29 |  | 0.01198 |
| JFBM48585 | 39 | 77.67 | Copepoda | 0.312 |  | 0.01501 |
| JFBM48585 | 39 | 77.67 | Copepoda | 0.235 |  | 0.00679 |
| JFBM48585 | 39 | 77.67 | Copepoda | 0.378 | 0.835 | 0.01424 |
| JFBM48585 | 39 | 77.67 | Copepoda | 0.344 | 0.984 | 0.01456 |
| JFBM48585 | 39 | 77.67 | Copepoda | 0.445 |  | 0.05897 |
| JFBM48585 | 39 | 77.67 | Copepoda | 0.49 |  | 0.09342 |
| JFBM48585 | 39 | 77.67 | Copepoda | 0.412 |  | 0.04186 |
| JFBM48585 | 39 | 77.67 | Copepoda | 0.475 | 0.84 | 0.02526 |
| JFBM48585 | 39 | 77.67 | Copepoda | 0.433 | 0.797 | 0.01849 |
| JFBM48585 | 39 | 77.67 | Copepoda | 0.467 | 1.008 | 0.02518 |
| JFBM48585 | 39 | 77.67 | Copepoda | 0.337 |  | 0.01945 |
| JFBM48585 | 39 | 77.67 | Copepoda | 0.492 |  | 0.09631 |
| JFBM48585 | 39 | 77.67 | Copepoda | 0.367 |  | 0.02627 |
| JFBM48585 | 39 | 77.67 | Copepoda | 0.483 |  | 0.08717 |
| JFBM48585 | 39 | 77.67 | Copepoda | 0.466 |  | 0.07294 |
| JFBM48585 | 39 | 77.67 | Copepoda | 0.355 |  | 0.02328 |
| JFBM48585 | 39 | 77.67 | Copepoda | 0.469 | 0.948 | 0.02329 |
| JFBM48585 | 39 | 77.67 | Copepoda | 0.496 |  | 0.10018 |
| JFBM48585 | 39 | 77.67 | Copepoda | 0.37 | 0.845 | 0.01498 |
| JFBM48585 | 39 | 77.67 | Copepoda | 0.31 |  | 0.01462 |
| JFBM48585 | 39 | 77.67 | Copepoda | 0.504 |  | 0.10823 |
| JFBM48585 | 39 | 77.67 | Copepoda | 0.299 |  | 0.01305 |
| JFBM48585 | 39 | 77.67 | Copepoda | 0.439 |  | 0.05537 |
| JFBM48585 | 39 | 77.67 | Copepoda | 0.399 | 0.838 | 0.01382 |
| JFBM48585 | 39 | 77.67 | Copepoda | 0.336 |  | 0.01914 |
| JFBM48585 | 39 | 77.67 | Copepoda | 0.342 | 0.861 | 0.01644 |
| JFBM48585 | 39 | 77.67 | Copepoda | 0.454 |  | 0.0651 |
| JFBM48585 | 39 | 77.67 | Copepoda | 0.376 |  | 0.02898 |
| JFBM48585 | 39 | 77.67 | Copepoda | 0.547 |  | 0.16848 |
| JFBM48585 | 39 | 77.67 | Copepoda | 0.48 | 1.332 | 0.03684 |
| JFBM48585 | 39 | 77.67 | Copepoda | 0.33 |  | 0.01796 |
| JFBM48585 | 39 | 77.67 | Copepoda | 0.507 |  | 0.11236 |
| JFBM48585 | 39 | 77.67 | Copepoda | 0.343 |  | 0.0207 |
| JFBM48585 | 39 | 77.67 | Copepoda | 0.284 |  | 0.01118 |
| JFBM48585 | 39 | 77.67 | Copepoda | 0.39 |  | 0.03349 |
| JFBM48585 | 39 | 77.67 | Copepoda | 0.352 |  | 0.0225 |
| JFBM48585 | 39 | 77.67 | Copepoda | 0.307 | 0.716 | 0.019 |
| JFBM48585 | 39 | 77.67 | Copepoda | 0.335 |  | 0.01895 |
| JFBM48585 | 39 | 77.67 | Copepoda | 0.338 |  | 0.01965 |
| JFBM48585 | 39 | 77.67 | Copepoda | 0.332 |  | 0.01844 |
| JFBM48585 | 39 | 77.67 | Copepoda | 0.387 |  | 0.0324 |
| JFBM48585 | 39 | 77.67 | Copepoda | 0.363 |  | 0.02525 |
| JFBM48585 | 39 | 77.67 | Copepoda | 0.388 |  | 0.0329 |
| JFBM48585 | 39 | 77.67 | Copepoda | 0.358 | 0.91 | 0.0168 |
| JFBM48585 | 39 | 77.67 | Copepoda | 0.354 |  | 0.02315 |
| JFBM48585 | 39 | 77.67 | Copepoda | 0.367 | 1.035 | 0.02037 |
| JFBM48585 | 39 | 77.67 | Copepoda | 0.307 | 0.858 | 0.01302 |
| JFBM48585 | 39 | 77.67 | Copepoda | 0.458 | 0.925 | 0.02377 |
| JFBM48585 | 39 | 77.67 | Copepoda | 0.364 |  | 0.02572 |
| JFBM48585 | 39 | 77.67 | Copepoda | 0.264 |  | 0.00916 |
| JFBM48585 | 40 | 74.74 | Copepoda | 0.36 | 0.748 | 0.01513 |
| JFBM48585 | 40 | 74.74 | Copepoda | 0.359 | 0.864 | 0.01102 |
| JFBM48585 | 40 | 74.74 | Copepoda | 0.291 |  | 0.0121 |
| JFBM48585 | 40 | 74.74 | Copepoda | 0.336 |  | 0.01926 |
| JFBM48585 | 40 | 74.74 | Copepoda | 0.47 |  | 0.07631 |
| JFBM48585 | 40 | 74.74 | Copepoda | 0.301 |  | 0.01339 |
| JFBM48585 | 40 | 74.74 | Copepoda | 0.467 |  | 0.07377 |
| JFBM48585 | 40 | 74.74 | Copepoda | 0.531 |  | 0.1435 |
| JFBM48585 | 40 | 74.74 | Copepoda | 0.296 |  | 0.01268 |
| JFBM48585 | 40 | 74.74 | Copepoda | 0.264 |  | 0.00915 |
| JFBM48585 | 40 | 74.74 | Copepoda | 0.442 |  | 0.05698 |
| JFBM48585 | 40 | 74.74 | Copepoda | 0.313 |  | 0.01505 |
| JFBM48585 | 40 | 74.74 | Copepoda | 0.51 |  | 0.11502 |
| JFBM48585 | 40 | 74.74 | Copepoda | 0.418 | 0.999 | 0.01975 |
| JFBM48585 | 40 | 74.74 | Copepoda | 0.355 |  | 0.02341 |
| JFBM48585 | 40 | 74.74 | Copepoda | 0.311 |  | 0.01479 |
| JFBM48585 | 40 | 74.74 | Copepoda | 0.364 |  | 0.02572 |
| JFBM48585 | 40 | 74.74 | Copepoda | 0.452 |  | 0.06321 |
| JFBM48585 | 40 | 74.74 | Copepoda | 0.389 |  | 0.03296 |
| JFBM48585 | 40 | 74.74 | Copepoda | 0.379 | 0.907 | 0.02449 |
| JFBM48585 | 40 | 74.74 | Copepoda | 0.346 | 0.796 | 0.01916 |
| JFBM48585 | 40 | 74.74 | Copepoda | 0.483 |  | 0.08717 |
| JFBM48585 | 40 | 74.74 | Copepoda | 0.414 |  | 0.04274 |
| JFBM48585 | 40 | 74.74 | Copepoda | 0.491 | 0.863 | 0.0166 |
| JFBM48585 | 40 | 74.74 | Copepoda | 0.561 |  | 0.19634 |
| JFBM48585 | 40 | 74.74 | Copepoda | 0.369 | 0.845 | 0.01349 |
| JFBM48585 | 40 | 74.74 | Copepoda | 0.479 |  | 0.08361 |
| JFBM48585 | 40 | 74.74 | Copepoda | 0.424 |  | 0.04769 |
| JFBM48585 | 40 | 74.74 | Copepoda | 0.382 |  | 0.03082 |
| JFBM48585 | 40 | 74.74 | Copepoda | 0.426 |  | 0.04841 |
| JFBM48585 | 40 | 74.74 | Copepoda | 0.423 |  | 0.04706 |
| JFBM48585 | 40 | 74.74 | Copepoda | 0.579 |  | 0.23561 |
| JFBM48585 | 40 | 74.74 | Copepoda | 0.476 | 0.869 | 0.02818 |
| JFBM48585 | 40 | 74.74 | Copepoda | 0.435 |  | 0.05316 |
| JFBM48585 | 40 | 74.74 | Copepoda | 0.511 |  | 0.11692 |
| JFBM48585 | 40 | 74.74 | Copepoda | 0.456 |  | 0.06643 |
| JFBM48585 | 40 | 74.74 | Copepoda | 0.283 |  | 0.01113 |
| JFBM48585 | 40 | 74.74 | Copepoda | 0.364 | 0.825 | 0.02249 |
| JFBM48585 | 40 | 74.74 | Copepoda | 0.398 | 0.838 | 0.02006 |
| JFBM48585 | 40 | 74.74 | Copepoda | 0.426 | 0.854 | 0.02215 |
| JFBM48585 | 40 | 74.74 | Copepoda | 0.413 |  | 0.04231 |
| JFBM48585 | 40 | 74.74 | Copepoda | 0.306 |  | 0.01411 |
| JFBM48585 | 40 | 74.74 | Copepoda | 0.316 |  | 0.0156 |
| JFBM48585 | 40 | 74.74 | Copepoda | 0.368 |  | 0.02659 |
| JFBM48585 | 40 | 74.74 | Copepoda | 0.345 |  | 0.02103 |
| JFBM48585 | 41 | 83.01 | Copepoda | 0.443 |  | 0.05768 |
| JFBM48585 | 41 | 83.01 | Copepoda | 0.412 | 0.851 | 0.02749 |
| JFBM48585 | 41 | 83.01 | Copepoda | 0.442 |  | 0.05726 |
| JFBM48585 | 41 | 83.01 | Copepoda | 0.362 |  | 0.02511 |
| JFBM48585 | 41 | 83.01 | Copepoda | 0.491 |  | 0.09464 |
| JFBM48585 | 41 | 83.01 | Copepoda | 0.33 |  | 0.01801 |
| JFBM48585 | 41 | 83.01 | Copepoda | 0.276 | 0.857 | 0.00586 |
| JFBM48585 | 41 | 83.01 | Copepoda | 0.435 | 0.756 | 0.01939 |
| JFBM48585 | 41 | 83.01 | Copepoda | 0.342 |  | 0.02044 |
| JFBM48585 | 41 | 83.01 | Copepoda | 0.353 |  | 0.02288 |
| JFBM48585 | 41 | 83.01 | Copepoda | 0.39 |  | 0.03334 |
| JFBM48585 | 41 | 83.01 | Copepoda | 0.316 | 0.816 | 0.01078 |
| JFBM48585 | 41 | 83.01 | Copepoda | 0.341 |  | 0.02011 |
| JFBM48585 | 41 | 83.01 | Copepoda | 0.333 | 0.836 | 0.01876 |
| JFBM48585 | 41 | 83.01 | Copepoda | 0.382 | 0.852 | 0.02619 |
| JFBM48585 | 41 | 83.01 | Copepoda | 0.343 |  | 0.02055 |
| JFBM48585 | 41 | 83.01 | Copepoda | 0.279 |  | 0.01061 |
| JFBM48585 | 41 | 83.01 | Copepoda | 0.45 | 0.924 | 0.02355 |
| JFBM48585 | 41 | 83.01 | Copepoda | 0.297 |  | 0.01282 |
| JFBM48585 | 41 | 83.01 | Copepoda | 0.352 | 0.891 | 0.01463 |
| JFBM48585 | 41 | 83.01 | Copepoda | 0.422 | 1.14 | 0.03079 |
| JFBM48585 | 41 | 83.01 | Copepoda | 0.362 | 0.947 | 0.0116 |
| JFBM48585 | 41 | 83.01 | Copepoda | 0.345 | 0.91 | 0.01861 |
| JFBM48585 | 41 | 83.01 | Copepoda | 0.411 | 1.028 | 0.02101 |
| JFBM48585 | 41 | 83.01 | Copepoda | 0.337 |  | 0.01946 |
| JFBM48585 | 41 | 83.01 | Copepoda | 0.358 | 0.882 | 0.01399 |
| JFBM48585 | 41 | 83.01 | Copepoda | 0.342 |  | 0.02049 |
| JFBM48585 | 41 | 83.01 | Copepoda | 0.337 |  | 0.01946 |
| JFBM48585 | 41 | 83.01 | Copepoda | 0.438 |  | 0.05492 |
| JFBM48585 | 41 | 83.01 | Copepoda | 0.365 |  | 0.02574 |
| JFBM48585 | 41 | 83.01 | Copepoda | 0.359 | 1.049 | 0.02476 |
| JFBM48585 | 41 | 83.01 | Copepoda | 0.346 |  | 0.02134 |
| JFBM48585 | 41 | 83.01 | Copepoda | 0.362 |  | 0.02498 |
| JFBM48585 | 41 | 83.01 | Copepoda | 0.46 |  | 0.06911 |
| JFBM48585 | 41 | 83.01 | Copepoda | 0.251 |  | 0.00795 |
| JFBM48585 | 41 | 83.01 | Copepoda | 0.379 |  | 0.02987 |
| JFBM48585 | 41 | 83.01 | Copepoda | 0.473 | 0.915 | 0.02742 |
| JFBM48585 | 41 | 83.01 | Copepoda | 0.295 | 0.954 | 0.01301 |
| JFBM48585 | 41 | 83.01 | Copepoda | 0.404 |  | 0.0386 |
| JFBM48585 | 41 | 83.01 | Copepoda | 0.318 |  | 0.01587 |
| JFBM48585 | 41 | 83.01 | Copepoda | 0.372 |  | 0.02784 |
| JFBM48585 | 41 | 83.01 | Copepoda | 0.388 |  | 0.03268 |
| JFBM48585 | 41 | 83.01 | Copepoda | 0.352 | 0.992 | 0.02259 |
| JFBM48585 | 41 | 83.01 | Copepoda | 0.367 |  | 0.02641 |
| JFBM48585 | 41 | 83.01 | Copepoda | 0.313 | 0.901 | 0.01568 |
| JFBM48585 | 41 | 83.01 | Copepoda | 0.411 |  | 0.04172 |
| JFBM48585 | 41 | 83.01 | Copepoda | 0.496 | 0.955 | 0.03762 |
| JFBM48585 | 41 | 83.01 | Copepoda | 0.393 |  | 0.03469 |
| JFBM48585 | 41 | 83.01 | Copepoda | 0.397 | 1.043 | 0.01441 |
| JFBM48585 | 41 | 83.01 | Copepoda | 0.373 | 0.916 | 0.01787 |
| JFBM48585 | 41 | 83.01 | Copepoda | 0.374 | 1.077 | 0.02036 |
| JFBM48585 | 41 | 83.01 | Copepoda | 0.323 |  | 0.01676 |
| JFBM48585 | 41 | 83.01 | Copepoda | 0.329 |  | 0.01784 |
| JFBM48585 | 41 | 83.01 | Copepoda | 0.33 |  | 0.01808 |
| JFBM48585 | 41 | 83.01 | Copepoda | 0.446 | 1.08 | 0.02782 |
| JFBM48585 | 41 | 83.01 | Copepoda | 0.455 |  | 0.06554 |
| JFBM48585 | 41 | 83.01 | Copepoda | 0.366 | 0.794 | 0.01346 |
| JFBM48585 | 41 | 83.01 | Copepoda | 0.272 |  | 0.00988 |
| JFBM48585 | 41 | 83.01 | Copepoda | 0.463 |  | 0.07087 |
| JFBM48585 | 41 | 83.01 | Copepoda | 0.372 |  | 0.02771 |
| JFBM48585 | 41 | 83.01 | Copepoda | 0.239 | 0.789 | 0.00513 |
| JFBM48585 | 41 | 83.01 | Copepoda | 0.311 | 1.103 | 0.01451 |
| JFBM48585 | 41 | 83.01 | Copepoda | 0.345 |  | 0.02106 |
| JFBM48585 | 41 | 83.01 | Copepoda | 0.321 | 0.851 | 0.00971 |
| JFBM48585 | 41 | 83.01 | Copepoda | 0.32 |  | 0.01634 |
| JFBM48585 | 41 | 83.01 | Copepoda | 0.307 |  | 0.01416 |
| JFBM48585 | 41 | 83.01 | Copepoda | 0.368 |  | 0.02659 |
| JFBM48585 | 41 | 83.01 | Cladocera | 0.697 |  | 0.7943 |
| JFBM48585 | 41 | 83.01 | Copepoda | 0.375 | 0.898 | 0.01478 |
| JFBM48585 | 41 | 83.01 | Copepoda | 0.35 |  | 0.02214 |
| JFBM48585 | 41 | 83.01 | Copepoda | 0.404 | 0.889 | 0.01328 |
| JFBM48585 | 41 | 83.01 | Copepoda | 0.374 | 0.81 | 0.01339 |
| JFBM48585 | 41 | 83.01 | Copepoda | 0.286 | 0.797 | 0.01331 |
| JFBM48585 | 41 | 83.01 | Copepoda | 0.448 | 0.823 | 0.03108 |
| JFBM48585 | 41 | 83.01 | Copepoda | 0.323 |  | 0.0168 |
| JFBM48585 | 41 | 83.01 | Copepoda | 0.367 |  | 0.02633 |
| JFBM48585 | 41 | 83.01 | Copepoda | 0.304 | 0.7 | 0.01254 |
| JFBM48585 | 41 | 83.01 | Copepoda | 0.421 | 0.782 | 0.01474 |
| JFBM48585 | 41 | 83.01 | Copepoda | 0.309 | 0.779 | 0.01707 |
| JFBM48585 | 41 | 83.01 | Copepoda | 0.385 |  | 0.03192 |
| JFBM48585 | 41 | 83.01 | Copepoda | 0.314 | 0.876 | 0.01753 |
| JFBM48585 | 41 | 83.01 | Copepoda | 0.387 | 0.806 | 0.01114 |
| JFBM48585 | 41 | 83.01 | Copepoda | 0.287 |  | 0.01154 |
| JFBM48585 | 41 | 83.01 | Copepoda | 0.318 | 0.836 | 0.01237 |
| JFBM48585 | 41 | 83.01 | Copepoda | 0.433 | 0.62 | 0.05618 |
| JFBM48585 | 41 | 83.01 | Copepoda | 0.417 | 0.96 | 0.01808 |
| JFBM48585 | 41 | 83.01 | Copepoda | 0.424 | 0.848 | 0.01157 |
| JFBM48585 | 41 | 83.01 | Copepoda | 0.412 |  | 0.04184 |
| JFBM48585 | 41 | 83.01 | Copepoda | 0.436 |  | 0.05397 |
| JFBM48585 | 41 | 83.01 | Copepoda | 0.423 | 0.901 | 0.02435 |
| JFBM48585 | 41 | 83.01 | Copepoda | 0.439 |  | 0.05552 |
| JFBM48585 | 41 | 83.01 | Copepoda | 0.473 |  | 0.07874 |
| JFBM48585 | 41 | 83.01 | Copepoda | 0.487 |  | 0.09137 |
| JFBM48585 | 41 | 83.01 | Copepoda | 0.348 |  | 0.02166 |
| JFBM48585 | 41 | 83.01 | Copepoda | 0.323 |  | 0.01682 |
| JFBM48585 | 41 | 83.01 | Copepoda | 0.46 | 1.018 | 0.02013 |
| JFBM48585 | 41 | 83.01 | Copepoda | 0.36 |  | 0.02456 |
| JFBM48585 | 41 | 83.01 | Copepoda | 0.246 |  | 0.00759 |
| JFBM48585 | 41 | 83.01 | Copepoda | 0.365 |  | 0.02574 |
| JFBM48585 | 41 | 83.01 | Copepoda | 0.292 |  | 0.01221 |
| JFBM48585 | 41 | 83.01 | Copepoda | 0.495 |  | 0.09884 |
| JFBM48585 | 41 | 83.01 | Copepoda | 0.404 |  | 0.03865 |
| JFBM48585 | 41 | 83.01 | Copepoda | 0.423 | 0.922 | 0.01855 |
| JFBM48585 | 41 | 83.01 | Copepoda | 0.404 |  | 0.03878 |
| JFBM48585 | 41 | 83.01 | Copepoda | 0.349 |  | 0.02188 |
| JFBM48585 | 41 | 83.01 | Copepoda | 0.333 |  | 0.01858 |
| JFBM48585 | 41 | 83.01 | Copepoda | 0.378 | 0.94 | 0.01448 |
| JFBM48585 | 41 | 83.01 | Copepoda | 0.467 | 0.859 | 0.02349 |
| JFBM48585 | 41 | 83.01 | Copepoda | 0.281 |  | 0.01088 |
| JFBM48585 | 41 | 83.01 | Copepoda | 0.295 |  | 0.01262 |
| JFBM48585 | 41 | 83.01 | Copepoda | 0.3 |  | 0.01316 |
| JFBM48585 | 41 | 83.01 | Copepoda | 0.332 |  | 0.01847 |
| JFBM48585 | 41 | 83.01 | Copepoda | 0.471 | 1.1 | 0.03719 |
| JFBM48585 | 41 | 83.01 | Copepoda | 0.35 |  | 0.02208 |
| JFBM48585 | 41 | 83.01 | Copepoda | 0.369 | 1.04 | 0.02343 |
| JFBM48585 | 41 | 83.01 | Copepoda | 0.345 |  | 0.02106 |
| JFBM48585 | 41 | 83.01 | Copepoda | 0.323 |  | 0.01672 |
| JFBM48585 | 41 | 83.01 | Copepoda | 0.348 |  | 0.02171 |
| JFBM48585 | 41 | 83.01 | Copepoda | 0.467 |  | 0.07399 |
| JFBM48585 | 42 | 78.98 | Copepoda | 0.3 | 0.912 | 0.01507 |
| JFBM48585 | 42 | 78.98 | Copepoda | 0.436 | 1.154 | 0.02401 |
| JFBM48585 | 42 | 78.98 | Copepoda | 0.303 |  | 0.01367 |
| JFBM48585 | 42 | 78.98 | Copepoda | 0.425 |  | 0.04819 |
| JFBM48585 | 42 | 78.98 | Copepoda | 0.403 | 0.776 | 0.02358 |
| JFBM48585 | 42 | 78.98 | Copepoda | 0.434 | 1.118 | 0.02532 |
| JFBM48585 | 42 | 78.98 | Copepoda | 0.504 | 0.903 | 0.02806 |
| JFBM48585 | 42 | 78.98 | Copepoda | 0.494 | 0.93 | 0.01809 |
| JFBM48585 | 42 | 78.98 | Copepoda | 0.353 | 0.841 | 0.01551 |
| JFBM48585 | 42 | 78.98 | Copepoda | 0.375 |  | 0.02868 |
| JFBM48585 | 42 | 78.98 | Copepoda | 0.374 | 0.843 | 0.01677 |
| JFBM48585 | 42 | 78.98 | Cladocera | 0.38 |  | 0.03025 |
| JFBM48585 | 42 | 78.98 | Copepoda | 0.382 |  | 0.03066 |
| JFBM48585 | 42 | 78.98 | Copepoda | 0.314 | 0.805 | 0.00873 |
| JFBM48585 | 42 | 78.98 | Cladocera | 0.253 |  | 0.00811 |
| JFBM48585 | 42 | 78.98 | Cladocera | 0.503 |  | 0.10743 |
| JFBM48585 | 42 | 78.98 | Cladocera | 0.368 |  | 0.02663 |
| JFBM48585 | 42 | 78.98 | Cladocera | 0.255 |  | 0.0083 |
| JFBM48585 | 42 | 78.98 | Insecta larvae | 0.369 |  | 0.27757 |
| JFBM48585 | 42 | 78.98 | Copepoda | 0.476 | 0.919 | 0.026 |
| JFBM48585 | 42 | 78.98 | Copepoda | 0.462 |  | 0.07036 |
| JFBM48585 | 42 | 78.98 | Copepoda | 0.371 |  | 0.02751 |
| JFBM48585 | 42 | 78.98 | Copepoda | 0.418 | 1.115 | 0.03615 |
| JFBM48585 | 42 | 78.98 | Copepoda | 0.423 | 0.918 | 0.02428 |
| JFBM48585 | 42 | 78.98 | Copepoda | 0.449 |  | 0.06171 |
| JFBM48585 | 42 | 78.98 | Copepoda | 0.382 |  | 0.03066 |
| JFBM48585 | 42 | 78.98 | Copepoda | 0.411 | 0.972 | 0.02724 |
| JFBM48585 | 42 | 78.98 | Copepoda | 0.418 | 1.206 | 0.02964 |
| JFBM48585 | 42 | 78.98 | Copepoda | 0.35 |  | 0.02208 |
| JFBM48585 | 42 | 78.98 | Copepoda | 0.451 | 1.013 | 0.02297 |
| JFBM48585 | 42 | 78.98 | Copepoda | 0.467 |  | 0.07399 |
| JFBM48585 | 42 | 78.98 | Copepoda | 0.408 | 1.012 | 0.02189 |
| JFBM48585 | 42 | 78.98 | Copepoda | 0.25 |  | 0.00787 |
| JFBM48585 | 42 | 78.98 | Copepoda | 0.383 | 0.771 | 0.01041 |
| JFBM48585 | 42 | 78.98 | Copepoda | 0.317 |  | 0.01576 |
| JFBM48585 | 42 | 78.98 | Copepoda | 0.395 | 0.794 | 0.01972 |
| JFBM48585 | 42 | 78.98 | Copepoda | 0.314 |  | 0.01521 |
| JFBM48585 | 42 | 78.98 | Copepoda | 0.413 |  | 0.04257 |
| JFBM48585 | 42 | 78.98 | Copepoda | 0.519 | 1.07 | 0.03889 |
| JFBM48585 | 42 | 78.98 | Copepoda | 0.507 | 0.888 | 0.02 |
| JFBM48585 | 42 | 78.98 | Copepoda | 0.492 | 0.774 | 0.01816 |
| JFBM48585 | 42 | 78.98 | Copepoda | 0.499 |  | 0.10309 |
| JFBM48585 | 42 | 78.98 | Copepoda | 0.431 | 1.028 | 0.02337 |
| JFBM48585 | 42 | 78.98 | Copepoda | 0.282 |  | 0.01103 |
| JFBM48585 | 42 | 78.98 | Copepoda | 0.485 | 0.97 | 0.02361 |
| JFBM48585 | 42 | 78.98 | Copepoda | 0.296 |  | 0.01268 |
| JFBM48585 | 42 | 78.98 | Copepoda | 0.449 |  | 0.06137 |
| JFBM48585 | 42 | 78.98 | Copepoda | 0.4 | 0.726 | 0.01339 |
| JFBM48585 | 42 | 78.98 | Copepoda | 0.315 |  | 0.01543 |
| JFBM48585 | 42 | 78.98 | Copepoda | 0.529 | 0.831 | 0.02872 |
| JFBM48585 | 42 | 78.98 | Copepoda | 0.446 | 0.879 | 0.01951 |
| JFBM48585 | 42 | 78.98 | Copepoda | 0.385 | 0.689 | 0.017 |
| JFBM48585 | 42 | 78.98 | Copepoda | 0.356 | 0.857 | 0.02233 |
| JFBM48585 | 42 | 78.98 | Copepoda | 0.342 |  | 0.02044 |
| JFBM48585 | 42 | 78.98 | Copepoda | 0.439 |  | 0.05551 |
| JFBM48585 | 42 | 78.98 | Copepoda | 0.335 |  | 0.01893 |
| JFBM48585 | 42 | 78.98 | Copepoda | 0.5 | 0.998 | 0.02839 |
| JFBM48585 | 42 | 78.98 | Copepoda | 0.36 | 0.765 | 0.01562 |
| JFBM48585 | 42 | 78.98 | Copepoda | 0.379 | 0.861 | 0.02151 |
| JFBM48585 | 42 | 78.98 | Copepoda | 0.342 | 0.859 | 0.01736 |
| JFBM48585 | 42 | 78.98 | Copepoda | 0.319 |  | 0.01604 |
| JFBM48585 | 42 | 78.98 | Copepoda | 0.26 |  | 0.00875 |
| JFBM48585 | 42 | 78.98 | Copepoda | 0.36 | 0.903 | 0.02763 |
| JFBM48585 | 42 | 78.98 | Copepoda | 0.34 | 0.845 | 0.01074 |
| JFBM48585 | 42 | 78.98 | Copepoda | 0.319 | 0.809 | 0.00867 |
| JFBM48585 | 42 | 78.98 | Copepoda | 0.352 | 0.877 | 0.01577 |
| JFBM48585 | 42 | 78.98 | Copepoda | 0.357 | 0.826 | 0.01675 |
| JFBM48585 | 42 | 78.98 | Copepoda | 0.317 | 0.888 | 0.0234 |
| JFBM48585 | 42 | 78.98 | Copepoda | 0.327 | 0.839 | 0.02266 |
| JFBM48585 | 42 | 78.98 | Copepoda | 0.319 |  | 0.01603 |
| JFBM48585 | 42 | 78.98 | Copepoda | 0.268 |  | 0.0095 |
| JFBM48585 | 42 | 78.98 | Copepoda | 0.291 |  | 0.01204 |
| JFBM48585 | 42 | 78.98 | Copepoda | 0.221 |  | 0.00584 |
| JFBM48585 | 42 | 78.98 | Copepoda | 0.424 | 0.986 | 0.02154 |
| JFBM48585 | 42 | 78.98 | Copepoda | 0.272 |  | 0.00993 |
| JFBM48585 | 42 | 78.98 | Copepoda | 0.316 | 0.882 | 0.00931 |
| JFBM48585 | 42 | 78.98 | Copepoda | 0.437 | 1.096 | 0.01662 |
| JFBM48585 | 42 | 78.98 | Copepoda | 0.458 | 0.915 | 0.03247 |
| JFBM48585 | 42 | 78.98 | Copepoda | 0.333 |  | 0.01864 |
| JFBM48585 | 42 | 78.98 | Copepoda | 0.464 | 0.992 | 0.02152 |
| JFBM48585 | 43 | 77.65 | Copepoda | 0.366 | 0.916 | 0.02422 |
| JFBM48585 | 43 | 77.65 | Copepoda | 0.365 | 1.07 | 0.01191 |
| JFBM48585 | 43 | 77.65 | Copepoda | 0.311 |  | 0.01483 |
| JFBM48585 | 43 | 77.65 | Copepoda | 0.277 |  | 0.01043 |
| JFBM48585 | 43 | 77.65 | Copepoda | 0.451 |  | 0.06263 |
| JFBM48585 | 43 | 77.65 | Copepoda | 0.341 |  | 0.02029 |
| JFBM48585 | 43 | 77.65 | Copepoda | 0.343 |  | 0.02055 |
| JFBM48585 | 43 | 77.65 | Copepoda | 0.447 |  | 0.0602 |
| JFBM48585 | 43 | 77.65 | Copepoda | 0.375 |  | 0.02868 |
| JFBM48585 | 43 | 77.65 | Copepoda | 0.415 |  | 0.04325 |
| JFBM48585 | 43 | 77.65 | Copepoda | 0.342 | 0.768 | 0.01913 |
| JFBM48585 | 43 | 77.65 | Copepoda | 0.279 | 0.82 | 0.01141 |
| JFBM48585 | 43 | 77.65 | Copepoda | 0.274 |  | 0.01007 |
| JFBM48585 | 43 | 77.65 | Copepoda | 0.309 | 0.73 | 0.01368 |
| JFBM48585 | 43 | 77.65 | Copepoda | 0.323 | 0.776 | 0.01545 |
| JFBM48585 | 43 | 77.65 | Copepoda | 0.42 | 0.968 | 0.0249 |
| JFBM48585 | 43 | 77.65 | Copepoda | 0.297 |  | 0.01286 |
| JFBM48585 | 43 | 77.65 | Copepoda | 0.313 | 0.774 | 0.01159 |
| JFBM48585 | 43 | 77.65 | Copepoda | 0.36 | 0.847 | 0.01187 |
| JFBM48585 | 43 | 77.65 | Copepoda | 0.338 | 0.858 | 0.01078 |
| JFBM48585 | 43 | 77.65 | Copepoda | 0.377 |  | 0.02941 |
| JFBM48585 | 43 | 77.65 | Copepoda | 0.322 | 0.817 | 0.0153 |
| JFBM48585 | 43 | 77.65 | Copepoda | 0.255 |  | 0.0083 |
| JFBM48585 | 43 | 77.65 | Copepoda | 0.297 | 0.858 | 0.01173 |
| JFBM48585 | 43 | 77.65 | Copepoda | 0.242 |  | 0.00727 |
| JFBM48585 | 43 | 77.65 | Copepoda | 0.444 | 0.859 | 0.02629 |
| JFBM48585 | 43 | 77.65 | Copepoda | 0.296 | 0.766 | 0.02037 |
| JFBM48585 | 43 | 77.65 | Copepoda | 0.309 |  | 0.01453 |
| JFBM48585 | 43 | 77.65 | Copepoda | 0.349 |  | 0.02192 |
| JFBM48585 | 43 | 77.65 | Copepoda | 0.446 | 0.797 | 0.01408 |
| JFBM48585 | 43 | 77.65 | Copepoda | 0.297 |  | 0.01286 |
| JFBM48585 | 43 | 77.65 | Copepoda | 0.353 |  | 0.02281 |
| JFBM48585 | 43 | 77.65 | Copepoda | 0.343 |  | 0.02058 |
| JFBM48585 | 43 | 77.65 | Copepoda | 0.25 |  | 0.0079 |
| JFBM48585 | 43 | 77.65 | Copepoda | 0.489 | 0.791 | 0.01897 |
| JFBM48585 | 43 | 77.65 | Copepoda | 0.314 | 0.732 | 0.01606 |
| JFBM48585 | 43 | 77.65 | Copepoda | 0.337 |  | 0.01939 |
| JFBM48585 | 43 | 77.65 | Copepoda | 0.361 | 0.878 | 0.02209 |
| JFBM48585 | 43 | 77.65 | Copepoda | 0.433 |  | 0.05211 |
| JFBM48585 | 43 | 77.65 | Copepoda | 0.387 | 0.916 | 0.01635 |
| JFBM48585 | 43 | 77.65 | Copepoda | 0.384 | 0.935 | 0.02121 |
| JFBM48585 | 43 | 77.65 | Copepoda | 0.419 |  | 0.04491 |
| JFBM48585 | 43 | 77.65 | Copepoda | 0.414 |  | 0.04277 |
| JFBM48585 | 43 | 77.65 | Copepoda | 0.331 |  | 0.01822 |
| JFBM48585 | 44 | 76.11 | Copepoda | 0.312 |  | 0.01501 |
| JFBM48585 | 44 | 76.11 | Copepoda | 0.307 | 0.754 | 0.01107 |
| JFBM48585 | 44 | 76.11 | Copepoda | 0.515 |  | 0.1221 |
| JFBM48585 | 44 | 76.11 | Copepoda | 0.348 |  | 0.02166 |
| JFBM48585 | 44 | 76.11 | Copepoda | 0.342 |  | 0.02046 |
| JFBM48585 | 44 | 76.11 | Copepoda | 0.24 |  | 0.00711 |
| JFBM48585 | 44 | 76.11 | Copepoda | 0.253 |  | 0.00816 |
| JFBM48585 | 44 | 76.11 | Copepoda | 0.446 |  | 0.0599 |
| JFBM48585 | 44 | 76.11 | Copepoda | 0.468 |  | 0.07452 |
| JFBM48585 | 44 | 76.11 | Copepoda | 0.502 |  | 0.1068 |
| JFBM48585 | 44 | 76.11 | Copepoda | 0.389 |  | 0.03317 |
| JFBM48585 | 44 | 76.11 | Copepoda | 0.367 |  | 0.02634 |
| JFBM48585 | 44 | 76.11 | Copepoda | 0.343 |  | 0.02063 |
| JFBM48585 | 44 | 76.11 | Copepoda | 0.319 |  | 0.01615 |
| JFBM48585 | 44 | 76.11 | Copepoda | 0.371 | 0.92 | 0.01771 |
| JFBM48585 | 44 | 76.11 | Copepoda | 0.38 |  | 0.03015 |
| JFBM48585 | 44 | 76.11 | Copepoda | 0.211 |  | 0.0053 |
| JFBM48585 | 44 | 76.11 | Copepoda | 0.27 |  | 0.00969 |
| JFBM48585 | 44 | 76.11 | Copepoda | 0.301 |  | 0.01339 |
| JFBM48585 | 44 | 76.11 | Copepoda | 0.347 |  | 0.02152 |
| JFBM48585 | 44 | 76.11 | Copepoda | 0.381 |  | 0.03042 |
| JFBM48585 | 44 | 76.11 | Copepoda | 0.514 | 0.951 | 0.03365 |
| JFBM48585 | 44 | 76.11 | Copepoda | 0.495 |  | 0.09937 |
| JFBM48585 | 44 | 76.11 | Copepoda | 0.364 |  | 0.02553 |
| JFBM48585 | 44 | 76.11 | Copepoda | 0.365 | 0.728 | 0.01052 |
| JFBM48585 | 44 | 76.11 | Copepoda | 0.304 | 0.726 | 0.01154 |
| JFBM48585 | 44 | 76.11 | Copepoda | 0.364 |  | 0.02572 |
| JFBM48585 | 44 | 76.11 | Copepoda | 0.378 |  | 0.02969 |
| JFBM48585 | 44 | 76.11 | Copepoda | 0.328 |  | 0.01774 |
| JFBM48585 | 44 | 76.11 | Copepoda | 0.353 |  | 0.02277 |
| JFBM48585 | 44 | 76.11 | Copepoda | 0.358 |  | 0.02416 |
| JFBM48585 | 44 | 76.11 | Copepoda | 0.292 |  | 0.01222 |
| JFBM48585 | 44 | 76.11 | Copepoda | 0.455 |  | 0.06546 |
| JFBM48585 | 44 | 76.11 | Copepoda | 0.308 |  | 0.01442 |
| JFBM48585 | 44 | 76.11 | Copepoda | 0.314 |  | 0.01526 |
| JFBM48585 | 44 | 76.11 | Copepoda | 0.401 |  | 0.03762 |
| JFBM48585 | 44 | 76.11 | Copepoda | 0.44 |  | 0.05594 |
| JFBM48585 | 44 | 76.11 | Copepoda | 0.433 |  | 0.05198 |
| JFBM48585 | 44 | 76.11 | Copepoda | 0.346 |  | 0.02131 |
| JFBM48585 | 44 | 76.11 | Copepoda | 0.301 |  | 0.01341 |
| JFBM48585 | 44 | 76.11 | Copepoda | 0.336 |  | 0.01918 |
| JFBM48585 | 44 | 76.11 | Copepoda | 0.326 |  | 0.01726 |
| JFBM48585 | 44 | 76.11 | Copepoda | 0.449 | 0.81 | 0.01602 |
| JFBM48585 | 44 | 76.11 | Copepoda | 0.377 |  | 0.02928 |
| JFBM48585 | 44 | 76.11 | Copepoda | 0.411 |  | 0.04172 |
| JFBM48585 | 44 | 76.11 | Copepoda | 0.444 |  | 0.05813 |
| JFBM48585 | 44 | 76.11 | Copepoda | 0.436 | 0.895 | 0.02257 |
| JFBM48585 | 45 | 72.15 | Copepoda | 0.304 |  | 0.01375 |
| JFBM48585 | 45 | 72.15 | Copepoda | 0.391 |  | 0.03365 |
| JFBM48585 | 45 | 72.15 | Copepoda | 0.329 |  | 0.01792 |
| JFBM48585 | 45 | 72.15 | Copepoda | 0.339 |  | 0.01976 |
| JFBM48585 | 45 | 72.15 | Copepoda | 0.301 | 1.033 | 0.01822 |
| JFBM48585 | 45 | 72.15 | Copepoda | 0.328 |  | 0.0176 |
| JFBM48585 | 45 | 72.15 | Copepoda | 0.333 |  | 0.01851 |
| JFBM48585 | 45 | 72.15 | Copepoda | 0.384 |  | 0.0315 |
| JFBM48585 | 45 | 72.15 | Copepoda | 0.338 | 0.842 | 0.01032 |
| JFBM48585 | 45 | 72.15 | Copepoda | 0.354 |  | 0.02319 |
| JFBM48585 | 45 | 72.15 | Copepoda | 0.345 |  | 0.02108 |
| JFBM48585 | 45 | 72.15 | Copepoda | 0.344 |  | 0.02084 |
| JFBM48585 | 45 | 72.15 | Copepoda | 0.329 |  | 0.01778 |
| JFBM48585 | 45 | 72.15 | Copepoda | 0.294 |  | 0.01238 |
| JFBM48585 | 46 | 72.96 | Hymenoptera terrestrial | 2.824 | 9.866 | 6.06346 |
| JFBM48585 | 46 | 72.96 | Hymenoptera terrestrial | 2.542 |  | 5.5095 |
| JFBM48585 | 46 | 72.96 | Hymenoptera terrestrial | 2.608 |  | 5.66838 |
| JFBM48585 | 46 | 72.96 | Hymenoptera terrestrial | 2.19 |  | 4.66217 |
| JFBM48585 | 46 | 72.96 | Hymenoptera terrestrial | 2.114 |  | 4.47922 |
| JFBM48585 | 46 | 72.96 | Hymenoptera terrestrial | 2.054 |  | 4.33479 |
| JFBM48585 | 46 | 72.96 | Hymenoptera terrestrial | 2.216 |  | 4.72476 |
| JFBM48585 | 46 | 72.96 | Hymenoptera terrestrial | 2.41 |  | 5.19175 |
| JFBM48585 | 46 | 72.96 | Copepoda | 0.313 | 0.833 | 0.01114 |
| JFBM48585 | 46 | 72.96 | Copepoda | 0.383 |  | 0.03109 |
| JFBM48585 | 46 | 72.96 | Insecta larvae | 0.582 | 5.395 | 0.10693 |
| JFBM48585 | 46 | 72.96 | Copepoda | 0.26 | 0.915 | 0.0067 |
| JFBM48585 | 46 | 72.96 | Copepoda | 0.333 |  | 0.01858 |
| JFBM48585 | 46 | 72.96 | Copepoda | 0.395 | 0.868 | 0.01814 |
| JFBM48585 | 46 | 72.96 | Insecta larvae aquatic | 0.404 |  | 0.57001 |
| JFBM48585 | 46 | 72.96 | Copepoda | 0.325 | 0.696 | 0.01867 |
| JFBM48585 | 46 | 72.96 | Copepoda | 0.3 |  | 0.01316 |
| JFBM48585 | 46 | 72.96 | Copepoda | 0.353 |  | 0.02286 |
| JFBM48585 | 46 | 72.96 | Copepoda | 0.388 |  | 0.03275 |
| JFBM48585 | 46 | 72.96 | Cladocera | 0.39 |  | 0.03334 |
| JFBM48585 | 46 | 72.96 | Copepoda | 0.382 | 0.935 | 0.02289 |
| JFBM48585 | 46 | 72.96 | Copepoda | 0.434 | 0.93 | 0.01664 |
| JFBM48585 | 46 | 72.96 | Copepoda | 0.271 | 0.698 | 0.00914 |
| JFBM48585 | 46 | 72.96 | Copepoda | 0.399 | 0.934 | 0.02034 |
| JFBM48585 | 46 | 72.96 | Copepoda | 0.35 | 0.901 | 0.02113 |
| JFBM48585 | 46 | 72.96 | Copepoda | 0.343 | 0.68 | 0.01237 |
| JFBM48585 | 46 | 72.96 | Copepoda | 0.318 | 0.881 | 0.00962 |
| JFBM48585 | 46 | 72.96 | Copepoda | 0.276 |  | 0.01031 |
| JFBM48585 | 46 | 72.96 | Copepoda | 0.374 |  | 0.02847 |
| JFBM48585 | 46 | 72.96 | Copepoda | 0.384 |  | 0.03162 |
| JFBM48585 | 46 | 72.96 | Copepoda | 0.438 | 1.013 | 0.02466 |
| JFBM48585 | 46 | 72.96 | Copepoda | 0.428 |  | 0.04933 |
| JFBM48585 | 46 | 72.96 | Copepoda | 0.365 |  | 0.02598 |
| JFBM48585 | 46 | 72.96 | Copepoda | 0.275 | 0.723 | 0.00702 |
| JFBM48585 | 46 | 72.96 | Copepoda | 0.351 | 0.809 | 0.01717 |
| JFBM48585 | 46 | 72.96 | Copepoda | 0.254 | 0.705 | 0.00732 |
| JFBM48585 | 46 | 72.96 | Copepoda | 0.338 | 0.874 | 0.01863 |
| JFBM48585 | 46 | 72.96 | Copepoda | 0.191 | 0.449 | 0.00314 |
| JFBM48585 | 46 | 72.96 | Copepoda | 0.32 | 0.69 | 0.00787 |
| JFBM48585 | 46 | 72.96 | Copepoda | 0.338 | 0.834 | 0.01943 |
| JFBM48585 | 46 | 72.96 | Copepoda | 0.297 |  | 0.01281 |
| JFBM48585 | 46 | 72.96 | Copepoda | 0.312 | 0.681 | 0.00953 |
| JFBM48585 | 46 | 72.96 | Copepoda | 0.306 |  | 0.01406 |
| JFBM48585 | 46 | 72.96 | Copepoda | 0.259 | 0.566 | 0.01492 |
| JFBM48585 | 46 | 72.96 | Copepoda | 0.282 |  | 0.01101 |
| JFBM48585 | 46 | 72.96 | Copepoda | 0.318 | 0.916 | 0.0168 |
| JFBM48585 | 46 | 72.96 | Copepoda | 0.237 |  | 0.00692 |
| JFBM48585 | 46 | 72.96 | Copepoda | 0.25 |  | 0.0079 |
| JFBM48585 | 46 | 72.96 | Copepoda | 0.437 | 0.841 | 0.02229 |
| JFBM48585 | 46 | 72.96 | Copepoda | 0.408 | 0.9 | 0.02792 |
| JFBM48585 | 46 | 72.96 | Copepoda | 0.261 | 0.582 | 0.00406 |
| JFBM48585 | 46 | 72.96 | Copepoda | 0.302 | 0.73 | 0.01066 |
| JFBM48585 | 46 | 72.96 | Copepoda | 0.233 | 0.693 | 0.00673 |
| JFBM48585 | 46 | 72.96 | Copepoda | 0.346 | 0.726 | 0.01598 |
| JFBM48585 | 46 | 72.96 | Copepoda | 0.296 | 0.864 | 0.02021 |
| JFBM48585 | 46 | 72.96 | Copepoda | 0.293 |  | 0.01235 |
| JFBM48585 | 46 | 72.96 | Copepoda | 0.186 |  | 0.00409 |
| JFBM48585 | 46 | 72.96 | Copepoda | 0.201 |  | 0.00474 |
| JFBM48585 | 46 | 72.96 | Copepoda | 0.301 | 0.894 | 0.02002 |
| JFBM48585 | 46 | 72.96 | Copepoda | 0.338 | 0.835 | 0.02448 |
| JFBM48585 | 46 | 72.96 | Copepoda | 0.374 | 0.88 | 0.01699 |
| JFBM48585 | 46 | 72.96 | Copepoda | 0.329 | 0.731 | 0.01713 |
| JFBM48585 | 46 | 72.96 | Copepoda | 0.318 | 0.958 | 0.01428 |
| JFBM48585 | 46 | 72.96 | Copepoda | 0.359 | 0.686 | 0.00969 |
| JFBM48585 | 46 | 72.96 | Copepoda | 0.376 |  | 0.02906 |
| JFBM48585 | 46 | 72.96 | Copepoda | 0.454 |  | 0.0651 |
| JFBM48585 | 46 | 72.96 | Copepoda | 0.35 | 0.923 | 0.00818 |
| JFBM48585 | 46 | 72.96 | Copepoda | 0.332 | 0.784 | 0.01283 |
| JFBM48585 | 46 | 72.96 | Copepoda | 0.389 |  | 0.03324 |
| JFBM48585 | 46 | 72.96 | Copepoda | 0.446 | 1.14 | 0.02997 |
| JFBM48585 | 46 | 72.96 | Copepoda | 0.347 | 0.796 | 0.0134 |
| JFBM48585 | 46 | 72.96 | Copepoda | 0.362 | 1.129 | 0.01697 |
| JFBM48585 | 46 | 72.96 | Copepoda | 0.319 | 0.824 | 0.01501 |
| JFBM48585 | 46 | 72.96 | Copepoda | 0.355 |  | 0.02342 |
| JFBM48585 | 46 | 72.96 | Copepoda | 0.238 |  | 0.00698 |
| JFBM48585 | 46 | 72.96 | Copepoda | 0.351 | 0.674 | 0.00505 |
| JFBM48585 | 46 | 72.96 | Copepoda | 0.35 |  | 0.0222 |
| JFBM48585 | 46 | 72.96 | Copepoda | 0.345 |  | 0.02097 |
| JFBM48585 | 46 | 72.96 | Copepoda | 0.375 | 0.782 | 0.01565 |
| JFBM48585 | 46 | 72.96 | Copepoda | 0.305 | 0.69 | 0.01287 |
| JFBM48585 | 46 | 72.96 | Copepoda | 0.302 | 0.81 | 0.00773 |
| JFBM48585 | 46 | 72.96 | Copepoda | 0.277 |  | 0.0104 |
| JFBM48585 | 46 | 72.96 | Copepoda | 0.438 | 0.833 | 0.02659 |
| JFBM48585 | 46 | 72.96 | Copepoda | 0.429 |  | 0.04995 |
| JFBM48585 | 46 | 72.96 | Copepoda | 0.416 | 0.997 | 0.02535 |
| JFBM48585 | 46 | 72.96 | Copepoda | 0.356 | 0.893 | 0.01519 |
| JFBM48585 | 46 | 72.96 | Copepoda | 0.271 | 0.644 | 0.00572 |
| JFBM48585 | 46 | 72.96 | Copepoda | 0.407 |  | 0.04004 |
| JFBM48585 | 46 | 72.96 | Copepoda | 0.281 |  | 0.01085 |
| JFBM48585 | 46 | 72.96 | Copepoda | 0.346 |  | 0.02116 |
| JFBM48585 | 46 | 72.96 | Copepoda | 0.253 |  | 0.00813 |
| JFBM48585 | 46 | 72.96 | Copepoda | 0.346 | 0.947 | 0.02257 |
| JFBM48585 | 46 | 72.96 | Copepoda | 0.318 | 1.021 | 0.0115 |
| JFBM48585 | 46 | 72.96 | Copepoda | 0.248 | 0.815 | 0.01178 |
| JFBM48585 | 46 | 72.96 | Copepoda | 0.42 | 1.191 | 0.02348 |
| JFBM48585 | 46 | 72.96 | Copepoda | 0.312 | 1.031 | 0.02144 |
| JFBM48585 | 46 | 72.96 | Copepoda | 0.298 | 0.813 | 0.01166 |
| JFBM48585 | 46 | 72.96 | Copepoda | 0.378 | 0.871 | 0.01754 |
| JFBM48585 | 46 | 72.96 | Copepoda | 0.364 | 0.915 | 0.02507 |
| JFBM48585 | 46 | 72.96 | Copepoda | 0.211 |  | 0.00527 |
| JFBM48585 | 46 | 72.96 | Insecta larvae | 0.433 |  | 0.81576 |
| JFBM48585 | 46 | 72.96 | Insecta larvae | 0.5 |  | 1.3777 |
| JFBM48585 | 46 | 72.96 | Copepoda | 0.359 | 0.776 | 0.01473 |
| JFBM48585 | 46 | 72.96 | Copepoda | 0.289 | 0.832 | 0.00787 |
| JFBM48585 | 46 | 72.96 | Copepoda | 0.279 |  | 0.01068 |
| JFBM48585 | 46 | 72.96 | Copepoda | 0.352 | 0.786 | 0.00803 |
| JFBM48585 | 46 | 72.96 | Copepoda | 0.354 | 0.767 | 0.01604 |
| JFBM48585 | 46 | 72.96 | Copepoda | 0.273 |  | 0.00996 |
| JFBM48585 | 46 | 72.96 | Copepoda | 0.302 |  | 0.01344 |
| JFBM48585 | 46 | 72.96 | Copepoda | 0.339 | 0.877 | 0.01086 |
| JFBM48585 | 46 | 72.96 | Copepoda | 0.346 | 0.87 | 0.01843 |
| JFBM48585 | 46 | 72.96 | Copepoda | 0.282 | 0.624 | 0.0053 |
| JFBM48585 | 47 | 74.86 | Copepoda | 0.28 |  | 0.0108 |
| JFBM48585 | 47 | 74.86 | Copepoda | 0.366 |  | 0.02604 |
| JFBM48585 | 47 | 74.86 | Copepoda | 0.429 | 0.912 | 0.02164 |
| JFBM48585 | 47 | 74.86 | Copepoda | 0.392 | 0.917 | 0.01635 |
| JFBM48585 | 47 | 74.86 | Copepoda | 0.398 | 0.92 | 0.01631 |
| JFBM48585 | 47 | 74.86 | Copepoda | 0.411 | 1.035 | 0.0245 |
| JFBM48585 | 47 | 74.86 | Copepoda | 0.466 | 0.851 | 0.02021 |
| JFBM48585 | 47 | 74.86 | Copepoda | 0.449 | 1.088 | 0.01862 |
| JFBM48585 | 47 | 74.86 | Copepoda | 0.37 | 1.1 | 0.02571 |
| JFBM48585 | 47 | 74.86 | Copepoda | 0.361 |  | 0.02483 |
| JFBM48585 | 47 | 74.86 | Copepoda | 0.404 |  | 0.03849 |
| JFBM48585 | 47 | 74.86 | Copepoda | 0.45 |  | 0.06213 |
| JFBM48585 | 47 | 74.86 | Copepoda | 0.345 | 0.795 | 0.01301 |
| JFBM48585 | 47 | 74.86 | Copepoda | 0.355 | 0.85 | 0.01911 |
| JFBM48585 | 47 | 74.86 | Copepoda | 0.355 | 0.84 | 0.01421 |
| JFBM48585 | 47 | 74.86 | Copepoda | 0.52 |  | 0.12845 |
| JFBM48585 | 47 | 74.86 | Copepoda | 0.386 |  | 0.03223 |
| JFBM48585 | 47 | 74.86 | Copepoda | 0.437 | 1.056 | 0.01957 |
| JFBM48585 | 47 | 74.86 | Copepoda | 0.302 | 1.183 | 0.01596 |
| JFBM48585 | 48 | 86.36 | Copepoda | 0.362 | 0.908 | 0.01613 |
| JFBM48585 | 48 | 86.36 | Copepoda | 0.417 |  | 0.04422 |
| JFBM48585 | 48 | 86.36 | Copepoda | 0.42 |  | 0.04579 |
| JFBM48585 | 48 | 86.36 | Copepoda | 0.388 |  | 0.03264 |
| JFBM48585 | 48 | 86.36 | Insecta unknown | 0.654 |  | 0.96504 |
| JFBM48585 | 48 | 86.36 | Copepoda | 0.391 | 0.928 | 0.01709 |
| JFBM48585 | 48 | 86.36 | Copepoda | 0.321 |  | 0.01644 |
| JFBM48585 | 48 | 86.36 | Copepoda | 0.34 |  | 0.01994 |
| JFBM48585 | 48 | 86.36 | Copepoda | 0.439 |  | 0.05531 |
| JFBM48585 | 48 | 86.36 | Copepoda | 0.433 |  | 0.05235 |
| JFBM48585 | 48 | 86.36 | Copepoda | 0.336 |  | 0.0191 |
| JFBM48585 | 48 | 86.36 | Copepoda | 0.358 | 0.843 | 0.01101 |
| JFBM48585 | 48 | 86.36 | Copepoda | 0.409 |  | 0.04061 |
| JFBM48585 | 48 | 86.36 | Copepoda | 0.561 |  | 0.19597 |
| JFBM48585 | 48 | 86.36 | Copepoda | 0.309 | 0.837 | 0.00812 |
| JFBM48585 | 48 | 86.36 | Copepoda | 0.246 |  | 0.00759 |
| JFBM48585 | 48 | 86.36 | Copepoda | 0.288 |  | 0.01171 |
| JFBM48585 | 48 | 86.36 | Copepoda | 0.457 | 1.34 | 0.04822 |
| JFBM48585 | 48 | 86.36 | Copepoda | 0.27 | 0.894 | 0.01286 |
| JFBM48585 | 48 | 86.36 | Copepoda | 0.385 | 0.848 | 0.01475 |
| JFBM48585 | 48 | 86.36 | Copepoda | 0.326 | 0.778 | 0.01143 |
| JFBM48585 | 48 | 86.36 | Copepoda | 0.451 | 0.896 | 0.02331 |
| JFBM48585 | 48 | 86.36 | Copepoda | 0.368 | 0.881 | 0.01385 |
| JFBM48585 | 48 | 86.36 | Copepoda | 0.387 |  | 0.03242 |
| JFBM48585 | 48 | 86.36 | Copepoda | 0.337 |  | 0.01927 |
| JFBM48585 | 48 | 86.36 | Copepoda | 0.283 |  | 0.0111 |
| JFBM48585 | 48 | 86.36 | Copepoda | 0.351 | 1.09 | 0.02576 |
| JFBM48585 | 48 | 86.36 | Copepoda | 0.389 |  | 0.0332 |
| JFBM48585 | 48 | 86.36 | Copepoda | 0.339 |  | 0.01985 |
| JFBM48585 | 48 | 86.36 | Copepoda | 0.274 |  | 0.01014 |
| JFBM48585 | 48 | 86.36 | Copepoda | 0.445 | 1.145 | 0.03118 |
| JFBM48585 | 48 | 86.36 | Copepoda | 0.342 |  | 0.02037 |
| JFBM48585 | 48 | 86.36 | Copepoda | 0.327 | 0.768 | 0.02222 |
| JFBM48585 | 48 | 86.36 | Copepoda | 0.343 |  | 0.02068 |
| JFBM48585 | 48 | 86.36 | Copepoda | 0.289 | 0.719 | 0.0154 |
| JFBM48585 | 48 | 86.36 | Copepoda | 0.323 | 0.7 | 0.01062 |
| JFBM48585 | 48 | 86.36 | Copepoda | 0.303 | 0.771 | 0.00972 |
| JFBM48585 | 48 | 86.36 | Copepoda | 0.405 |  | 0.03921 |
| JFBM48585 | 48 | 86.36 | Copepoda | 0.328 |  | 0.01766 |
| JFBM48585 | 48 | 86.36 | Copepoda | 0.279 |  | 0.01067 |
| JFBM48585 | 48 | 86.36 | Copepoda | 0.488 | 0.966 | 0.02352 |
| JFBM48585 | 48 | 86.36 | Copepoda | 0.287 |  | 0.01154 |
| JFBM48585 | 48 | 86.36 | Copepoda | 0.379 |  | 0.02991 |
| JFBM48585 | 48 | 86.36 | Copepoda | 0.301 |  | 0.01336 |
| JFBM48585 | 48 | 86.36 | Copepoda | 0.423 | 1.176 | 0.02733 |
| JFBM48585 | 48 | 86.36 | Copepoda | 0.332 | 0.77 | 0.00858 |
| JFBM48585 | 48 | 86.36 | Copepoda | 0.317 |  | 0.01571 |
| JFBM48585 | 48 | 86.36 | Copepoda | 0.34 |  | 0.01999 |
| JFBM48585 | 48 | 86.36 | Copepoda | 0.375 | 0.8 | 0.01026 |
| JFBM48585 | 48 | 86.36 | Copepoda | 0.434 |  | 0.05267 |
| JFBM48585 | 48 | 86.36 | Copepoda | 0.367 | 0.929 | 0.01917 |
| JFBM48585 | 48 | 86.36 | Copepoda | 0.367 | 0.961 | 0.01744 |
| JFBM48585 | 48 | 86.36 | Copepoda | 0.333 |  | 0.0186 |
| JFBM48585 | 48 | 86.36 | Copepoda | 0.371 |  | 0.02757 |
| JFBM48585 | 49 | 85.27 | Copepoda | 0.315 | 1.21 | 0.02827 |
| JFBM48585 | 49 | 85.27 | Copepoda | 0.342 |  | 0.0203 |
| JFBM48585 | 49 | 85.27 | Copepoda | 0.349 | 0.753 | 0.00955 |
| JFBM48585 | 49 | 85.27 | Copepoda | 0.369 |  | 0.02698 |
| JFBM48585 | 49 | 85.27 | Copepoda | 0.427 |  | 0.04905 |
| JFBM48585 | 49 | 85.27 | Copepoda | 0.382 |  | 0.03097 |
| JFBM48585 | 49 | 85.27 | Copepoda | 0.405 | 1.125 | 0.02593 |
| JFBM48585 | 49 | 85.27 | Copepoda | 0.31 |  | 0.01465 |
| JFBM48585 | 49 | 85.27 | Copepoda | 0.353 |  | 0.0229 |
| JFBM48585 | 49 | 85.27 | Copepoda | 0.303 | 0.688 | 0.00749 |
| JFBM48585 | 49 | 85.27 | Copepoda | 0.437 |  | 0.05416 |
| JFBM48585 | 49 | 85.27 | Copepoda | 0.402 |  | 0.03782 |
| JFBM48585 | 49 | 85.27 | Copepoda | 0.293 | 1.009 | 0.01978 |
| JFBM48585 | 49 | 85.27 | Copepoda | 0.398 |  | 0.03631 |
| JFBM48585 | 49 | 85.27 | Copepoda | 0.479 | 1.212 | 0.02913 |
| JFBM48585 | 49 | 85.27 | Copepoda | 0.407 | 1.067 | 0.02461 |
| JFBM48585 | 49 | 85.27 | Copepoda | 0.338 | 1.007 | 0.02263 |
| JFBM48585 | 49 | 85.27 | Copepoda | 0.355 | 0.804 | 0.0166 |
| JFBM48585 | 49 | 85.27 | Copepoda | 0.322 | 1.026 | 0.01418 |
| JFBM48585 | 49 | 85.27 | Copepoda | 0.409 |  | 0.04071 |
| JFBM48585 | 49 | 85.27 | Copepoda | 0.372 | 0.891 | 0.01263 |
| JFBM48585 | 49 | 85.27 | Copepoda | 0.285 | 0.668 | 0.00607 |
| JFBM48585 | 49 | 85.27 | Copepoda | 0.339 | 0.864 | 0.01186 |
| JFBM48585 | 49 | 85.27 | Copepoda | 0.324 | 0.988 | 0.0131 |
| JFBM48585 | 49 | 85.27 | Copepoda | 0.436 | 0.781 | 0.02317 |
| JFBM48585 | 49 | 85.27 | Copepoda | 0.473 | 0.878 | 0.02578 |
| JFBM48585 | 49 | 85.27 | Copepoda | 0.459 |  | 0.06853 |
| JFBM48585 | 49 | 85.27 | Copepoda | 0.46 |  | 0.06872 |
| JFBM48585 | 49 | 85.27 | Copepoda | 0.33 | 0.75 | 0.01125 |
| JFBM48585 | 49 | 85.27 | Copepoda | 0.426 |  | 0.04841 |
| JFBM48585 | 49 | 85.27 | Copepoda | 0.467 |  | 0.07416 |
| JFBM48585 | 49 | 85.27 | Copepoda | 0.396 |  | 0.03551 |
| JFBM48585 | 49 | 85.27 | Copepoda | 0.349 |  | 0.02192 |
| JFBM48585 | 49 | 85.27 | Copepoda | 0.493 | 1.221 | 0.02949 |
| JFBM48585 | 49 | 85.27 | Copepoda | 0.448 | 1.17 | 0.02923 |
| JFBM48585 | 49 | 85.27 | Copepoda | 0.26 |  | 0.00873 |
| JFBM48585 | 49 | 85.27 | Copepoda | 0.31 |  | 0.01469 |
| JFBM48585 | 49 | 85.27 | Copepoda | 0.287 |  | 0.0116 |
| JFBM48585 | 49 | 85.27 | Copepoda | 0.379 |  | 0.02995 |
| JFBM48585 | 49 | 85.27 | Copepoda | 0.452 |  | 0.06364 |
| JFBM48585 | 49 | 85.27 | Copepoda | 0.293 |  | 0.01224 |
| JFBM48585 | 49 | 85.27 | Copepoda | 0.464 | 1.184 | 0.02595 |
| JFBM48585 | 50 | 84.49 | Copepoda | 0.391 |  | 0.03384 |
| JFBM48585 | 50 | 84.49 | Copepoda | 0.266 | 0.742 | 0.00662 |
| JFBM48585 | 50 | 84.49 | Copepoda | 0.333 |  | 0.01863 |
| JFBM48585 | 50 | 84.49 | Copepoda | 0.319 |  | 0.01603 |
| JFBM48585 | 50 | 84.49 | Copepoda | 0.228 |  | 0.00632 |
| JFBM48585 | 50 | 84.49 | Copepoda | 0.43 |  | 0.05066 |
| JFBM48585 | 50 | 84.49 | Copepoda | 0.294 |  | 0.01238 |
| JFBM48585 | 50 | 84.49 | Copepoda | 0.47 | 0.859 | 0.0211 |
| JFBM48585 | 50 | 84.49 | Copepoda | 0.422 |  | 0.04657 |
| JFBM48585 | 50 | 84.49 | Copepoda | 0.316 | 0.69 | 0.00728 |
| JFBM48585 | 50 | 84.49 | Copepoda | 0.301 |  | 0.01341 |
| JFBM48585 | 50 | 84.49 | Copepoda | 0.565 |  | 0.20294 |
| JFBM48585 | 50 | 84.49 | Copepoda | 0.251 | 0.85 | 0.04768 |
| JFBM48585 | 50 | 84.49 | Copepoda | 0.467 | 0.771 | 0.02366 |
| JFBM48585 | 50 | 84.49 | Copepoda | 0.297 |  | 0.01282 |
| JFBM48585 | 50 | 84.49 | Copepoda | 0.307 |  | 0.01428 |
| JFBM48585 | 50 | 84.49 | Copepoda | 0.362 |  | 0.02494 |
| JFBM48585 | 50 | 84.49 | Copepoda | 0.349 |  | 0.02192 |
| JFBM48585 | 50 | 84.49 | Copepoda | 0.369 |  | 0.02691 |
| JFBM48585 | 50 | 84.49 | Copepoda | 0.338 |  | 0.01957 |
| JFBM48585 | 50 | 84.49 | Copepoda | 0.315 | 0.827 | 0.01241 |
| JFBM48585 | 50 | 84.49 | Copepoda | 0.297 |  | 0.01288 |
| JFBM48585 | 50 | 84.49 | Copepoda | 0.264 |  | 0.00915 |
| JFBM48585 | 50 | 84.49 | Copepoda | 0.349 | 0.799 | 0.0174 |
| JFBM48585 | 50 | 84.49 | Copepoda | 0.259 | 0.785 | 0.00591 |
| JFBM48585 | 50 | 84.49 | Copepoda | 0.456 | 0.841 | 0.00993 |
| JFBM48585 | 50 | 84.49 | Copepoda | 0.263 | 0.795 | 0.01018 |
| JFBM48585 | 50 | 84.49 | Copepoda Calanoida | 0.352 | 0.8 | 0.02507 |
| JFBM48585 | 50 | 84.49 | Copepoda Calanoida | 0.404 | 0.874 | 0.02277 |
| JFBM48585 | 50 | 84.49 | Copepoda Calanoida | 0.259 | 0.484 | 0.00504 |
| JFBM48585 | 50 | 84.49 | Copepoda Calanoida | 0.323 | 0.761 | 0.00832 |
| JFBM48585 | 52 | 73.92 | Copepoda Calanoida | 0.375 | 1.091 | 0.1461 |
| JFBM48585 | 52 | 73.92 | Copepoda | 0.423 |  | 0.04685 |
| JFBM48585 | 52 | 73.92 | Copepoda Calanoida | 0.371 | 0.797 | 0.08883 |
| JFBM48585 | 52 | 73.92 | Copepoda | 0.444 | 0.852 | 0.18723 |
| JFBM48585 | 52 | 73.92 | Copepoda | 0.519 | 0.881 | 0.21324 |
| JFBM48585 | 52 | 73.92 | Copepoda Calanoida | 0.347 | 0.912 | 0.15706 |
| JFBM48585 | 52 | 73.92 | Copepoda | 0.374 |  | 0.0285 |
| JFBM48585 | 52 | 73.92 | Copepoda | 0.467 | 0.811 | 0.20477 |
| JFBM48585 | 52 | 73.92 | Copepoda | 0.434 |  | 0.05246 |
| JFBM48585 | 52 | 73.92 | Copepoda | 0.319 |  | 0.01602 |
| JFBM48585 | 52 | 73.92 | Copepoda | 0.354 |  | 0.02312 |
| JFBM48585 | 52 | 73.92 | Copepoda | 0.283 |  | 0.01111 |
| JFBM48585 | 52 | 73.92 | Copepoda | 0.455 | 0.78 | 0.0889 |
| JFBM48585 | 53 | 85.65 | Copepoda | 0.245 | 0.701 | 0.0984 |
| JFBM48585 | 53 | 85.65 | Copepoda | 0.265 | 0.842 | 0.09754 |
| JFBM48585 | 53 | 85.65 | Copepoda | 0.33 | 0.811 | 0.0765 |
| JFBM48585 | 53 | 85.65 | Copepoda Calanoida | 0.449 | 0.922 | 0.21305 |
| JFBM48585 | 53 | 85.65 | Copepoda Calanoida | 0.381 | 0.716 | 0.09313 |
| JFBM48585 | 53 | 85.65 | Copepoda Calanoida | 0.454 | 0.922 | 0.2388 |
| JFBM48585 | 53 | 85.65 | Copepoda Calanoida | 0.496 | 0.832 | 0.1821 |
| JFBM48585 | 53 | 85.65 | Copepoda Calanoida | 0.362 | 0.974 | 0.11898 |
| JFBM48585 | 53 | 85.65 | Copepoda Calanoida | 0.411 | 0.973 | 0.11596 |
| JFBM48585 | 53 | 85.65 | Copepoda Calanoida | 0.344 | 0.908 | 0.22767 |
| JFBM48585 | 53 | 85.65 | Copepoda Calanoida | 0.411 | 1.128 | 0.21351 |
| JFBM48585 | 53 | 85.65 | Copepoda | 0.283 |  | 0.01114 |
| JFBM48585 | 53 | 85.65 | Copepoda | 0.26 |  | 0.00879 |
| JFBM48606 | 1 | 40.65 | Diptera larvae aquatic | 0.955 |  | 5.18678 |
| JFBM48606 | 1 | 40.65 | Diptera larvae aquatic | 0.351 | 3.638 | 0.03172 |
| JFBM48606 | 1 | 40.65 | Egg | 0.564 | 0.65 | 0.03198 |
| JFBM48606 | 1 | 40.65 | Fish | 8.38 |  | 514.4458 |
| JFBM48606 | 1 | 40.65 | Ostracoda | 0.391 | 0.464 | 0.00855 |
| JFBM48606 | 2 | 39.04 | Seed | 0.086 | 0.245 | 0.0002 |
| JFBM48606 | 2 | 39.04 | Seed | 0.087 | 0.239 | 0.00029 |
| JFBM48606 | 2 | 39.04 | Copepoda | 0.187 | 0.45 | 0.00159 |
| JFBM48606 | 2 | 39.04 | Copepoda | 0.192 | 0.407 | 0.00177 |
| JFBM48606 | 2 | 39.04 | Copepoda Calanoida | 0.193 |  | 0.0044 |
| JFBM48606 | 2 | 39.04 | Copepoda | 0.204 | 0.474 | 0.00206 |
| JFBM48606 | 2 | 39.04 | Copepoda Calanoida | 0.284 |  | 0.01122 |
| JFBM48606 | 2 | 39.04 | Trichoptera larvae aquatic | 0.767 | 4.473 | 0.50592 |
| JFBM48606 | 3 | 34.76 | Egg | 0.148 | 0.174 | 0.00052 |
| JFBM48606 | 3 | 34.76 | Egg | 0.153 | 0.159 | 0.00056 |
| JFBM48606 | 3 | 34.76 | Dinoflagellata | 0.157 | 0.196 | 0.00059 |
| JFBM48606 | 3 | 34.76 | Copepoda | 0.195 | 0.463 | 0.00206 |
| JFBM48606 | 3 | 34.76 | Copepoda | 0.223 |  | 0.006 |
| JFBM48606 | 3 | 34.76 | Diptera larvae aquatic | 0.232 |  | 0.23408 |
| JFBM48606 | 3 | 34.76 | Amphipoda | 0.276 |  | 0.05464 |
| JFBM48606 | 3 | 34.76 | Ostracoda | 0.282 | 0.383 | 0.00481 |
| JFBM48606 | 3 | 34.76 | Ostracoda | 0.289 | 0.451 | 0.00588 |
| JFBM48606 | 3 | 34.76 | Copepoda | 0.317 | 0.572 | 0.00545 |
| JFBM48606 | 3 | 34.76 | Copepoda | 0.322 | 0.563 | 0.00755 |
| JFBM48606 | 3 | 34.76 | Ostracoda | 0.335 | 0.518 | 0.0076 |
| JFBM48606 | 3 | 34.76 | Ostracoda | 0.343 | 0.489 | 0.00711 |
| JFBM48606 | 3 | 34.76 | Ostracoda | 0.346 | 0.46 | 0.0078 |
| JFBM48606 | 3 | 34.76 | Copepoda | 0.368 |  | 0.02658 |
| JFBM48606 | 3 | 34.76 | Ostracoda | 0.422 | 0.561 | 0.01285 |
| JFBM48606 | 4 | 38.59 | Diptera larvae aquatic | 0.086 |  | 0.18757 |
| JFBM48606 | 4 | 38.59 | Diptera larvae aquatic | 0.174 |  | 0.21552 |
| JFBM48606 | 5 | 35.15 | Copepoda | 0.319 | 0.587 | 0.00525 |
| JFBM48606 | 5 | 35.15 | Diptera larvae aquatic | 0.363 | 3.758 | 0.07619 |
| JFBM48606 | 5 | 35.15 | Copepoda Cyclopoida | 0.563 | 0.973 | 0.03261 |
| JFBM48606 | 6 | 38.36 | Copepoda | 0.257 | 0.451 | 0.00526 |
| JFBM48606 | 6 | 38.36 | Copepoda | 0.272 | 0.47 | 0.00351 |
| JFBM48606 | 6 | 38.36 | Copepoda Calanoida | 0.286 | 0.656 | 0.00751 |
| JFBM48606 | 6 | 38.36 | Copepoda | 0.312 |  | 0.01501 |
| JFBM48606 | 6 | 38.36 | Copepoda Calanoida | 0.337 | 0.749 | 0.0087 |
| JFBM48606 | 6 | 38.36 | Copepoda Calanoida | 0.339 | 0.724 | 0.00764 |
| JFBM48606 | 6 | 38.36 | Copepoda | 0.341 |  | 0.02017 |
| JFBM48606 | 6 | 38.36 | Copepoda | 0.357 |  | 0.02378 |
| JFBM48606 | 6 | 38.36 | Cladocera | 0.506 | 0.747 | 0.01626 |
| JFBM48606 | 7 | 29.96 | Copepoda | 0.209 | 0.376 | 0.00218 |
| JFBM48606 | 7 | 29.96 | Copepoda | 0.262 | 0.473 | 0.00442 |
| JFBM48606 | 7 | 29.96 | Copepoda | 0.274 | 0.482 | 0.00518 |
| JFBM48606 | 7 | 29.96 | Copepoda | 0.202 | 0.325 | 0.00063 |
| JFBM48606 | 7 | 29.96 | Copepoda | 0.129 |  | 0.00227 |
| JFBM48606 | 7 | 29.96 | Cladocera | 0.394 | 0.76 | 0.01503 |
| JFBM48606 | 7 | 29.96 | Copepoda | 0.305 | 0.792 | 0.0076 |
| JFBM48606 | 7 | 29.96 | Copepoda | 0.411 | 0.738 | 0.01729 |
| JFBM48606 | 7 | 29.96 | Copepoda | 0.204 | 0.519 | 0.00797 |
| JFBM48606 | 7 | 29.96 | Copepoda | 0.166 | 0.473 | 0.00432 |
| JFBM48606 | 7 | 29.96 | Copepoda | 0.16 | 0.391 | 0.00275 |
| JFBM48606 | 7 | 29.96 | Copepoda | 0.184 | 0.395 | 0.00154 |
| JFBM48606 | 7 | 29.96 | Copepoda | 0.223 |  | 0.006 |
| JFBM48606 | 7 | 29.96 | Cladocera | 0.367 | 0.666 | 0.01119 |
| JFBM48606 | 7 | 29.96 | Copepoda | 0.214 | 0.317 | 0.0029 |
| JFBM48606 | 7 | 29.96 | Cladocera | 0.412 | 0.614 | 0.01147 |
| JFBM48606 | 7 | 29.96 | Copepoda | 0.22 |  | 0.00577 |
| JFBM48606 | 7 | 29.96 | Cladocera | 0.34 | 0.516 | 0.01117 |
| JFBM48606 | 7 | 29.96 | Cladocera | 0.309 | 0.588 | 0.00956 |
| JFBM48606 | 7 | 29.96 | Copepoda | 0.097 | 0.223 | 0.00037 |
| JFBM48606 | 7 | 29.96 | Copepoda | 0.156 | 0.435 | 0.00254 |
| JFBM48606 | 7 | 29.96 | Copepoda | 0.27 | 0.46 | 0.00532 |
| JFBM48606 | 7 | 29.96 | Copepoda | 0.201 | 0.342 | 0.00349 |
| JFBM48606 | 7 | 29.96 | Copepoda | 0.431 | 0.636 | 0.01416 |
| JFBM48606 | 7 | 29.96 | Cladocera | 0.412 | 0.67 | 0.01664 |
| JFBM48606 | 7 | 29.96 | Copepoda | 0.32 |  | 0.01626 |
| JFBM48606 | 7 | 29.96 | Copepoda | 0.291 | 0.556 | 0.01049 |
| JFBM48606 | 7 | 29.96 | Cladocera | 0.405 | 0.748 | 0.0179 |
| JFBM48606 | 7 | 29.96 | Cladocera | 0.457 | 0.734 | 0.02013 |
| JFBM48606 | 7 | 29.96 | Cladocera | 0.367 | 0.707 | 0.00389 |
| JFBM48606 | 7 | 29.96 | Copepoda | 0.296 |  | 0.01273 |
| JFBM48606 | 7 | 29.96 | Copepoda | 0.269 | 0.466 | 0.00303 |
| JFBM48606 | 7 | 29.96 | Copepoda | 0.312 | 0.543 | 0.00766 |
| JFBM48606 | 7 | 29.96 | Copepoda | 0.214 | 0.367 | 0.00073 |
| JFBM48606 | 7 | 29.96 | Copepoda | 0.133 |  | 0.00235 |
| JFBM48606 | 7 | 29.96 | Copepoda | 0.177 |  | 0.00372 |
| JFBM48606 | 7 | 29.96 | Copepoda | 0.244 |  | 0.00743 |
| JFBM48606 | 7 | 29.96 | Cladocera | 0.281 |  | 0.01085 |
| JFBM48606 | 7 | 29.96 | Copepoda | 0.135 | 0.285 | 0.00041 |
| JFBM48606 | 7 | 29.96 | Copepoda | 0.247 | 0.5 | 0.00322 |
| JFBM48606 | 7 | 29.96 | Cladocera | 0.524 | 0.727 | 0.02382 |
| JFBM48606 | 7 | 29.96 | Copepoda | 0.088 |  | 0.00148 |
| JFBM48606 | 7 | 29.96 | Copepoda | 0.232 |  | 0.00657 |
| JFBM48606 | 7 | 29.96 | Copepoda | 0.379 | 0.745 | 0.01448 |
| JFBM48606 | 7 | 29.96 | Copepoda | 0.342 | 0.627 | 0.00666 |
| JFBM48606 | 7 | 29.96 | Copepoda | 0.279 | 0.63 | 0.0097 |
| JFBM48606 | 7 | 29.96 | Copepoda | 0.212 | 0.477 | 0.00222 |
| JFBM48606 | 7 | 29.96 | Copepoda | 0.144 | 0.382 | 0.00117 |
| JFBM48606 | 7 | 29.96 | Copepoda | 0.209 |  | 0.00515 |
| JFBM48606 | 7 | 29.96 | Copepoda | 0.283 |  | 0.0111 |
| JFBM48606 | 7 | 29.96 | Copepoda | 0.186 |  | 0.00408 |
| JFBM48606 | 7 | 29.96 | Copepoda | 0.282 | 0.547 | 0.00442 |
| JFBM48606 | 7 | 29.96 | Copepoda | 0.19 | 0.373 | 0.00188 |
| JFBM48606 | 7 | 29.96 | Copepoda | 0.353 | 0.653 | 0.00802 |
| JFBM48606 | 7 | 29.96 | Copepoda | 0.386 | 0.654 | 0.01059 |
| JFBM48606 | 7 | 29.96 | Copepoda | 0.206 |  | 0.00499 |
| JFBM48606 | 7 | 29.96 | Copepoda | 0.17 |  | 0.00347 |
| JFBM48606 | 7 | 29.96 | Copepoda | 0.231 | 0.485 | 0.00288 |
| JFBM48606 | 7 | 29.96 | Cladocera | 0.271 |  | 0.00978 |
| JFBM48606 | 7 | 29.96 | Egg | 0.107 | 0.17 | 0.00028 |
| JFBM48606 | 7 | 29.96 | Egg | 0.105 | 0.174 | 0.00026 |
| JFBM48606 | 7 | 29.96 | Copepoda | 0.165 | 0.393 | 0.00198 |
| JFBM48606 | 7 | 29.96 | Copepoda | 0.211 |  | 0.00529 |
| JFBM48606 | 7 | 29.96 | Copepoda | 0.217 | 0.411 | 0.00272 |
| JFBM48606 | 7 | 29.96 | Cladocera | 0.365 | 0.604 | 0.01011 |
| JFBM48606 | 7 | 29.96 | Cladocera | 0.442 | 0.772 | 0.01484 |
| JFBM48606 | 7 | 29.96 | Copepoda | 0.378 | 0.679 | 0.01715 |
| JFBM48606 | 7 | 29.96 | Copepoda | 0.353 | 0.674 | 0.00737 |
| JFBM48606 | 7 | 29.96 | Copepoda | 0.256 | 0.683 | 0.00292 |
| JFBM48606 | 7 | 29.96 | Egg | 0.103 | 0.542 | 0.00002 |
| JFBM48606 | 7 | 29.96 | Cladocera | 0.378 |  | 0.02954 |
| JFBM48606 | 7 | 29.96 | Copepoda | 0.18 | 0.454 | 0.00082 |
| JFBM48606 | 7 | 29.96 | Ostracoda | 0.245 | 0.357 | 0.00257 |
| JFBM48606 | 7 | 29.96 | Copepoda | 0.224 | 0.488 | 0.00417 |
| JFBM48606 | 7 | 29.96 | Cladocera | 0.333 |  | 0.01855 |
| JFBM48606 | 7 | 29.96 | Cladocera | 0.323 |  | 0.01676 |
| JFBM48606 | 7 | 29.96 | Copepoda | 0.274 | 0.619 | 0.00418 |
| JFBM48606 | 7 | 29.96 | Copepoda | 0.4 | 0.658 | 0.00906 |
| JFBM48606 | 7 | 29.96 | Copepoda | 0.236 | 0.44 | 0.00316 |
| JFBM48606 | 7 | 29.96 | Copepoda | 0.229 | 0.465 | 0.00447 |
| JFBM48606 | 7 | 29.96 | Egg | 0.102 | 0.163 | 0.00033 |
| JFBM48606 | 7 | 29.96 | Egg | 0.102 | 0.165 | 0.0003 |
| JFBM48606 | 7 | 29.96 | Cladocera | 0.298 | 0.617 | 0.00561 |
| JFBM48606 | 7 | 29.96 | Copepoda | 0.253 |  | 0.00811 |
| JFBM48606 | 7 | 29.96 | Copepoda | 0.188 | 0.447 | 0.00339 |
| JFBM48606 | 7 | 29.96 | Copepoda | 0.191 | 0.489 | 0.00363 |
| JFBM48606 | 7 | 29.96 | Copepoda | 0.172 |  | 0.00354 |
| JFBM48606 | 7 | 29.96 | Copepoda | 0.21 |  | 0.00521 |
| JFBM48606 | 7 | 29.96 | Copepoda | 0.157 | 0.346 | 0.00112 |
| JFBM48606 | 7 | 29.96 | Copepoda | 0.233 | 0.439 | 0.00282 |
| JFBM48606 | 7 | 29.96 | Copepoda | 0.191 | 0.564 | 0.00352 |
| JFBM48606 | 7 | 29.96 | Copepoda | 0.208 |  | 0.00511 |
| JFBM48606 | 7 | 29.96 | Copepoda | 0.199 | 0.418 | 0.00255 |
| JFBM48606 | 7 | 29.96 | Copepoda | 0.181 |  | 0.00388 |
| JFBM48606 | 7 | 29.96 | Copepoda | 0.225 |  | 0.00609 |
| JFBM48606 | 7 | 29.96 | Diptera larvae aquatic | 2.944 | 14.336 | 10.33738 |
| JFBM48606 | 8 | 49.94 | Copepoda | 0.229 |  | 0.00638 |
| JFBM48606 | 8 | 49.94 | Fish | 2.186 |  | 93.81126 |
| JFBM48606 | 8 | 49.94 | Insecta terrestrial | 0.593 | 1.354 | 0.03408 |
| JFBM48606 | 9 | 56.07 | Copepoda | 0.281 |  | 0.01089 |
| JFBM48606 | 10 | 45.94 | Ostracoda | 0.161 |  | 0.00317 |
| JFBM48606 | 11 | 29.17 | Copepoda | 0.266 | 0.679 | 0.00718 |
| JFBM48606 | 11 | 29.17 | Ostracoda | 0.218 | 0.391 | 0.00297 |
| JFBM48606 | 11 | 29.17 | Ostracoda | 0.181 | 0.445 | 0.00196 |
| JFBM48606 | 11 | 29.17 | Ostracoda | 0.251 | 0.471 | 0.00315 |
| JFBM48606 | 11 | 29.17 | Egg | 0.155 | 0.234 | 0.00094 |
| JFBM48606 | 11 | 29.17 | Egg | 0.152 | 0.245 | 0.00087 |
| JFBM48606 | 11 | 29.17 | Egg | 0.13 | 0.252 | 0.00084 |
| JFBM48606 | 11 | 29.17 | Egg | 0.146 | 0.235 | 0.00067 |
| JFBM48606 | 11 | 29.17 | Egg | 0.161 | 0.253 | 0.00121 |
| JFBM48606 | 11 | 29.17 | Egg | 0.131 | 0.242 | 0.00069 |
| JFBM48606 | 11 | 29.17 | Egg | 0.145 | 0.244 | 0.00093 |
| JFBM48606 | 11 | 29.17 | Egg | 0.133 | 0.233 | 0.00079 |
| JFBM48606 | 11 | 29.17 | Egg | 0.146 | 0.237 | 0.00083 |
| JFBM48606 | 11 | 29.17 | Egg | 0.126 | 0.214 | 0.00067 |
| JFBM48606 | 11 | 29.17 | Egg | 0.127 | 0.236 | 0.00066 |
| JFBM48606 | 11 | 29.17 | Egg | 0.135 | 0.263 | 0.00067 |
| JFBM48606 | 11 | 29.17 | Insecta unknown | 0.305 |  | 0.12559 |
| JFBM48606 | 12 | 37.97 | Copepoda | 0.279 | 0.696 | 0.00539 |
| JFBM48606 | 12 | 37.97 | Copepoda | 0.328 | 0.584 | 0.009 |
| JFBM48606 | 12 | 37.97 | Copepoda | 0.342 | 0.776 | 0.01083 |
| JFBM48606 | 12 | 37.97 | Copepoda | 0.408 | 0.726 | 0.01487 |
| JFBM48606 | 12 | 37.97 | Fish | 1.367 |  | 38.22077 |
| JFBM48606 | 13 | 42.9 | Ostracoda | 0.159 | 0.216 | 0.00099 |
| JFBM48606 | 14 | 36.82 | Copepoda | 0.188 |  | 0.00416 |
| JFBM48606 | 14 | 36.82 | Copepoda | 0.193 | 0.321 | 0.00104 |
| JFBM48606 | 14 | 36.82 | Insecta larvae aquatic | 0.206 |  | 0.22584 |
| JFBM48606 | 14 | 36.82 | Copepoda | 0.223 | 0.544 | 0.00276 |
| JFBM48606 | 14 | 36.82 | Egg | 0.223 | 0.321 | 0.00178 |
| JFBM48606 | 14 | 36.82 | Copepoda | 0.246 | 0.471 | 0.0031 |
| JFBM48606 | 14 | 36.82 | Copepoda | 0.247 | 0.659 | 0.00527 |
| JFBM48606 | 14 | 36.82 | Copepoda | 0.255 | 0.472 | 0.00324 |
| JFBM48606 | 14 | 36.82 | Copepoda | 0.257 |  | 0.00849 |
| JFBM48606 | 14 | 36.82 | Egg | 0.26 | 0.445 | 0.00333 |
| JFBM48606 | 14 | 36.82 | Copepoda | 0.264 | 0.409 | 0.00327 |
| JFBM48606 | 14 | 36.82 | Copepoda | 0.272 |  | 0.00986 |
| JFBM48606 | 14 | 36.82 | Copepoda | 0.276 |  | 0.01031 |
| JFBM48606 | 14 | 36.82 | Cladocera | 0.323 | 0.502 | 0.00686 |
| JFBM48606 | 14 | 36.82 | Insecta larvae aquatic | 0.339 |  | 0.02519 |
| JFBM48606 | 14 | 36.82 | Cladocera | 0.362 | 0.572 | 0.00755 |
| JFBM48606 | 14 | 36.82 | Copepoda | 0.438 | 0.763 | 0.01452 |
| JFBM48606 | 14 | 36.82 | Copepoda | 0.549 | 0.744 | 0.02896 |
| JFBM48606 | 15 | 38.19 | Diptera larvae aquatic | 0.389 |  | 0.44887 |
| JFBM48606 | 15 | 38.19 | Diptera larvae aquatic | 0.5 |  | 1.3723 |
| JFBM48606 | 15 | 38.19 | Hymenoptera terrestrial | 0.916 | 4.612 | 0.36613 |
| JFBM48606 | 16 | 35.44 | Egg | 0.14 | 0.272 | 0.00069 |
| JFBM48606 | 17 | 35.44 | Cladocera | 0.525 | 0.634 | 0.02762 |
| JFBM48606 | 18 | 37.22 | Copepoda | 0.282 | 0.559 | 0.00982 |
| JFBM48606 | 19 | 36.66 | Copepoda | 0.495 | 0.933 | 0.02755 |
| JFBM48606 | 19 | 36.66 | Ostracoda | 0.66 | 1.208 | 0.07472 |
| JFBM48606 | 21 | 33.44 | Insecta larvae aquatic | 0.666 |  | 2.7606 |
| JFBM48606 | 26 | 29.95 | Diptera larvae aquatic | 0.557 |  | 1.85332 |
| JFBM48606 | 26 | 29.95 | Diptera pupa aquatic | 0.785 |  | 3.76314 |
| JFBM48606 | 27 | 30.09 | Egg | 0.144 | 0.176 | 0.00049 |
| JFBM48606 | 27 | 30.09 | Copepoda | 0.227 | 0.303 | 0.00243 |
| JFBM48606 | 27 | 30.09 | Cladocera | 0.276 |  | 0.00572 |
| JFBM48606 | 28 | 28.25 | Cladocera | 0.438 | 0.693 | 0.01199 |
| JFBM48606 | 28 | 28.25 | Copepoda | 0.2 | 0.299 | 0.00171 |
| JFBM48606 | 28 | 28.25 | Copepoda | 0.304 | 0.753 | 0.00485 |
| JFBM48606 | 28 | 28.25 | Cladocera | 0.3 |  | 0.01322 |
| JFBM48606 | 28 | 28.25 | Copepoda | 0.208 |  | 0.00509 |
| JFBM48606 | 28 | 28.25 | Copepoda | 0.209 |  | 0.00517 |
| JFBM48606 | 28 | 28.25 | Copepoda | 0.165 |  | 0.00327 |
| JFBM48606 | 28 | 28.25 | Cladocera | 0.173 |  | 0.00357 |
| JFBM48606 | 28 | 28.25 | Copepoda | 0.211 | 0.5 | 0.00284 |
| JFBM48606 | 28 | 28.25 | Copepoda | 0.383 | 0.72 | 0.01134 |
| JFBM48606 | 28 | 28.25 | Cladocera | 0.53 | 0.678 | 0.01217 |
| JFBM48606 | 28 | 28.25 | Copepoda | 0.324 |  | 0.01688 |
| JFBM48606 | 28 | 28.25 | Cladocera | 0.502 | 0.782 | 0.01157 |
| JFBM48606 | 28 | 28.25 | Copepoda | 0.26 | 0.433 | 0.00305 |
| JFBM48606 | 28 | 28.25 | Copepoda | 0.222 |  | 0.0059 |
| JFBM48606 | 28 | 28.25 | Copepoda | 0.162 |  | 0.00319 |
| JFBM48606 | 28 | 28.25 | Copepoda | 0.214 | 0.53 | 0.00158 |
| JFBM48606 | 28 | 28.25 | Copepoda | 0.198 |  | 0.00461 |
| JFBM48606 | 28 | 28.25 | Copepoda | 0.186 |  | 0.00408 |
| JFBM48606 | 28 | 28.25 | Copepoda | 0.409 |  | 0.04084 |
| JFBM48606 | 28 | 28.25 | Copepoda | 0.266 | 0.633 | 0.00494 |
| JFBM48606 | 28 | 28.25 | Copepoda | 0.252 |  | 0.00807 |
| JFBM48606 | 28 | 28.25 | Copepoda | 0.248 | 0.435 | 0.00277 |
| JFBM48606 | 28 | 28.25 | Copepoda | 0.26 | 0.497 | 0.00298 |
| JFBM48606 | 28 | 28.25 | Copepoda | 0.242 |  | 0.00727 |
| JFBM48606 | 28 | 28.25 | Copepoda | 0.281 | 0.488 | 0.00856 |
| JFBM48606 | 28 | 28.25 | Copepoda | 0.234 |  | 0.00672 |
| JFBM48606 | 28 | 28.25 | Copepoda | 0.221 |  | 0.00584 |
| JFBM48606 | 28 | 28.25 | Copepoda | 0.307 | 0.484 | 0.00736 |
| JFBM48606 | 28 | 28.25 | Copepoda | 0.211 | 0.525 | 0.0021 |
| JFBM48606 | 28 | 28.25 | Copepoda | 0.231 | 0.426 | 0.00353 |
| JFBM48606 | 28 | 28.25 | Copepoda | 0.323 | 0.626 | 0.00663 |
| JFBM48606 | 28 | 28.25 | Copepoda | 0.42 |  | 0.04573 |
| JFBM48606 | 28 | 28.25 | Copepoda | 0.169 | 0.32 | 0.00257 |
| JFBM48606 | 28 | 28.25 | Copepoda | 0.242 | 0.462 | 0.0035 |
| JFBM48606 | 28 | 28.25 | Copepoda | 0.235 |  | 0.00675 |
| JFBM48606 | 28 | 28.25 | Copepoda | 0.311 | 0.584 | 0.00739 |
| JFBM48606 | 28 | 28.25 | Cladocera | 0.346 |  | 0.02132 |
| JFBM48606 | 28 | 28.25 | Copepoda | 0.312 | 0.55 | 0.00698 |
| JFBM48606 | 28 | 28.25 | Copepoda | 0.206 | 0.371 | 0.0025 |
| JFBM48606 | 28 | 28.25 | Copepoda | 0.306 | 0.49 | 0.00545 |
| JFBM48606 | 28 | 28.25 | Copepoda | 0.239 |  | 0.00705 |
| JFBM48606 | 28 | 28.25 | Copepoda | 0.236 | 0.435 | 0.00295 |
| JFBM48606 | 28 | 28.25 | Copepoda | 0.252 | 0.467 | 0.00358 |
| JFBM48606 | 28 | 28.25 | Copepoda | 0.184 |  | 0.004 |
| JFBM48606 | 28 | 28.25 | Copepoda | 0.358 | 0.737 | 0.0141 |
| JFBM48606 | 28 | 28.25 | Copepoda | 0.178 |  | 0.00374 |
| JFBM48606 | 28 | 28.25 | Copepoda | 0.301 |  | 0.0133 |
| JFBM48606 | 28 | 28.25 | Copepoda | 0.232 | 0.452 | 0.00261 |
| JFBM48606 | 28 | 28.25 | Copepoda | 0.313 |  | 0.01505 |
| JFBM48606 | 28 | 28.25 | Copepoda | 0.232 |  | 0.00656 |
| JFBM48606 | 28 | 28.25 | Copepoda | 0.193 | 0.403 | 0.00189 |
| JFBM48606 | 28 | 28.25 | Copepoda | 0.268 |  | 0.00955 |
| JFBM48606 | 28 | 28.25 | Copepoda | 0.237 |  | 0.00688 |
| JFBM48606 | 28 | 28.25 | Copepoda | 0.224 | 0.456 | 0.0031 |
| JFBM48606 | 28 | 28.25 | Copepoda | 0.195 |  | 0.00447 |
| JFBM48606 | 28 | 28.25 | Copepoda | 0.27 |  | 0.00972 |
| JFBM48606 | 28 | 28.25 | Copepoda | 0.295 |  | 0.01258 |
| JFBM48606 | 28 | 28.25 | Copepoda | 0.334 | 0.652 | 0.00651 |
| JFBM48606 | 28 | 28.25 | Copepoda | 0.357 |  | 0.02389 |
| JFBM48606 | 28 | 28.25 | Copepoda | 0.366 |  | 0.02611 |
| JFBM48606 | 28 | 28.25 | Copepoda | 0.23 |  | 0.0064 |
| JFBM48606 | 28 | 28.25 | Copepoda | 0.212 | 0.566 | 0.00599 |
| JFBM48607 | 1 | 77.62 | Copepoda | 0.399 | 0.713 | 0.01248 |
| JFBM48607 | 1 | 77.62 | Cladocera | 0.403 | 0.628 | 0.01134 |
| JFBM48607 | 1 | 77.62 | Copepoda | 0.354 | 0.693 | 0.00933 |
| JFBM48607 | 1 | 77.62 | Copepoda | 0.369 | 0.685 | 0.00967 |
| JFBM48607 | 1 | 77.62 | Copepoda | 0.319 | 0.573 | 0.00615 |
| JFBM48607 | 1 | 77.62 | Copepoda | 0.273 | 0.592 | 0.00546 |
| JFBM48607 | 1 | 77.62 | Copepoda | 0.272 | 0.641 | 0.00627 |
| JFBM48607 | 1 | 77.62 | Copepoda | 0.201 |  | 0.00474 |
| JFBM48607 | 1 | 77.62 | Copepoda | 0.455 | 0.832 | 0.0201 |
| JFBM48607 | 1 | 77.62 | Ostracoda | 0.295 | 0.427 | 0.0048 |
| JFBM48607 | 2 | 79.98 | Cladocera | 0.369 | 0.592 | 0.00739 |
| JFBM48607 | 2 | 79.98 | Copepoda | 0.271 |  | 0.00979 |
| JFBM48607 | 2 | 79.98 | Copepoda | 0.381 | 0.68 | 0.01212 |
| JFBM48607 | 2 | 79.98 | Insecta terrestrial | 1.938 |  | 4.05555 |
| JFBM48607 | 2 | 79.98 | Insecta terrestrial | 2.482 |  | 5.36507 |
| JFBM48607 | 2 | 79.98 | Insecta larvae aquatic | 1.458 |  | 9.39346 |
| JFBM48607 | 2 | 79.98 | Insecta larvae aquatic | 1.96 |  | 13.5952 |
| JFBM48607 | 2 | 79.98 | Insecta larvae aquatic | 1.832 |  | 12.52384 |
| JFBM48607 | 2 | 79.98 | Insecta terrestrial | 6.364 |  | 14.70982 |
| JFBM48678 | 1 | 156.17 | Fish Cyprinidae | 5.728 |  | 747.8 |
| JFBM48678 | 2 | 100.93 | Nematoda | 0.073 |  | 0.30496 |
| JFBM48678 | 2 | 100.93 | Insecta terrestrial | 0.524 |  | 0.652 |
| JFBM48678 | 3 | 128.86 | Detritus | 3.378 | 3.727 | 12.59139 |
| JFBM48678 | 3 | 128.86 | Insecta aquatic | 0.713 |  | 6.2957 |
| JFBM48678 | 3 | 128.86 | Insecta aquatic | 1.079 |  | 6.22108 |
| JFBM48678 | 4 | 136.53 | Fish *Notropis volucellus* | 7.287 | 47.652 | 1168.79137 |
| JFBM48678 | 5 | 130.75 | Plant | 1.023 | 1.054 | 1.28946 |
| JFBM48678 | 5 | 130.75 | Fish *Hypophthalmichthys* | 8.487 |  | 1713.45 |
| JFBM48678 | 6 | 123.59 | Fish Cyprinidae | 3.68 |  | 31 |
| JFBM48678 | 7 | 118.96 | Fish unidentifiable | 2.644 |  | 124.91404 |
| JFBM48678 | 7 | 118.96 | Formicidae terrestrial | 1.995 |  | 4.19276 |
| JFBM48678 | 7 | 118.96 | Formicidae terrestrial | 2.189 |  | 4.65976 |
| JFBM48678 | 7 | 118.96 | Formicidae terrestrial | 1.596 |  | 3.23229 |
| JFBM48678 | 7 | 118.96 | Formicidae terrestrial | 2.311 |  | 4.95344 |
| JFBM48678 | 9 | 118.96 | Nematoda | 0.091 | 3.339 | 0.00777 |
| JFBM48678 | 9 | 118.96 | Nematoda | 0.071 | 4.211 | 0.01556 |
| JFBM48678 | 9 | 118.96 | Fish | 1.916 |  | 75.47556 |
| JFBM48678 | 10 | 116.73 | Insecta terrestrial | 1.114 |  | 2.07202 |
| JFBM48678 | 10 | 116.73 | Fish *Notropis* | 11.178 | 50.546 | 5156.54687 |
| JFBM48678 | 10 | 116.73 | Wood | 2.256 | 6.336 | 57.30985 |
| JFBM48678 | 11 | 107.3 | Fish | 7.671 |  | 2222.682 |
| JFBM48678 | 11 | 107.3 | Fish | 4.977 |  | 484.95 |
| JFBM48678 | 11 | 107.3 | Fish *Dorosoma* | 8.856 |  | 4049.952 |
| JFBM48931 | 3 | 71.29 | Hymenoptera terrestrial | 0.447 |  | 0.46642 |
| JFBM48931 | 3 | 71.29 | Hymenoptera terrestrial | 0.342 | 0.971 | 0.12617 |
| JFBM48931 | 3 | 71.29 | Hymenoptera terrestrial | 0.4 | 1.327 | 0.24569 |
| JFBM48931 | 3 | 71.29 | Insecta terrestrial | 0.507 |  | 0.61085 |
| JFBM48931 | 3 | 71.29 | Insecta terrestrial | 0.438 |  | 0.44475 |
| JFBM48931 | 3 | 71.29 | Insecta terrestrial | 0.474 |  | 0.53141 |
| JFBM48931 | 3 | 71.29 | Hymenoptera terrestrial | 0.34 |  | 0.20885 |
| JFBM48931 | 3 | 71.29 | Diptera terrestrial | 0.67 | 1.697 | 0.87804 |
| JFBM48931 | 3 | 71.29 | Insecta terrestrial | 0.462 |  | 0.50253 |
| JFBM48931 | 3 | 71.29 | Insecta terrestrial | 0.299 | 1.128 | 0.22727 |
| JFBM48931 | 3 | 71.29 | Diptera terrestrial | 0.562 | 1.339 | 0.57029 |
| JFBM48931 | 3 | 71.29 | Hymenoptera terrestrial | 0.37 |  | 0.28106 |
| JFBM48931 | 3 | 71.29 | Insecta terrestrial | 0.365 |  | 0.26903 |
| JFBM48931 | 3 | 71.29 | Hymenoptera terrestrial | 0.386 |  | 0.31958 |
| JFBM48931 | 3 | 71.29 | Hymenoptera terrestrial | 0.433 |  | 0.43272 |
| JFBM48931 | 3 | 71.29 | Hymenoptera terrestrial | 0.422 |  | 0.40624 |
| JFBM48931 | 3 | 71.29 | Gastropoda | 0.321 | 0.476 | 0.09634 |
| JFBM48931 | 3 | 71.29 | Hymenoptera terrestrial | 0.38 |  | 0.30514 |
| JFBM48931 | 3 | 71.29 | Insecta unknown | 0.389 |  | 0.3268 |
| JFBM48931 | 3 | 71.29 | Hymenoptera terrestrial | 0.431 |  | 0.4279 |
| JFBM48931 | 3 | 71.29 | Hymenoptera terrestrial | 0.476 |  | 0.53623 |
| JFBM48931 | 3 | 71.29 | Hymenoptera terrestrial | 0.354 | 1.34 | 0.35531 |
| JFBM48931 | 3 | 71.29 | Insecta pupa aquatic | 0.335 |  | 0.19681 |
| JFBM48931 | 3 | 71.29 | Insecta terrestrial | 0.399 |  | 0.35087 |
| JFBM48931 | 3 | 71.29 | Insecta terrestrial | 0.428 |  | 0.42068 |
| JFBM48931 | 3 | 71.29 | Insecta terrestrial | 0.516 |  | 0.63252 |
| JFBM48931 | 3 | 71.29 | Coleoptera terrestrial | 0.996 |  | 1.78797 |
| JFBM48931 | 3 | 71.29 | Diptera larvae aquatic | 0.376 | 1.724 | 0.25506 |
| JFBM48931 | 3 | 71.29 | Insecta unknown | 0.478 |  | 0.54104 |
| JFBM48931 | 3 | 71.29 | Insecta terrestrial | 0.788 |  | 1.28727 |
| JFBM48931 | 3 | 71.29 | Hymenoptera terrestrial | 0.67 |  | 1.00322 |
| JFBM48935 | 1 | 159.04 | Fish | 9.703 |  | 2139.05 |
| JFBM48935 | 1 | 159.04 | Trichoptera larvae aquatic | 1.262 |  | 7.75294 |
| JFBM48947 | 1 | 122.23 | Hymenoptera terrestrial | 1.161 |  | 2.18516 |
| JFBM48947 | 1 | 122.23 | Hymenoptera terrestrial | 1.113 |  | 2.06961 |
| JFBM48947 | 1 | 122.23 | Hymenoptera terrestrial | 1.176 |  | 2.22127 |
| JFBM48947 | 1 | 122.23 | Hymenoptera terrestrial | 1.027 |  | 1.86259 |
| JFBM48947 | 1 | 122.23 | Hymenoptera terrestrial | 1.051 |  | 1.92037 |
| JFBM48947 | 1 | 122.23 | Hymenoptera terrestrial | 1.383 |  | 2.71956 |
| JFBM48947 | 1 | 122.23 | Hymenoptera terrestrial | 1.269 |  | 2.44514 |
| JFBM48947 | 1 | 122.23 | Hymenoptera terrestrial | 0.999 |  | 1.79519 |
| JFBM48947 | 1 | 122.23 | Hymenoptera terrestrial | 0.916 |  | 1.5954 |
| JFBM48947 | 1 | 122.23 | Hymenoptera terrestrial | 1.179 |  | 2.22849 |
| JFBM48947 | 1 | 122.23 | Trichoptera larvae aquatic | 0.979 | 6.216 | 5.71571 |
| JFBM48947 | 2 | 124.8 | Copepoda | 1.681 | 2.535 | 6.75814 |
| JFBM48947 | 3 | 108.95 | Hymenoptera terrestrial | 0.841 |  | 4.22917 |
| JFBM48947 | 3 | 108.95 | Hymenoptera terrestrial | 0.963 |  | 5.25031 |
| JFBM48947 | 3 | 108.95 | Hymenoptera terrestrial | 1.147 |  | 6.79039 |
| JFBM48947 | 3 | 108.95 | Hymenoptera terrestrial | 1.199 |  | 7.22563 |
| JFBM48947 | 3 | 108.95 | Hymenoptera terrestrial | 1.064 |  | 6.09568 |
| JFBM48947 | 4 | 105.38 | Copepoda | 1.129 |  | 2.10828 |
| JFBM48947 | 4 | 105.38 | Fish *Hypophthalmichthys* | 7.375 |  | 1324.25 |
| JFBM48947 | 4 | 105.38 | Fish unidentifiable | 9.258 |  | 1983.3 |
| JFBM48947 | 5 | 119.54 | Fish *Hypophthalmichthys* | 9.92 |  | 2215 |
| JFBM48947 | 5 | 119.54 | Fish *Hypophthalmichthys* | 10.844 |  | 2538.4 |
| JFBM48947 | 5 | 119.54 | Fish Hypophthalmichthys | 8.025 |  | 1551.75 |
| JFBM48947 | 6 | 116.25 | Fish unidentifiable | 4.128 |  | 187.8 |
| JFBM48947 | 7 | 130.82 | Fish unidentifiable | 4.035 |  | 155.25 |
| JFBM48947 | 7 | 130.82 | Fish unidentifiable | 4.142 |  | 192.7 |
| JFBM48947 | 8 | 109.94 | Fish unidentifiable | 7.368 |  | 1321.8 |
| JFBM48947 | 9 | 89.93 | Fish *Hypophthalmichthys* | 6.415 | 25.723 | 611.15568 |
| JFBM48947 | 9 | 89.93 | Fish *Hypophthalmichthys* | 7.196 | 30.416 | 1203.57786 |
| JFBM48947 | 9 | 89.93 | Fish *Hypopthalmichthys* | 6.791 |  | 865.722 |
| JFBM48947 | 9 | 89.93 | Fish (unidentifiable) | 7.017 |  | 1214.214 |
| JFBM48947 | 9 | 89.93 | Formicidae terrestrial | 1.402 |  | 2.76529 |
| JFBM48948 | 1 | 93.52 | Fish *Hypophthalmichthys* | 6.676 |  | 1079.6 |
| JFBM48948 | 1 | 93.52 | Fish *Hypophthalmichthys* | 7.553 |  | 1386.55 |
| JFBM48948 | 1 | 93.52 | Fish *Hypophthalmichthys* | 10.212 |  | 2317.2 |
| JFBM48948 | 1 | 93.52 | Fish *Hypophthalmichthys* | 7.107 |  | 1230.45 |
| JFBM48948 | 1 | 116.4 | Fish *Hypophthalmichthys* | 8.326 |  | 1657.1 |
| JFBM48948 | 2 | 116.4 | Fish unidentifiable | 8.454 |  | 1701.9 |
| JFBM48948 | 3 | 116.4 | Fish not *Hypophthalmichthys* | 7.241 |  | 1277.35 |
| JFBM48948 | 4 | 69.04 | Copepoda | 0.282 | 0.844 | 0.07966 |
| JFBM48948 | 4 | 69.04 | Copepoda | 0.472 | 0.721 | 0.16281 |
| JFBM48948 | 4 | 69.04 | Copepoda | 0.353 |  | 0.02288 |
| JFBM48948 | 4 | 69.04 | Nematoda | 0.046 |  | 0.00096 |
| JFBM48948 | 4 | 116.4 | Fish unidentifiable | 7.517 |  | 1373.95 |
| JFBM48948 | 7 | 44.88 | Copepoda | 0.271 | 0.683 | 0.03609 |
| JFBM48948 | 8 | 123.35 | Fish *Hypophthalmichthys* | 6.957 |  | 1177.95 |
| JFBM48948 | 8 | 123.35 | Fish *Hypophthalmichthys* | 9.062 |  | 1914.7 |
| JFBM48948 | 8 | 123.35 | Fish *Hypophthalmichthys* | 8.878 |  | 1850.3 |
| JFBM48948 | 8 | 123.35 | Fish *Hypophthalmichthys* | 9.769 |  | 2162.15 |
| JFBM48948 | 9 | 120.25 | Fish unidentifiable | 9.733 |  | 2149.55 |
| JFBM48948 | 11 | 78.3 | Insecta unknown | 1.23 |  | 2.35126 |
| JFBM48948 | 11 | 78.3 | Plant | 0.121 | 1.646 | 0.01563 |
| JFBM48948 | 13 | 51.65 | Insecta larvae aquatic | 0.215 | 1.349 | 0.08284 |
| JFBM48948 | 13 | 51.65 | Insecta larvae aquatic | 0.164 |  | 0.00323 |
| JFBM48948 | 13 | 51.65 | Insecta larvae aquatic | 0.139 | 1.232 | 0.02196 |
| JFBM48948 | 13 | 51.65 | Insecta larvae aquatic | 0.226 | 1.981 | 0.04018 |
| JFBM48948 | 13 | 51.65 | Copepoda | 0.18 | 0.365 | 0.02377 |
| JFBM48948 | 13 | 51.65 | Insecta terrestrial | 0.833 | 1.363 | 1.0841 |
| JFBM48948 | 13 | 51.65 | Insecta terrestrial | 0.391 | 1.041 | 0.29797 |
| JFBM48948 | 13 | 51.65 | Insecta terrestrial | 0.721 | 2.118 | 1.0032 |
| JFBM48948 | 13 | 51.65 | Insecta terrestrial | 0.537 | 1.81 | 0.92283 |
| JFBM48948 | 13 | 51.65 | Insecta terrestrial | 1.837 | 5.765 | 20.23621 |
| JFBM48948 | 13 | 51.65 | Insecta terrestrial | 0.297 |  | 0.10534 |
| JFBM48948 | 13 | 51.65 | Insecta terrestrial | 0.286 |  | 0.07886 |
| JFBM48948 | 13 | 51.65 | Insecta terrestrial | 0.424 |  | 0.41105 |
| JFBM48948 | 13 | 51.65 | Insecta terrestrial | 0.278 |  | 0.0596 |
| JFBM48948 | 13 | 51.65 | Insecta terrestrial | 0.267 |  | 0.03312 |
| JFBM48948 | 13 | 51.65 | Insecta terrestrial | 0.609 |  | 0.85638 |
| JFBM48948 | 13 | 51.65 | Insecta terrestrial | 0.339 |  | 0.20644 |
| JFBM48948 | 13 | 51.65 | Insecta terrestrial | 0.201 |  | 0.00476 |
| JFBM48948 | 13 | 51.65 | Insecta terrestrial | 0.275 |  | 0.05238 |
| JFBM48948 | 13 | 51.65 | Insecta terrestrial | 0.387 |  | 0.32199 |
| JFBM48948 | 13 | 51.65 | Insecta terrestrial | 0.316 |  | 0.15108 |
| JFBM48948 | 13 | 51.65 | Insecta terrestrial | 0.354 | 0.476 | 0.05391 |
| JFBM48948 | 13 | 51.65 | Insecta terrestrial | 0.525 | 0.704 | 0.17466 |
| JFBM48948 | 13 | 51.65 | Insecta terrestrial | 0.354 | 0.501 | 0.05723 |
| JFBM48948 | 14 | 58.66 | Copepoda | 0.243 | 0.529 | 0.02536 |
| JFBM48948 | 14 | 58.66 | Copepoda | 0.315 | 0.703 | 0.04581 |
| JFBM48948 | 14 | 58.66 | Copepoda | 0.396 | 0.915 | 0.12906 |
| JFBM48948 | 15 | 109.64 | Fish unidentifiable | 8.435 |  | 1695.25 |
| JFBM48948 | 15 | 109.64 | Fish unidentifiable | 5.88 |  | 801 |
| JFBM48948 | 16 | 107.49 | Fish *Hypophthalmichthys* | 9.106 |  | 1930.1 |
| JFBM48948 | 16 | 107.49 | Fish *Hypophthalmichthys* | 7.801 |  | 1473.35 |
| JFBM48948 | 16 | 107.49 | Fish *Hypophthalmichthys* | 8.888 |  | 1853.8 |
| JFBM48948 | 16 | 107.49 | Fish *Hypophthalmichthys* | 7.29 |  | 1294.5 |
| JFBM48948 | 16 | 107.49 | Fish *Hypophthalmichthys* | 9.011 |  | 1896.85 |
| JFBM48948 | 17 | 90.48 | Insecta larvae aquatic | 0.447 | 4.787 | 1.02218 |
| JFBM48948 | 17 | 90.48 | Insecta larvae aquatic | 0.426 | 5.289 | 0.71952 |
| JFBM48948 | 17 | 90.48 | Insecta larvae aquatic | 0.436 | 5.008 | 0.93561 |
| JFBM48948 | 18 | 115.98 | Fish *Hypophthalmichthys* | 11.463 |  | 2755.05 |
| JFBM48948 | 18 | 115.98 | Fish *Hypophthalmichthys* | 7.164 |  | 1250.4 |
| JFBM48948 | 18 | 115.98 | Fish *Hypophthalmichthys* | 7.404 |  | 1334.4 |
| JFBM48948 | 18 | 115.98 | Fish *Hypophthalmichthys* | 8.102 |  | 1578.7 |
| JFBM48948 | 18 | 115.98 | Fish *Hypophthalmichthys* | 7.426 |  | 1342.1 |
| JFBM48948 | 19 | 117.42 | Fish *Hypophthalmichthys* | 9.879 |  | 2200.65 |
| JFBM48948 | 19 | 117.42 | Fish *Hypophthalmichthys* | 9.032 |  | 1904.2 |
| JFBM48948 | 19 | 117.42 | Fish *Hypophthalmichthys* | 7.625 |  | 1411.75 |
| JFBM48948 | 19 | 117.42 | Fish *Hypophthalmichthys* | 9.277 |  | 1989.95 |
| JFBM48948 | 19 | 117.42 | Fish *Hypophthalmichthys* | 7.19 |  | 1259.5 |
| JFBM48948 | 19 | 117.42 | Fish *Hypophthalmichthys* | 9.307 |  | 2000.45 |
| JFBM48948 | 22 | 91.79 | Insecta terrestrial | 1.97 |  | 4.13258 |
| JFBM48948 | 22 | 91.79 | Fish unidentifiable | 9.672 |  | 2128.2 |
| JFBM48948 | 22 | 91.79 | Fish *Hypophthalmichthys* | 10.267 |  | 2336.45 |
| JFBM48948 | 24 | 49.45 | Copepoda | 0.246 | 0.561 | 0.00438 |
| JFBM48948 | 24 | 49.45 | Copepoda | 0.218 |  | 0.00569 |
| JFBM48948 | 24 | 49.45 | Copepoda | 0.3 |  | 0.01318 |
| JFBM48948 | 24 | 49.45 | Copepoda | 0.215 | 0.548 | 0.01293 |
| JFBM48948 | 24 | 49.45 | Copepoda | 0.268 | 0.607 | 0.08291 |
| JFBM48948 | 24 | 49.45 | Copepoda | 0.241 | 0.619 | 0.05619 |
| JFBM48948 | 24 | 49.45 | Ostracoda | 0.366 | 0.531 | 0.06915 |
| JFBM48948 | 25 | 50.99 | Copepoda | 0.259 | 0.655 | 0.04192 |
| JFBM48948 | 25 | 50.99 | Copepoda | 0.217 | 0.707 | 0.02993 |
| JFBM48948 | 25 | 50.99 | Copepoda | 0.19 | 0.31 | 0.00945 |
| JFBM48948 | 25 | 50.99 | Copepoda | 0.288 | 0.838 | 0.09163 |
| JFBM48948 | 25 | 50.99 | Copepoda | 0.498 | 0.734 | 0.16247 |
| JFBM48948 | 25 | 50.99 | Chironomidae larvae aquatic | 0.319 | 3.617 | 0.3639 |
| JFBM48948 | 25 | 50.99 | Insecta terrestrial | 1.221 |  | 2.32959 |
| JFBM48948 | 25 | 50.99 | Insecta terrestrial | 1.317 |  | 2.56068 |
| JFBM48948 | 25 | 50.99 | Insecta terrestrial | 1.306 |  | 2.5342 |
| JFBM48948 | 25 | 50.99 | Ostracoda | 0.371 | 0.498 | 0.07977 |
| JFBM48948 | 25 | 50.99 | Ostracoda | 0.256 | 0.391 | 0.03013 |
| JFBM48948 | 25 | 50.99 | Plant | 4.691 | 6.039 | 178.68773 |
| JFBM48948 | 26 | 45.69 | Ostracoda | 0.409 | 0.55 | 0.10245 |
| JFBM48948 | 26 | 45.69 | Ostracoda | 0.286 | 0.408 | 0.05508 |
| JFBM48948 | 26 | 45.69 | Ostracoda | 0.319 | 0.37 | 0.0552 |
| JFBM48948 | 26 | 45.69 | Ostracoda | 0.41 | 0.523 | 0.11306 |
| JFBM48948 | 27 | 53.68 | Copepoda | 0.249 | 0.512 | 0.05057 |
| JFBM48948 | 27 | 53.68 | Copepoda | 0.308 | 0.725 | 0.0689 |
| JFBM48948 | 27 | 53.68 | Copepoda | 0.301 | 0.674 | 0.07119 |
| JFBM48948 | 27 | 53.68 | Copepoda | 0.264 | 0.87 | 0.05949 |
| JFBM48948 | 27 | 53.68 | Copepoda | 0.372 | 0.746 | 0.13168 |
| JFBM48948 | 27 | 53.68 | Copepoda | 0.28 | 0.739 | 0.06315 |
| JFBM48948 | 27 | 53.68 | Copepoda | 0.262 |  | 0.00891 |
| JFBM48948 | 27 | 53.68 | Copepoda | 0.247 |  | 0.00762 |
| JFBM48948 | 27 | 53.68 | Copepoda | 0.358 | 0.723 | 0.1322 |
| JFBM48948 | 27 | 53.68 | Copepoda | 0.405 | 0.786 | 0.13625 |
| JFBM48948 | 27 | 53.68 | Copepoda | 0.292 |  | 0.01218 |
| JFBM48948 | 27 | 53.68 | Copepoda | 0.211 |  | 0.00526 |
| JFBM48948 | 27 | 53.68 | Ostracoda | 0.294 | 0.399 | 0.029 |
| JFBM48948 | 27 | 53.68 | Copepoda | 0.233 |  | 0.00665 |
| JFBM48948 | 27 | 53.68 | Copepoda | 0.177 |  | 0.00372 |
| JFBM48948 | 27 | 53.68 | Copepoda | 0.382 | 0.547 | 0.09523 |
| JFBM48948 | 27 | 53.68 | Copepoda | 0.241 |  | 0.00722 |
| JFBM48948 | 27 | 53.68 | Copepoda | 0.546 |  | 0.16798 |
| JFBM48948 | 27 | 53.68 | Ostracoda | 0.353 | 0.492 | 0.06535 |
| JFBM48948 | 28 | 47.25 | Copepoda | 0.187 |  | 0.00413 |
| JFBM48948 | 28 | 47.25 | Cladocera | 0.217 |  | 0.00564 |
| JFBM48948 | 28 | 47.25 | Copepoda | 0.222 | 0.69 | 0.05295 |
| JFBM48948 | 28 | 47.25 | Copepoda | 0.341 | 0.797 | 0.05095 |
| JFBM48948 | 28 | 47.25 | Copepoda | 0.309 | 0.773 | 0.04804 |
| JFBM48948 | 28 | 47.25 | Copepoda | 0.33 |  | 0.01802 |
| JFBM48948 | 28 | 47.25 | Copepoda | 0.289 |  | 0.01174 |
| JFBM48948 | 28 | 47.25 | Copepoda | 0.319 | 0.803 | 0.05473 |
| JFBM48948 | 28 | 47.25 | Copepoda | 0.317 |  | 0.01575 |
| JFBM48948 | 28 | 47.25 | Copepoda | 0.377 | 0.66 | 0.12472 |
| JFBM48948 | 28 | 47.25 | Copepoda | 0.325 | 0.696 | 0.09689 |
| JFBM48948 | 28 | 47.25 | Copepoda | 0.336 |  | 0.0192 |
| JFBM48948 | 28 | 47.25 | Copepoda | 0.304 |  | 0.01378 |
| JFBM48948 | 28 | 47.25 | Copepoda | 0.372 | 0.525 | 0.0667 |
| JFBM48948 | 28 | 47.25 | Copepoda | 0.386 | 0.535 | 0.07637 |
| JFBM48948 | 28 | 47.25 | Copepoda | 0.181 | 0.424 | 0.02851 |
| JFBM48948 | 28 | 47.25 | Ostracoda | 0.344 | 0.486 | 0.06865 |
| JFBM48948 | 28 | 47.25 | Copepoda | 0.303 |  | 0.0137 |
| JFBM48948 | 28 | 47.25 | Ostracoda | 0.412 | 0.551 | 0.09899 |
| JFBM48948 | 28 | 47.25 | Ostracoda | 0.435 | 0.616 | 0.10882 |
| JFBM48948 | 28 | 47.25 | Copepoda | 0.281 |  | 0.01085 |
| JFBM48948 | 29 | 123.03 | Fish *Hypophthalmichthys* | 10.319 |  | 2354.65 |
| JFBM48948 | 29 | 123.03 | Fish *Hypophthalmichthys* | 7.346 |  | 1721.532 |
| JFBM48948 | 29 | 123.03 | Fish *Hypophthalmichthys* | 6.896 |  | 1156.6 |
| JFBM48948 | 29 | 123.03 | Fish not *Hypophthalmichthys* | 10.353 |  | 2366.55 |
| JFBM48948 | 30 | 96.91 | Fish *Hypophthalmichthys* | 4.805 |  | 424.75 |
| JFBM48948 | 30 | 96.91 | Fish *Hypophthalmichthys* | 5.928 |  | 817.8 |
| JFBM48948 | 30 | 96.91 | Fish *Hypophthalmichthys* | 5.046 |  | 509.1 |
| JFBM48948 | 30 | 96.91 | Fish *Hypophthalmichthys* | 8.173 |  | 1603.55 |
| JFBM48948 | 30 | 96.91 | Fish *Hypophthalmichthys* | 6.345 |  | 963.75 |
| JFBM48948 | 31 | 123.03 | Fish *Hypophthalmichthys* | 6.883 |  | 1007.586 |
| JFBM48948 | 31 | 123.03 | Fish *Hypophthalmichthys* | 7.849 |  | 2497.158 |
| JFBM48948 | 32 | 101.57 | Fish *Hypophthalmichthys* | 10.722 |  | 2495.7 |
| JFBM48948 | 32 | 101.57 | Fish unidentifiable | 5.711 |  | 741.85 |
| JFBM48948 | 32 | 101.57 | Fish | 11.852 |  | 8669.784 |
| JFBM48948 | 33 | 114.82 | Fish | 8.416 |  | 3371.472 |
| JFBM48948 | 33 | 114.82 | Fish *Hypophthalmichthys* | 8.93 |  | 1868.5 |
| JFBM48948 | 33 | 114.82 | Fish | 8.627 |  | 1762.45 |
| JFBM48948 | 33 | 114.82 | Fish | 7.002 |  | 1193.7 |
| JFBM48948 | 33 | 114.82 | Fish *Hypophthalmichthys* | 9.583 |  | 2097.05 |
| JFBM48948 | 34 | 95.55 | Fish *Hypophthalmichthys* | 9.154 |  | 4509.468 |
| JFBM48948 | 35 | 98.01 | Fish unidentifiable | 5.293 |  | 595.55 |
| JFBM48948 | 35 | 98.01 | Fish *Hypophthalmichthys* | 7.485 |  | 1362.75 |
| JFBM48948 | 35 | 98.01 | Fish *Hypophthalmichthys* | 6.158 |  | 898.3 |
| JFBM48948 | 37 | 55.45 | Ostracoda | 0.322 | 0.538 | 0.05343 |
| JFBM48948 | 37 | 55.45 | Ostracoda | 0.353 | 0.526 | 0.06223 |
| JFBM48948 | 38 | 46.02 | Copepoda | 0.188 |  | 0.00417 |
| JFBM48948 | 38 | 46.02 | Copepoda | 0.235 |  | 0.00674 |
| JFBM48948 | 38 | 46.02 | Copepoda | 0.187 | 0.501 | 0.01476 |
| JFBM48948 | 38 | 46.02 | Copepoda | 0.262 | 0.732 | 0.06367 |
| JFBM48948 | 38 | 46.02 | Copepoda | 0.208 | 0.55 | 0.0396 |
| JFBM48948 | 38 | 46.02 | Copepoda | 0.347 | 0.717 | 0.06423 |
| JFBM48948 | 38 | 46.02 | Copepoda | 0.224 | 0.796 | 0.05901 |
| JFBM48948 | 38 | 46.02 | Copepoda | 0.332 | 0.825 | 0.07783 |
| JFBM48948 | 38 | 46.02 | Copepoda | 0.256 |  | 0.00841 |
| JFBM48948 | 38 | 46.02 | Copepoda | 0.249 | 0.574 | 0.02399 |
| JFBM48948 | 38 | 46.02 | Copepoda | 0.262 | 0.578 | 0.06126 |
| JFBM48948 | 38 | 46.02 | Copepoda | 0.22 | 0.667 | 0.04068 |
| JFBM48948 | 38 | 46.02 | Copepoda | 0.261 | 0.719 | 0.04178 |
| JFBM48948 | 38 | 46.02 | Copepoda | 0.209 |  | 0.00519 |
| JFBM48948 | 38 | 46.02 | Copepoda | 0.227 |  | 0.00623 |
| JFBM48948 | 38 | 46.02 | Copepoda | 0.218 |  | 0.00567 |
| JFBM48948 | 38 | 46.02 | Copepoda | 0.206 | 0.491 | 0.01427 |
| JFBM48948 | 38 | 46.02 | Copepoda | 0.189 |  | 0.00422 |
| JFBM48948 | 38 | 46.02 | Copepoda | 0.221 |  | 0.00584 |
| JFBM48948 | 38 | 46.02 | Copepoda | 0.356 |  | 0.02351 |
| JFBM48948 | 38 | 46.02 | Copepoda | 0.225 |  | 0.00608 |
| JFBM48948 | 38 | 46.02 | Copepoda | 0.241 | 0.681 | 0.04957 |
| JFBM48948 | 38 | 46.02 | Copepoda | 0.249 | 0.648 | 0.1445 |
| JFBM48948 | 38 | 46.02 | Copepoda | 0.168 | 0.45 | 0.02596 |
| JFBM48948 | 38 | 46.02 | Copepoda | 0.272 |  | 0.00991 |
| JFBM48948 | 38 | 46.02 | Copepoda | 0.18 |  | 0.00385 |
| JFBM48948 | 38 | 46.02 | Copepoda | 0.234 | 0.491 | 0.03731 |
| JFBM48948 | 38 | 46.02 | Copepoda | 0.22 | 0.68 | 0.04227 |
| JFBM48948 | 38 | 46.02 | Copepoda | 0.268 | 0.734 | 0.08099 |
| JFBM48948 | 38 | 46.02 | Copepoda | 0.238 | 0.643 | 0.07215 |
| JFBM48948 | 38 | 46.02 | Copepoda | 0.186 | 0.517 | 0.0229 |
| JFBM48948 | 38 | 46.02 | Copepoda | 0.308 | 0.703 | 0.05398 |
| JFBM48948 | 38 | 46.02 | Copepoda | 0.233 | 0.594 | 0.03931 |
| JFBM48948 | 38 | 46.02 | Copepoda | 0.249 |  | 0.00781 |
| JFBM48948 | 38 | 46.02 | Copepoda | 0.255 | 0.75 | 0.07437 |
| JFBM48948 | 38 | 46.02 | Copepoda | 0.236 |  | 0.00687 |
| JFBM48948 | 38 | 46.02 | Copepoda | 0.257 |  | 0.00849 |
| JFBM48948 | 38 | 46.02 | Copepoda | 0.263 |  | 0.00904 |
| JFBM48948 | 38 | 46.02 | Copepoda | 0.232 |  | 0.00658 |
| JFBM48948 | 38 | 46.02 | Copepoda | 0.204 | 0.683 | 0.04745 |
| JFBM48948 | 38 | 46.02 | Copepoda | 0.284 | 0.744 | 0.04929 |
| JFBM48948 | 38 | 46.02 | Copepoda | 0.2 |  | 0.00469 |
| JFBM48948 | 38 | 46.02 | Copepoda | 0.219 |  | 0.00574 |
| JFBM48948 | 38 | 46.02 | Copepoda | 0.261 |  | 0.00883 |
| JFBM48948 | 38 | 46.02 | Copepoda | 0.365 |  | 0.02575 |
| JFBM48948 | 38 | 46.02 | Copepoda | 0.273 | 0.685 | 0.03444 |
| JFBM48948 | 38 | 46.02 | Copepoda | 0.249 | 0.876 | 0.0471 |
| JFBM48948 | 38 | 46.02 | Copepoda | 0.21 |  | 0.00524 |
| JFBM48948 | 38 | 46.02 | Copepoda | 0.375 | 0.796 | 0.11679 |
| JFBM48948 | 38 | 46.02 | Copepoda | 0.31 |  | 0.01467 |
| JFBM48948 | 38 | 46.02 | Copepoda | 0.268 | 0.645 | 0.07016 |
| JFBM48948 | 38 | 46.02 | Copepoda | 0.274 |  | 0.01009 |
| JFBM48948 | 38 | 46.02 | Copepoda | 0.208 |  | 0.00512 |
| JFBM48948 | 39 | 51.01 | Copepoda | 0.196 |  | 0.0045 |
| JFBM48948 | 39 | 51.01 | Copepoda | 0.226 |  | 0.00616 |
| JFBM48948 | 39 | 51.01 | Copepoda | 0.215 |  | 0.00549 |
| JFBM48948 | 39 | 51.01 | Copepoda | 0.171 |  | 0.00351 |
| JFBM48948 | 39 | 51.01 | Copepoda | 0.183 |  | 0.00396 |
| JFBM48948 | 39 | 51.01 | Copepoda | 0.216 |  | 0.00555 |
| JFBM48948 | 39 | 51.01 | Copepoda | 0.202 | 0.476 | 0.02083 |
| JFBM48948 | 39 | 51.01 | Copepoda | 0.218 |  | 0.00566 |
| JFBM48948 | 39 | 51.01 | Copepoda | 0.199 |  | 0.00466 |
| JFBM48948 | 39 | 51.01 | Copepoda | 0.221 | 0.738 | 0.02886 |
| JFBM48948 | 39 | 51.01 | Copepoda | 0.296 | 0.864 | 0.03738 |
| JFBM48948 | 39 | 51.01 | Copepoda | 0.16 |  | 0.00311 |
| JFBM48948 | 39 | 51.01 | Copepoda | 0.204 |  | 0.00493 |
| JFBM48948 | 39 | 51.01 | Copepoda | 0.204 |  | 0.0049 |
| JFBM48948 | 39 | 51.01 | Copepoda | 0.187 |  | 0.00413 |
| JFBM48948 | 39 | 51.01 | Ostracoda | 0.313 | 0.456 | 0.04429 |
| JFBM48948 | 39 | 51.01 | Copepoda | 0.197 | 0.579 | 0.02939 |
| JFBM48948 | 40 | 47.82 | Copepoda | 0.21 | 0.505 | 0.02449 |
| JFBM48948 | 40 | 47.82 | Copepoda | 0.246 |  | 0.00755 |
| JFBM48948 | 40 | 47.82 | Copepoda | 0.213 |  | 0.00539 |
| JFBM48948 | 40 | 47.82 | Copepoda | 0.248 |  | 0.00773 |
| JFBM48948 | 40 | 47.82 | Copepoda | 0.226 |  | 0.00616 |
| JFBM48948 | 40 | 47.82 | Copepoda | 0.211 | 0.708 | 0.03816 |
| JFBM48948 | 40 | 47.82 | Copepoda | 0.308 | 0.484 | 0.0429 |
| JFBM48948 | 40 | 47.82 | Copepoda | 0.166 |  | 0.00332 |
| JFBM48948 | 40 | 47.82 | Copepoda | 0.283 | 0.679 | 0.05795 |
| JFBM48948 | 40 | 47.82 | Copepoda | 0.347 | 0.832 | 0.0807 |
| JFBM48948 | 40 | 47.82 | Ostracoda | 0.309 | 0.444 | 0.0455 |
| JFBM48948 | 40 | 47.82 | Copepoda | 0.198 |  | 0.0046 |
| JFBM48948 | 40 | 47.82 | Ostracoda | 0.421 | 0.575 | 0.08396 |
| JFBM48948 | 41 | 104.25 | Fish *Hypophthalmichthys* | 7.564 |  | 2057.688 |
| JFBM48948 | 42 | 102.55 | Fish *Hypophthalmichthys* | 6.237 |  | 925.95 |
| JFBM48948 | 44 | 126.91 | Fish *Hypophthalmichthys* | 9.216 |  | 1968.6 |
| JFBM48948 | 44 | 126.91 | Fish *Hypophthalmichthys* | 8.573 |  | 1743.55 |
| JFBM48948 | 45 | 118.07 | Fish *Hypophthalmichthys* | 9.97 | 37.513 | 5767.74 |
| JFBM48948 | 45 | 118.07 | Fish *Hypophthalmichthys* | 8.517 |  | 3527.214 |
| JFBM48948 | 46 | 105.7 | Fish *Hypophthalmichthys* | 8.552 | 40.665 | 3581.184 |
| JFBM48948 | 47 | 118.22 | Fish | 5.56 |  | 689 |
| JFBM48948 | 48 | 96.42 | Fish *Hypophthalmichthys* | 7.954 |  | 1526.9 |
| JFBM48948 | 48 | 96.42 | Fish *Hypophthalmichthys* | 8.989 |  | 1889.15 |
| JFBM48948 | 48 | 96.42 | Fish *Hypophthalmichthys* | 9.551 |  | 2085.85 |
| JFBM48948 | 49 | 112.68 | Fish *Hypophthalmichthys* | 8.839 |  | 1836.65 |
| JFBM48948 | 49 | 112.68 | Fish unidentifiable | 7.91 |  | 1511.5 |
| JFBM48948 | 49 | 112.68 | Fish unidentifiable | 5.65 |  | 720.5 |
| JFBM48948 | 49 | 112.68 | Fish unidentifiable | 6.072 |  | 868.2 |
| JFBM48948 | 49 | 112.68 | Fish unidentifiable | 9.654 |  | 2121.9 |
| JFBM48948 | 52 | 102.91 | Fish *Hypophthalmichthys* | 6.408 |  | 985.8 |
| JFBM48948 | 52 | 102.91 | Fish *Hypophthalmichthys* | 7.239 |  | 1276.65 |
| JFBM48948 | 52 | 102.91 | Fish *Hypophthalmichthys* | 8.604 |  | 1754.4 |
| JFBM48948 | 52 | 102.91 | Fish unidentifiable | 7.957 |  | 1527.95 |
| JFBM48948 | 53 | 99.46 | Insecta larvae aquatic | 0.425 | 4.671 | 0.6975 |
| JFBM48948 | 53 | 99.46 | Insecta larvae aquatic | 0.561 |  | 1.88557 |
| JFBM48948 | 54 | 104.51 | Fish *Hypophthalmichthys* | 8.073 |  | 1568.55 |
| JFBM48948 | 54 | 104.51 | Fish *Hypophthalmichthys* | 7.296 |  | 1296.6 |
| JFBM48948 | 57 | 103.52 | Fish *Hypophthalmichthys* | 8.008 |  | 2742.336 |
| JFBM48948 | 57 | 103.52 | Fish unidentifiable | 9.505 |  | 5050.71 |
| JFBM48948 | 57 | 103.52 | Fish *Hypophthalmichthys* | 8.394 |  | 3337.548 |
| JFBM48948 | 57 | 103.52 | Fish not *Hypophthalmichthys* | 10.488 |  | 6566.496 |
| JFBM48948 | 57 | 103.52 | Fish unidentifiable | 11.834 |  | 8642.028 |
| JFBM48948 | 58 | 106.74 | Fish *Hypophthalmichthys* | 8.179 |  | 1605.65 |
| JFBM48948 | 58 | 106.74 | Fish *Hypophthalmichthys* | 9.589 |  | 2099.15 |
| JFBM48948 | 58 | 106.74 | Fish unidentifiable | 6.6 |  | 1053 |
| JFBM48948 | 58 | 106.74 | Fish unidentifiable | 8.155 |  | 1597.25 |
| JFBM48948 | 58 | 106.74 | Fish unidentifiable | 7.914 |  | 1512.9 |
| JFBM48948 | 58 | 106.74 | Fish unidentifiable | 10.952 |  | 2576.2 |
| JFBM48948 | 58 | 106.74 | Fish Lepomis | 7.579 | 22.392 | 2080.818 |
| JFBM48948 | 60 | 96.94 | Fish unidentifiable | 7.63 |  | 2159.46 |
| JFBM48948 | 61 | 123.41 | Fish *Hypophthalmichthys* | 9.13 |  | 4472.46 |
| JFBM48948 | 61 | 123.41 | Odonata terrestrial | 1.278 |  | 2.46788 |
| JFBM48948 | 62 | 94.93 | Fish *Hypophthalmichthys* | 10.524 |  | 6622.008 |
| JFBM48948 | 62 | 94.93 | Fish *Hypophthalmichthys* | 7.305 |  | 1658.31 |
| JFBM48948 | 62 | 94.93 | Trichoptera pupa aquatic | 1.841 | 8.098 | 25.02605 |
| JFBM48948 | 63 | 111.63 | Fish *Hypophthalmichthys* | 8.579 |  | 3622.818 |
| JFBM48948 | 63 | 111.63 | Fish *Hypophthalmichthys* | 9.112 |  | 4444.704 |
| JFBM48948 | 63 | 111.63 | Plant | 1.797 | 5.879 | 16.51538 |
| JFBM48948 | 63 | 111.63 | Fish *Hypophthalmichthys* | 10.668 |  | 2476.8 |
| JFBM48948 | 63 | 111.63 | Fish *Hypophthalmichthys* | 8.742 |  | 1802.7 |
| JFBM48948 | 64 | 109.85 | Fish *Hypophthalmichthys* | 8.032 | 32.333 | 1727.81412 |
| JFBM48948 | 64 | 109.85 | Fish | 7.333 |  | 1701.486 |
| JFBM48948 | 64 | 109.85 | Fish | 7.481 |  | 1929.702 |
| JFBM48948 | 65 | 117.39 | Fish *Hypophthalmichthys* | 10.656 |  | 6825.552 |
| JFBM48948 | 65 | 117.39 | Fish *Hypophthalmichthys* | 12.104 |  | 9058.368 |
| JFBM48948 | 65 | 117.39 | Fish *Hypophthalmichthys* | 10.788 |  | 7029.096 |
| JFBM48948 | 65 | 117.39 | Nematoda | 0.076 |  | 6.73884 |
| JFBM48948 | 65 | 117.39 | Fish *Hypophthalmichthys* | 8.632 |  | 3704.544 |
| JFBM48948 | 65 | 117.39 | Fish *Hypophthalmichthys* | 13.636 |  | 11420.712 |
| JFBM48948 | 65 | 117.39 | Fish *Hypophthalmichthys* | 9.295 |  | 4726.89 |
| JFBM48948 | 65 | 117.39 | Fish *Hypophthalmichthys* | 11.526 |  | 8167.092 |
| JFBM48948 | 66 | 109.21 | Fish not *Hypophthalmichthys* | 10.583 |  | 6712.986 |
| JFBM48948 | 66 | 109.21 | Fish *Hypophthalmichthys* | 9.635 |  | 5251.17 |
| JFBM48948 | 66 | 109.21 | Fish *Hypophthalmichthys* | 7.237 |  | 1553.454 |
| JFBM48948 | 66 | 109.21 | Fish *Hypophthalmichthys* | 6.797 |  | 874.974 |
| JFBM48948 | 66 | 109.21 | Fish *Hypophthalmichthys* | 8.431 |  | 3394.602 |
| JFBM48948 | 66 | 109.21 | Fish *Hypophthalmichthys* | 7.688 |  | 2248.896 |
| JFBM48948 | 67 | 110.67 | Fish *Hypophthalmichthys* | 9.125 |  | 4464.75 |
| JFBM48948 | 67 | 110.67 | Fish | 8.482 |  | 3473.244 |
| JFBM48948 | 68 | 110.32 | Fish not *Hypophthalmichthys* | 7.537 |  | 2016.054 |
| JFBM48948 | 68 | 110.32 | Fish *Hypophthalmichthys* | 7.78 |  | 2390.76 |
| JFBM48948 | 68 | 110.32 | Odonata terrestrial | 0.662 |  | 0.98397 |
| JFBM48948 | 68 | 110.32 | Fish *Hypophthalmichthys* | 8.118 |  | 2911.956 |
| JFBM48948 | 68 | 110.32 | Fish unidentifiable | 6.091 |  | 874.85 |
| JFBM48948 | 68 | 110.32 | Fish unidentifiable | 6.166 |  | 901.1 |
| JFBM48967 | 4 | 62.14 | Fish *Hypophthalmichthys* | 7.362 | 29.226 | 1252.70228 |
| JFBM48967 | 5 | 89.49 | Fish *Hypophthalmichthys* | 6.985 | 29.619 | 838.86359 |
| JFBM48967 | 6 | 69.13 | Insecta terrestrial | 2.298 | 7.459 | 36.61423 |
| JFBM48967 | 7 | 71.9 | Coleoptera aquatic | 0.756 |  | 3.51772 |
| JFBM48967 | 7 | 71.9 | Fish *Hypopthalmichthys* | 3.2 |  | 162.672 |
| JFBM48967 | 8 | 63.25 | Fish *Hypopthalmichthys* | 5.643 |  | 718.05 |
| JFBM48967 | 8 | 63.25 | Fish *Hypopthalmichthys* | 5.706 |  | 740.1 |
| JFBM48967 | 9 | 53.06 | Insecta terrestrial | 0.529 |  | 0.66381 |
| JFBM48967 | 9 | 53.06 | Insecta terrestrial | 0.833 |  | 1.3956 |
| JFBM48967 | 9 | 53.06 | Insecta terrestrial | 0.907 |  | 1.57373 |
| JFBM48967 | 9 | 53.06 | Insecta terrestrial | 1.143 |  | 2.14183 |
| JFBM48967 | 9 | 53.06 | Insecta terrestrial | 0.713 |  | 1.10673 |
| JFBM48967 | 9 | 53.06 | Insecta terrestrial | 0.598 |  | 0.82991 |
| JFBM48967 | 9 | 53.06 | Insecta terrestrial | 0.59 |  | 0.81065 |
| JFBM48967 | 9 | 53.06 | Insecta terrestrial | 0.449 |  | 0.47123 |
| JFBM48967 | 9 | 53.06 | Insecta terrestrial | 0.727 |  | 1.14043 |
| JFBM48967 | 9 | 53.06 | Insecta terrestrial | 0.758 |  | 1.21506 |
| JFBM48967 | 9 | 53.06 | Insecta terrestrial | 1.314 |  | 2.55346 |
| JFBM48967 | 9 | 53.06 | Insecta terrestrial | 0.473 |  | 0.52901 |
| JFBM48967 | 9 | 53.06 | Insecta terrestrial | 0.458 |  | 0.4929 |
| JFBM48967 | 9 | 53.06 | Insecta terrestrial | 0.986 |  | 1.7639 |
| JFBM48967 | 9 | 53.06 | Insecta terrestrial | 0.572 |  | 0.76732 |
| JFBM48967 | 9 | 53.06 | Insecta terrestrial | 0.479 |  | 0.54345 |
| JFBM48967 | 9 | 53.06 | Insecta terrestrial | 0.295 |  | 0.10052 |
| JFBM48967 | 9 | 53.06 | Insecta terrestrial | 0.455 |  | 0.48568 |
| JFBM48967 | 9 | 53.06 | Insecta terrestrial | 0.303 |  | 0.11978 |
| JFBM48967 | 9 | 53.06 | Insecta terrestrial | 0.445 |  | 0.4616 |
| JFBM48967 | 9 | 53.06 | Insecta terrestrial | 0.466 |  | 0.51216 |
| JFBM48967 | 9 | 53.06 | Insecta terrestrial | 0.558 |  | 0.73362 |
| JFBM48967 | 9 | 53.06 | Insecta terrestrial | 0.355 |  | 0.24496 |
| JFBM48967 | 9 | 53.06 | Insecta terrestrial | 1.475 |  | 2.94102 |
| JFBM48967 | 9 | 53.06 | Insecta terrestrial | 0.823 |  | 1.37153 |
| JFBM48967 | 9 | 53.06 | Insecta terrestrial | 0.429 |  | 0.42309 |
| JFBM48967 | 9 | 53.06 | Insecta terrestrial | 0.292 |  | 0.0933 |
| JFBM48967 | 9 | 53.06 | Insecta terrestrial | 0.394 |  | 0.33884 |
| JFBM48967 | 9 | 53.06 | Insecta terrestrial | 0.479 |  | 0.54345 |
| JFBM48967 | 9 | 53.06 | Insecta terrestrial | 0.457 |  | 0.49049 |
| JFBM48967 | 9 | 53.06 | Insecta terrestrial | 0.979 |  | 1.74705 |
| JFBM48967 | 9 | 53.06 | Insecta terrestrial | 0.842 |  | 1.41726 |
| JFBM48967 | 9 | 53.06 | Insecta terrestrial | 0.468 |  | 0.51697 |
| JFBM48967 | 9 | 53.06 | Insecta terrestrial | 0.711 |  | 1.10192 |
| JFBM48967 | 9 | 53.06 | Insecta terrestrial | 0.94 |  | 1.65317 |
| JFBM48967 | 9 | 53.06 | Insecta terrestrial | 0.47 |  | 0.52178 |
| JFBM48967 | 9 | 53.06 | Insecta terrestrial | 0.675 |  | 1.01526 |
| JFBM48967 | 9 | 53.06 | Insecta terrestrial | 0.779 |  | 1.26561 |
| JFBM48967 | 9 | 53.06 | Insecta terrestrial | 0.722 |  | 1.1284 |
| JFBM48967 | 9 | 53.06 | Insecta terrestrial | 0.475 |  | 0.53382 |
| JFBM48967 | 9 | 53.06 | Insecta terrestrial | 0.449 |  | 0.47123 |
| JFBM48967 | 9 | 53.06 | Insecta terrestrial | 0.632 |  | 0.91175 |
| JFBM48967 | 9 | 53.06 | Insecta terrestrial | 0.572 |  | 0.76732 |
| JFBM48967 | 9 | 53.06 | Insecta terrestrial | 0.545 |  | 0.70232 |
| JFBM48967 | 9 | 53.06 | Insecta terrestrial | 0.523 |  | 0.64937 |
| JFBM48967 | 10 | 73.98 | Fish *Hypopthalmichthys* | 5.28 |  | 591 |
| JFBM48967 | 10 | 73.98 | Fish *Hypopthalmichthys* | 4.452 |  | 301.2 |
| JFBM48967 | 12 | 64.06 | Fish *Hypopthalmichthys* | 5.358 |  | 618.3 |
| JFBM48967 | 13 | 58.16 | Fish *Hypopthalmichthys* | 4.593 |  | 350.55 |
| JFBM48967 | 13 | 58.16 | Fish *Hypopthalmichthys* | 5.293 |  | 595.55 |
| JFBM48967 | 13 | 58.16 | Fish *Hypopthalmichthys* | 3.651 |  | 20.85 |
| JFBM48967 | 15 | 80.37 | Insecta terrestrial | 1.551 |  | 3.12397 |
| JFBM48967 | 15 | 80.37 | Insecta terrestrial | 2.709 | 11.018 | 67.99188 |
| JFBM48967 | 15 | 80.37 | Insecta larvae aquatic | 1.331 | 6.791 | 3.12826 |
| JFBM48967 | 15 | 80.37 | Fish *Hypopthalmichthys* | 5.349 |  | 615.15 |
| JFBM48967 | 15 | 80.37 | Fish *Hypopthalmichthys* | 6.476 |  | 379.992 |
| JFBM48967 | 16 | 80.29 | Fish *Hypopthalmichthys* | 6.833 |  | 930.486 |
| JFBM48967 | 16 | 80.29 | Fish *Hypopthalmichthys* | 5.11 |  | 531.5 |
| JFBM48967 | 16 | 80.29 | Fish *Hypopthalmichthys* | 4.032 |  | 154.2 |
| JFBM48967 | 16 | 80.29 | Insecta terrestrial | 1.504 |  | 3.01083 |
| JFBM48967 | 17 | 36.45 | Insecta terrestrial | 0.196 |  | 0.00214 |
| JFBM48967 | 17 | 36.45 | Insecta terrestrial | 0.302 |  | 0.11737 |
| JFBM48967 | 17 | 36.45 | Insecta terrestrial | 0.326 | 3.704 | 0.41174 |
| JFBM48967 | 17 | 36.45 | Insecta terrestrial | 0.367 | 3.337 | 0.37161 |
| JFBM48967 | 17 | 36.45 | Insecta terrestrial | 0.491 |  | 0.57234 |
| JFBM48967 | 17 | 36.45 | Insecta terrestrial | 1.044 |  | 1.90352 |
| JFBM48967 | 17 | 36.45 | Insecta terrestrial | 1.161 |  | 2.18516 |
| JFBM48967 | 17 | 36.45 | Insecta terrestrial | 1.229 |  | 2.34885 |
| JFBM48967 | 17 | 36.45 | Plant |  |  | 27.685 |
| JFBM48967 | 19 | 81.19 | Fish *Hypopthalmichthys* | 7.085 |  | 1319.07 |
| JFBM48967 | 20 | 61.59 | Fish *Hypopthalmichthys* | 5.92 |  | 815 |
| JFBM48967 | 20 | 61.59 | Fish *Hypopthalmichthys* | 4.121 |  | 185.35 |
| JFBM48967 | 21 | 74.11 | Fish *Hypopthalmichthys* | 5.025 |  | 501.75 |
| JFBM48967 | 21 | 74.11 | Fish *Hypopthalmichthys* | 6.342 |  | 173.364 |
| JFBM48967 | 21 | 74.11 | Fish *Hypopthalmichthys* | 6.842 |  | 944.364 |
| JFBM48967 | 22 | 72.14 | Insecta terrestrial | 1.5 |  | 3 |
| JFBM48967 | 22 | 72.14 | Insecta terrestrial | 1.277 |  | 2.46439 |
| JFBM48967 | 23 | 58.85 | Insecta terrestrial | 0.989 |  | 1.77112 |
| JFBM48967 | 23 | 58.85 | Insecta terrestrial | 1.093 |  | 2.02147 |
| JFBM48967 | 23 | 58.85 | Fish *Hypopthalmichthys* | 4.462 |  | 304.7 |
| JFBM48967 | 23 | 58.85 | Fish *Hypopthalmichthys* | 4.265 |  | 235.75 |
| JFBM48967 | 24 | 73.18 | Fish *Hypopthalmichthys* | 3.694 |  | 35.9 |
| JFBM48967 | 24 | 73.18 | Fish *Hypopthalmichthys* | 6.204 |  | 914.4 |
| JFBM48967 | 24 | 73.18 | Fish *Hypopthalmichthys* | 5.613 |  | 707.55 |
| JFBM48967 | 25 | 59.88 | Fish *Hypopthalmichthys* | 4.691 |  | 384.85 |
| JFBM48967 | 25 | 59.88 | Fish *Hypopthalmichthys* | 4.443 |  | 298.05 |
| JFBM48967 | 25 | 59.88 | Fish *Hypopthalmichthys* | 3.612 |  | 7.2 |
| JFBM48967 | 25 | 59.88 | Fish *Hypopthalmichthys* | 3.335 |  | 171.83985 |
| JFBM48967 | 25 | 59.88 | Insecta terrestrial | 1.14 |  | 2.13461 |
| JFBM48967 | 25 | 59.88 | Insecta larvae aquatic | 0.46 |  | 1.0402 |
| JFBM48967 | 25 | 59.88 | Insecta larvae aquatic | 0.351 |  | 0.12787 |
| JFBM48967 | 26 | 44.77 | Insecta larvae aquatic | 0.358 | 3.058 | 0.30876 |
| JFBM48967 | 26 | 44.77 | Insecta larvae aquatic | 0.402 | 4.238 | 0.87543 |
| JFBM48967 | 26 | 44.77 | Insecta larvae aquatic | 0.436 |  | 0.83932 |
| JFBM48967 | 26 | 44.77 | Insecta larvae aquatic | 0.419 |  | 0.69703 |
| JFBM48967 | 26 | 44.77 | Insecta larvae aquatic | 0.402 |  | 0.55474 |
| JFBM48967 | 26 | 44.77 | Insecta larvae aquatic | 0.26 |  | 0.24293 |
| JFBM48967 | 26 | 44.77 | Insecta larvae aquatic | 0.37 |  | 0.2869 |
| JFBM48967 | 26 | 44.77 | Insecta terrestrial | 0.539 |  | 0.68788 |
| JFBM48967 | 26 | 44.77 | Insecta terrestrial | 0.414 |  | 0.38698 |
| JFBM48967 | 26 | 44.77 | Insecta terrestrial | 0.378 |  | 0.30032 |
| JFBM48967 | 26 | 44.77 | Insecta terrestrial | 0.636 |  | 0.92138 |
| JFBM48967 | 26 | 44.77 | Insecta terrestrial | 0.741 |  | 1.17414 |
| JFBM48967 | 26 | 44.77 | Insecta terrestrial | 0.769 |  | 1.24154 |
| JFBM48967 | 26 | 44.77 | Insecta terrestrial | 0.497 |  | 0.58678 |
| JFBM48967 | 26 | 44.77 | Insecta terrestrial | 0.395 |  | 0.34124 |
| JFBM48967 | 26 | 44.77 | Insecta terrestrial | 0.834 |  | 1.398 |
| JFBM48967 | 26 | 44.77 | Insecta terrestrial | 0.656 |  | 0.96952 |
| JFBM48967 | 26 | 44.77 | Insecta terrestrial | 0.778 |  | 1.2632 |
| JFBM48967 | 26 | 44.77 | Insecta terrestrial | 0.833 |  | 1.3956 |
| JFBM48967 | 26 | 44.77 | Insecta terrestrial | 0.611 |  | 0.8612 |
| JFBM48967 | 26 | 44.77 | Insecta terrestrial | 0.428 |  | 0.42068 |
| JFBM48967 | 26 | 44.77 | Insecta terrestrial | 0.641 |  | 0.93342 |
| JFBM48967 | 26 | 44.77 | Insecta terrestrial | 0.91 |  | 1.58095 |
| JFBM48967 | 26 | 44.77 | Insecta terrestrial | 0.313 |  | 0.14385 |
| JFBM48967 | 26 | 44.77 | Insecta terrestrial | 0.47 |  | 0.52178 |
| JFBM48967 | 26 | 44.77 | Insecta terrestrial | 0.816 |  | 1.35468 |
| JFBM48967 | 26 | 44.77 | Insecta terrestrial | 1.467 |  | 2.92176 |
| JFBM48967 | 26 | 44.77 | Insecta terrestrial | 0.9 |  | 1.55688 |
| JFBM48967 | 26 | 44.77 | Insecta terrestrial | 0.749 |  | 1.19339 |
| JFBM48967 | 26 | 44.77 | Insecta terrestrial | 0.686 |  | 1.04174 |
| JFBM48967 | 26 | 44.77 | Insecta terrestrial | 0.702 |  | 1.08025 |
| JFBM48967 | 26 | 44.77 | Insecta terrestrial | 1.234 |  | 2.36088 |
| JFBM48967 | 26 | 44.77 | Insecta terrestrial | 0.646 |  | 0.94545 |
| JFBM48967 | 26 | 44.77 | Insecta terrestrial | 0.478 |  | 0.54104 |
| JFBM48967 | 26 | 44.77 | Insecta terrestrial | 0.721 |  | 1.12599 |
| JFBM48967 | 27 | 72.6 | Fish *Hypopthalmichthys* | 6.7 |  | 725.4 |
| JFBM48967 | 27 | 72.6 | Fish *Hypopthalmichthys* | 5.738 |  | 751.3 |
| JFBM48967 | 28 | 63.75 | Fish *Hypopthalmichthys* | 4.352 |  | 266.2 |
| JFBM48967 | 28 | 63.75 | Fish *Hypopthalmichthys* | 4.562 |  | 339.7 |
| JFBM48967 | 28 | 63.75 | Fish *Hypopthalmichthys* | 4 |  | 143 |
| JFBM48967 | 29 | 68.08 | Insecta terrestrial | 1.202 |  | 2.28385 |
| JFBM48967 | 29 | 68.08 | Insecta terrestrial | 1.519 |  | 3.04694 |
| JFBM48967 | 29 | 68.08 | Insecta terrestrial | 1.652 |  | 3.36709 |
| JFBM48967 | 29 | 68.08 | Insecta terrestrial | 1.695 |  | 3.4706 |
| JFBM48967 | 30 | 63.39 | Fish *Hypopthalmichthys* | 4.336 |  | 260.6 |
| JFBM48967 | 30 | 63.39 | Fish *Hypopthalmichthys* | 4.635 |  | 365.25 |
| JFBM48967 | 30 | 63.39 | Fish *Hypopthalmichthys* | 5.449 |  | 650.15 |
| JFBM48967 | 31 | 67.37 | Fish *Hypopthalmichthys* | 4.071 |  | 167.85 |
| JFBM48967 | 31 | 67.37 | Fish *Hypopthalmichthys* | 3.394 |  | 7.56044 |
| JFBM48967 | 31 | 67.37 | Fish *Hypopthalmichthys* | 4.958 |  | 478.3 |
| JFBM48967 | 31 | 67.37 | Fish *Hypopthalmichthys* | 4.242 |  | 227.7 |
| JFBM48967 | 31 | 67.37 | Fish *Hypopthalmichthys* | 5.447 | 20.011 | 458.77241 |
| JFBM48967 | 33 | 56.26 | Fish *Hypopthalmichthys* | 5.273 |  | 588.55 |
| JFBM48967 | 33 | 56.26 | Fish *Hypopthalmichthys* | 5.192 |  | 560.2 |
| JFBM48967 | 33 | 56.26 | Fish *Hypopthalmichthys* | 5.804 |  | 774.4 |
| JFBM48967 | 33 | 56.26 | Insecta larvae aquatic | 0.366 | 4.577 | 0.5246 |
| JFBM48967 | 33 | 56.26 | Insecta larvae aquatic | 0.36 | 4.592 | 0.526 |
| JFBM48967 | 33 | 56.26 | Insecta larvae aquatic | 0.425 | 3.956 | 0.99485 |
| JFBM48967 | 33 | 56.26 | Insecta terrestrial | 0.745 |  | 1.18376 |
| JFBM48967 | 33 | 56.26 | Insecta terrestrial | 0.896 |  | 1.54725 |
| JFBM48967 | 33 | 56.26 | Insecta terrestrial | 0.636 |  | 0.92138 |
| JFBM48967 | 33 | 56.26 | Insecta terrestrial | 0.813 |  | 1.34745 |
| JFBM48967 | 33 | 56.26 | Insecta terrestrial | 0.403 |  | 0.3605 |
| JFBM48967 | 33 | 56.26 | Insecta terrestrial | 0.52 |  | 0.64214 |
| JFBM48967 | 33 | 56.26 | Insecta terrestrial | 0.931 |  | 1.6315 |
| JFBM48967 | 33 | 56.26 | Insecta terrestrial | 0.511 | 1.851 | 0.19549 |
| JFBM48967 | 33 | 56.26 | Insecta terrestrial | 0.923 |  | 1.61225 |
| JFBM48967 | 33 | 56.26 | Insecta terrestrial | 1.041 |  | 1.8963 |
| JFBM48967 | 33 | 56.26 | Insecta terrestrial | 1.237 |  | 2.36811 |
| JFBM48967 | 33 | 56.26 | Insecta terrestrial | 0.648 |  | 0.95027 |
| JFBM48967 | 33 | 56.26 | Insecta terrestrial | 0.955 |  | 1.68928 |
| JFBM48967 | 33 | 56.26 | Insecta terrestrial | 1.42 |  | 2.80862 |
| JFBM48967 | 33 | 56.26 | Insecta terrestrial | 0.792 |  | 1.2969 |
| JFBM48967 | 33 | 56.26 | Insecta terrestrial | 0.422 |  | 0.40624 |
| JFBM48967 | 33 | 56.26 | Insecta terrestrial | 0.44 |  | 0.44957 |
| JFBM48967 | 33 | 56.26 | Insecta terrestrial | 0.493 |  | 0.57715 |
| JFBM48967 | 33 | 56.26 | Insecta terrestrial | 0.872 |  | 1.48948 |
| JFBM48967 | 33 | 56.26 | Insecta terrestrial | 0.748 |  | 1.19099 |
| JFBM48967 | 33 | 56.26 | Insecta terrestrial | 0.986 |  | 1.7639 |
| JFBM48967 | 33 | 56.26 | Insecta terrestrial | 0.602 |  | 0.83953 |
| JFBM48967 | 33 | 56.26 | Insecta terrestrial | 0.472 |  | 0.5266 |
| JFBM48967 | 33 | 56.26 | Insecta terrestrial | 0.586 |  | 0.80102 |
| JFBM48967 | 33 | 56.26 | Insecta terrestrial | 0.747 |  | 1.18858 |
| JFBM48967 | 33 | 56.26 | Insecta terrestrial | 0.496 |  | 0.58437 |
| JFBM48967 | 33 | 56.26 | Insecta terrestrial | 0.23 |  | 0.23339 |
| JFBM48967 | 33 | 56.26 | Insecta terrestrial | 0.436 |  | 0.43994 |
| JFBM48967 | 33 | 56.26 | Insecta terrestrial | 1.335 |  | 2.60401 |
| JFBM48967 | 33 | 56.26 | Insecta terrestrial | 0.638 |  | 0.92619 |
| JFBM48967 | 33 | 56.26 | Insecta terrestrial | 0.383 |  | 0.31236 |
| JFBM48967 | 33 | 56.26 | Insecta terrestrial | 0.641 |  | 0.93342 |
| JFBM48967 | 33 | 56.26 | Insecta terrestrial | 0.532 |  | 0.67103 |
| JFBM48967 | 33 | 56.26 | Insecta terrestrial | 0.704 |  | 1.08507 |
| JFBM48967 | 33 | 56.26 | Insecta terrestrial | 0.746 |  | 1.18617 |
| JFBM48967 | 33 | 56.26 | Insecta terrestrial | 0.724 |  | 1.13321 |
| JFBM48967 | 33 | 56.26 | Insecta terrestrial | 0.728 |  | 1.14284 |
| JFBM48967 | 33 | 56.26 | Insecta terrestrial | 0.288 |  | 0.08367 |
| JFBM48967 | 33 | 56.26 | Insecta terrestrial | 0.509 |  | 0.61566 |
| JFBM48967 | 33 | 56.26 | Insecta terrestrial | 0.602 |  | 0.83953 |
| JFBM48967 | 33 | 56.26 | Insecta terrestrial | 0.718 |  | 1.11877 |
| JFBM48967 | 33 | 56.26 | Insecta terrestrial | 0.719 |  | 1.12118 |
| JFBM48967 | 33 | 56.26 | Insecta terrestrial | 0.757 |  | 1.21265 |
| JFBM48967 | 33 | 56.26 | Insecta terrestrial | 1.135 |  | 2.12257 |
| JFBM48967 | 33 | 56.26 | Insecta terrestrial | 0.468 |  | 0.51697 |
| JFBM48967 | 33 | 56.26 | Insecta terrestrial | 0.264 |  | 0.0259 |
| JFBM48967 | 33 | 56.26 | Insecta terrestrial | 0.468 |  | 0.51697 |
| JFBM48967 | 33 | 56.26 | Insecta terrestrial | 1.158 |  | 2.17794 |
| JFBM48967 | 33 | 56.26 | Insecta terrestrial | 1.437 |  | 2.84955 |
| JFBM48967 | 33 | 56.26 | Insecta terrestrial | 0.577 |  | 0.77935 |
| JFBM48967 | 33 | 56.26 | Insecta terrestrial | 0.73 |  | 1.14766 |
| JFBM48967 | 33 | 56.26 | Insecta terrestrial | 0.433 |  | 0.43272 |
| JFBM48967 | 34 | 62.59 | Insecta terrestrial | 0.896 |  | 1.54725 |
| JFBM48967 | 34 | 62.59 | Insecta terrestrial | 0.979 |  | 1.74705 |
| JFBM48967 | 34 | 62.59 | Insecta terrestrial | 1.143 |  | 2.14183 |
| JFBM48967 | 34 | 62.59 | Insecta terrestrial | 1.389 |  | 2.734 |
| JFBM48967 | 34 | 62.59 | Insecta terrestrial | 0.624 |  | 0.89249 |
| JFBM48967 | 34 | 62.59 | Fish (*Cypriniformes* sp.) | 2.623 |  | 123.48793 |
| JFBM48967 | 35 | 50.82 | Fish *Hypopthalmichthys* | 5.183 |  | 557.05 |
| JFBM48967 | 35 | 50.82 | Fish *Hypopthalmichthys* | 4.461 |  | 304.35 |
| JFBM48967 | 36 | 59.59 | Fish *Hypopthalmichthys* | 4.543 |  | 333.05 |
| JFBM48967 | 36 | 59.59 | Fish *Hypopthalmichthys* | 3.61 |  | 6.5 |
| JFBM48967 | 37 | 57.08 | Fish *Hypopthalmichthys* | 3.326 |  | 171.22866 |
| JFBM48967 | 37 | 57.08 | Insecta larvae aquatic | 2.311 |  | 16.53307 |
| JFBM48967 | 37 | 57.08 | Insecta larvae aquatic | 0.648 | 3.672 | 2.3873 |
| JFBM48967 | 37 | 57.08 | Insecta larvae aquatic | 0.719 |  | 3.20803 |
| JFBM48967 | 37 | 57.08 | Insecta larvae aquatic | 0.569 |  | 1.95253 |
| JFBM48967 | 37 | 57.08 | Insecta larvae aquatic | 0.698 |  | 3.03226 |
| JFBM48967 | 37 | 57.08 | Insecta larvae aquatic | 0.681 | 1.065 | 0.87217 |
| JFBM48967 | 38 | 50.84 | Fish | 3.682 |  | 31.7 |
| JFBM48967 | 38 | 50.84 | Insecta terrestrial | 0.734 |  | 1.15728 |
| JFBM48967 | 38 | 50.84 | Insecta terrestrial | 1.03 |  | 1.86982 |
| JFBM48967 | 39 | 50.41 | Insecta larvae aquatic | 0.261 | 2.973 | 0.23765 |
| JFBM48967 | 39 | 50.41 | Insecta larvae aquatic | 1.416 | 7.078 | 7.54665 |
| JFBM48967 | 39 | 50.41 | Insecta larvae aquatic | 0.511 | 5.698 | 1.15727 |
| JFBM48967 | 39 | 50.41 | Insecta larvae aquatic | 0.531 | 4.611 | 1.2221 |
| JFBM48967 | 39 | 50.41 | Insecta terrestrial | 0.681 |  | 1.0297 |
| JFBM48967 | 39 | 50.41 | Insecta terrestrial | 0.72 |  | 1.12358 |
| JFBM48967 | 39 | 50.41 | Insecta terrestrial | 0.785 |  | 1.28005 |
| JFBM48967 | 39 | 50.41 | Insecta terrestrial | 1.317 |  | 2.56068 |
| JFBM48967 | 39 | 50.41 | Insecta terrestrial | 0.865 |  | 1.47263 |
| JFBM48967 | 39 | 50.41 | Insecta terrestrial | 0.566 |  | 0.75288 |
| JFBM48967 | 39 | 50.41 | Insecta terrestrial | 0.446 |  | 0.46401 |
| JFBM48967 | 39 | 50.41 | Insecta terrestrial | 0.974 | 2.651 | 1.78993 |
| JFBM48967 | 39 | 50.41 | Insecta terrestrial | 0.98 |  | 1.74946 |
| JFBM48967 | 39 | 50.41 | Insecta terrestrial | 0.78 |  | 1.26802 |
| JFBM48967 | 39 | 50.41 | Insecta terrestrial | 0.864 |  | 1.47022 |
| JFBM48967 | 39 | 50.41 | Insecta terrestrial | 1.485 |  | 2.96509 |
| JFBM48967 | 39 | 50.41 | Insecta terrestrial | 0.846 |  | 1.42689 |
| JFBM48967 | 39 | 50.41 | Insecta terrestrial | 0.689 |  | 1.04896 |
| JFBM48967 | 39 | 50.41 | Insecta terrestrial | 1.128 |  | 2.10572 |
| JFBM48967 | 39 | 50.41 | Insecta terrestrial | 1.093 |  | 2.02147 |
| JFBM48967 | 39 | 50.41 | Insecta terrestrial | 0.661 |  | 0.98156 |
| JFBM48967 | 39 | 50.41 | Insecta terrestrial | 0.815 |  | 1.35227 |
| JFBM48967 | 39 | 50.41 | Insecta terrestrial | 0.627 |  | 0.89971 |
| JFBM48967 | 39 | 50.41 | Insecta terrestrial | 0.806 |  | 1.3306 |
| JFBM48967 | 39 | 50.41 | Insecta terrestrial | 0.775 |  | 1.25598 |
| JFBM48967 | 39 | 50.41 | Insecta terrestrial | 1.294 |  | 2.50532 |
| JFBM48967 | 39 | 50.41 | Insecta terrestrial | 0.637 | 1.752 | 0.72046 |
| JFBM48967 | 39 | 50.41 | Insecta terrestrial | 0.723 |  | 1.13081 |
| JFBM48967 | 39 | 50.41 | Insecta terrestrial | 0.724 |  | 1.13321 |
| JFBM48967 | 39 | 50.41 | Insecta terrestrial | 0.474 |  | 0.53141 |
| JFBM48967 | 39 | 50.41 | Insecta terrestrial | 0.66 |  | 0.97915 |
| JFBM48967 | 39 | 50.41 | Insecta terrestrial | 0.506 |  | 0.60844 |
| JFBM48967 | 39 | 50.41 | Insecta terrestrial | 1.025 |  | 1.85778 |
| JFBM48967 | 39 | 50.41 | Insecta terrestrial | 0.449 |  | 0.47123 |
| JFBM48967 | 39 | 50.41 | Insecta terrestrial | 0.582 |  | 0.79139 |
| JFBM48967 | 39 | 50.41 | Insecta terrestrial | 0.651 |  | 0.95749 |
| JFBM48967 | 39 | 50.41 | Insecta terrestrial | 0.836 |  | 1.40282 |
| JFBM48967 | 39 | 50.41 | Insecta terrestrial | 0.764 |  | 1.2295 |
| JFBM48967 | 39 | 50.41 | Insecta terrestrial | 0.72 |  | 1.12358 |
| JFBM48967 | 39 | 50.41 | Insecta terrestrial | 0.702 | 1.988 | 0.55668 |
| JFBM48967 | 40 | 35.55 | Insecta larvae aquatic | 0.299 |  | 0.25532 |
| JFBM48967 | 40 | 35.55 | Insecta larvae aquatic | 0.301 | 2.437 | 0.14934 |
| JFBM48967 | 40 | 35.55 | Insecta terrestrial | 0.312 |  | 0.14145 |
| JFBM48967 | 40 | 35.55 | Insecta larvae aquatic | 0.352 |  | 0.13624 |
| JFBM48967 | 40 | 35.55 | Daphnia | 0.533 | 0.848 | 0.20451 |
| JFBM48967 | 40 | 35.55 | Daphnia | 0.548 | 0.867 | 0.34858 |
| JFBM48967 | 40 | 35.55 | Insecta larvae aquatic | 0.555 |  | 1.83535 |
| JFBM48967 | 40 | 35.55 | Daphnia | 0.578 | 1.129 | 0.73012 |
| JFBM48967 | 40 | 35.55 | Daphnia | 0.591 | 0.983 | 0.81756 |
| JFBM48967 | 40 | 35.55 | Daphnia | 0.627 | 1.016 | 0.48881 |
| JFBM48967 | 40 | 35.55 | Insecta larvae aquatic | 0.637 |  | 2.52169 |
| JFBM48967 | 40 | 35.55 | Insecta larvae aquatic | 0.79 |  | 3.8023 |
| JFBM48967 | 40 | 35.55 | Insecta larvae aquatic | 0.808 | 3.051 | 1.23317 |
| JFBM48967 | 40 | 35.55 | Insecta larvae aquatic | 0.834 |  | 4.17058 |
| JFBM48989 | 1 | 140.91 | Seed | 2.503 | 6.81 | 115.33873 |
| JFBM48989 | 1 | 140.91 | Fish | 2.578 |  | 120.43198 |
| JFBM48989 | 2 | 71.01 | Diptera larvae aquatic | 0.383 |  | 0.39405 |
| JFBM48989 | 4 | 61.77 | Cladocera | 0.254 |  | 0.00821 |
| JFBM48989 | 4 | 61.77 | Cladocera | 0.318 |  | 0.01596 |
| JFBM48989 | 4 | 61.77 | Copepoda | 0.282 |  | 0.01094 |
| JFBM48989 | 4 | 61.77 | Cladocera | 0.231 |  | 0.00648 |
| JFBM48989 | 4 | 61.77 | Copepoda | 0.163 |  | 0.00321 |
| JFBM48989 | 4 | 61.77 | Copepoda | 0.356 |  | 0.02354 |
| JFBM48989 | 4 | 61.77 | Copepoda | 0.447 | 0.686 | 0.12182 |
| JFBM48989 | 4 | 61.77 | Copepoda | 0.347 | 1.722 | 0.01253 |
| JFBM48989 | 4 | 61.77 | Copepoda | 0.302 | 0.542 | 0.03681 |
| JFBM48989 | 6 | 57.42 | Copepoda | 0.37 | 0.763 | 0.12364 |
| JFBM48989 | 6 | 57.42 | Diptera larvae aquatic | 0.204 |  | 0.22524 |
| JFBM48989 | 6 | 57.42 | Copepoda | 0.322 |  | 0.01657 |
| JFBM48989 | 7 | 67.42 | Copepoda | 0.253 |  | 0.00815 |
| JFBM48989 | 7 | 67.42 | Diptera larvae aquatic | 0.491 |  | 0.01534 |
| JFBM48989 | 7 | 67.42 | Diptera larvae aquatic | 0.921 |  | 0.03455 |
| JFBM48989 | 7 | 67.42 | Insecta terrestrial | 0.566 |  | 0.75334 |
| JFBM48989 | 7 | 67.42 | Ostracoda | 0.314 | 0.527 | 0.05943 |
| JFBM48989 | 7 | 67.42 | Trichoptera larvae aquatic | 0.385 |  | 0.41617 |
| JFBM48989 | 8 | 52.73 | Trichoptera larvae aquatic | 0.237 |  | 0.23558 |
| JFBM48989 | 10 | 65.18 | Copepoda | 0.365 | 0.773 | 0.14956 |
| JFBM48989 | 10 | 65.18 | Ostracoda | 0.288 | 0.468 | 0.0561 |
| JFBM48989 | 12 | 62 | Cladocera | 0.491 |  | 0.09467 |
| JFBM48989 | 12 | 62 | Cladocera | 0.389 |  | 0.03314 |
| JFBM48989 | 12 | 62 | Cladocera | 0.412 |  | 0.04195 |
| JFBM48989 | 12 | 62 | Cladocera | 0.387 |  | 0.03231 |
| JFBM48989 | 12 | 62 | Cladocera | 0.249 |  | 0.00783 |
| JFBM48989 | 12 | 62 | Cladocera | 0.401 |  | 0.03763 |
| JFBM48989 | 12 | 62 | Insecta larvae aquatic | 0.256 |  | 0.24172 |
| JFBM48989 | 12 | 62 | Insecta terrestrial | 1.149 |  | 2.15671 |
| JFBM48989 | 12 | 62 | Trichoptera larvae aquatic | 0.729 |  | 3.29173 |
| JFBM48989 | 13 | 63.05 | Cladocera | 0.299 |  | 0.01311 |
| JFBM48989 | 13 | 63.05 | Cladocera | 0.367 |  | 0.02647 |
| JFBM48989 | 13 | 63.05 | Cladocera | 0.272 |  | 0.00991 |
| JFBM48989 | 13 | 63.05 | Cladocera | 0.289 |  | 0.01176 |
| JFBM48989 | 13 | 63.05 | Cladocera | 0.3 |  | 0.01319 |
| JFBM48989 | 13 | 63.05 | Cladocera | 0.31 |  | 0.0147 |
| JFBM48989 | 13 | 63.05 | Cladocera | 0.221 |  | 0.00586 |
| JFBM48989 | 13 | 63.05 | Seed | 0.701 | 1.472 | 0.74513 |
| JFBM48989 | 14 | 60.66 | Cladocera | 0.27 |  | 0.00966 |
| JFBM48989 | 14 | 60.66 | Copepoda | 0.471 |  | 0.07736 |
| JFBM48989 | 14 | 60.66 | Diptera larvae aquatic | 0.777 |  | 3.69349 |
| JFBM48989 | 14 | 60.66 | Diptera larvae aquatic | 0.336 |  | 0.26716 |
| JFBM48989 | 14 | 60.66 | Diptera larvae aquatic | 0.279 |  | 0.249 |
| JFBM48989 | 14 | 60.66 | Diptera larvae aquatic | 0.367 |  | 0.25793 |
| JFBM48989 | 14 | 60.66 | Odonata terrestrial | 1.758 |  | 3.62226 |
| JFBM48989 | 14 | 60.66 | Plecoptera aquatic | 0.825 |  | 4.09525 |
| JFBM48989 | 16 | 70 | Insecta larvae aquatic | 0.606 |  | 2.25856 |
| JFBM48989 | 17 | 86.32 | Trichoptera larvae aquatic | 0.459 |  | 1.02878 |
| JFBM48989 | 20 | 81.42 | Insecta larvae aquatic | 0.549 |  | 1.78513 |
| JFBM48989 | 21 | 73.1 | Insecta larvae aquatic | 0.411 |  | 0.63378 |
| JFBM48989 | 22 | 70.38 | Diptera larvae aquatic | 0.219 | 2.298 | 0.14557 |
| JFBM48989 | 22 | 70.38 | Insecta larvae aquatic | 0.534 | 5.102 | 1.08103 |
| JFBM48989 | 22 | 70.38 | Ostracoda | 0.335 | 0.5 | 0.06413 |
| JFBM48989 | 22 | 70.38 | Trichoptera larvae aquatic | 0.88 | 5.049 | 4.74338 |
| JFBM48989 | 22 | 70.38 | Trichoptera larvae aquatic | 0.726 | 4.963 | 2.70782 |
| JFBM48989 | 22 | 70.38 | Trichoptera larvae aquatic | 0.885 | 5.686 | 3.43722 |
| JFBM48989 | 24 | 76.02 | Trichoptera larvae aquatic | 0.495 |  | 1.33315 |
| JFBM48989 | 24 | 76.02 | Trichoptera larvae aquatic | 0.308 |  | 0.25818 |
| JFBM48989 | 24 | 76.02 | Trichoptera larvae aquatic | 0.594 |  | 2.16178 |
| JFBM48989 | 24 | 76.02 | Trichoptera larvae aquatic | 0.468 |  | 1.10716 |
| JFBM48989 | 24 | 76.02 | Trichoptera larvae aquatic | 0.532 |  | 1.64284 |
| JFBM48989 | 24 | 76.02 | Trichoptera larvae aquatic | 0.47 |  | 1.1239 |
| JFBM48989 | 25 | 69.77 | Diptera larvae aquatic | 0.466 |  | 1.09298 |
| JFBM48989 | 26 | 146.87 | Insecta larvae aquatic | 0.456 |  | 1.0091 |
| JFBM48989 | 30 | 138.46 | Ostracoda | 0.534 | 0.448 | 0.03811 |
| JFBM48989 | 33 | 91.46 | Diptera larvae aquatic | 0.126 | 1.349 | 0.05638 |
| JFBM48989 | 36 | 77.9 | Insecta larvae aquatic | 0.487 |  | 1.26208 |
| JFBM48989 | 43 | 80.49 | Cladocera | 0.407 |  | 0.03989 |
| JFBM48989 | 43 | 80.49 | Copepoda | 0.305 | 0.665 | 0.08641 |
| JFBM48989 | 47 | 65.44 | Copepoda | 0.389 | 0.996 | 0.09717 |
| JFBM48989 | 47 | 65.44 | Trichoptera larvae aquatic | 0.283 | 1.956 | 0.16206 |
| JFBM48989 | 49 | 67.96 | Cladocera | 0.259 | 0.583 | 0.14256 |
| JFBM48989 | 49 | 67.96 | Cladocera | 0.348 | 0.628 | 0.07314 |
| JFBM48989 | 49 | 67.96 | Insecta larvae aquatic | 0.229 |  | 0.23312 |
| JFBM48989 | 49 | 67.96 | Ostracoda | 0.374 | 0.484 | 0.05937 |
| JFBM49003 | 1 | 88.81 | Fish unidentifiable | 7.327 |  | 1307.45 |
| JFBM49003 | 1 | 88.81 | Insecta terrestrial Hymenoptera | 1.2 | 4.873 | 11.85181 |
| JFBM49003 | 1 | 88.81 | Insecta terrestrial Hymenoptera | 1.28 |  | 7.9036 |
| JFBM49003 | 2 | 105.15 | Insecta terrestrial Ephemeroptera | 2.095 |  | 14.72515 |
| JFBM49003 | 2 | 105.15 | Fish unidentifiable | 6.572 |  | 1043.2 |
| JFBM49003 | 2 | 105.15 | Insecta larvae aquatic diptera |  |  | 0.0006 |
| JFBM49004 | 1 | 101.13 | Fish unmeasureable | 4.642 |  | 367.7 |
| JFBM49004 | 2 | 107.79 | Fish unidentifiable | 4.457 |  | 302.95 |
| JFBM49005 | 1 | 143.85 | Fish *Gambusia* | 7.588 | 30.743 | 1542.97411 |
| JFBM49006 | 1 | 133.42 | Fish unidentifiable | 6.354 |  | 966.9 |
| JFBM49006 | 1 | 133.42 | Fish unidentifiable | 4.531 |  | 328.85 |
| JFBM49006 | 1 | 133.42 | Fish unidentifiable | 6.516 |  | 1023.6 |
| JFBM49006 | 1 | 133.42 | Fish unidentifiable | 4.413 |  | 287.55 |
| JFBM49006 | 1 | 133.42 | Fish unidentifiable | 5.906 |  | 810.1 |
| JFBM49007 | 1 | 137.87 | Insecta terrestrial | 1.16 |  | 6.8992 |
| JFBM49007 | 1 | 137.87 | Insecta terrestrial | 1.484 |  | 9.61108 |
| JFBM49007 | 1 | 137.87 | Insecta terrestrial | 1.263 |  | 7.76131 |
| JFBM49007 | 1 | 137.87 | Insecta terrestrial Diptera | 1.133 |  | 6.67321 |
| JFBM49013 | 1 | 184.24 | Fish *Cyprinidae* | 9.281 |  | 1991.35 |
| JFBM49013 | 1 | 184.24 | Fish *Cyprinidae* | 10.447 |  | 2399.45 |
| JFBM49014 | 1 | 147.88 | Nematoda | 0.519 | 20.782 | 11.85303 |
| JFBM49014 | 1 | 147.88 | Trichoptera larvae aquatic | 1.789 | 11.262 | 16.26448 |
| JFBM49014 | 1 | 147.88 | Trichoptera larvae aquatic | 1.399 |  | 8.89963 |
| JFBM49014 | 1 | 147.88 | Trichoptera larvae aquatic | 1.623 |  | 10.77451 |
| JFBM49014 | 1 | 147.88 | Trichoptera larvae aquatic | 1.236 |  | 7.53532 |
| JFBM49014 | 1 | 147.88 | Trichoptera larvae aquatic | 0.898 |  | 4.70626 |
| JFBM49016 | 1 | 164.14 | Fish unidentifiable | 4.531 | 21.257 | 395.94834 |
| JFBM49017 | 1 | 160.99 | Fish | 4.726 |  | 397.1 |
| JFBM49017 | 1 | 160.99 | Insecta terrestrial Plecoptera | 2.212 |  | 15.70444 |
| JFBM49017 | 1 | 160.99 | Insecta terrestrial Plecoptera | 1.275 |  | 7.86175 |
| JFBM49017 | 1 | 160.99 | Insecta terrestrial Plecoptera | 2.242 | 7.495 | 52.68011 |
| JFBM49017 | 4 | 68.1 | Fish unidentifiable | 3.225 |  | 24.18325 |
| JFBM49017 | 4 | 68.1 | Fish unidentifiable | 3.493 |  | 26.42641 |
| JFBM49029 | 1 | 108.46 | Insecta terrestrial | 4.364 |  | 33.71668 |
| JFBM49029 | 1 | 108.46 | Insecta terrestrial | 3.731 |  | 28.41847 |
| JFBM49029 | 1 | 108.46 | Insecta terrestrial | 3.536 |  | 26.78632 |
| JFBM49029 | 1 | 108.46 | Insecta terrestrial | 3.12 |  | 23.3044 |
| JFBM49029 | 1 | 108.46 | Insecta terrestrial | 3.031 | 16.592 | 271.39404 |
| JFBM49029 | 1 | 108.46 | Insecta terrestrial | 3.188 |  | 23.87356 |
| JFBM49029 | 1 | 108.46 | Insecta terrestrial | 4.959 |  | 38.69683 |
| JFBM49029 | 1 | 108.46 | Insecta terrestrial | 3.497 |  | 26.45989 |
| JFBM49029 | 1 | 108.46 | Insecta terrestrial | 2.852 |  | 21.06124 |
| JFBM49029 | 1 | 108.46 | Insecta terrestrial | 2.614 |  | 19.06918 |
| JFBM49029 | 1 | 108.46 | Insecta terrestrial | 3.143 | 9.301 | 49.3217 |
| JFBM49029 | 1 | 108.46 | Insecta terrestrial | 2.72 |  | 19.9564 |
| JFBM49029 | 1 | 108.46 | Insecta terrestrial | 3.332 |  | 25.07884 |
| JFBM49029 | 1 | 108.46 | Insecta terrestrial | 3.433 |  | 25.92421 |
| JFBM49029 | 1 | 108.46 | Insecta terrestrial | 3.53 |  | 26.7361 |
| JFBM49029 | 1 | 108.46 | Insecta terrestrial | 3.363 |  | 25.33831 |
| JFBM49029 | 1 | 108.46 | Insecta terrestrial | 3.813 |  | 29.10481 |
| JFBM49029 | 1 | 108.46 | Insecta terrestrial | 3.077 |  | 22.94449 |
| JFBM49029 | 3 | 111.69 | Insecta terrestrial | 3.69 |  | 28.0753 |
| JFBM49029 | 3 | 111.69 | Insecta terrestrial | 3.57 |  | 27.0709 |
| JFBM49029 | 3 | 111.69 | Insecta terrestrial | 2.366 |  | 16.99342 |
| JFBM49029 | 3 | 111.69 | Insecta terrestrial | 3.59 |  | 27.2383 |
| JFBM49029 | 3 | 111.69 | Insecta terrestrial | 3.718 |  | 28.30966 |
| JFBM49029 | 3 | 111.69 | Insecta terrestrial | 3.128 |  | 23.37136 |
| JFBM49029 | 3 | 111.69 | Insecta terrestrial | 3.77 |  | 28.7449 |
| JFBM49029 | 3 | 111.69 | Insecta terrestrial | 2.535 |  | 18.40795 |
| JFBM49029 | 3 | 111.69 | Insecta terrestrial | 2.625 |  | 19.16125 |
| JFBM49029 | 3 | 111.69 | Insecta terrestrial | 2.908 |  | 21.52996 |
| JFBM49029 | 3 | 111.69 | Insecta terrestrial | 3.771 | 13.234 | 122.05353 |
| JFBM49029 | 4 | 111.31 | Insecta terrestrial | 2.267 | 10.439 | 16.16479 |
| JFBM49029 | 4 | 111.31 | Insecta terrestrial | 2.137 |  | 15.07669 |
| JFBM49029 | 4 | 111.31 | Insecta terrestrial | 2.912 | 13.177 | 63.56394 |
| JFBM49029 | 4 | 111.31 | Insecta terrestrial | 3.543 |  | 26.84491 |
| JFBM49029 | 4 | 111.31 | Insecta terrestrial | 3.547 |  | 26.87839 |
| JFBM49029 | 5 | 120.61 | Insecta terrestrial | 2.725 |  | 19.99825 |
| JFBM49029 | 5 | 120.61 | Insecta terrestrial | 3.144 |  | 23.50528 |
| JFBM49029 | 5 | 120.61 | Insecta terrestrial | 3.445 | 9.329 | 94.30544 |
| JFBM49029 | 6 | 96.87 | Insecta terrestrial | 3.341 |  | 25.15417 |
| JFBM49029 | 6 | 96.87 | Insecta terrestrial | 3.688 | 12.938 | 115.09806 |
| JFBM49029 | 6 | 96.87 | Insecta terrestrial | 4.679 |  | 36.35323 |
| JFBM49029 | 6 | 96.87 | Insecta terrestrial | 3.232 |  | 24.24184 |
| JFBM49029 | 7 | 92.95 | Insecta terrestrial | 3.535 | 15.942 | 164.95978 |
| JFBM49029 | 8 | 105.99 | Insecta terrestrial | 1.803 | 6.255 | 24.07865 |
| JFBM49029 | 8 | 105.99 | Insecta terrestrial | 3.836 |  | 29.29732 |
| JFBM49029 | 8 | 105.99 | Insecta terrestrial | 3.339 |  | 25.13743 |
| JFBM49029 | 8 | 105.99 | Insecta terrestrial | 3.65 | 10.696 | 58.07023 |
| JFBM49029 | 9 | 93.59 | Insecta terrestrial | 1.839 |  | 12.58243 |
| JFBM49179 | 1 | 231.18 | Ephemeroptera nymph aquatic | 3.635 | 17.505 | 361.01056 |
| JFBM49179 | 1 | 231.18 | Insecta larvae aquatic | 0.603 | 5.624 | 1.69954 |
| JFBM49180 | 1 | 93.32 | Terrestrial insecta larvae aquatic | 1.393 | 5.17 | 3.34139 |
| JFBM49180 | 1 | 93.32 | Terrestrial insecta larvae aquatic | 1.004 | 5.548 | 4.71559 |
| JFBM49180 | 1 | 93.32 | Terrestrial insecta larvae aquatic | 1.307 | 6.639 | 5.52307 |
| JFBM49180 | 1 | 93.32 | Insecta (terrestrial) | 1.173 |  | 2.21405 |
| JFBM49180 | 1 | 93.32 | Insecta (terrestrial) | 0.721 |  | 1.12599 |
| JFBM49180 | 1 | 93.32 | Terrestrial insecta larvae aquatic | 0.366 | 3.289 | 0.22666 |
| JFBM49180 | 1 | 93.32 | Terrestrial insecta larvae aquatic | 0.49 | 5.944 | 0.68884 |
| JFBM49180 | 1 | 93.32 | Terrestrial insecta larvae aquatic | 0.649 | 6.724 | 2.97524 |
| JFBM49180 | 1 | 93.32 | Terrestrial insecta larvae aquatic | 0.56 | 5.544 | 1.55602 |
| JFBM49180 | 1 | 93.32 | Terrestrial insecta larvae aquatic | 0.568 |  | 1.94416 |
| JFBM49180 | 1 | 93.32 | Terrestrial insecta larvae aquatic | 0.497 |  | 1.34989 |
| JFBM49180 | 1 | 93.32 | Terrestrial insecta larvae aquatic | 0.368 | 3.702 | 0.64 |
| JFBM49180 | 1 | 93.32 | Terrestrial insecta larvae aquatic | 0.392 |  | 0.47104 |
| JFBM49180 | 1 | 93.32 | Terrestrial insecta larvae aquatic | 0.374 |  | 0.32038 |
| JFBM49180 | 1 | 93.32 | Terrestrial insecta larvae aquatic | 0.819 |  | 4.04503 |
| JFBM49180 | 1 | 93.32 | Terrestrial insecta larvae aquatic | 0.598 |  | 2.19526 |
| JFBM49180 | 1 | 93.32 | Terrestrial insecta larvae aquatic | 0.92 |  | 4.8904 |
| JFBM49180 | 1 | 93.32 | Terrestrial insecta larvae aquatic | 0.911 |  | 4.81507 |
| JFBM49180 | 1 | 93.32 | Copepoda | 0.644 | 2.198 | 1.36016 |
| JFBM49180 | 1 | 93.32 | Copepoda | 0.399 | 0.97 | 0.41152 |
| JFBM49180 | 2 | 107.23 | Terrestrial insecta larvae aquatic | 0.845 |  | 4.26265 |
| JFBM49180 | 3 | 75.93 | Plant material | 4.215 | 4.811 | 116.89956 |
| JFBM49180 | 3 | 75.93 | Amphipoda | 1.291 | 4.596 | 4.62629 |
| JFBM49180 | 3 | 75.93 | Terrestrial insecta larvae aquatic | 0.405 | 4.902 | 1.06826 |
| JFBM49180 | 3 | 75.93 | Terrestrial insecta larvae aquatic | 0.388 | 5.639 | 0.62994 |
| JFBM49180 | 3 | 75.93 | Terrestrial insecta larvae aquatic | 0.25 | 2.609 | 0.2998 |
| JFBM49180 | 3 | 75.93 | Terrestrial insecta larvae aquatic | 0.484 |  | 1.24108 |
| JFBM49180 | 3 | 75.93 | Terrestrial insecta larvae aquatic | 0.505 |  | 1.41685 |
| JFBM49180 | 3 | 75.93 | Terrestrial insecta larvae aquatic | 0.353 | 3.386 | 0.83797 |
| JFBM49180 | 3 | 75.93 | Terrestrial insecta larvae aquatic | 0.385 | 3.966 | 0.60758 |
| JFBM49180 | 3 | 75.93 | Terrestrial insecta larvae aquatic | 0.297 | 2.564 | 0.18419 |
| JFBM49180 | 3 | 75.93 | Amphipoda | 1.158 | 4.868 | 2.27688 |
| JFBM49180 | 3 | 75.93 | Amphipoda | 0.982 |  | 12.04762 |
| JFBM49180 | 3 | 75.93 | Terrestrial insecta larvae aquatic | 0.466 |  | 1.09042 |
| JFBM49180 | 3 | 75.93 | Terrestrial insecta larvae aquatic | 0.369 |  | 0.27853 |
| JFBM49180 | 3 | 75.93 | Terrestrial insecta larvae aquatic | 0.397 | 2.974 | 0.41523 |
| JFBM49180 | 3 | 75.93 | Terrestrial insecta larvae aquatic | 0.332 | 3.1 | 0.34298 |
| JFBM49180 | 3 | 75.93 | Terrestrial insecta larvae aquatic | 0.636 | 4.83 | 3.20146 |
| JFBM49180 | 3 | 75.93 | Terrestrial insecta larvae aquatic | 0.41 | 2.956 | 0.21961 |
| JFBM49180 | 3 | 75.93 | Terrestrial insecta larvae aquatic | 0.39 | 3.349 | 0.60876 |
| JFBM49180 | 3 | 75.93 | Terrestrial insecta larvae aquatic | 0.765 | 4.73 | 2.37427 |
| JFBM49180 | 3 | 75.93 | Terrestrial insecta larvae aquatic | 0.16 |  | 0.21115 |
| JFBM49180 | 3 | 75.93 | Terrestrial insecta larvae aquatic | 0.651 | 4.482 | 1.20122 |
| JFBM49180 | 3 | 75.93 | Insecta terrestrial | 1.095 |  | 2.02628 |
| JFBM49180 | 3 | 75.93 | Insecta terrestrial | 1.208 |  | 2.2983 |
| JFBM49180 | 3 | 75.93 | Insecta terrestrial | 1.004 |  | 1.80723 |
| JFBM49180 | 3 | 75.93 | Insecta terrestrial | 0.967 |  | 1.71816 |
| JFBM49180 | 3 | 75.93 | Insecta terrestrial | 0.851 |  | 1.43893 |
| JFBM49180 | 3 | 75.93 | Insecta terrestrial | 1.556 |  | 3.136 |
| JFBM49180 | 3 | 75.93 | Insecta terrestrial | 1.767 |  | 3.64392 |
| JFBM49180 | 3 | 75.93 | Insecta terrestrial | 1.475 |  | 2.94102 |
| JFBM49180 | 3 | 75.93 | Insecta terrestrial | 1.368 |  | 2.68345 |
| JFBM49180 | 3 | 75.93 | Egg | 1.909 | 4.054 | 18.95518 |
| JFBM49180 | 3 | 75.93 | Insecta terrestrial | 1.041 |  | 1.8963 |
| JFBM49180 | 3 | 75.93 | Amphipoda | 0.666 | 1.824 | 2.06023 |
| JFBM49180 | 3 | 75.93 | Amphipoda | 0.654 | 2.2 | 4.04252 |
| JFBM49180 | 3 | 75.93 | Terrestrial insecta larvae aquatic | 0.319 | 3.408 | 0.38285 |
| JFBM49180 | 3 | 75.93 | Copepoda | 0.278 | 0.749 | 0.194 |
| JFBM49180 | 3 | 75.93 | Terrestrial insecta larvae aquatic | 0.361 | 3.819 | 0.58454 |
| JFBM49180 | 3 | 75.93 | Terrestrial insecta larvae aquatic | 0.264 | 3.091 | 0.27184 |
| JFBM49180 | 3 | 75.93 | Terrestrial insecta larvae aquatic | 0.376 |  | 0.33712 |
| JFBM49180 | 3 | 75.93 | Terrestrial insecta larvae aquatic | 0.269 |  | 0.24579 |
| JFBM49180 | 3 | 75.93 | Terrestrial insecta larvae aquatic | 0.333 |  | 0.26613 |
| JFBM49180 | 3 | 75.93 | Terrestrial insecta larvae aquatic | 0.304 |  | 0.25691 |
| JFBM49180 | 3 | 75.93 | Daphnia | 0.454 | 0.74 | 0.17496 |
| JFBM49180 | 3 | 75.93 | Daphnia | 0.471 | 0.589 | 0.10903 |
| JFBM49180 | 3 | 75.93 | Daphnia | 0.329 | 0.523 | 0.06312 |
| JFBM49180 | 3 | 75.93 | Daphnia | 0.339 | 0.591 | 0.03739 |
| JFBM49180 | 3 | 75.93 | Terrestrial insecta larvae aquatic | 0.27 |  | 0.24611 |
| JFBM49180 | 3 | 75.93 | Terrestrial insecta larvae aquatic | 0.472 |  | 1.14064 |
| JFBM49180 | 3 | 75.93 | Copepoda | 0.301 | 0.777 | 0.07171 |
| JFBM49180 | 3 | 75.93 | Copepoda | 0.398 |  | 0.03634 |
| JFBM49180 | 4 | 79.02 | Terrestrial insecta larvae aquatic | 1.03 | 6.523 | 5.74233 |
| JFBM49180 | 4 | 79.02 | Terrestrial insecta larvae aquatic | 0.79 | 5.322 | 4.37317 |
| JFBM49180 | 4 | 79.02 | Terrestrial insecta larvae aquatic | 0.199 | 2.048 | 0.16824 |
| JFBM49180 | 4 | 79.02 | Terrestrial insecta larvae aquatic | 0.49 | 4.428 | 0.8201 |
| JFBM49180 | 4 | 79.02 | Terrestrial insecta larvae aquatic | 0.52 | 3.864 | 0.85757 |
| JFBM49180 | 4 | 79.02 | Terrestrial insecta larvae aquatic | 0.585 | 5.06 | 1.76502 |
| JFBM49180 | 4 | 79.02 | Terrestrial insecta larvae aquatic | 0.711 | 4.93 | 3.59801 |
| JFBM49180 | 4 | 79.02 | Terrestrial insecta larvae aquatic | 0.318 | 4.099 | 0.73461 |
| JFBM49180 | 4 | 79.02 | Terrestrial insecta larvae aquatic | 0.242 | 2.16 | 0.06156 |
| JFBM49180 | 4 | 79.02 | Terrestrial insecta larvae aquatic | 0.643 | 3.719 | 2.53542 |
| JFBM49180 | 4 | 79.02 | Terrestrial insecta larvae aquatic | 0.394 | 2.612 | 0.2239 |
| JFBM49180 | 4 | 79.02 | Terrestrial insecta larvae aquatic | 0.433 | 3.633 | 0.86791 |
| JFBM49180 | 4 | 79.02 | Terrestrial insecta larvae aquatic | 0.327 |  | 0.26422 |
| JFBM49180 | 4 | 79.02 | Terrestrial insecta larvae aquatic | 0.675 | 4.13 | 1.3623 |
| JFBM49180 | 4 | 79.02 | Terrestrial insecta larvae aquatic | 0.345 |  | 0.07765 |
| JFBM49180 | 4 | 79.02 | Terrestrial insecta larvae aquatic | 0.377 |  | 0.34549 |
| JFBM49180 | 4 | 79.02 | Terrestrial insecta larvae aquatic | 0.423 | 3.784 | 0.62287 |
| JFBM49180 | 4 | 79.02 | Terrestrial insecta larvae aquatic | 0.495 | 3.683 | 0.51824 |
| JFBM49180 | 4 | 79.02 | Terrestrial insecta larvae aquatic | 0.576 |  | 2.01112 |
| JFBM49180 | 4 | 79.02 | Terrestrial insecta larvae aquatic | 0.465 |  | 1.08205 |
| JFBM49180 | 4 | 79.02 | Terrestrial insecta larvae aquatic | 0.782 | 4.417 | 2.55678 |
| JFBM49180 | 4 | 79.02 | Amphipoda | 1.285 | 4.699 | 5.91437 |
| JFBM49180 | 4 | 79.02 | Amphipoda | 1.763 | 4.885 | 8.04982 |
| JFBM49180 | 4 | 79.02 | Terrestrial insecta larvae aquatic | 0.746 | 5.399 | 2.69998 |
| JFBM49180 | 4 | 79.02 | Terrestrial insecta larvae aquatic | 0.249 | 3.325 | 0.31947 |
| JFBM49180 | 4 | 79.02 | Terrestrial insecta larvae aquatic | 0.762 | 6.071 | 3.95879 |
| JFBM49180 | 4 | 79.02 | Terrestrial insecta larvae aquatic | 0.446 |  | 0.92302 |
| JFBM49180 | 4 | 79.02 | Terrestrial insecta larvae aquatic | 0.535 |  | 1.66795 |
| JFBM49180 | 4 | 79.02 | Terrestrial insecta larvae aquatic | 0.236 | 3.173 | 0.4571 |
| JFBM49180 | 4 | 79.02 | Terrestrial insecta larvae aquatic | 0.352 |  | 0.13624 |
| JFBM49180 | 4 | 79.02 | Terrestrial insecta larvae aquatic | 0.811 | 5.177 | 1.96609 |
| JFBM49180 | 4 | 79.02 | Terrestrial insecta larvae aquatic | 0.356 | 3.803 | 0.48599 |
| JFBM49180 | 4 | 79.02 | Terrestrial insecta larvae aquatic | 0.5 |  | 1.375 |
| JFBM49180 | 4 | 79.02 | Terrestrial insecta larvae aquatic | 0.359 | 2.624 | 0.28354 |
| JFBM49180 | 4 | 79.02 | Terrestrial insecta larvae aquatic | 0.561 | 4.398 | 1.493 |
| JFBM49180 | 4 | 79.02 | Terrestrial insecta larvae aquatic | 0.625 | 3.748 | 0.83257 |
| JFBM49180 | 4 | 79.02 | Terrestrial insecta larvae aquatic | 0.547 |  | 1.76839 |
| JFBM49180 | 4 | 79.02 | Terrestrial insecta larvae aquatic | 0.702 |  | 3.06574 |
| JFBM49180 | 4 | 79.02 | Terrestrial insecta larvae aquatic | 0.298 |  | 0.255 |
| JFBM49180 | 4 | 79.02 | Terrestrial insecta larvae aquatic | 0.693 | 5.99 | 0.88951 |
| JFBM49180 | 4 | 79.02 | Terrestrial insecta larvae aquatic | 0.63 | 3.669 | 1.49884 |
| JFBM49180 | 4 | 79.02 | Terrestrial insecta larvae aquatic | 0.77 | 4.504 | 2.36267 |
| JFBM49180 | 4 | 79.02 | Terrestrial insecta larvae aquatic | 0.858 |  | 4.37146 |
| JFBM49180 | 4 | 79.02 | Terrestrial insecta larvae aquatic | 0.406 | 3.642 | 0.51837 |
| JFBM49180 | 4 | 79.02 | Terrestrial insecta larvae aquatic | 0.501 |  | 1.38337 |
| JFBM49180 | 4 | 79.02 | Ostracoda | 0.334 | 0.526 | 0.07583 |
| JFBM49180 | 4 | 79.02 | Terrestrial insecta larvae aquatic | 0.642 | 4.827 | 2.45571 |
| JFBM49180 | 4 | 79.02 | Terrestrial insecta larvae aquatic | 0.369 | 3.719 | 0.61925 |
| JFBM49180 | 4 | 79.02 | Terrestrial insecta larvae aquatic | 0.543 | 4.991 | 1.1831 |
| JFBM49180 | 4 | 79.02 | Terrestrial insecta larvae aquatic | 0.312 | 2.391 | 0.37769 |
| JFBM49180 | 4 | 79.02 | Terrestrial insecta larvae aquatic | 0.407 | 4.786 | 0.57172 |
| JFBM49180 | 4 | 79.02 | Terrestrial insecta larvae aquatic | 0.592 | 3.452 | 1.03412 |
| JFBM49180 | 4 | 79.02 | Terrestrial insecta larvae aquatic | 0.394 | 2.408 | 0.37109 |
| JFBM49180 | 4 | 79.02 | Terrestrial insecta larvae aquatic | 0.531 | 5.229 | 1.73224 |
| JFBM49180 | 4 | 79.02 | Terrestrial insecta larvae aquatic | 0.466 | 4.219 | 0.33933 |
| JFBM49180 | 4 | 79.02 | Terrestrial insecta larvae aquatic | 0.388 | 3.403 | 0.45001 |
| JFBM49180 | 4 | 79.02 | Terrestrial insecta larvae aquatic | 0.603 | 5.531 | 1.43873 |
| JFBM49180 | 4 | 79.02 | Terrestrial insecta larvae aquatic | 0.487 |  | 1.26619 |
| JFBM49180 | 4 | 79.02 | Terrestrial insecta larvae aquatic | 0.222 |  | 0.23085 |
| JFBM49180 | 4 | 79.02 | Terrestrial insecta larvae aquatic | 0.33 | 3.514 | 0.4144 |
| JFBM49180 | 4 | 79.02 | Terrestrial insecta larvae aquatic | 0.338 | 4.594 | 1.52579 |
| JFBM49180 | 4 | 79.02 | Terrestrial insecta larvae aquatic | 0.662 |  | 2.73094 |
| JFBM49180 | 4 | 79.02 | Terrestrial insecta larvae aquatic | 0.391 | 4.043 | 0.7379 |
| JFBM49180 | 4 | 79.02 | Terrestrial insecta larvae aquatic | 0.503 | 3.42 | 0.71071 |
| JFBM49180 | 4 | 79.02 | Terrestrial insecta larvae aquatic | 0.58 | 4.027 | 0.91334 |
| JFBM49180 | 4 | 79.02 | Terrestrial insecta larvae aquatic | 0.277 | 2.89 | 0.26739 |
| JFBM49180 | 4 | 79.02 | Terrestrial insecta larvae aquatic | 0.724 | 4.607 | 2.09918 |
| JFBM49180 | 4 | 79.02 | Terrestrial insecta larvae aquatic | 0.558 | 4.644 | 2.35191 |
| JFBM49180 | 4 | 79.02 | Copepoda | 0.368 | 0.751 | 0.13173 |
| JFBM49180 | 4 | 79.02 | Copepoda | 0.436 | 0.702 | 0.14614 |
| JFBM49180 | 4 | 79.02 | Copepoda | 0.374 |  | 0.02837 |
| JFBM49180 | 4 | 79.02 | Copepoda | 0.329 | 0.673 | 0.11779 |
| JFBM49180 | 4 | 79.02 | Copepoda | 0.36 |  | 0.02456 |
| JFBM49181 | 1 | 207.28 | Terrestrial insecta larvae aquatic | 3.281 |  | 24.65197 |
| JFBM49181 | 1 | 207.28 | *Dorosoma sp.* | 18.357 | 78.788 | 21438.3222 |
| JFBM49181 | 2 | 95.18 | Terrestrial insecta larvae aquatic | 0.506 |  | 1.42522 |
| JFBM49181 | 2 | 95.18 | Terrestrial insecta larvae aquatic | 0.438 |  | 0.85606 |
| JFBM49181 | 2 | 95.18 | Terrestrial insecta larvae aquatic | 0.825 |  | 4.09525 |
| JFBM49181 | 2 | 95.18 | Terrestrial insecta larvae aquatic | 1.033 |  | 5.83621 |
| JFBM49181 | 2 | 95.18 | Terrestrial insecta larvae aquatic | 0.642 |  | 2.56354 |
| JFBM49181 | 2 | 95.18 | Terrestrial insecta larvae aquatic | 0.695 |  | 3.00715 |
| JFBM49181 | 2 | 95.18 | Terrestrial insecta larvae aquatic | 0.797 |  | 3.86089 |
| JFBM49181 | 2 | 95.18 | Terrestrial insecta larvae aquatic | 0.791 |  | 3.81067 |
| JFBM49181 | 2 | 95.18 | Terrestrial insecta larvae aquatic | 0.607 |  | 2.27059 |
| JFBM49181 | 2 | 95.18 | Terrestrial insecta larvae aquatic | 0.746 |  | 3.43402 |
| JFBM49181 | 2 | 95.18 | Terrestrial insecta larvae aquatic | 0.616 |  | 2.34592 |
| JFBM49181 | 3 | 96.87 | Terrestrial insecta larvae aquatic | 0.572 |  | 1.97764 |
| JFBM49181 | 3 | 96.87 | Terrestrial insecta larvae aquatic | 0.387 |  | 0.42919 |
| JFBM49181 | 3 | 96.87 | Terrestrial insecta larvae aquatic | 0.74 |  | 3.3838 |
| JFBM49181 | 3 | 96.87 | Terrestrial insecta larvae aquatic | 1.21 |  | 7.3177 |
| JFBM49181 | 3 | 96.87 | Terrestrial insecta larvae aquatic | 1.022 |  | 5.74414 |
| JFBM49181 | 3 | 96.87 | Terrestrial insecta larvae aquatic | 0.898 |  | 4.70626 |
| JFBM49181 | 3 | 96.87 | Terrestrial insecta larvae aquatic | 0.567 |  | 1.93579 |
| JFBM49181 | 3 | 96.87 | Insecta terrestrial | 0.701 |  | 1.07785 |
| JFBM49181 | 3 | 96.87 | Insecta terrestrial | 0.764 |  | 1.2295 |
| JFBM49181 | 3 | 96.87 | Insecta terrestrial | 0.583 |  | 0.7938 |
| JFBM49181 | 3 | 96.87 | Insecta terrestrial | 0.772 |  | 1.24876 |
| JFBM49181 | 3 | 96.87 | Insecta terrestrial | 0.804 |  | 1.32579 |
| JFBM49181 | 3 | 96.87 | Terrestrial insecta larvae aquatic | 0.89 |  | 4.6393 |
| JFBM49181 | 3 | 96.87 | Terrestrial insecta larvae aquatic | 1.309 |  | 8.14633 |
| JFBM49181 | 3 | 96.87 | Terrestrial insecta larvae aquatic | 1.192 |  | 7.16704 |
| JFBM49181 | 3 | 96.87 | Terrestrial insecta larvae aquatic | 1.332 |  | 8.33884 |
| JFBM49181 | 3 | 96.87 | Terrestrial insecta larvae aquatic | 0.849 |  | 4.29613 |
| JFBM49181 | 3 | 96.87 | Terrestrial insecta larvae aquatic | 1.358 |  | 8.55646 |
| JFBM49181 | 3 | 96.87 | Terrestrial insecta larvae aquatic | 0.865 |  | 4.43005 |
| JFBM49181 | 3 | 96.87 | Terrestrial insecta larvae aquatic | 0.999 |  | 5.55163 |
| JFBM49181 | 3 | 96.87 | Terrestrial insecta larvae aquatic | 0.881 |  | 4.56397 |
| JFBM49181 | 3 | 96.87 | Terrestrial insecta larvae aquatic | 0.56 |  | 1.8772 |
| JFBM49181 | 3 | 96.87 | Terrestrial insecta larvae aquatic | 0.618 |  | 2.36266 |
| JFBM49181 | 3 | 96.87 | Terrestrial insecta larvae aquatic | 0.707 |  | 3.10759 |
| JFBM49181 | 3 | 96.87 | Terrestrial insecta larvae aquatic | 0.541 |  | 1.71817 |
| JFBM49181 | 3 | 96.87 | Terrestrial insecta larvae aquatic | 0.433 |  | 0.81421 |
| JFBM49183 | 1 | 171.29 | Fish | 5.299 |  | 597.65 |
| JFBM49183 | 1 | 171.29 | Fish | 4.541 |  | 332.35 |
| JFBM49183 | 1 | 171.29 | Fish | 1.986 |  | 80.22926 |
| JFBM49183 | 1 | 171.29 | Fish | 1.911 |  | 75.13601 |
| JFBM49183 | 1 | 171.55 | Fish | 3.229 |  | 164.64139 |
| JFBM49183 | 1 | 171.55 | Fish | 2.662 |  | 126.13642 |
| JFBM49183 | 1 | 171.55 | Fish | 3.179 |  | 161.24589 |
| JFBM49183 | 1 | 171.55 | Fish | 4.867 |  | 446.45 |
| JFBM49183 | 1 | 171.55 | Fish | 2.614 |  | 122.87674 |
| JFBM49183 | 1 | 171.55 | Fish | 2.756 |  | 132.51996 |
| JFBM49183 | 1 | 171.55 | Fish | 2.469 |  | 113.02979 |
| JFBM49183 | 1 | 171.55 | Fish | 3.462 |  | 180.46442 |
| JFBM49183 | 3 | 61.95 | Fish | 2.228 |  | 15.83836 |
| JFBM49183 | 3 | 61.95 | Fish | 2.957 |  | 21.94009 |
| JFBM49192 | 1 | 144.41 | Fish | 6.567 |  | 520.314 |
| JFBM49192 | 1 | 144.41 | Fish | 7.705 | 42.203 | 1075.52166 |
| JFBM49192 | 1 | 144.41 | Fish | 7.958 | 49.701 | 1650.0431 |
| JFBM49192 | 1 | 144.41 | Fish | 8.034 | 46.611 | 1650.63556 |
| JFBM49192 | 2 | 144.41 | Fish | 5.811 |  | 776.85 |
| JFBM49192 | 2 | 144.41 | Fish | 7.073 | 44.816 | 2118.32508 |
| JFBM49192 | 4 | 133.64 | Terrestrial insecta larvae aquatic | 0.452 |  | 0.97324 |
| JFBM49192 | 4 | 133.64 | Terrestrial insecta larvae aquatic | 0.461 | 4.491 | 0.61896 |
| JFBM49192 | 4 | 133.64 | Terrestrial insecta larvae aquatic | 0.541 |  | 1.71817 |
| JFBM49192 | 4 | 133.64 | Terrestrial insecta larvae aquatic | 0.622 | 5.097 | 0.73549 |
| JFBM49192 | 4 | 133.64 | Terrestrial insecta larvae aquatic | 0.701 |  | 3.05737 |
| JFBM49192 | 4 | 133.64 | Terrestrial insecta larvae aquatic | 0.554 | 4.504 | 1.49523 |
| JFBM49192 | 4 | 133.64 | Terrestrial insecta larvae aquatic | 0.254 | 3.254 | 0.29254 |
| JFBM49192 | 4 | 133.64 | Terrestrial insecta larvae aquatic | 0.314 |  | 0.26009 |
| JFBM49192 | 4 | 133.64 | Terrestrial insecta larvae aquatic | 0.388 | 3.704 | 0.72114 |
| JFBM49192 | 4 | 133.64 | Terrestrial insecta larvae aquatic | 0.517 |  | 1.51729 |
| JFBM49192 | 4 | 133.64 | Terrestrial insecta larvae aquatic | 0.653 | 4.582 | 0.87577 |
| JFBM49192 | 4 | 133.64 | Terrestrial insecta larvae aquatic | 0.372 |  | 0.30364 |
| JFBM49192 | 4 | 133.64 | Terrestrial insecta larvae aquatic | 0.416 | 5.054 | 0.32497 |
| JFBM49192 | 4 | 133.64 | Terrestrial insecta larvae aquatic | 0.27 |  | 0.24611 |
| JFBM49192 | 4 | 133.64 | Terrestrial insecta larvae aquatic | 0.66 |  | 2.7142 |
| JFBM49192 | 4 | 133.64 | Terrestrial insecta larvae aquatic | 0.998 |  | 5.54326 |
| JFBM49192 | 4 | 133.64 | Terrestrial insecta larvae aquatic | 0.38 | 3.028 | 0.18327 |
| JFBM49192 | 4 | 133.64 | Terrestrial insecta larvae aquatic | 0.683 |  | 2.90671 |
| JFBM49192 | 4 | 133.64 | Terrestrial insecta larvae aquatic | 0.331 | 2.9 | 0.4297 |
| JFBM49192 | 4 | 133.64 | Terrestrial insecta larvae aquatic | 0.559 |  | 1.86883 |
| JFBM49192 | 4 | 133.64 | Terrestrial insecta larvae aquatic | 0.544 |  | 1.74328 |
| JFBM49192 | 4 | 133.64 | Terrestrial insecta larvae aquatic | 0.305 |  | 0.25723 |
| JFBM49192 | 4 | 133.64 | Terrestrial insecta larvae aquatic | 0.605 | 5.292 | 2.11536 |
| JFBM49192 | 4 | 133.64 | Terrestrial insecta larvae aquatic | 0.435 | 5.065 | 0.36308 |
| JFBM49192 | 4 | 133.64 | Terrestrial insecta larvae aquatic | 0.44 |  | 0.8728 |
| JFBM49192 | 4 | 133.64 | Terrestrial insecta larvae aquatic | 0.452 | 4.772 | 0.81423 |
| JFBM49192 | 4 | 133.64 | Terrestrial insecta larvae aquatic | 0.514 |  | 1.49218 |
| JFBM49192 | 4 | 133.64 | Terrestrial insecta larvae aquatic | 0.66 | 8.76 | 1.46913 |
| JFBM49192 | 4 | 133.64 | Terrestrial insecta larvae aquatic | 0.582 | 7.319 | 2.88432 |
| JFBM49192 | 4 | 133.64 | Terrestrial insecta larvae aquatic | 0.469 |  | 1.11553 |
| JFBM49192 | 4 | 133.64 | Terrestrial insecta larvae aquatic | 0.575 | 6.867 | 1.22183 |
| JFBM49192 | 4 | 133.64 | Terrestrial insecta larvae aquatic | 0.486 | 5.721 | 1.50491 |
| JFBM49192 | 4 | 133.64 | Terrestrial insecta larvae aquatic | 0.76 | 5.493 | 2.59626 |
| JFBM49192 | 4 | 133.64 | Terrestrial insecta larvae aquatic | 0.345 | 3.292 | 0.45312 |
| JFBM49192 | 4 | 133.64 | Terrestrial insecta larvae aquatic | 0.556 |  | 1.84372 |
| JFBM49192 | 4 | 133.64 | Terrestrial insecta larvae aquatic | 0.434 | 3.696 | 0.3635 |
| JFBM49192 | 4 | 133.64 | Terrestrial insecta larvae aquatic | 0.763 | 6.702 | 2.889 |
| JFBM49192 | 4 | 133.64 | Terrestrial insecta larvae aquatic | 0.726 | 5.472 | 3.15547 |
| JFBM49192 | 4 | 133.64 | Terrestrial insecta larvae aquatic | 0.648 | 7.548 | 4.91302 |
| JFBM49192 | 4 | 133.64 | Terrestrial insecta larvae aquatic | 0.464 |  | 1.07368 |
| JFBM49192 | 4 | 133.64 | Terrestrial insecta larvae aquatic | 0.573 | 6.099 | 1.88937 |
| JFBM49192 | 4 | 133.64 | Terrestrial insecta larvae aquatic | 0.405 |  | 0.57985 |
| JFBM49192 | 4 | 133.64 | Terrestrial insecta larvae aquatic | 0.391 | 3.399 | 0.49153 |
| JFBM49192 | 4 | 133.64 | Terrestrial insecta larvae aquatic | 0.493 |  | 1.31641 |
| JFBM49192 | 4 | 133.64 | Terrestrial insecta larvae aquatic | 0.387 |  | 0.42919 |
| JFBM49192 | 4 | 133.64 | Terrestrial insecta larvae aquatic | 0.358 |  | 0.18646 |
| JFBM49192 | 4 | 133.64 | Terrestrial insecta larvae aquatic | 0.589 | 4.959 | 1.42188 |
| JFBM49192 | 4 | 133.64 | Terrestrial insecta larvae aquatic | 0.417 | 4.024 | 0.58128 |
| JFBM49192 | 5 | 125.4 | Terrestrial insecta larvae aquatic | 0.559 | 6.937 | 3.3806 |
| JFBM49192 | 5 | 125.4 | Terrestrial insecta larvae aquatic | 0.435 | 4.304 | 1.17241 |
| JFBM49192 | 5 | 125.4 | Terrestrial insecta larvae aquatic | 0.538 | 4.329 | 2.24566 |
| JFBM49192 | 7 | 82.33 | Terrestrial insecta larvae aquatic | 0.44 |  | 0.8728 |
| JFBM49192 | 7 | 82.33 | Insecta terrestrial | 0.657 |  | 0.97193 |
| JFBM49192 | 9 | 83.92 | Terrestrial insecta larvae aquatic | 0.315 |  | 0.07489 |
| JFBM49192 | 9 | 83.92 | Terrestrial insecta larvae aquatic | 0.453 |  | 0.98161 |
| JFBM49192 | 9 | 83.92 | Terrestrial insecta larvae aquatic | 0.368 |  | 0.27016 |
| JFBM49192 | 9 | 83.92 | Terrestrial insecta larvae aquatic | 0.201 |  | 0.00476 |
| JFBM49192 | 9 | 83.92 | Terrestrial insecta larvae aquatic | 0.329 |  | 0.01783 |
| JFBM49192 | 9 | 83.92 | Terrestrial insecta larvae aquatic | 0.518 |  | 1.52566 |
| JFBM49192 | 9 | 83.92 | Terrestrial insecta larvae aquatic | 0.375 |  | 0.32875 |
| JFBM49192 | 9 | 83.92 | Terrestrial insecta larvae aquatic | 0.424 |  | 0.73888 |
| JFBM49192 | 9 | 83.92 | Terrestrial insecta larvae aquatic | 0.202 | 2.425 | 0.33345 |
| JFBM49192 | 9 | 83.92 | Terrestrial insecta larvae aquatic | 0.436 | 4.231 | 0.99773 |
| JFBM49192 | 9 | 83.92 | Terrestrial insecta larvae aquatic | 0.296 |  | 0.25437 |
| JFBM49192 | 9 | 83.92 | Terrestrial insecta larvae aquatic | 0.379 |  | 0.36223 |
| JFBM49192 | 9 | 83.92 | Terrestrial insecta larvae aquatic | 0.374 |  | 0.32038 |
| JFBM49192 | 9 | 83.92 | Terrestrial insecta larvae aquatic | 0.613 |  | 2.32081 |
| JFBM49192 | 11 | 50.44 | Cladocera | 0.24 | 0.4 | 0.05629 |
| JFBM49192 | 11 | 50.44 | Cladocera | 0.189 | 0.446 | 0.0233 |
| JFBM49192 | 11 | 50.44 | Cladocera | 0.217 | 0.478 | 0.04626 |
| JFBM49192 | 11 | 50.44 | Cladocera | 0.353 | 0.635 | 0.07727 |
| JFBM49192 | 11 | 50.44 | Cladocera | 0.376 | 0.502 | 0.13083 |
| JFBM49192 | 11 | 50.44 | Cladocera | 0.331 | 0.463 | 0.04395 |
| JFBM49192 | 11 | 50.44 | Cladocera | 0.289 | 0.377 | 0.01883 |
| JFBM49192 | 11 | 50.44 | Cladocera | 0.208 | 0.331 | 0.02153 |
| JFBM49192 | 11 | 50.44 | Cladocera | 0.278 | 0.503 | 0.04922 |
| JFBM49192 | 11 | 50.44 | Cladocera | 0.378 | 0.561 | 0.09245 |
| JFBM49192 | 11 | 50.44 | Cladocera | 0.262 |  | 0.00893 |
| JFBM49192 | 11 | 50.44 | Cladocera | 0.416 | 0.663 | 0.12246 |
| JFBM49192 | 11 | 50.44 | Cladocera | 0.366 |  | 0.02612 |
| JFBM49192 | 11 | 50.44 | Copepoda | 0.292 |  | 0.01217 |
| JFBM49192 | 11 | 50.44 | Copepoda | 0.33 | 0.665 | 0.07803 |
| JFBM49192 | 11 | 50.44 | Copepoda | 0.353 |  | 0.02285 |
| JFBM49192 | 12 | 68.3 | Insecta terrestrial | 0.782 |  | 3.73534 |
| JFBM49192 | 12 |  | Formicidae | 0.705 |  | 1.08748 |
| JFBM49192 | 12 |  | Formicidae | 0.638 |  | 0.92619 |
| JFBM49192 | 13 | 81.46 | Insecta terrestrial | 2.609 |  | 19.02733 |
| JFBM49192 | 13 | 81.46 | Insecta terrestrial | 2.622 |  | 19.13614 |
| JFBM49192 | 14 | 81.46 | Insecta aquatic | 0.84 |  | 4.2208 |
| JFBM49192 | 14 | 81.46 | Insecta aquatic | 0.748 |  | 3.45076 |
| JFBM49192 | 14 | 85.9 | Insecta aquatic | 0.732 |  | 3.31684 |
| JFBM49192 | 14 | 85.9 | Insecta unidentifiable | 1.033 |  | 5.83621 |
| JFBM49192 | 14 | 85.9 | Insecta unidentifiable | 0.726 |  | 3.26662 |
| JFBM49192 | 14 | 85.9 | Insecta unidentifiable | 1.013 |  | 5.66881 |
| JFBM49455 | 1 | 94.87 | Fish *Notropis* | 3.788 | 30.797 | 805.34895 |
| JFBM49455 | 1 | 94.87 | Fish *Notropis* | 2.965 |  | 22.00705 |
| JFBM49455 | 1 | 94.87 | Insecta terrestrial | 0.98 |  | 5.3926 |
| JFBM49455 | 1 | 94.87 | Insecta terrestrial | 0.989 |  | 5.46793 |
| JFBM49455 | 1 | 94.87 | Insecta terrestrial | 0.895 |  | 4.68115 |
| JFBM49455 | 1 | 94.87 | Fish | 3.953 | 26.953 | 510.16479 |
| JFBM49455 | 1 | 94.87 | Insecta terrestrial | 0.921 |  | 4.89877 |
| JFBM49455 | 1 | 94.87 | Insecta terrestrial | 0.803 |  | 3.91111 |
| JFBM49455 | 1 | 94.87 | Insecta terrestrial | 1.011 |  | 5.65207 |
| JFBM49455 | 2 | 88.14 | Fish *Notropis* | 5.446 |  | 649.1 |
| JFBM49455 | 2 | 88.14 | Fish *Notropis* | 7.073 |  | 1218.55 |
| JFBM49455 | 2 | 88.14 | Fish *Notropis* | 3.769 | 26.963 | 225.06517 |
| JFBM49455 | 2 | 88.14 | Fish *Notropis* | 4.952 |  | 476.2 |
| JFBM49455 | 2 | 88.14 | Insecta terrestrial | 0.912 |  | 4.82344 |
| JFBM49455 | 2 | 88.14 | Insecta terrestrial | 1.216 |  | 7.36792 |
| JFBM49455 | 2 | 88.14 | Coleoptera terrestrial | 1.475 | 3.744 | 8.71252 |
| JFBM49455 | 2 | 88.14 | Fish *Notropis* | 5.534 |  | 679.9 |
| JFBM49455 | 2 | 88.14 | Fish unidentifiable | 6.306 |  | 950.1 |
| JFBM49455 | 2 | 88.14 | Fish Notropis | 4.369 |  | 272.15 |
| JFBM49455 | 2 | 88.14 | Fish Notropis | 4.891 |  | 454.85 |
| JFBM49455 | 2 | 88.14 | Insecta terrestrial | 0.882 |  | 4.57234 |
| JFBM49455 | 2 | 88.14 | Insecta terrestrial | 1.321 |  | 8.24677 |
| JFBM49455 | 2 | 88.14 | Insecta Coleoptera | 1.472 |  | 9.51064 |
| JFBM49455 | 3 | 72.25 | Cladocera | 0.53 | 0.677 | 0.16748 |
| JFBM49455 | 3 | 72.25 | Cladocera | 0.498 |  | 0.31856 |
| JFBM49455 | 3 | 72.25 | Cladocera | 0.622 | 0.868 | 0.39023 |
| JFBM49455 | 3 | 72.25 | Cladocera | 0.384 |  | 0.28234 |
| JFBM49455 | 3 | 72.25 | Cladocera | 0.417 |  | 0.29282 |
| JFBM49455 | 3 | 72.25 | Cladocera | 0.368 |  | 0.27725 |
| JFBM49455 | 3 | 72.25 | Cladocera | 0.362 |  | 0.27534 |
| JFBM49455 | 3 | 72.25 | Cladocera | 0.486 |  | 0.31475 |
| JFBM49455 | 3 | 72.25 | Cladocera | 0.381 |  | 0.28138 |
| JFBM49455 | 3 | 72.25 | Cladocera | 0.411 |  | 0.29106 |
| JFBM49455 | 3 | 72.25 | Cladocera | 0.382 |  | 0.28165 |
| JFBM49455 | 3 | 72.25 | Cladocera | 0.299 |  | 0.25538 |
| JFBM49455 | 3 | 72.25 | Cladocera | 0.314 |  | 0.26005 |
| JFBM49455 | 3 | 72.25 | Cladocera | 0.274 | 0.551 | 0.0949 |
| JFBM49455 | 3 | 72.25 | Cladocera | 0.331 | 0.533 | 0.16525 |
| JFBM49455 | 3 | 72.25 | Cladocera | 0.48 |  | 0.31273 |
| JFBM49455 | 3 | 72.25 | Cladocera | 0.359 | 0.568 | 0.21542 |
| JFBM49455 | 3 | 72.25 | Cladocera | 0.428 |  | 0.29625 |
| JFBM49455 | 3 | 72.25 | Insecta terrestrial | 1.017 |  | 5.70229 |
| JFBM49455 | 3 | 72.25 | Cladocera | 0.454 | 0.869 | 0.30407 |
| JFBM49455 | 3 | 72.25 | Copepoda | 0.319 | 0.719 | 0.11795 |
| JFBM49455 | 3 | 72.25 | Cladocera | 0.329 | 0.581 | 0.10077 |
| JFBM49455 | 3 | 72.25 | Insecta terrestrial | 0.674 |  | 2.83138 |
| JFBM49455 | 3 | 72.25 | Cladocera | 0.562 | 0.663 | 0.14002 |
| JFBM49455 | 3 | 72.25 | Cladocera | 0.476 | 0.708 | 0.13486 |
| JFBM49455 | 3 | 72.25 | Cladocera | 0.586 | 0.959 | 0.28049 |
| JFBM49455 | 3 | 72.25 | Cladocera | 0.329 | 0.61 | 0.07232 |
| JFBM49455 | 3 | 72.25 | Cladocera | 0.328 | 0.591 | 0.15321 |
| JFBM49455 | 3 | 72.25 | Cladocera | 0.329 | 0.618 | 0.08157 |
| JFBM49455 | 3 | 72.25 | Cladocera | 0.364 | 0.608 | 0.16643 |
| JFBM49455 | 3 | 72.25 | Cladocera | 0.472 |  | 0.3103 |
| JFBM49455 | 3 | 72.25 | Cladocera | 0.383 |  | 0.28202 |
| JFBM49455 | 3 | 72.25 | Cladocera | 0.373 | 0.601 | 0.09202 |
| JFBM49455 | 3 | 72.25 | Cladocera | 0.323 |  | 0.263 |
| JFBM49455 | 3 | 72.25 | Cladocera | 0.322 |  | 0.2627 |
| JFBM49455 | 3 | 72.25 | Cladocera | 0.319 |  | 0.2618 |
| JFBM49455 | 3 | 72.25 | Cladocera | 0.292 | 0.611 | 0.06827 |
| JFBM49455 | 3 | 72.25 | Cladocera | 0.303 | 0.655 | 0.11405 |
| JFBM49455 | 3 | 72.25 | Cladocera | 0.469 | 0.655 | 0.191 |
| JFBM49455 | 3 | 72.25 | Cladocera | 0.564 | 0.973 | 0.83989 |
| JFBM49455 | 3 | 72.25 | Cladocera | 0.535 | 1.051 | 0.27099 |
| JFBM49455 | 3 | 72.25 | Insecta terrestrial | 0.947 |  | 5.11639 |
| JFBM49455 | 3 | 72.25 | Copepoda | 0.483 | 0.943 | 0.22706 |
| JFBM49455 | 3 | 72.25 | Copepoda | 0.357 | 0.751 | 0.11745 |
| JFBM49455 | 3 | 72.25 | Cladocera | 0.332 | 0.58 | 0.09848 |
| JFBM49455 | 3 | 72.25 | Insecta terrestrial | 0.707 |  | 3.10759 |
| JFBM49456 | 1 | 49.17 | Insecta terrestrial | 0.893 |  | 4.66441 |
| JFBM49456 | 1 | 49.17 | Insecta terrestrial | 0.906 |  | 4.77322 |
| JFBM49456 | 1 | 49.17 | Insecta terrestrial | 0.584 |  | 2.07808 |
| JFBM49456 | 1 | 49.17 | Insecta terrestrial | 0.891 |  | 4.64767 |
| JFBM49456 | 1 | 49.17 | Insecta terrestrial | 0.631 |  | 2.47147 |
| JFBM49456 | 1 | 49.17 | Insecta terrestrial | 1.192 |  | 7.16704 |
| JFBM49456 | 1 | 49.17 | Insecta terrestrial | 0.956 |  | 5.19172 |
| JFBM49456 | 1 | 49.17 | Insecta terrestrial | 0.587 |  | 2.10319 |
| JFBM49456 | 1 | 49.17 | Insecta terrestrial | 1.2 |  | 7.234 |
| JFBM49456 | 1 | 49.17 | Insecta terrestrial | 0.857 |  | 4.36309 |
| JFBM49456 | 1 | 49.17 | Insecta terrestrial | 1.063 |  | 6.08731 |
| JFBM49456 | 1 | 49.17 | Insecta terrestrial | 0.955 |  | 5.18335 |
| JFBM49456 | 1 | 49.17 | Insecta terrestrial | 1.46 |  | 9.4102 |
| JFBM49456 | 1 | 49.17 | Insecta terrestrial | 1.018 |  | 5.71066 |
| JFBM49456 | 1 | 49.17 | Insecta larvae aquatic | 0.858 |  | 4.37146 |
| JFBM49457 | 2 | 137.57 | Fish *Hypopthalmichthys* | 9.614 | 52.843 | 2107.9 |
| JFBM49457 | 7 | 141.4 | Fish | 6.317 |  | 953.95 |
| JFBM49457 | 11 | 90.46 | Insecta larvae aquatic | 1.228 |  | 7.46836 |
| JFBM49457 | 11 | 90.46 | Insecta larvae aquatic | 0.866 |  | 4.43842 |
| JFBM49457 | 11 | 90.46 | Insecta larvae aquatic | 0.886 |  | 4.60582 |
| JFBM49457 | 11 | 90.46 | Insecta larvae aquatic | 0.758 |  | 3.53446 |
| JFBM49457 | 11 | 90.46 | Insecta larvae aquatic | 0.826 |  | 4.10362 |
| JFBM49457 | 11 | 90.46 | Insecta larvae aquatic | 0.816 |  | 4.01992 |
| JFBM49457 | 11 | 90.46 | Insecta larvae aquatic | 0.786 |  | 3.76882 |
| JFBM49457 | 11 | 90.46 | Insecta larvae aquatic | 0.903 |  | 4.74811 |
| JFBM49457 | 11 | 90.46 | Insecta larvae aquatic | 0.543 |  | 1.73491 |
| JFBM49457 | 12 | 73.42 | Insecta larvae aquatic | 1.155 |  | 6.85735 |
| JFBM49457 | 12 | 73.42 | Insecta larvae aquatic | 0.911 |  | 4.81507 |
| JFBM49457 | 12 | 73.42 | Insecta larvae aquatic | 0.675 |  | 2.83975 |
| JFBM49457 | 12 | 73.42 | Insecta larvae aquatic | 0.743 |  | 3.40891 |
| JFBM49457 | 12 | 73.42 | Insecta larvae aquatic | 0.608 |  | 2.27896 |
| JFBM49457 | 12 | 73.42 | Insecta larvae aquatic | 0.483 |  | 1.23271 |
| JFBM49457 | 12 | 73.42 | Insecta larvae aquatic | 0.997 |  | 5.53489 |
| JFBM49457 | 12 | 73.42 | Insecta larvae aquatic | 0.684 |  | 2.91508 |
| JFBM49457 | 12 | 73.42 | Insecta larvae aquatic | 0.663 |  | 2.73931 |
| JFBM49457 | 12 | 73.42 | Insecta larvae aquatic | 0.68 |  | 2.8816 |
| JFBM49457 | 12 | 73.42 | Insecta larvae aquatic | 0.454 |  | 0.98998 |
| JFBM49457 | 12 | 73.42 | Insecta larvae aquatic | 0.554 |  | 1.82698 |
| JFBM49457 | 12 | 73.42 | Insecta larvae aquatic | 0.705 |  | 3.09085 |
| JFBM49457 | 12 | 73.42 | Ostracoda | 0.413 | 0.614 | 0.12857 |
| JFBM49459 | 1 | 155.71 | Fish *Dorosoma* | 10.794 | 40.423 | 2544.64027 |
| JFBM49459 | 1 | 155.71 | Fish | 8.738 |  | 1801.3 |
| JFBM49459 | 1 | 155.71 | Fish | 11.432 |  | 2744.2 |
| JFBM49459 | 2 | 116.79 | Fish *Dorosoma* | 6.995 |  | 1191.25 |
| JFBM49459 | 2 | 116.79 | Insecta larvae aquatic | 0.599 |  | 2.20363 |
| JFBM49459 | 2 | 116.79 | Insecta terrestrial | 1.375 |  | 8.69875 |
| JFBM49459 | 2 | 116.79 | Insecta terrestrial | 1.197 |  | 7.20889 |
| JFBM49459 | 2 | 116.79 | Insecta terrestrial | 1.045 |  | 5.93665 |
| JFBM49459 | 2 | 116.79 | Insecta terrestrial | 2.374 |  | 17.06038 |
| JFBM49459 | 2 | 116.79 | Insecta terrestrial | 1.296 |  | 8.03752 |
| JFBM49459 | 2 | 116.79 | Insecta terrestrial | 2.424 |  | 17.47888 |
| JFBM49459 | 2 | 116.79 | Insecta terrestrial | 1.194 |  | 7.18378 |
| JFBM49459 | 3 | 64.72 | Insecta larvae aquatic | 1.122 |  | 6.58114 |
| JFBM49459 | 3 | 64.72 | Insecta terrestrial | 0.275 |  | 0.2477 |
| JFBM49459 | 3 | 64.72 | Insecta terrestrial | 0.231 |  | 0.23371 |
| JFBM49459 | 3 | 64.72 | Insecta terrestrial | 0.456 |  | 1.00672 |
| JFBM49459 | 3 | 64.72 | Insecta terrestrial | 0.591 |  | 2.13667 |
| JFBM49459 | 3 | 64.72 | Insecta terrestrial | 0.431 |  | 0.79747 |
| JFBM49459 | 3 | 64.72 | Insecta terrestrial | 0.366 |  | 0.25342 |
| JFBM49459 | 3 | 64.72 | Insecta terrestrial | 0.276 |  | 0.24801 |
| JFBM49459 | 3 | 64.72 | Insecta terrestrial | 0.356 |  | 0.16972 |
| JFBM49459 | 3 | 64.72 | Insecta terrestrial | 0.449 |  | 0.94813 |
| JFBM49459 | 3 | 64.72 | Insecta terrestrial | 0.65 |  | 2.6305 |
| JFBM49459 | 3 | 64.72 | Insecta terrestrial | 0.455 |  | 0.99835 |
| JFBM49459 | 3 | 64.72 | Insecta terrestrial | 0.855 |  | 4.34635 |
| JFBM49459 | 3 | 64.72 | Insecta terrestrial | 1.373 |  | 8.68201 |
| JFBM49459 | 3 | 64.72 | Insecta terrestrial | 0.787 |  | 3.77719 |
| JFBM49459 | 3 | 64.72 | Insecta terrestrial | 0.572 |  | 1.97764 |
| JFBM49459 | 3 | 64.72 | Insecta terrestrial | 0.34 |  | 0.0358 |
| JFBM49459 | 3 | 64.72 | Insecta terrestrial | 0.298 |  | 0.255 |
| JFBM49459 | 3 | 64.72 | Insecta terrestrial | 0.313 |  | 0.25977 |
| JFBM49459 | 3 | 64.72 | Insecta terrestrial | 0.514 |  | 1.49218 |
| JFBM49459 | 3 | 64.72 | Insecta terrestrial | 0.825 |  | 4.09525 |
| JFBM49459 | 3 | 64.72 | Insecta terrestrial | 0.619 |  | 2.37103 |
| JFBM49459 | 3 | 64.72 | Insecta terrestrial | 0.749 |  | 3.45913 |
| JFBM49459 | 3 | 64.72 | Insecta terrestrial | 0.69 |  | 2.9653 |
| JFBM49459 | 3 | 64.72 | Insecta terrestrial | 0.415 |  | 0.66355 |
| JFBM49459 | 3 | 64.72 | Insecta terrestrial | 0.515 |  | 1.50055 |
| JFBM49459 | 3 | 64.72 | Insecta terrestrial | 0.719 |  | 3.20803 |
| JFBM49459 | 3 | 64.72 | Insecta terrestrial | 0.647 |  | 2.60539 |
| JFBM49459 | 3 | 64.72 | Insecta terrestrial | 0.654 |  | 2.66398 |
| JFBM49459 | 3 | 64.72 | Insecta terrestrial | 0.45 |  | 0.9565 |
| JFBM49459 | 3 | 64.72 | Insecta terrestrial | 0.541 |  | 1.71817 |
| JFBM49459 | 3 | 64.72 | Insecta terrestrial | 0.441 |  | 0.88117 |
| JFBM49459 | 3 | 64.72 | Insecta terrestrial | 0.46 |  | 1.0402 |
| JFBM49459 | 3 | 64.72 | Insecta terrestrial | 0.602 |  | 2.22874 |
| JFBM49459 | 3 | 64.72 | Insecta terrestrial | 0.41 |  | 0.6217 |
| JFBM49459 | 3 | 64.72 | Insecta terrestrial | 0.367 |  | 0.26179 |
| JFBM49459 | 3 | 64.72 | Insecta terrestrial | 0.428 |  | 0.77236 |
| JFBM49459 | 4 | 74.64 | Insecta larvae aquatic | 1.021 | 6.603 | 5.73577 |
| JFBM49459 | 4 | 74.64 | Insecta larvae aquatic | 1.334 |  | 8.35558 |
| JFBM49459 | 4 | 74.64 | Insecta larvae aquatic | 0.467 |  | 1.09879 |
| JFBM49459 | 4 | 74.64 | Insecta larvae aquatic | 0.937 |  | 5.03269 |
| JFBM49459 | 4 | 74.64 | Insecta larvae aquatic | 0.819 |  | 4.04503 |
| JFBM49459 | 4 | 74.64 | Insecta larvae aquatic | 0.832 |  | 4.15384 |
| JFBM49459 | 4 | 74.64 | Insecta larvae aquatic | 0.761 |  | 3.55957 |
| JFBM49459 | 4 | 74.64 | Insecta larvae aquatic | 0.81 | 9.253 | 8.50434 |
| JFBM49459 | 4 | 74.64 | Insecta larvae aquatic | 1.348 | 7.936 | 6.46076 |
| JFBM49459 | 5 | 57.17 | Copepoda | 0.351 |  | 0.27185 |
| JFBM49459 | 5 | 57.17 | Insecta larvae aquatic | 1.09 | 7.365 | 10.34408 |
| JFBM49459 | 5 | 57.17 | Insecta larvae aquatic | 1.232 |  | 7.50184 |
| JFBM49459 | 5 | 57.17 | Insecta larvae aquatic | 0.749 |  | 3.45913 |
| JFBM49459 | 5 | 57.17 | Insecta larvae aquatic | 0.349 | 3.873 | 0.62469 |
| JFBM49459 | 5 | 57.17 | Insecta larvae aquatic | 1.342 |  | 8.42254 |
| JFBM49459 | 5 | 57.17 | Insecta larvae aquatic | 0.607 |  | 2.27059 |
| JFBM49459 | 5 | 57.17 | Insecta larvae aquatic | 1.963 |  | 13.62031 |
| JFBM49459 | 5 | 57.17 | Insecta larvae aquatic | 1.28 |  | 7.9036 |
| JFBM49459 | 5 | 57.17 | Insecta larvae aquatic | 1.273 |  | 7.84501 |
| JFBM49459 | 5 | 57.17 | Insecta larvae aquatic | 0.951 |  | 5.14987 |
| JFBM49459 | 5 | 57.17 | Insecta larvae aquatic | 1.087 |  | 6.28819 |
| JFBM49459 | 5 | 57.17 | Insecta larvae aquatic | 1.097 |  | 6.37189 |
| JFBM49459 | 5 | 57.17 | Insecta larvae aquatic | 0.403 |  | 0.56311 |
| JFBM49459 | 6 | 60.29 | Insecta larvae aquatic | 1.19 |  | 7.1503 |
| JFBM49459 | 6 | 60.29 | Insecta larvae aquatic | 1.105 |  | 6.43885 |
| JFBM49459 | 6 | 60.29 | Insecta larvae aquatic | 1.388 |  | 8.80756 |
| JFBM49459 | 6 | 60.29 | Insecta larvae aquatic | 1.083 |  | 6.25471 |
| JFBM49459 | 6 | 60.29 | Insecta larvae aquatic | 1.41 | 6.602 | 9.74838 |
| JFBM49459 | 6 | 60.29 | Insecta larvae aquatic | 1.26 |  | 7.7362 |
| JFBM49459 | 6 | 60.29 | Insecta larvae aquatic | 0.938 |  | 5.04106 |
| JFBM49459 | 6 | 60.29 | Insecta larvae aquatic | 1.031 |  | 5.81947 |
| JFBM49459 | 6 | 60.29 | Insecta larvae aquatic | 1.152 |  | 6.83224 |
| JFBM49459 | 6 | 60.29 | Insecta larvae aquatic | 1.225 |  | 7.44325 |
| JFBM49459 | 6 | 60.29 | Insecta terrestrial | 0.587 |  | 2.10319 |
| JFBM49459 | 6 | 60.29 | Insecta terrestrial | 0.322 |  | 0.26263 |
| JFBM49459 | 6 | 60.29 | Insecta terrestrial | 0.984 |  | 5.42608 |
| JFBM49459 | 6 | 60.29 | Insecta terrestrial | 0.782 |  | 3.73534 |
| JFBM49459 | 6 | 60.29 | Insecta terrestrial | 0.599 |  | 2.20363 |
| JFBM49459 | 6 | 60.29 | Insecta terrestrial | 0.525 |  | 1.58425 |
| JFBM49459 | 6 | 60.29 | Insecta terrestrial | 0.586 |  | 2.09482 |
| JFBM49459 | 6 | 60.29 | Insecta terrestrial | 0.761 |  | 3.55957 |
| JFBM49459 | 6 | 60.29 | Insecta terrestrial | 0.571 |  | 1.96927 |
| JFBM49459 | 6 | 60.29 | Insecta terrestrial | 0.929 |  | 4.96573 |
| JFBM49459 | 6 | 60.29 | Insecta terrestrial | 0.405 |  | 0.57985 |
| JFBM49459 | 6 | 60.29 | Insecta terrestrial | 0.366 |  | 0.25342 |
| JFBM49459 | 6 | 60.29 | Insecta terrestrial | 0.617 |  | 2.35429 |
| JFBM49459 | 7 | 47.85 | Insecta terrestrial | 0.295 |  | 0.25405 |
| JFBM49459 | 7 | 47.85 | Insecta terrestrial | 0.32 |  | 0.262 |
| JFBM49459 | 7 | 47.85 | Insecta terrestrial | 0.345 |  | 0.07765 |
| JFBM49459 | 7 | 47.85 | Insecta terrestrial | 0.416 |  | 0.67192 |
| JFBM49459 | 7 | 47.85 | Insecta terrestrial | 0.413 |  | 0.64681 |
| JFBM49459 | 7 | 47.85 | Insecta terrestrial | 0.265 |  | 0.24452 |
| JFBM49459 | 7 | 47.85 | Insecta terrestrial | 0.423 |  | 0.73051 |
